# Immunogenicity of a novel Clade B HIV-1 vaccine combination: Results of phase 1 randomized placebo controlled trial of an HIV-1 GM-CSF-expressing DNA prime with a modified vaccinia Ankara vaccine boost in healthy HIV-1 uninfected adults

**DOI:** 10.1371/journal.pone.0179597

**Published:** 2017-07-20

**Authors:** Susan P. Buchbinder, Nicole A. Grunenberg, Brittany J. Sanchez, Kelly E. Seaton, Guido Ferrari, M. Anthony Moody, Nicole Frahm, David C. Montefiori, Christine M. Hay, Paul A. Goepfert, Lindsey R. Baden, Harriet L. Robinson, Xuesong Yu, Peter B. Gilbert, M. Juliana McElrath, Yunda Huang, Georgia D. Tomaras, on behalf of the HIV Vaccine Trials Network (HVTN) 094 Study Group

**Affiliations:** 1 Bridge HIV, San Francisco Department of Public Health, San Francisco, California, United States of America; 2 Departments of Medicine, Epidemiology and Biostatistics, University of California, San Francisco, California, United States of America; 3 Vaccine and Infectious Disease Division, Fred Hutchinson Cancer Research Center, Seattle, Washington, United States of America; 4 Department of Surgery, Duke Human Vaccine Institute, Durham, North Carolina, United States of America; 5 Department of Global Health, University of Washington, Seattle, Washington, United States of America; 6 Department of Medicine, University of Rochester Medical Center, Rochester, New York, United States of America; 7 Department of Medicine, University of Alabama, Birmingham, Alabama, United States of America; 8 Department of Medicine, Brigham and Women’s Hospital, Boston, Massachusetts, United States of America; 9 GeoVax Labs, Inc., Smyrna, Georgia, United States of America; 10 Department of Biostatistics, University of Washington, Seattle, Washington, United States of America; 11 Department of Medicine, University of Washington, Seattle, Washington, United States of America; Rush University, UNITED STATES

## Abstract

**Background:**

A phase 1 trial of a clade B HIV vaccine in HIV-uninfected adults evaluated the safety and immunogenicity of a DNA prime co-expressing GM-CSF (Dg) followed by different numbers and intervals of modified vaccinia Ankara Boosts (M). Both vaccines produce virus-like particles presenting membrane-bound Env.

**Methods:**

Four US sites randomized 48 participants to receiving 1/10^th^ the DNA dose as DgDgMMM given at 0, 2, 4, 6 and 8 months, or full dose DgDgM_M or DgDgMM_M regimens, given at 0, 2, 4, and 8 months, and 0, 2, 4, 6, and 10 months, respectively. Peak immunogenicity was measured 2 weeks post-last vaccination.

**Results:**

All regimens were well tolerated and safe. Full dose DgDgM_M and DgDgMM_M regimens generated Env-specific IgG to HIV-1 Env in >90%, IgG3 in >80%, and IgA in <20% of participants. Responses to gp140 and gp41 targets were more common and of higher magnitude than to gp120 and V1V2. The gp41 antibody included reactivity to the conserved immunodominant region with specificities known to mediate virus capture and phagocytosis and did not cross-react with a panel of intestinal flora antigens. The 3^rd^ dose of MVA increased the avidity of elicited antibody (7.5% to 39%), the ADCC response to Bal gp120 (14% to 64%), and the one-year durability of the IgG3 responses to gp41 by 4-fold (13% vs. 3.5% retention of peak response). The co-expressed GM-CSF did not enhance responses over those in trials testing this vaccine without GM-CSF.

**Conclusion:**

This DNA/MVA prime-boost regimen induced durable, functional humoral responses that included ADCC, high antibody avidity, and Env IgG1 and IgG3 binding responses to the immunodominant region of gp41. The third, spaced MVA boost improved the overall quality of the antibody response. These products without co-expressed GM-CSF but combined with protein boosts will be considered for efficacy evaluation.

**Trial registration:**

ClinicalTrials.gov NCT01571960

## Introduction

The RV144 vaccine reigmen of canarypox (ALVAC) and rgp120 subunit protein (AIDSVAX) demonstrated 31% efficacy against HIV acquisition among 16,000 Thai women and men when the clinical trial concluded at 42 months [1]. It is the only one of the six preventive HIV-1 vaccine efficacy trials conducted to date [1–6] that has demonstrated protection against HIV acquisition, albeit of modest efficacy and durability [7, 8]. Immune correlates analyses identified IgG antibodies to the V1V2 region of HIV-1 envelope as a significant correlate with decreased HIV-1 acquisition [9, 10]; the results from a virus sieve analysis were consistent with immune pressure on the V2 site [11]. Although the V1V2 IgG antibodies did not broadly neutralize panels of globally circulating tier 2 viruses, they did have other functional attributes, mediating antibody dependent cellular cytotoxicity (ADCC) against tier 1 and 2 viruses [12, 13]. Moreover, IgG3 responses correlated with functional antibody-mediated responses and decreased HIV-1 risk of infection [14]. In contrast, HIV-1 Env IgA responses recognizing the Env C1 region were associated with increased risk of infection and appeared to interfere with protective immune responses, possibly through binding that blocked ADCC function [9, 15]. New vaccine regimens that elicit stronger and more durable protective responses, possibly including ADCC, IgG and IgG3 to Env, with low serum IgA, may be able to protect against sexual HIV-1 transmission, and should be evaluated.

The Clade B HIV-1 vaccines furthest along in development are the GeoVax (Smyrna, GA) DNA (pGA2/JS7) vaccine and recombinant modified vaccinia Ankara poxvirus (MVA/HIV62B) vaccine. A unique property of these vaccines is that both produce non-infectious virus-like particles (VLPs) displaying trimeric membrane-bound forms of the HIV-1 envelope glycoprotein. A previous Phase 2 study of these vaccines demonstrated a predominant gp41 IgG response when two DNA priming doses were followed by two MVA boosting doses; giving three MVA doses without the DNA prime improved the gp120 IgG response [16]. However, this earlier trial did not evaluate IgG subclasses, fine specificity of the IgG response or ADCC, immune responses found to be associated with reduced HIV acquisition in RV144. Non-human primate SIV prototypes of the JS7 DNA and MVA vaccines in a prime-boost regimen of two DNA immunizations followed by two MVA immunizations (DDMM) resulted in 81% and 76% reductions in the per challenge risk of intra-rectal infection over the first 6 and 12 weekly challenges, respectively [17]).

Therefore, the HVTN launched a novel Phase 1 trial of the GeoVax DNA prime, MVA boost regimen, to advance understanding and enhance immunogenicity of these two vaccines used in combination. The trial compared 2 vs. 3 MVA boosts after 2 DNA primes, and a 2- vs. 4-month pause prior to the final MVA dose based on improved immunogenicity with this pause in influenza vaccination [18]. Because of preclinical data suggesting improved protection with co-expression of GM-CSF in the JS7 DNA pGA2/JS7 vaccine [19], the HVTN 094 trial substituted a GM-CSF co-expressing JS7 DNA vaccine, GEO- D03 (Dg), in these prime-boost regimens.

## Materials and methods

### Study volunteers

Forty-eight HIV-uninfected adult male and female vaccinia-naïve participants were enrolled at HVTN Clinical Research Sites in 4 US cities (Birmingham, Boston, Rochester, and San Francisco) from May through November 2012. Subjects were required to meet the following criteria for enrollment: age 18–50 years; good general health on the basis of medical history, physical examination, laboratory tests, and electrocardiogram (ECG) findings; and assessed as low risk for HIV infection. Pregnant women were excluded, and volunteers who could become pregnant agreed to consistently use effective contraception. All participants provided written informed consent.

### Vaccines

The GEO-D03 co-expresses GM-CSF and non-infectious VLPs from clade B HIV-HXB2/BH10 (Gag-Pol) and HIV-ADA sequences (gp120/gp41 cleavage site intact), from a single transcript by subgenomic splicing [20–22]. Approximately 235 ng of GM-CSF are produced at 48 hours post transient transfection of 1x10^6^ HEK293T cells. Modified Vaccinia Ankara MVA/HIV62B (MVA62B) encodes HIV-1 VLP from the same HIV sequences mutated for expression by a poxvirus as GEO-D03 [23]. In MVA62B, the ADA Env gene is truncated for the 115 C-terminal amino acids of the endodomain of gp41 to increase membrane display of Env and the stability of the vaccine insert during manufacture [23]. Vaccines were delivered intramuscularly by needle injection at a final volume of 1 mL into the deltoid region.

### Study design

HVTN 094 was a randomized, double-blind, placebo-controlled trial among participants considered to be at low risk for HIV infection (clinical trials registration NCT01571960, S1 Protocol). The institutional review boards or ethics committees for each site provided initial and ongoing approvals and review of the research, as did the coordinating Fred Hutchinson Cancer Research Center IRB. All participants provided written informed consent. Initially 12 participants (10 vaccinees; 2 placebo recipients) were randomized and enrolled into Group 1, receiving low dose GEO-D03 DNA (0.3mg), and sequentially 36 (30 vaccinees; 6 placebo recipients) were randomized in blocks of 6 and enrolled into Group 2, receiving full dose GEO-D03 DNA (3mg). Each Group was to receive GEO-D03 DNA vaccine at months 0 and 2 and three doses of MVA62B at months 4, 6 and 8. After the study was fully enrolled and prior to any Group 2 participants receiving the second MVA62B/placebo injection, the original Group 2 was divided into 2 new groups of 18 participants each: new Group 2 received full-dose DgDgMM_M at months 0, 2, 4, 6, and 10, and Group 3 received full-dose DgDgM_M at months 0, 2, 4, and 8, to evaluate immunogenicity with a 4-month pause before the final MVA dose (S1 CONSORT checklist). To maintain the integrity of the randomization and blinding within the new groups, the first 18 of the original 36 Group 2 participants were placed into the revised Group 2, while the last 18 participants who enrolled into the original Group 2 were placed into Group 3. Due to the proximity of timing of enrollments along the split line of the original Group 2 participants, the vaccinee: placebo assignment ratio became 16:2 in the new Group 2 and 14:4 in Group 3. Follow-up for the 1/10^th^ dose group and for each full-dose group was 6 months and 12 months, respectively after their last vaccination: month 14 for Group 1, month 22 for new Group 2 and month 20 for Group 3 during the main study. Participants were also contacted annually by telephone for completion of health questionnaires for 3 years following enrollment.

### Vaccine safety and reactogenicity evaluation

Safety evaluations included physical examinations, standard clinical chemistry, hematological tests, and urinalysis. Cardiac safety monitoring was performed via cardiac symptoms assessments at each MVA62B/placebo vaccination- and post-vaccination visit. Post-vaccination chest symptoms suggestive of possible myo/pericarditis were evaluated with cardiac troponin levels and a 12-lead ECG, and findings interpreted by a central ECG laboratory. Local and systemic reactogenicity were assessed for 3 days following each vaccination or until resolution. Adverse events were graded for each participant until completion of follow-up according to Division of AIDS Table for Grading the Severity of Adult and Pediatric Adverse Events, Version 1.0, December 2004 (Clarification August 2009). Immune responses were measured at baseline, 2 weeks after each MVA/placebo dose, and 6- (all Groups) and 9- or 12-months (Groups 2 and 3) after the last MVA/placebo dose.

### GM-CSF and anti-GM-CSF antibody levels

Serum for measuring GM-CSF levels was collected pre- and 3 and 7 days post- each DNA vaccination. Serum for measuring anti-GM-CSF binding antibodies was collected at baseline before the first DNA vaccination and at 2 weeks after each DNA and first MVA vaccinations. Levels of GM-CSF were measured using the Quantikine HS GM-CSF Immunoassay (R&D Systems).

### Immune response assays

#### Binding antibody

Serum HIV-1-specific IgA and IgG responses (1:50 dilution) and IgG1- IgG4 subclass responses (1:40 dilution) were performed by binding antibody multiplex assays (BAMA) under cGLP-compliant conditions using 21CFR Part 11 compliant software. The readout is expressed as mean fluorescence intensity (MFI) [16, 24–26] against the following HIV -1 envelope proteins: ADA gp120 (MyBioSource, San Diego, CA), B.HXB/BaL_120 AVI, Con 6 gp120/B, ConS gp140 CFI, gp41 subtype B (Immunodiagnostics), gp70_B.CaseA_V1/V2 (V1/V2) (Env proteins provided by Drs. Liao and Haynes, Duke University)[27–29] and p24 Gag protein (BD Biosciences). Consensus Clade B gp41 immunodominant (ID) epitope in tetramer form (Bcon03 ID epitope tetramer) was produced by Dr. M.A. Moody [30]. In addition, serum IgG responses against the microbiome were assessed for aerobic and anaerobic antigens (provided by Drs. M.A. Moody, B.F. Haynes, Duke) as previously described [31].

#### Antibody avidity

The affinity maturation of the vaccine-elicited antibody responses to the immunodominant region of gp41 (Bcon03 ID epitope tetramer) was measured by an avidity index assay using the BAMA described above with the following modifications. Avidity index is measured by inclusion of a denaturation step (treating the samples with 0.1 M Na-citrate, pH3.0) and comparing the MFI in the treated well vs. MFI in the untreated well. Untreated wells contain Phosphate Buffered Saline (PBS) during the avidity incubation step. Avidity index (AI) is defined as 100*MFI in the treated divided by MFI in the untreated well [32, 33].

#### ADCC-GranToxiLux (ADCC-GTL) assay

Antibody dependent cellular cytotoxic activity mediated by the participants’ plasma/sera was detected according to our modification of the previously described GranToxiLux (GTL) cell-mediated cytotoxicity procedure [34]. The CEM.NKRCCR5 target cells were coated with recombinant gp120 HIV-1 protein derived from Env of HIV-1 BaL (Genebank No. M68893; provided by Dr. Liao-Duke Human Vaccine Institute), ADA (Genebank No. M60472; MyBioSource), and Con6 (provided by Dr. Liao-Duke Human Vaccine Institute). The assay readout is the percent of antigen-coated target cells taking up Granzyme B. A positive response is defined if the peak percent Granzyme B activity across six dilutions is greater than or equal to 8%.

#### Intracellular cytokine staining (ICS)

A validated intracellular cytokine staining (ICS) assay with detection by flow cytometry was used to examine HIV-1-specific CD4+ and CD8+ T-cell responses as previously described [35]. Previously cryopreserved PBMC were stimulated with peptide pools based on HIV-1 global potential T-cell epitopes (PTE) for Env, Gag, Pol, Rev, and Vpu. The primary measure is T cells producing IL-2 and/or IFN-γ. A 12- color staining panel including several other functional markers was used as previously described [36].

#### Vaccine-induced seropositivity (VISP)

End of study HIV diagnostics were performed on samples drawn 6 months after the last vaccination to evaluate ongoing seroreactivity to licensed HIV-1 antibody tests. Four enzyme-linked immunosorbent assays (ELISAs) were used: Abbott Architect HIV Ag/Ab Combo, Abbott Prism, BioRad GS HIV Combo Ag/Ab EIA, and Multi-spot HIV-1/HIV-2 Rapid Test. Western blot testing was also performed, but this test is no longer CDC-recommended and therefore these results are not included in the summary VISP results. Verification of HIV-1 uninfected status on reactive samples was confirmed with negative HIV nucleic acid testing.

#### Neutralizing antibodies

Neutralizing antibodies were measured as a function of reductions in luciferase (Luc) reporter gene expression after a single round of infection in TZM-bl cells [37, 38]. TZM-bl cells (also called JC57BL-13) were obtained from the NIH AIDS Research and Reference Reagent Program, as contributed by John Kappes and Xiaoyun Wu. Briefly, a pre-titrated dose of virus was incubated with serial 3-fold dilutions of test sample in duplicate in a total volume of 150 μl for 1 hr at 37°C in 96-well flat-bottom culture plates. Freshly trypsinized cells (10,000 cells in 100 μl of growth medium containing 75 μg/ml DEAE dextran) were added to each well. One set of 8 control wells received cells + virus (virus control) and another set received cells only (background control). After 48 hours of incubation, 100 μl of cells was transferred to a 96-well black solid plate (Costar) for measurements of luminescence using the Britelite Luminescence Reporter Gene Assay System (PerkinElmer Life Sciences). Assay stocks of molecularly cloned Env- pseudotyped viruses were prepared by transfection in 293T/17 cells (American Type Culture Collection) and titrated in TZM-bl cells as described [37]. This assay has been formally optimized and validated [38] and was performed in compliance with Good Clinical Laboratory Practices, including participation in a formal proficiency testing program [39]. Additional information on the assay and all supporting protocols may be found at: http://www.hiv.lanl.gov/content/nab-reference-strains/html/home.htm.

### Statistical methods

The sample size of the study was determined to provide a reasonable precision in the assessement of the primary safety and immuenogenicity endpoints (See details in Section 6.1 of the Protocol). The randomization allocation sequence was obtained by computer-generated random numbers and provided to each clinical site through the HVTN statistics and data monitoring center’s web-based randomization system. At each site, the pharmacist with primary responsibility for dispensing study products was charged with maintaining security of the treatment assignments. Participants and site staff (except for site pharmacists) were blinded as to participant treatment arm assignments. All randomized participants were included in the safety analyses. The number and percentage of participants experiencing each adverse event or reactogenicity symptom were tabulated by severity and vaccination regimens. All randomized participants with reliable assay data based on blood draw dates within the allowable visit window were included in the immunogenicity analysis. Fisher’s exact and Wilcoxon rank sum tests were used to compare the response rates and magnitudes, respectively, between two groups. For the comparison of two visits within a group, McNemar’s and Wilcoxon signed rank tests were used to compare the response rates and magnitudes among responders, respectively. Two-sided 95% confidence intervals (CI) for binomial proportions were calculated using the Wilson score method [40]. All tests were two-sided, and differences were considered statistically significant if the p-value <0 .05. For the ICS assay, positivity for a peptide pool was based on comparing the percentage of T cells with positive staining for IL-2 and/ or IFN-γ between the experimental and negative control wells using a one-sided Fisher’s exact test with a p-value cutoff for positivity of 10^−5^, and with a Bonferroni multiplicity adjustment for the number of peptide pools [41]. For the binding antibody and avidity assays, samples were declared to have positive responses if they met three conditions: (1) the MFI values were greater than or equal to the antigen-specific pre-specified cutoffs, (2) the MFI- blank values were greater than 3 times the baseline MFI-blank values, and (3) the MFI values were greater than 3 times the baseline MFI values.

To summarize multiple assay readouts simultaneously, radar plots were used to display the response rate and median response magnitude of each assay readout by vaccine regimen. To assess the relationship between different assay readouts and the groups of observations in a reduced dimensionality, principal component analysis (PCA) biplot was used. PCA was performed on scaled, centered data. In PCA, the original assay readouts were algebraically converted and combined into a new set of uncorrelated composite variables, ranked in order of their contribution to explaining the variation in the data. These new variables were termed “principal components” (PCs). In the biplot, each point represented one participant and each arrow represented one assay readout in the plane defined by the first two PCs, along with the percent of variation explained by each PC.

## Results

### Participant accrual, demographics, and disposition

**Fig 1:**
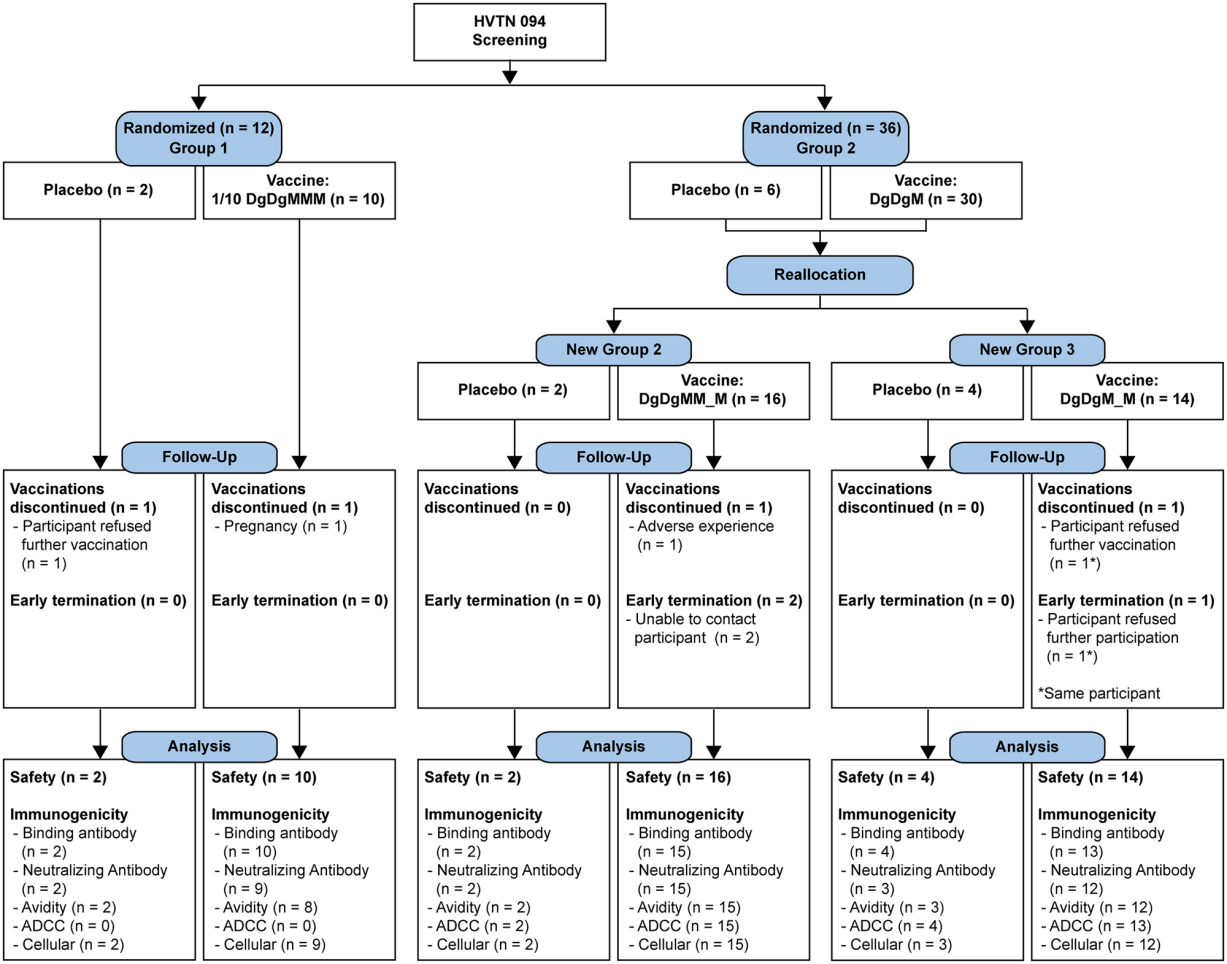
CONSORT flow diagram. Allocation, follow-up, and analysis for HIV Vaccine Trials Network (HVTN) study 094. Abbreviations: 1/10 DgDgMMM, treatment arm receiving two 1/10 doses of DNA followed by three doses of MVA at 0, 2, 4, 6 and 8 months; DgDgMM_M, treatment arm receiving two full doses of DNA followed by three doses of MVA given at 0, 2, 4, 6, and 10 months; DgDgM_M, treatment arm receiving two full doses of DNA followed by two doses of MVA at 0, 2, 4, and 8 months; ADCC, antibody dependent cellular cytotoxicity.

The four US-based HVTN clinical research sites enrolled 12 participants each, for a total of 48 enrollees in the trial. Participants were assigned to placebo or active product in one of three vaccine arms: a 0.3 mg (1/10^th^ dose) DNA and 1x10^8^ TCID50 (full dose) MVA in a DgDgMMM regimen given at 0, 2, 4, 6 and 8 months, or full dose (3 mg DNA, 1x10^8^ TCID50 MVA) in a DgDgM_M or DgDgMM_M regimens, given at 0, 2, 4, and 8 months, and 0, 2, 4, 6, and 10 months, respectively (Fig 1).

Participants were mostly young adults (median age 25 years, 80% aged 30 or younger) and predominantly female (63%). The participants self-identified as white (63%), Hispanic (13%), Black or African-American (10%), Asian (8%), and multi-racial (6%). Demographic characteristics did not differ significantly by randomization arm (S1 Table). All participants received their first vaccine dose (Fig 1); 41 (85%) received all scheduled doses. One participant in the 1/10 DgDgMMM group was found to be pregnant after 2 DNA doses; no further vaccinations were administered and she delivered a healthy baby at term. One participant in the DgDgMM_M group discontinued vaccination because of a complaint of facial numbness 17 hours after her first dose of MVA; further vaccinations were held, although this symptom was considered unrelated to vaccination by the study site investigator. Two additional participants refused vaccination after the 2^nd^ and 3^rd^ vaccine dose, from the DgDgM_M arm and the placebo arm, respectively. Two additional participants from the DgDgMM_M arm terminated study follow-up early. All participants with available data were included in the analyses, regardless of completion of vaccination or the study.

### Safety and reactogenicity

The vaccinations in all arms were well-tolerated, with mild to moderate local reactogenicity (S1 Fig). Only 3 participants, all in the DgDgMM_M arm, reported severe systemic reactogenicity: 2 reported fatigue after the 2^nd^ dose of DNA and 3^rd^ dose of MVA respectively, and 1 reported a severe headache after the first MVA vaccination. All recovered spontaneously within 3 days. Participants receiving full dose vaccine regimens did not have significantly more local or systemic reactogenicity than those in the 1/10 dose arm. Adverse events (AEs) were reported by 94% of participants, with no significant difference in AE reporting between active and placebo groups. Eight participants experienced AEs attributed to the study product by the investigator; all were mild and transient. There were no serious adverse events nor myocarditis or pericarditis events reported by any of the participants.

As GEO-D03 contains GM-CSF DNA, serum levels of GM-CSF and anti-GM-CSF antibodies were measured. Anti-GM-CSF antibodies are known to be present in healthy people at levels of approximately 1 mcg/ml, but are elevated above 10 mcg/ml in patients with pulmonary alveolar proteinosis [42]. The median anti-GM-CSF measurement for all participants post-DNA doses was 0.242 mcg/ml, range from 0.156 to 2.762 mcg/ml, with no significant difference between active arm and placebo recipients. No participants had detectable GM-CSF in serum at any time point throughout the study.

### Immunogenicity

Because the full-dose regimens were safe and tolerable and will likely be considered or modified in future trials, all immunogenicity results presented here compare data within or between the 2 full-dose arms. The effect of the 3^rd^ MVA boost was assessed by comparing responses after the 2^nd^ vs. 3^rd^ dose within the DgDgMM_M arm. To evaluate the effect of a 4- vs. 2-month pause before the last MVA dose, the two study arms were compared after 2 doses of MVA (DgDgM_M vs. after 2 doses in the DgDgMM_M arm). Peak responses were measured 2 weeks after the final MVA dose; durability was compared between the two arms at peak, 6, 9, and 12 months post the last MVA immunization.

**Fig 2:**
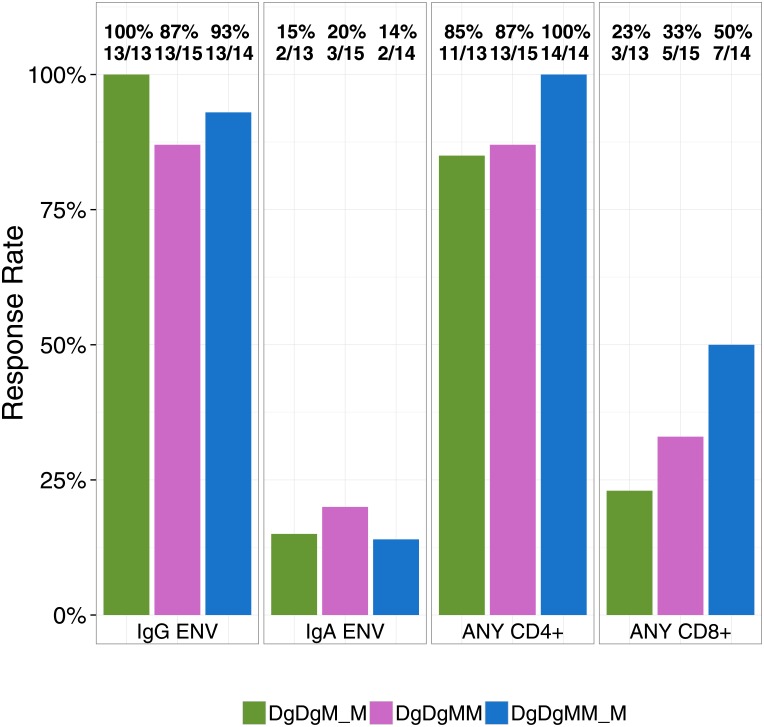
Summary immune responses. Overall peak binding antibody and ICS response rates among vaccine recipients at two weeks after the 2nd and 3rd MVA in the DgDgM_M and DgDgMM_M groups. DgDgM_M refers to immune responses after the last MVA in the DgDgM_M group; DgDgMM and DgDgMM_M refer to immune responses after the 2nd and 3rd MVA in DgDgMM_M group, respectively. A positive response to IgG Env or IgA Env indicates positive responses to at least one ENV antigens measured by the BAMA binding antibody assay; a positive response to ANY CD4+ or ANY CD8+ indicates positive responses to any least one peptide pool and/or cytokine measured by the ICS assay.

Overall rates of humoral and cellular immune responses at the peak time-points are depicted in Fig 2. Both regimens induced high rates of IgG responses to HIV-1 Env gp140 (ConS gp140). IgA response rates were low (<20%) in all vaccine groups. Overall CD4+ T cell response rates were higher than CD8+ T cell responses within each vaccine group, and were not significantly different between groups. There were no positive responses among the placebo recipients in any of these assays.

### HIV-1-specific binding antibody responses

**Fig 3:**
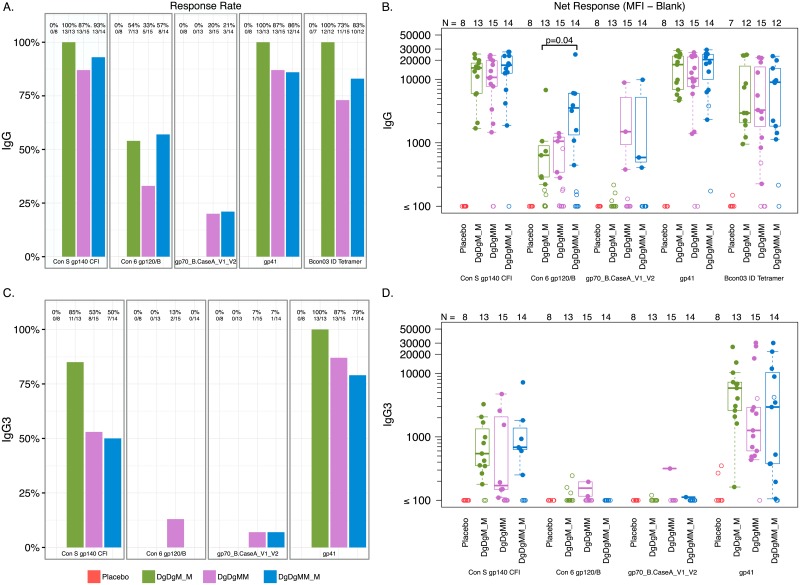
Peak IgG and IgG3 response rates and magnitudes. (A) Peak IgG binding antibody response rates. (B) Peak IgG binding response magnitudes. (C) Peak IgG3 binding antibody response rates. (D) Peak IgG3 binding antibody response magnitudes. Responses are to individual antigens at two weeks after the 2nd and 3rd MVA or placebo in the Placebo, DgDgM_M and DgDgMM_M groups. DgDgM_M refers to immune responses after the last MVA in the DgDgM_M group; DgDgMM and DgDgMM_M refer to immune responses after the 2nd and 3rd MVA in DgDgMM_M group, respectively. Shown are total IgG binding antibody response rates to HIV-1 Env gp140 (ConS gp140), HIV-1 Env gp120 (Con 6 gp120 B), the V1V2 loop, gp41 and the gp41 immunodominant region (IDR).

To further define the specificity of antibody binding, Fig 3A and 3B show total IgG response rates and magnitudes at peak time-points against HIV-1 Env (Con S gp140 and Con 6 gp120/B), V1V2, gp41, and the gp41 immunodominant region (IDR) for each of the study arms. Total IgG rates and magnitude were higher to gp41 and the IDR of gp41 than to gp120 and V1V2 for all study groups. Within the DgDgMM_M group, there was no significant increase in the proportion of responders against any of these antigens after 3 compared with 2 doses of MVA. However, there was a significant increase in the magnitude of response for Con6 gp120/B in the 3- compared to the 2-dose MVA arm (p = 0.04, Fig 3B). There were no significant differences in response rates or magnitudes after 2 doses of MVA comparing a 2- vs. 4-month pause, except for the response rate to the V1V2 target, driven by the lack of IgG response in the DgDgM_M group (Fig 3A).

At the peak time-points, all participants in the 2-MVA dose arm developed IgG3 against gp41, as did 87% and 79% of participants in the 3-dose arm after the 2^nd^ and 3^rd^ MVA doses, respectively (Fig 3C). IgG3 responses to Con6 gp120/B and V1V2 were generally absent; 2/15 participants in the 3-dose arm developed IgG3 against Con6 g120/B after 2- but not 3-MVA doses, and a different participant developed IgG3 against V1V2 after the 2^nd^ and 3^rd^ MVA doses. All response magnitudes were low, with no significant differences between arms (Fig 3D). There were low level gp41 IgG4 responses with no significant gp120 IgG2 or IgG4 responses in any group. IgG response rate and magnitude against other Clade B and C envelope and p24 Gag antigens, as described in the Methods section, are shown in S2 Fig.

**Fig 4:**
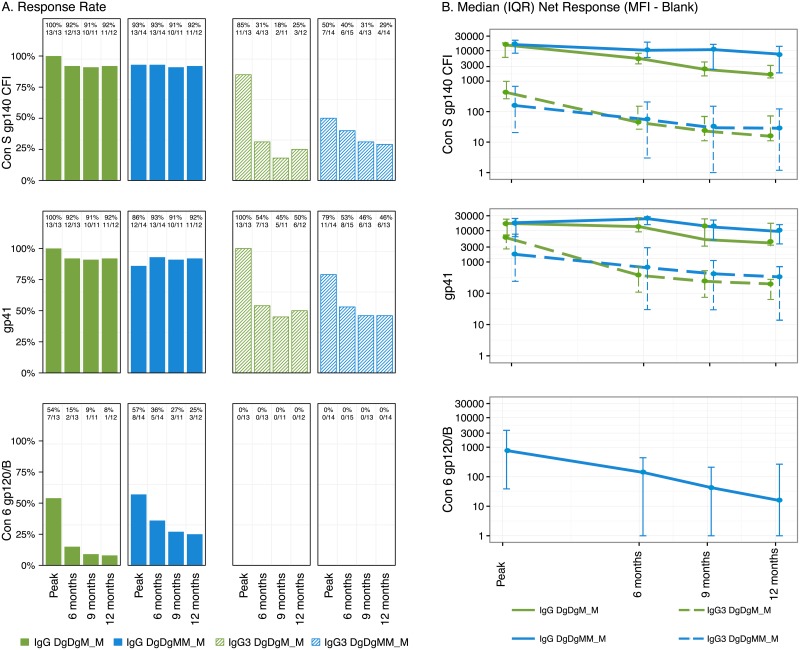
IgG and IgG3 response rates and magnitudes over time. (A) Total IgG and IgG3 binding antibody response rates. (B) Total IgG and IgG3 binding antibody response median magnitudes and inter-quartile range (IQR) responses. Responses among vaccine recipients over time at two weeks, 6 months, 9 months, and 12 months after the last vaccination in the DgDgM_M and DgDgMM_M groups. DgDgM_M refers to immune responses after the last MVA in the DgDgM_M group; DgDgMM and DgDgMM_M refer to immune responses after the 2nd and 3rd MVA in DgDgMM_M group, respectively. For the comparison of two visits within an arm, McNemar’s and Wilcoxon signed rank tests were used to compare the response rates (A) and magnitudes (B) among responders, respectively.

In evaluating IgG durability, the proportion of total IgG responders 12 months post-vaccination remained high (>90%) in both arms of the study against gp140 and gp41 (Fig 4A). The proportion of IgG3 responders to these two antigens declined significantly from peak to 6 months, but then remained stable through 12 months post-vaccination. The proportion of IgG responders to Con6 gp120/B appeared to decline more rapidly in the 2 vs. 3-dose MVA arms, with no appreciable IgG3 to this target (Fig 4A). Three participants generated positive IgG responses to V1V2 at peak, and one remained positive at 12 months post-vaccination in the 3-dose MVA arm, whereas none developed IgG response to V1V2 in the 2-dose MVA arm at any time-point. Only 1 participant in the 3-MVA dose arm generated IgG3 to V1V2 at peak; none of the participants in either arm had subsequent measurable IgG3 to V1V2.

Total IgG and IgG3 magnitudes are shown for both arms through 12 months post last vaccination in Fig 4B. For each individual, the fold-change in antibody was calculated as the magnitude 12 months post-vaccination divided by the magnitude at peak immunogenicity. Participants receiving 3 doses of MVA appeared to maintain higher IgG and IgG3 titers to gp140 and/or gp41 than the 2-dose arm: the median fold change for total IgG to gp140 was 0.47 versus 0.16 (p = 0.15) in the 3- versus 2-dose MVA arms, and the median fold change in IgG3 to gp140 was 0.21 versus 0.06 (p = 0.04), respectively. In addition, the median fold-change for total IgG3 to gp41 was 0.13 versus 0.035 (p = 0.058) in the 3- versus 2-dose MVA arms, respectively. IgA responses to gp140 were minimal for both vaccine regimens, comprising fewer than 20% in each of the groups (Fig 2).

None of the DgDgM_M participants had subsequent measurable IgA against ConS gp140 at 12 months (S3 Fig, panel A), and IgA titers were relatively low at all time points (S3 Fig, Panel B). None of the participants in the DgDgM_M arm generated IgA to gp41 at peak or subsequent time-points; the response rate and magnitude were low in the DgDgMM_M arm. No participants generated detectable levels of IgA to Con6 gp120/B or to V1V2.

#### IgG cross-reactivity with intestinal microbiome antigens

To determine if cross-reactive antibodies to the microbiome were elicited by this vaccine regimen, as previously described for gp41 antibodies [31], we measured circulating binding IgG responses to the microbiome (aerobic and anaerobic). There were no positive responders to either anaerobic or aerobic microbiota in any of the treatment groups using the same positivity methods previously described [31].

#### Functional antibody-mediated responses

**Fig 5:**
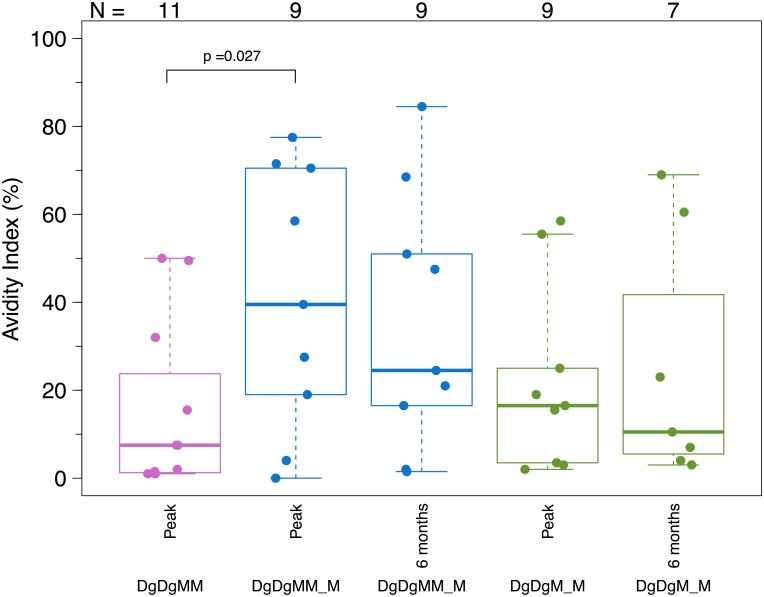
Antibody avidity. Distribution of binding antibody avidity response magnitude among vaccine recipients at two weeks and 6 months after the 2nd and 3rd MVA in the DgDgM_M and DgDgMM_M groups. DgDgM_M refers to immune responses after the last MVA in the DgDgM_M group; DgDgMM and DgDgMM_M refer to immune responses after the 2nd and 3rd MVA in DgDgMM_M group, respectively. Avidity Index (AI) was calculated for the ID epitope tetramer for those with a positive binding response to the ID epitope tetramer and only for samples not saturated (MFI < 23000). Wilcoxon signed rank tests were used to compare AI values among positive responders.

At the peak time-point, the IgG avidity index to the IDR was significantly higher after the third vs. second MVA in the DgDgMM_M arm (median = 39.5% vs. 7.5%, p = 0.027) among IgG responders, but was similar between the 2- vs. 4-month pause (Fig 5). At 6 months after the last MVA dose, the IgG avidity index was similar between the 2- vs. 3-dose MVA arms, with ranges from 1.5% to 84.5% (median = 24.5%) in the DgDgMM_M arm and 3% to 69% (median = 10.5%) in the DgDgM_M arm. Neutralizing antibodies against Tier 1 viruses were generally weak for all but the MN.3 virus (S4 Fig). For MN.3, 15 and 33% of participants responded after 2 MVA doses with 4- and 2-month pause, respectively, and 57% responded after 3 MVA doses. Among positive responders to MN.3, median titers ranged from 55 in the DgDgM_M group to 22 and 29 after the second and third MVA doses in the DgDgMM_M arm. There were no significant differences in response rates or magnitudes between the arms after the second boost, nor for the third MVA dose in the DgDgMM_M arm.

**Fig 6:**
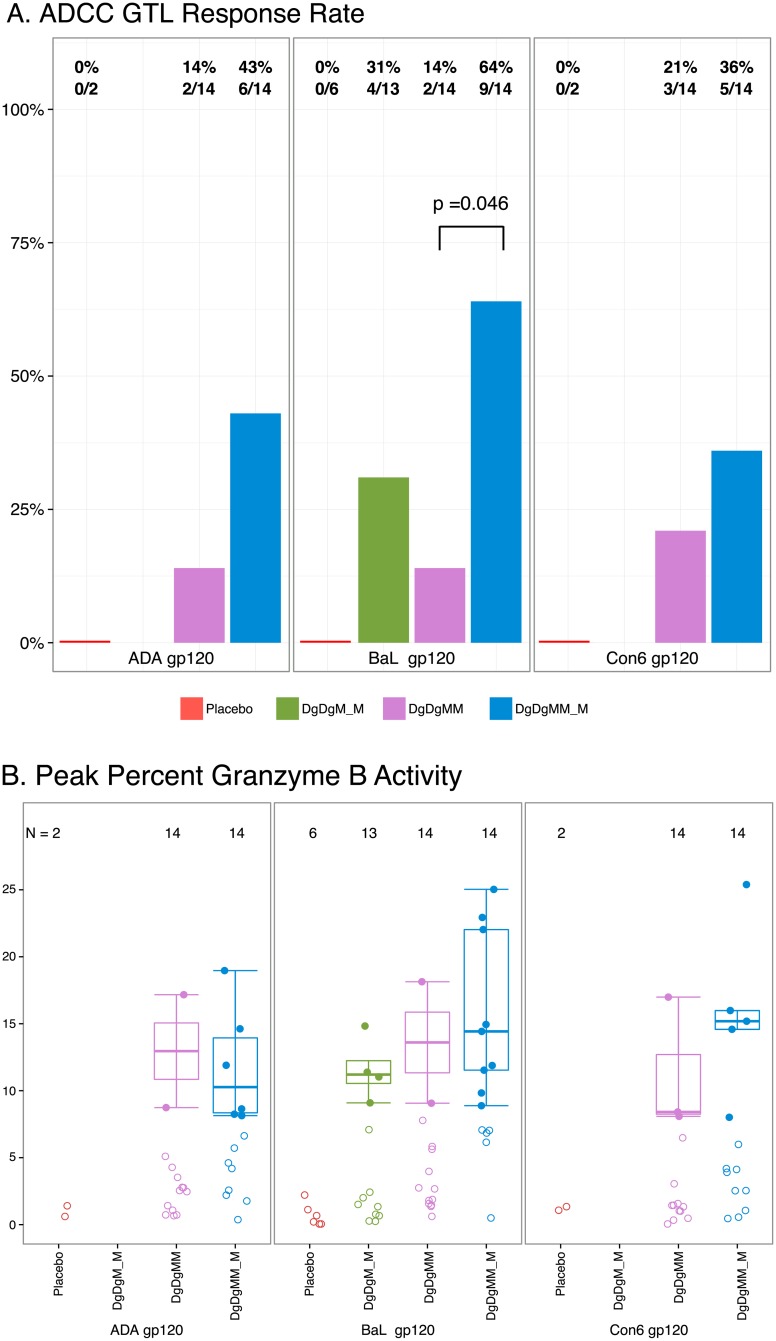
A. ADCC response rate (GTL). B. ADCC response magnitude. (A) Peak ADCC response rates. (B) Peak ADCC response magnitudes. Responses are to individual antigens at two weeks after the 2nd and 3rd MVA or placebo in the Placebo, DgDgM_M and DgDgMM_M groups. DgDgM_M refers to immune responses after the last MVA in the DgDgM_M group; DgDgMM and DgDgMM_M refer to immune responses after the 2nd and 3rd MVA in DgDgMM_M group, respectively. For the DgDgM_M arm, only peak percent Granzyme B activity for BaL gp120 was measured. Positive responses are shown as filled circles and negative responses are shown as open circles (B). Box-plots represent the distribution for the positive responders only. Response rates were compared using Fisher’s exact test (A); response magnitudes among responders across treatment arms were compared using Wilcoxon Rank Sum test (B).

At the peak time-point, the proportion of ADCC responders was significantly higher after the 3- vs. 2- MVA in the DgDgMM_M arm for BaL gp120 (64% vs. 14%, p = 0.046), but not for the two other gp120 antigens although with a similar trend (Fig 6A). No significant differences in ADCC response rates or response magnitudes (Fig 6A and 6B) were seen between the 2- vs. 4-month pause. At 6-months after the last MVA, only 23% and 13% of participants generated a positive ADCC response in the DgDgM_M and DgDgMM_M arms, respectively.

#### HIV-1 specific cellular immune responses

**Fig 7:**
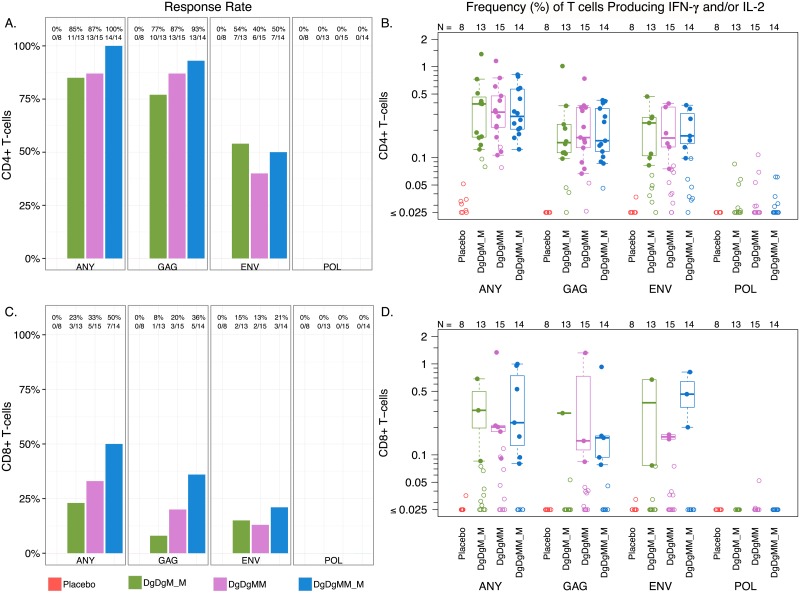
Cellular immune responses measured by intracellular cytokine staining (ICS). (A) CD4+ response rates. (B) CD4+ response magnitude. (C) CD8+ response rate. (D) CD8+ response magnitudes. Peak ICS response rates and response magnitude to Gag, Env and Pol in the CD4+ (Panels A & B) and CD8+ (Panels C & D) T-cell subsets at two weeks after the 2nd and 3rd MVA or placebo in the Placebo, DgDgM_M and DgDgMM_M groups. DgDgM_M refers to immune responses after the last MVA in the DgDgM_M group; DgDgMM and DgDgMM_M refer to immune responses after the 2nd and 3rd MVA in DgDgMM_M group, respectively. Shown are response rates (A, C) and response magnitudes (B, D) for the sum of individual antigen responses, reported as % of CD4+ or CD8+ T cells producing IFN-gamma and/or IL-2. Positive responses are shown as filled circles and negative responses are shown as open circles (B, D). Box-plots represent the distribution for the positive responders only. Response rates were compared using Fisher’s exact test (A, C); response magnitudes among responders across treatment arms were compared using Wilcoxon Rank Sum test (B, D).

Overall response rates from ICS assays for IL-2 and/or IFN-y were higher for CD4+ than CD8+ T cells (Fig 7A and 7C). CD4+ and CD8+ T cell responses were primarily to Gag, and to a lesser extent to Env, with minimal or no response to Pol. Response rates did not differ significantly after 2- vs. 3-doses of MVA, nor after a 2- vs. 4-month pause between the first and second MVA dose. CD4+ and CD8+ T cell magnitudes at peak time points were also not significantly different between 2- vs. 3-dose MVA or the 2- vs. 4-month pause arms (Fig 7B and 7D).

### Multivariate analysis of peak antibody-mediated and cellular immune responses

**Fig 8:**
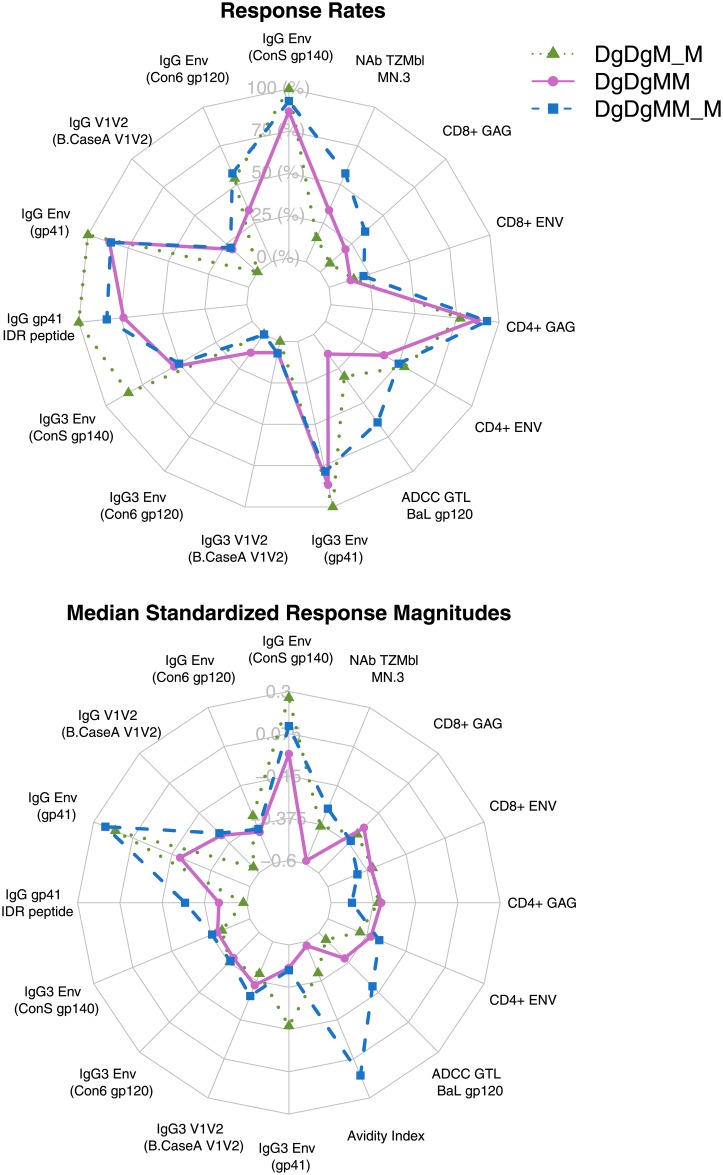
Radar plot of peak antibody-mediated and cellular immune responses by vaccine regimen. (A) Response rates of each assay readout. (B) Response magnitudes of each assay readout. DgDgM_M refers to immune responses after the last MVA in the DgDgM_M group; DgDgMM and DgDgMM_M refer to immune responses after the 2nd and 3rd MVA in DgDgMM_M group, respectively.

Collectively, the 3-dose MVA arm generated comparable or better antibody-mediated and cellular immune responses compared to the 2-dose MVA arm, whereas the overall immune response profile were generally comparable between the 2- vs. 4-month pause arms (Fig 8). In addition, immune responses to multiple antigens measured by the same assay tended to be more correlated among themselves, compared to correlations between antibody-mediated and cellular immune responses. No clear separation of the vaccine regimens was observed in a reduced 2-dimension immune response coordinate, possibly due to the fact that the first two principal components explained only 40% of the total variation in the data with a limited sample size (S5 Fig).

### End of study vaccine-induced Seropositivity/Reactivity (VISP/R) results

At the final study visit (6 months post-last MVA vaccination for the 1/10th dose arm and 12 months post- for each full-dose arm), each participant was assessed for the detection of HIV-1 antibodies by commercial assays, with confirmatory HIV nucleic acid testing performed for reactive samples. No participants were HIV-1 infected. The Vaccine-Induced Seropositivity/Reactivity (VISP/R) rates were 90%, 93% and 94% for the 1/10th dose arm, DgDgM_M, and DgDgMM_M arms, respectively.

## Discussion

These GEO-D03/MVA HIV62B regimens induced an HIV-1 humoral immune profile that included Env IgG1 and IgG3 binding responses, high antibody avidity, and low level ADCC and neutralizing antibody responses, without substantial IgA; the cellular immune response was biased toward CD4+ T cells. The RV144 trial [9] had a similar immune profile but targeted different epitopes and had a more short-lived immune response [14]. IgG3, an independent predictor of reduced HIV-1 infection and putatively of the level of vaccine efficacy in the RV144 trial [14], nearly disappeared at 6 months post-vaccination in RV144, while in HVTN 094, approximately one- half of the participants in each full-dose arm still had IgG3 responses by 6 months post-vaccination, and response rates and magnitudes dropped only slightly from 6- to 12-months post-vaccination. These data suggest that the GEO-D03/MVA HIV62B regimens produce a more durable response than was seen in RV144.

As noted above, the targeted epitopes were completely different between RV144 and HVTN 094. HVTN 094 vaccinees predominantly targeted gp41 while the protective responses in RV144 were directed at gp120. A preclinical trial using a SHIV prototype of the vaccine used here found that avidity to the IDR of gp41 correlated inversely with peak viremia when the animals were challenged with a single high dose of SHIV-162P3 [43]. Similarly, a passive-transfer study of the F240 monoclonal antibody directed at gp41 was associated with prevention of vaginal SHIV acquisition in 2 of 5 macaques and a delay in the appearance and reduction in the levels of viremia in 2 other animals [44]. Other passive transfer experiments with antibody to the IDR have shown reductions in peak viremia, but not prevention of infection [45]. Although antibodies to the gp41 IDR are not broadly neutralizing [44, 45] they can capture virions and mediate antibody-mediated cellular phagocytosis (ADCP), antibody functions associated with control of infection [44–46]. Antibodies to the gp41 IDR also have been associated with significantly reduced transmitted/founder virus genomes in a non-human primate high-dose SHIV challenge model, although did not protect against productive clinical infection [47]. However, a gp41 subunit virosome vaccine regimen that elicited antiviral gp41 specific antibodies did protect nonhuman primates against vaginal SHIV challenges [48]. Additionally, in a nonhuman primate SIV immunization model, gp41 antibodies concentrated at the mucosa correlated with protection [49, 50]. Thus, studies from multiple independent groups employing different strategies of passive infusion and vaccination studies in nonhuman primates have demonstrated the potential of gp41 antibodies to protect.

A recent paper hypothesized that the predominance of the gp41 humoral response in many HIV-1 vaccine trials originates from cross-reactive priming by intestinal microbial antigens [31]. B cell repertoire analysis of Env-reactive memory B cells from recipients of the DNA/recombinant Ad5 HIV-1 vaccine regimen found to be non-efficacious in the HVTN 505 trial isolated conformational gp41 mAbs that were cross-reactive with intestinal microbial antigens. These gp41-reactive antibodies were unable to neutralize HIV or mediate ADCC. Notably, the IDR was deleted in that vaccine immunogen, whereas the HVTN 094 vaccines elicited functional gp41 antibodies to the IDR. Moreover, the HVTN 094 vaccine products did not elicit circulating gp41 IgG cross-reactive with intestinal microflora. Thus, the HVTN 094 vaccine regimen explores a novel immunologic space by eliciting functional antibodies with a different but promising target antigen than was seen in RV144. Differences in the target antigen may also explain improved durability of antibodies in HVTN 094 compared with RV 144. The extent to which humoral responses to gp41, either conformational or linear, contribute to protection will be important to study in future HIV-1 vaccine efficacy studies.

Virtually all of the IgG produced by participants in the HVTN 094 trial were of the IgG1 and IgG3 subtypes, with almost no IgG2 or IgG4 responses. In RV144, HIV-1 Env IgG3 correlated with decreased HIV-1 risk [14] and has been shown to have favorable functional features, such as the ability to fix complement, neutralize HIV-1 [51], and mediate ADCC and ADCP [46, 52]. As in HVTN 094, IgG1 and IgG3 responses predominated in the RV144 trial; in the VAX003 trial showing no efficacy, IgG1, IgG2 and IgG4 were predominant [14, 53]. Further studies are needed to examine the full functional attributes of the antibody specificities elicited in this study.

On the other hand, IgA blocked ADCC-mediating antibodies to envelope in the RV144 trial, and ADCC was only associated decreased risk of HIV-1 infection in the absence of IgA [15]. Because of this competition for envelope binding between IgA and IgG, having a low IgA to IgG ratio may be important in protection, particularly if they block a functional IgG-mediated response. In HVTN 094, few participants developed envelope-specific IgA and those who did only generated low response titers.

The third MVA dose appeared to improve both the quality and longevity of the antibody response. ADCC responses were substantially improved after the third MVA dose, and the magnitude of IgG to gp140 remained stable from 6 to 12 months post-vaccination, in contrast to waning values in the two-dose MVA arm. Response rates and titers of neutralizing antibody, also increased for MN3, the virus with the most substantial neutralizing antibody responses, with the 3^rd^ MVA dose. A previous study demonstrated improved antibody responses to an influenza DNA/monovalent inactivated vaccine prime-boost regimen if the pause between immunizations was at least 12 weeks [18]. In this study, we found trends but no significant difference if the second MVA was given with a 4- versus 2-month window based on the limited sample sizes. Because this comparison was made before a third MVA boost in one study arm, we cannot assess any effects of the pause on the longevity of the immune response.

A non-human primate study using an earlier HIV prototype vaccine demonstrated greater protection when the GM-CSF co-expressing DNA was used as the prime [19]. This increased protection correlated with an increased avidity of the Env-specific Ab response in the GM-CSF adjuvanted group. However, a subsequent non-human primate trial using a DNA prime updated to be an SIV analogue of JS7 DNA co-expressing GM-CSF generated neither improved antibody avidity nor improved protection against SHIV challenge. Although the GEO-D03 vaccine appeared safe in the HVTN 094 study, the co-expressed GM- CSF did not enhance the avidity of antibody responses (K. Seaton, personal communication), nor binding antibody or T cell responses [16, 54]. Future studies will include the DNA product without inclusion of co-expressed GM-CSF.

This study is limited by its small size, calling for confirmation of the findings of this study using a DDMM_M regimen moving forward. In addition, in RV144, certain class I and class II HLA genotypes have been found to be associated with immune responses and protection [55, 56], as was a single nucleotide polymorphism in an Fc-γ receptor gene [57]. Only ADCC directed at gp120 was measured in the HVTN 094 study; gp41-mediated responses may have been missed. These may be areas to explore in future trials of these products. Finally, results of this trial in four US cities may not reflect the immune responses from participants in other clade B regions, and the cross-clade applicability of this regimen is not known.

The Global HIV Vaccine Enterprise has called for a rational approach to moving forward vaccine candidates, particularly those that build upon previous evidence of protection while exploring a novel immune space [58]. The DNA prime, MVA boost HIV-1 vaccine combination has been protective in non- human primate models, and in this study, generated durable IgG and IgG3 to envelope proteins, high antibody avidity, and ADCC, with low to absent IgA responses. Future studies will explore whether adding a gp120 protein boost will improve the breadth, magnitude, or durability of the immune response.. With the HIV-1 epidemic ongoing in the US [59], South America [60], and Europe [61, 62], development of a safe HIV-1 vaccine effective in clade B epidemics remains one of the highest priorities in HIV prevention science.

## Supporting information

S1 Protocol Protocol HVTN 094 version 2. (PDF)

S1 CONSORT checklist Checklist to ensure compliance with CONSORT guidelines. (PDF)

S1 Table Demographics and vaccination frequencies. (DOCX)

S1 Fig Local and systemic reactogenicity. (A) Local reactogenicity. (B) Systemic reactogenicity. Frequency of maximum severity of reactogenicity symptoms by vaccination for placebo recipients (combined for arms 1–3) and vaccine recipients in each treatment arm. DgDgM_M refers to immune responses after the last MVA in the DgDgM_M group; DgDgMM and DgDgMM_M refer to immune responses after the 2nd and 3rd MVA in DgDgMM_M group, respectively.
(EPS)

S2 Fig Peak IgG binding antibody response rates and magnitude. (A) Peak IgG binding antibody response rates. (B) Peak IgG binding antibody response magnitudes. Binding antibody response to individual antigens at two weeks after the 2nd and 3rd MVA or placebo in the Placebo, DgDgM_M and DgDgMM_M groups. DgDgM_M refers to immune responses after the last MVA in the DgDgM_M group; DgDgMM and DgDgMM_M refer to immune responses after the 2nd and 3rd MVA in DgDgMM_M group, respectively. Shown are total IgG binding antibody to Clade B Lab Adapted Env, Clade C Transmitted/Founder Env, and Non-Env antigen. Positive responses are shown as filled circles and negative responses are shown as open circles. Box-plots represent the distribution for the positive responders only.
(EPS)

S3 Fig Peak IgA binding antibody response rates and magnitude. (A) IgA response rates. (B) IgA response magnitude. Binding antibody responses to individual antigens at two weeks after the 2nd and 3rd MVA or placebo in the Placebo, DgDgM_M and DgDgMM_M groups. DgDgM_M refers to immune responses after the last MVA in the DgDgM_M group; DgDgMM and DgDgMM_M refer to immune responses after the 2nd and 3rd MVA in DgDgMM_M group, respectively. Shown are total IgA binding antibody responses to HIV-1 Env gp140 (ConS gp140), HIV-1 Env gp120 (Con 6 gp120 B), the V1V2 loop, and gp41. Positive responses are shown as filled circles and negative responses are shown as open circles. Box-plots represent the distribution for the positive responders only.
(EPS)

S4 Fig Peak neutralizing antibody response rates. Peak response rates of neutralization antibody responses at two weeks after the 2nd and 3rd MVA or placebo in the Placebo, DgDgM_M and DgDgMM_M groups. DgDgM_M refers to immune responses after the last MVA in the DgDgM_M group; DgDgMM and DgDgMM_M refer to immune responses after the 2nd and 3rd MVA in DgDgMM_M group, respectively. Neutralization IC50 antibody titers were measured in TZM-bl cells against a panel of heterologous Env-pseudotyped viruses (Clade B: BaL.26, MN.3, SF162.LS; Clade C: MW965.26).
(EPS)

S5 Fig Principal component (PC) biplot of peak antibody-mediated and cellular immune responses by vaccine regimen. DgDgM_M refers to immune responses after the last MVA in the DgDgM_M group; DgDgMM and DgDgMM_M refer to immune responses after the 2nd and 3rd MVA in DgDgMM_M group, respectively. The x- and y-axes are the values from the 1st and 2nd PC, respectively, that explain the most variation in the data. Points on the plot represent the values of the PCs of each observation. Points that are close together correspond to observations that have similar values in the two PCs. The top axis is the 1st PC loadings and the right axis is the 2nd PC loadings, where loadings are the weights by which each original assay readout is multiplied to get the value of the corresponding PCs. An arrow (vector) is drawn for each assay readout from the origin to the point defined by its first two PC loadings. Vectors that point in the same direction correspond to readouts that have similar response profiles on the basis of the first two PCs. The observations whose points project furthest in (opposite of) the direction in which the vector points are the observations that have the most (least) weight of the corresponding readout. The angle between two arrows conveys information about the correlation of the assay readouts, with a zero degree angle denoting perfect correlation and a 90 degree angle denoting no correlation.
(EPS)
