# Supplementary material for: Immunogenicity of a novel Clade B HIV-1 vaccine combination: Results of phase 1 randomized placebo controlled trial of an HIV-1 GM-CSF-expressing DNA prime with a modified vaccinia Ankara vaccine boost in healthy HIV-1 uninfected adults
Source: PLoS One. 2017 Jul 20;12(7):e0179597. doi: 10.1371/journal.pone.0179597 (PMC5519050; doi:10.1371/journal.pone.0179597)
Supplement: S1 Protocol — (PDF) [file pone.0179597.s001.pdf]

HIV VACCINE
TRIALS NETWORK

PROTOCOL

HVTN 094

A phase 1 placebo controlled clinical trial to evaluate the safety and immunogenicity of a prime-boost vaccine regimen of GEO-D03 DNA and MVA/HIV62B vaccines in healthy, HIV-1-uninfected vaccinia naïve adult participants

DAIDS DOCUMENT ID 11827

BB IND 14980

CLINICAL TRIAL SPONSORED BY

Division of AIDS (DAIDS)
National Institute of Allergy and Infectious Diseases (NIAID)
National Institutes of Health (NIH)
Department of Health and Human Services (DHHS)
Bethesda, Maryland, USA

STUDY PRODUCTS PROVIDED BY

GeoVax, Inc.
Smyrna, Georgia, USA

January 7, 2013

HVTN 094, Version 2.0

In Version 2.0 of HVTN 094, the protocol and specifically Group 2, is modified to evaluate two additional aims. The first is to determine the extent to which 3 versus 2 doses of MVA will affect the antibody responses if there is a 4 month interval before the last MVA dose (new secondary objective 1 in Section 5.2). The second aim is to determine the extent to which a lengthening of the interval between the second and third MVA will affect the antibody responses (new secondary objective 2 in Section 5.2). To accomplish these aims, the original Group 2 will be split into two new and different groups. The first new group is termed Group 2 and participants in this group will receive MVA injections at months 4, 6 and 10. The second new group is termed Group 3 and participants in this group will receive MVA injections at months 4 and 8. Some participants in the revised Group 2 and in Group 3 may receive the Month 4 MVA injection under Version 1.0. Both the revised Group 2 and Group 3 will have received the GEO-D03 DNA injections previously at months 0 and 2 per Version 1.0 of the protocol. The new schema is illustrated in Section 3, Overview. Group 1 is unaffected by this modification.

The rationale for these new study aims comes from several sources. Both previously reported antibody data as well as newly generated avidity data from HVTN 065 demonstrate enhanced antibody responses in both the DMM and MMM groups which had a 4 month interval between the last two vaccinations, compared to the DDMM group which had only a 2 month interval between all vaccinations (see Section 4.9.1). Recent data from influenza vaccine clinical trials has substantiated these observations, demonstrating kinetics of antibody responses which begin to show improvements at 3 months, with functional activity (avidity) beginning to improve at 4 months and epitope breadth at 6 months (Barney Graham, VRC personal communication) (see Section 4.5). Furthermore, data has emerged from the ongoing NHP studies indicating improved anti-Env antibody titers and enhanced avidity in late boosted animals (see Section 4.8). Also, it has been known for some time from earlier studies of envelope subunit HIV vaccines [1,2] and pneumococcal vaccines [3] that a rest period is needed to maximize the humoral immune response to these vaccines Therefore, the composite of these data warrant the modification of HVTN 094 to examine these vaccine products administered at a longer 4 month interval between the last 2 MVA doses.

In order to accommodate the splitting of the original Group 2 from Version 1.0 into the revised Group 2 and Group 3 of Version 2.0, the first 18 participants (who were originally randomized into blocks of 5:1 active to placebo) enrolled into the original Group 2 will follow the new Version 2.0 Group 2 schedule. The last 18 participants enrolled into the original Group 2 will follow the new Version 2.0 Group 3 schedule. This will ensure the integrity of randomization and blinding within each group.

Contents

1 Ethical considerations ... 6

2 IRB/EC review considerations ... 8
2.1 Minimized risks to participants ... 8
2.2 Reasonable risk/benefit balance ... 8
2.3 Equitable subject selection ... 8
2.4 Appropriate informed consent ... 9
2.5 Adequate safety monitoring ... 9
2.6 Protect privacy/confidentiality ... 9

3 Overview ... 10
3.1 Protocol Team ... 13

4 Background ... 14
4.1 Rationale for trial concept ... 14
4.2 GEO-D03 DNA vaccine ... 15
4.3 MVA62B vaccine ... 16
4.4 Additional GeoVax vaccine product information ... 17
4.5 Trial design rationale ... 19
4.6 Plans for future product development and testing ... 20
4.7 Preclinical safety studies ... 21
4.8 Preclinical immunogenicity studies ... 27
4.9 Clinical studies ... 37
4.10 Potential risks of study products and administration ... 44

5 Objectives and endpoints ... 46
5.1 Primary objectives and endpoints ... 46
5.2 Secondary objectives and endpoints ... 46
5.3 Exploratory objectives ... 48

6 Statistical considerations ... 50
6.1 Accrual and sample size calculations ... 50
6.2 Randomization ... 58
6.3 Blinding ... 58
6.4 Statistical analysis ... 59

7 Selection and withdrawal of participants ... 63
7.1 Inclusion criteria ... 63
7.2 Exclusion criteria ... 65
7.3 Participant departure from vaccination schedule or withdrawal ... 68

8 Study product preparation and administration ... 71
8.1 Vaccine regimen ... 71
8.2 Study product formulation ... 73
8.3 Preparation of study products ... 74
8.4 Administration ... 76
8.5 Acquisition of study products ... 77
8.6 Pharmacy records ... 77
8.7 Final disposition of study products ... 77

9 Clinical procedures ... 78
9.1 Informed consent ... 78
9.2 Pre-enrollment procedures ... 80
9.3 Enrollment and vaccination visits ... 81

9.4 Follow-up visits ... 82
9.5 Annual health contacts ... 85
9.6 HIV counseling and testing ... 86
9.7 Contraception status ... 87
9.8 Urinalysis ... 87
9.9 Assessments of reactogenicity ... 88
9.10 Visit windows and missed visits ... 89
9.11 Early termination visit ... 89
9.12 Pregnancy ... 89

10 Laboratory ... 90
10.1 HVTN CRS laboratory procedures ... 90
10.2 Total blood volume ... 90
10.3 Primary immunogenicity timepoint ... 90
10.4 Endpoint assays: cellular ... 90
10.5 Endpoint assays: humoral ... 91
10.6 Innate immunity assays ... 92
10.7 Genotyping ... 92
10.8 Exploratory studies ... 92
10.9 Ancillary studies ... 93
10.10 Other use of stored specimens ... 93
10.11 Biohazard containment ... 94

11 Safety monitoring and safety review ... 95
11.1 Safety monitoring and oversight ... 95
11.2 Safety reporting ... 96
11.3 Safety reviews ... 98
11.4 Safety pause and prompt PSRT AE review ... 99
11.5 Review of cumulative safety data ... 100
11.6 Study termination ... 100

12 Protocol conduct ... 101
12.1 Social impacts ... 101
12.2 Compliance with NIH guidelines for research involving products containing recombinant DNA ... 102
12.3 Emergency communication with study participants ... 102

13 Version history ... 103

14 Document references (other than literature citations) ... 104

15 Acronyms and abbreviations ... 106

16 Literature cited ... 109

Appendix A Sample informed consent form ... 117

Appendix B Approved birth control methods (for sample informed consent form) ... 134

Appendix C HVTN VISP registry consent ... 135

Appendix D Table of procedures for Group 1 (for sample informed consent form) ... 138

Appendix E Table of procedures for Group 2 (for sample informed consent form) ... 139

Appendix F Table of procedures for Group 3 (for sample informed consent form) ... 140

Appendix G Laboratory procedures for Group 1 ... 141

Appendix H Laboratory procedures for Group 2 ... 142

Appendix I Laboratory procedures for Group 3 ... 143

Appendix J Procedures at HVTN CRS for Group 1 144
Appendix K Procedures at HVTN CRS for Group 2 145
Appendix L Procedures at HVTN CRS for Group 3 146
Appendix M Procedures at CRS for annual health contacts 147
Appendix N Case definition of myo/pericarditis for use in adverse events monitoring 148
Appendix O AESI in HVTN 094 149

1 Ethical considerations

Multiple candidate HIV vaccines will need to be studied simultaneously in different populations around the world before a successful HIV preventive vaccine is found. It is critical that universally accepted ethical guidelines are followed at all sites involved in the conduct of these clinical trials. The HIV Vaccine Trials Network (HVTN) has addressed ethical concerns in the following ways:

- HVTN trials are designed and conducted to enhance the knowledge base necessary to find a preventive vaccine, using methods that are scientifically rigorous and valid, and in accordance with Good Clinical Practice (GCP) guidelines.

- HVTN scientists and operational staff incorporate the philosophies underlying major codes [4-6], declarations, and other guidance documents relevant to human subjects research into the design and conduct of HIV vaccine clinical trials.

- HVTN scientists and operational staff are committed to substantive community input—into the planning, conduct, and follow-up of its research—to help ensure that locally appropriate cultural and linguistic needs of study populations are met. Community Advisory Boards (CAB) are required by DAIDS and supported at all HVTN research sites to ensure community input.

- HVTN clinical trial staff counsel study participants routinely on how to reduce HIV risk. Participants who become HIV-infected during the trial are provided counseling on notifying their partners and about HIV infection according to local guidelines. Staff members will also counsel them about reducing their risk of transmitting HIV to others.

- Participants who become HIV-infected during the trial are referred to medical practitioners to manage their HIV infection and to identify potential clinical trials they may want to join.

- The HVTN provides training so that all participating sites similarly ensure fair participant selection, protect the privacy of research participants, and obtain meaningful informed consent. During the study, participants will have their wellbeing monitored, and to the fullest extent possible, their privacy protected. Participants may withdraw from the study at any time.

- Prior to implementation, HVTN trials are rigorously reviewed by scientists who are not involved in the conduct of the trials under consideration.

- HVTN trials are reviewed by local and national regulatory bodies and are conducted in compliance with all applicable national and local regulations.

- The HVTN designs its research to minimize risk and maximize benefit to both study participants and their local communities. For example, HVTN protocols provide enhancement of participants' knowledge of HIV and HIV prevention, as well as counseling, guidance, and assistance with any social impacts that may result from research participation. HVTN protocols also include careful medical review of each research participant's health conditions and reactions to study products while in the study.

- HVTN research aims to benefit local communities by directly addressing the health and HIV prevention needs of those communities and by strengthening the capacity of the communities through training, support, shared knowledge, and equipment. Researchers involved in HVTN trials are able to conduct other critical research in their local research settings.

- The HVTN recognizes the importance of institutional review and values the role of in country Institutional Review Boards (IRBs) and Ethics Committees (ECs) as custodians responsible for ensuring the ethical conduct of research in each local setting.

2 IRB/EC review considerations

US Food and Drug Administration (FDA) and other US federal regulations require IRBs or ECs to ensure that certain requirements are satisfied on initial and continuing review of research (Title 45, Code of Federal Regulations (CFR), Part 46.111(a) 1-7; 21 CFR 56.111(a) 1-7). The following section highlights how this protocol addresses each of these research requirements. Each HVTN Investigator welcomes IRB/EC questions or concerns regarding these research requirements.

2.1 Minimized risks to participants

45 CFR 46.111 (a) 1 and 21 CFR 56.111 (a) 1: Risks to subjects are minimized.

This protocol minimizes risks to participants by (a) correctly and promptly informing participants about risks so that they can join in partnership with the researcher in recognizing and reporting harms; (b) respecting local/national blood draw limits; (c) performing direct observation of participants postvaccination and collecting information regarding side effects for several days postvaccination; (d) having staff properly trained in administering study procedures that may cause physical harm or psychological distress, such as blood draws, vaccinations, HIV testing and counseling and HIV risk reduction counseling; (e) providing HIV risk reduction counseling and checking on contraception use (for women); and (f) providing safety monitoring.

2.2 Reasonable risk/benefit balance

45 CFR 46.111 (a) 2 and 21 CFR 56 (a) 2: Risks to subjects are reasonable in relation to anticipated benefits, if any, to subjects, and the importance of the knowledge that may reasonably be expected to result.

In all public health research, the risk-benefit ratio may be difficult to assess because the benefits to a healthy participant are not as apparent as they would be in treatment protocols, where a study participant may be ill and may have exhausted all conventional treatment options. However, this protocol is designed to minimize the risks to participants while maximizing the potential value of the knowledge it is designed to generate.

2.3 Equitable subject selection

45 CFR 46.111 (a) 3 and 21 CFR 56.111 (a) 3: Subject selection is equitable

This protocol has specific inclusion and exclusion criteria for investigators to follow in admitting participants into the protocol. Participants are selected because of these criteria and not because of positions of vulnerability or privilege. Investigators are required to maintain screening and enrollment logs to document volunteers who screened into and out of the protocol and for what reasons.

2.4 Appropriate informed consent

45 CFR 46.111 (a) 4 and 5 and 21 CFR 56.111 (a) 4 and 5: Informed consent is sought from each prospective subject or the subject's legally authorized representative as required by 45 CFR 46.416; informed consent is appropriately documented as required by 45 CFR 46.417

The protocol specifies that informed consent must be obtained before any study procedures are initiated and assessed throughout the trial (see Section 9.1). Each site is provided training in informed consent by the HVTN as part of its entering the HVTN. The HVTN requires a signed consent document for documentation, in addition to chart notes or a consent checklist.

2.5 Adequate safety monitoring

45 CFR 46.111 (a) 6 and 21 CFR 56.111 (a) 6: There is adequate provision for monitoring the data collected to ensure the safety of subjects.

This protocol has extensive safety monitoring in place (see Section 11). Safety is monitored daily by clinical affairs staff and routinely by the HVTN 094 Protocol Safety Review Team (PSRT). Site staff have 24-hour cell phone access to clinical affairs staff. In addition, the HVTN Safety Monitoring Board (SMB) or a Data and Safety Monitoring Board (DSMB) periodically reviews study data.

2.6 Protect privacy/confidentiality

45 CFR 46.111 (a) 7 and 21 CFR 56.111 (a) 7: There are adequate provisions to protect the privacy of subjects and maintain the confidentiality of data.

Privacy refers to an individual's right to be free from unauthorized or unreasonable intrusion into his/her private life and the right to control access to individually identifiable information about him/her. The term "privacy" concerns research participants or potential research participants as individuals whereas the term "confidentiality" is used to refer to the treatment of information about those individuals. This protocol respects the privacy of participants by informing them about who will have access to their personal information and study data (see Appendix A). The privacy of participants is protected by assigning unique identifiers in place of the participant's name on study data and specimens. In the United States, research participants in HVTN protocols are protected by a Certificate of Confidentiality from the US NIH, which can prevent disclosure of study participation even when that information is requested by subpoena. Participants are told of the use and limits of the certificate in the study consent form. In addition, each staff member at each study site in this protocol signs a Confidentiality Agreement with the HVTN and each study site participating in the protocol is required to have a standard operating procedure on how the staff members will protect the confidentiality of study participants.

3 Overview

Title

A phase 1 placebo controlled clinical trial to evaluate the safety and immunogenicity of a prime-boost vaccine regimen of GEO-D03 DNA and MVA/HIV62B vaccines in healthy, HIV-1-uninfected vaccinia naïve adult participants

Primary objective

- To assess the safety and tolerability of a heterologous prime-boost regimen consisting of two injections of GEO-D03 DNA vaccine followed by two or three injections of modified vaccinia Ankara (MVA)/HIV62B (MVA62B) with dose escalation of the GEO-D03 DNA vaccine from 0.3 mg to 3 mg

Study products and routes of administration

- **GEO-D03 DNA vaccine**: a 9.9 kb plasmid DNA expressing HIV-1 proteins Gag, PR, RT, Env, Tat, Rev, and Vpu, and human granulocyte-macrophage colony-stimulating factor (GM-CSF). The DNA vaccine has been vialed at a concentration of 3 mg/mL. Both the 0.3 mg and 3 mg injections will be administered as a 1 mL intramuscular (IM) injection into the deltoid.

- **Formulation Buffer for dilution of GEO-D03 DNA**: Phosphate-buffered saline (PBS), EDTA (ethylenediamine tetraacetic acid), and ethanol.

- **MVA/HIV62B (MVA62B) vaccine***: a highly attenuated vaccinia virus expressing HIV-1 *gag*, *pol*, and *env* genes from the same HIV-1 sequences present in the GEO-D03 DNA vaccine. The MVA62B vaccine has been vialed at $1x10^8$ 50% tissue culture infective dose ($TCID_{50}$)/mL. A $1x10^8$ $TCID_{50}$ dose will be administered as a 1mL IM injection into the deltoid.

  **Note:* MVA/HIV62B (MVA62B) was formerly designated as MVA/HIV62 (MVA62); the B was added to indicate Clade B. The product lot being used in this study was labeled as HIV/MVA62.

- **Placebo for GEO-D03 DNA**: Sodium Chloride for Injection USP, 0.9%. The GEO-D03 DNA placebo will be administered as a 1mL IM injection into the deltoid.

- **Placebo for MVA62B**: Sodium Chloride for Injection USP, 0.9%. The MVA62B placebo will be administered as a 1mL IM injection into the deltoid

Table 3-1 Schema for Version 2.0

Study arm	N	GEO-D03 DNA	MVA62B	Month 0 (Day 0)	Month 2 (Day 56)	Month 4 (Day 112)	Month 6 (Day 168)	Month 8 (Day 224)	Month 10 (Day 303)
Group 1	10	0.3mg	$1x10^8$ $TCID_{50}$	GEO-D03 DNA	GEO-D03 DNA	MVA62B	MVA62B	MVA62B	------
	2	0	0	placebo	placebo	placebo	placebo	placebo	------
Group 2	15	3mg	$1x10^8$ $TCID_{50}$	GEO-D03 DNA	GEO-D03 DNA	MVA62B	MVA62B	------	MVA62B
	3	0	0	Placebo	Placebo	placebo	placebo	------	placebo
Group 3	15	3mg	$1x10^8$ $TCID_{50}$	GEO-D03 DNA	GEO-D03 DNA	MVA62B	------	MVA62B	------
	3	0	0	placebo	placebo	placebo	------	placebo	------
Total	48 (40 vaccine / 8 placebo)								

Notes

All enrollments occurred during Version 1.0. Group 1 participants are unaffected by the change described in Version 2.0

Enrollment will begin with Group 1 participants. The first six subjects across all sites will be enrolled at a rate of no more than one per day. Prior to enrolling any additional participants into Group 1, the HVTN 094 PSRT will review and approve the reactogenicity and safety data of the first 6 participants through day 14 after the initial GEO-D03 DNA injection. If the reactogenicity and safety data are deemed acceptable, Group 1 will continue to enroll until completion. Prior to beginning enrollment into Group 2, the HVTN 094 PSRT will review and approve the reactogenicity and safety data of all Group 1 participants through day 14 after the initial GEO-D03 DNA injection. If the reactogenicity and safety data are deemed acceptable, Group 2 may begin enrollment. The first six Group 2 subjects across all sites will be enrolled at a rate of no more than one per day. Prior to enrolling any additional participants into Group 2, the HVTN 094 PSRT will review and approve the reactogenicity and safety data of the first 6 participants through day 14 after the initial GEO-D03 DNA injection. If the reactogenicity and safety data are deemed acceptable, Group 2 will continue to enroll until completion. To ensure each group has the same number of participants in the treatment and placebo groups, randomization is being done in blocks of 6 participants to which 5 treatment and one placebo slots have been assigned.

For Version 2.0:

In order to accommodate the splitting of the original Group 2 from Version 1.0 into the new Groups 2 and 3 of Version 2.0, the first 18 participants (who were originally randomized into blocks of 5:1 active to placebo) enrolled into the original Group 2 will follow the new Version 2.0 Group 2 schedule. The last 18 participants enrolled into the original Group 2 will follow the new Version 2.0 Group 3 schedule. This will ensure the integrity of randomization and blinding within each group.

Participants

48 healthy, HIV-1–uninfected volunteers aged 18 to 50 years; 40 vaccinees, 8 placebo recipients

Design

Multicenter, randomized, placebo-controlled, double-blind trial

Duration per participant

Group 2: 16 months of scheduled clinic visits (main study), followed by annual participant health contacts to a total of 3 years following initial study injection.

Groups 1 and 3: 14 months of scheduled clinic visits (main study) followed by annual participant health contacts to a total of 3 years following initial study injection.

Estimated total study duration

45 months (includes enrollment, planned safety holds, follow-up, and annual health contacts)

Investigational New Drug (IND) sponsor

DAIDS, NIAID, NIH, DHHS (Bethesda, Maryland, USA)

Study product providers

- GEO-D03 DNA vaccine: GeoVax, Inc. (Smyrna, Georgia, USA)
- Formulation Buffer for dilution of GEO-D03 DNA: GeoVax, Inc. (Smyrna, Georgia, USA)
- MVA62B vaccine: GeoVax, Inc. (Smyrna, Georgia, USA))

Core operations

HVTN Vaccine Leadership Group/Core Operations Center, Fred Hutchinson Cancer Research Center (FHCRC) (Seattle, Washington, USA)

Statistical and data management center (SDMC)

Statistical Center for HIV/AIDS Research and Prevention (SCHARP), FHCRC (Seattle, Washington, USA)

HIV diagnostic laboratory

University of Washington Virology Specialty Laboratory (UW-VSL) (Seattle, Washington, USA)

Endpoint assay laboratories

- Duke University Medical Center (Durham, North Carolina, USA)
- FHCRC/University of Washington (Seattle, Washington, USA)
- Rare Lung Disease Network Central Laboratory, Cincinnati Children's Hospital Research Foundation (Cincinnati, OH, USA)

Study sites

HVTN Clinical Research Sites (HVTN CRSs) to be specified in the Site Announcement Memo

Safety monitoring

HVTN 094 PSRT; HVTN Safety Monitoring Board (SMB)

3.1 Protocol Team

Protocol leadership

Chair	Susan Buchbinder<br>San Francisco Department of Public Health<br>415-554-9070<br>susan.buchbinder@sfdph.org	*Statistician*	Peter Gilbert<br>SCHARP, FHCRC<br>206-667-7299<br>pgilbert@scharp.org
Cochair	Christine (Mhorag) Hay<br>University of Rochester<br>585-275-6962<br>christine_hay@urmc.rochester.edu	*Medical officer*	G. Laissa Ouedraogo<br>DAIDS, NIAID<br>301-451-2779<br>ouedraogol@mail.nih.gov
Protocol Team leader	Nicole Grunenberg<br>HVTN Core, FHCRC<br>206-667-2043<br>ngrunenb@fhcrc.org		

Other contributors to the original protocol

Core medical monitor	Nicole Grunenberg<br>HVTN Core, FHCRC	*Statistical Research Associate*	Alicia Sato<br>SCHARP, FHCRC
Vaccine developer representative	Harriet Robinson<br>GeoVax, Inc.	*Clinical affairs*	Pat Farrell<br>SCHARP, FHCRC
Laboratory Program representative	Jin Bae<br>HVTN Laboratory Program, FHCRC	*Clinical trials manager*	Niles Eaton<br>HVTN Core, FHCRC
Regulatory affairs	Liz Briesemeister<br>HVTN Core, FHCRC	*Project manager*	Corey Kelly<br>SCHARP, FHCRC
Clinic coordinator	Theresa Wagner<br>San Francisco Department of Public Health	*Senior project manager*	Drienna Holman<br>SCHARP, FHCRC
Community Advisory Board (CAB) members	Alejandra Diaz<br>New York Blood Center / Columbia University CAB	*Protocol development coordinator*	Julie Foster<br>HVTN Core, FHCRC
	Victoria Chinnell, UW / FHCRC CAB	*Community relations and education unit representative*	Gail Broder<br>HVTN Core, FHCRC
HVTN pharmacist	Jan Johannessen<br>HVTN Core, FHCRC	*Community educator/recruiter*	Teress Votto<br>Brigham and Women's Hospital
DAIDS protocol pharmacist	Scharla Estep<br>DAIDS, NIAID<br>301-435-3746	*Technical editor*	Adi Ferrara<br>HVTN Core, FHCRC

4 Background

4.1 Rationale for trial concept

This first-in-human trial using a new GM-CSF adjuvanted DNA priming vaccine administered twice then boosted with 3 injections of MVA vaccine will be studied in a dose escalating fashion to determine safety, tolerability and immunogenicity compared to placebo.

GeoVax is developing recombinant MVA vaccines to be used with or without a DNA prime for the control of HIV-1 infections. Both the DNA and MVA components of the GeoVax vaccine express non-infectious immature virus-like particles (VLP) displaying trimeric membrane-bound forms of the HIV-1 envelope glycoprotein. Testing in humans has been conducted through the US National Institutes of Health (NIH)-sponsored HIV Vaccine Trials Network (HVTN) which has completed a phase 1 study (HVTN 065) [7] and is currently conducting a phase 2a study (HVTN 205). The HVTN trials tested the pGA2/JS7 (JS7) DNA vaccine and the MVA/HIV62B (MVA62B) vaccine in DNA prime-MVA boost regimens (JS7/MVA62B) and in MVA62B priming and boosting regimens (see Sections 4.9.1 and 4.9.2 for information on clinical trials). HVTN 065 was a dose and regimen comparison trial with initially both vaccines administered at one-tenth doses and subsequent regimens with various combinations of DNA (D) and MVA (M) primes with MVA boosts. Two regimens were chosen for further evaluation in HVTN 205 – the full dose DDMM and the full dose MMM regimens. The DNA prime-MVA boost vaccine to be tested in this trial, designated GEO-D03/MVA62B, differs from the previously tested JS7/MVA62B in adding co-expressed GM-CSF to the DNA priming vector.

The rationale for co-expressing GM-CSF with the JS7 plasmid in the GEO-D03 DNA prime is to modify the response to achieve better prevention of mucosal infection. This is believed achievable based on immune stimulatory functions of GM-CSF for innate immunity. GM-CSF favors avidity maturation of Env-specific IgG by stimulating the expansion and differentiation of myeloid dendritic cells [8]. Primate myeloid dendritic cells produce high levels of IL-6, a cytokine that promotes the formation of germinal centers and the growth and differentiation of B cells in germinal centers [9,10]. High avidity Env-specific antibody (Ab) responses are desirable for a preventative vaccine because they correlate with enhanced neutralizing activity, antibody dependent cellular cytotoxicity (ADCC), and antibody dependent cell-mediated virus inhibition(ACDVI) [11,12]. It has also been shown in a GM-CSF co-expressing DNA/MVA simian immunodeficiency virus (SIV) vaccine regimen model that the avidity of vaccine-elicited Ab for the Env of the challenge virus directly correlates with prevention of a repeat mucosal challenge in rhesus macaques ($r=0.9$, $p<0.0001$) [13]. GM-CSF favors mucosal responses by stimulating retinoic acid production in dendritic cells [14]. Retinoic acid enhances mucosal homing of elicited T and B cells [15,16] which is important for targeting immune responses at the site of viral entry in mucosal surfaces.

In the absence of additional signals, GM-CSF elicits Th2 cells that display CCR4 and CCR3 (rather than Th1 cells which display CCR5) [17-20]. This is desirable for an HIV vaccine since anti-viral CCR5 CD4 T cells are preferential targets for infection [21]. Additionally, GM-CSF expands myeloid suppressor cells that can suppress inflammatory and adaptive responses [22]. It is worth noting that the level of GM-CSF expression

appears to be important for the efficacy of cancer vaccines. While 30-300 ng/10^6 cells have augmented immune responses, too much has resulted in an expansion of myeloid suppressor cells and inhibition of adaptive immune responses [18,21,22]

The rationale for adding co-expressed GM-CSF to the DNA prime also comes from preclinical studies in macaques, using the SIV239 prototypes for the GEO-D03/MVA62B vaccine regimen (DNA prime co-expressing SIV proteins and rhesus GM-CSF) [13]. When comparing SIV infection using a heterologous SIVsmE660 moderate dose intra-rectal challenge, animals receiving two doses of the SIV239 vaccine DNA with co-expressed GM-CSF followed by two doses of the MVA/SIV239 boost had higher levels of protection from infection than animals given the same prime-boost regimen except without GM-CSF co-expression in the DNA component [13]. The two animals with breakthrough infections controlled their levels of plasma viremia. The co-expressed GM-CSF enhanced the avidity of the Env-specific IgG for the Env of the SIVsmE660 challenge stock, which in turn showed a strong correlation with prevention of infection ($r=0.9$, $p<0.0001$). In prior vaccine trials in macaques, avidity of anti-Env IgG also correlated with reductions in peak postchallenge viremia [23,24]. Therefore, the GM-CSF, when encoded within the DNA plasmid vaccine, can increase the protective efficacy of a DNA/MVA vaccine regimen, possibly through increased antibody avidity and/or other mechanisms.

The DNA/MVA concept advances the HIV-1 vaccine field by adding depth to the testing of heterologous prime-boost regimens using recombinant poxviruses as either prime or boost. This trial tests MVA (a vaccinia virus attenuated during passage on chicken cells), not the ALVAC (attenuated canary pox virus) or NYVAC (molecularly attenuated vaccinia virus) vectors that are being advanced in other trials [25]. MVA has significant differences in growth, the stimulation of the innate immune response and immunogenicity from NYVAC and ALVAC [25-29]. In clinical studies using recombinant MVA, NYVAC, and ALVAC vectors; all have effectively elicited CD4+ T cell responses; but MVA appears most effective at eliciting CD8+ T-cell responses [7,30,31]. Study of poxvirus vectors is relevant because a poxvirus vector (ALVAC) was used in the RV144 trial, the first clinical trial to show some efficacy [30].

Additionally GeoVax DNA and MVA vaccines express non-infectious VLP rather than shuffled proteins [31], demyristoylated Gag [31], or mosaic antigens [32] of other vaccines. The non-infectious VLP expressed by the GeoVax vaccine elicit both CD4+ and CD8+ T cell responses that are balanced between Gag and Env [7].

This proposed phase 1 trial is a dose escalation study in which 0.3 mg of GEO-D03 DNA and then 3 mg of GEO-D03 DNA will be used to prime immune responses which will be boosted using a constant MVA62B dose ($1x10^8$ $TCID_{50}$).

4.2 GEO-D03 DNA vaccine

The GEO-D03 DNA vaccine was developed from the JS7 plasmid DNA vaccine that was administered to healthy volunteers in HVTN 065 and 205 under BB-IND 12930. The GEO-D03 DNA vaccine differs from the JS7 vaccine by the insertion of a 435 base pair open reading frame for human GM-CSF in the position of a deleted *nef* sequence (Figure 4-1). JS7 is a 9.5 kb plasmid DNA composed of a 2.9 kb expression vector named pGA2 and a 6.6 kb vaccine insert expressing multiple HIV-1 clade B proteins from a single transcript that undergoes subgenomic splicing [33]. The vaccine insert expresses Protease (*PR*) and Reverse Transcriptase (*RT*) sequences of the BH10 strain of HIV-1; *tat, rev,*

vpu, and *env* from a recombinant of HXB-2 and ADA HIV-1 sequences; and *gag* from HIV-1 HXB-2. The vaccine is rendered non-infectious by deletion of the long terminal repeat (LTR), *vif, vpr,* and *nef* and the region of *pol* encoding integrase; and by the introduction of inactivating point mutations into packaging sequences for viral RNA and the protease, reverse transcriptase, strand transfer and RNase H domains of Pol. Addition of GM-CSF was achieved through insertion of a synthetic gene using standard recombinant DNA technology. With the addition of the GM-CSF gene, the size of the new plasmid (GEO-D03) is 9.9 kb. With the exception of the HIV-1 sequences, there are no known viral or oncogenic protein coding sequences within the GEO-D03 plasmid DNA. In transient transfections in 293T cells, GEO-D03 DNA expresses approximately 250 ng of human GM-CSF per 10^6 cells per 48 hours.

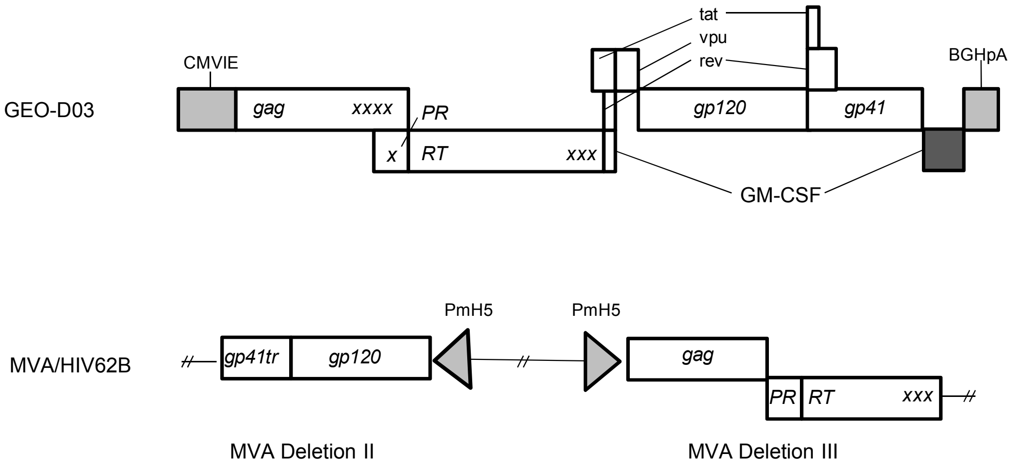

Figure 4-1 Schematic representations of the GEO-D03 DNA and MVA/HIV62B HIV-1 vaccines. CMVIE, CMV immediate early promoter; *gag*, HIV-1 gene encoding group specific antigens; *PR* and *RT*, protease and reverse transcriptase encoding regions of HIV-1 *pol*; *tat, vpu*, and *rev*, HIV-1 regulatory genes; *gp120* and *gp41*, surface and transmembrane subunit-encoding regions of HIV-1 *env* ; *gp41tr*, gp41 with a 115 amino acid C-terminal truncation; BGHpA, bovine growth hormone polyadenylation sequence; x, presence of inactivating point mutations in packaging sequences for viral RNA in Gag and the protease, reverse transcriptase, strand transfer and RNase H activities of Pol [9]; PmH5, the modified H5 early/late vaccinia promoter; deletions II and III, naturally occurring deletions in MVA.

The GEO-D03 DNA vaccine was manufactured under cGMP/GLP conditions by VGXI Inc., The Woodlands, Texas, using the E. coli DH10B-T1^R bacterial strain (Invitrogen Inc.). The vaccine is formulated in a buffer consisting of phosphate-buffered saline (PBS), 0.2 mM EDTA, and 1% v/v ethanol, pH 7.4. The placebo for the DNA vaccine is Sodium Chloride for Injection USP, 0.9%.

4.3 MVA62B vaccine

MVA62B is a highly attenuated vaccinia virus expressing HIV-1 *gag*, *pol*, and *env* genes from the same sequences used to construct the JS7 DNA. The MVA62B vaccine was constructed by introducing a Gag-Pol expression cassette into deletion III of MVA and an Env expression cassette into deletion II (Figure 4-1) [34]. Both expression cassettes use the mH5 early/late promoter for expression of vaccine inserts. The *pol* gene in the MVA62B vaccine contains the same mutations as found in the JS7 DNA vaccine with the exception of not including the inactivating point mutation in PR. The Env expression cassette contains an upstream start codon that has the potential for expressing a 33 amino

acid fusion protein comprised of 7 amino acid residues encoded by a multiple cloning site and the 26 C-terminal amino acids of Vpu. The upstream start codon attenuates the expression of Env. The sequences in the fusion protein have no matches in the genome database for the 7 amino acid sequence and its fusion outside of the known Vpu match.

The MVA62B was manufactured under cGMP/GLP conditions by BioReliance Ltd, Glasgow, Scotland, in specific pathogen-free chicken embryo fibroblasts (SPF CEF) and is formulated in a buffer consisting of PBS and 7.5% sucrose. The placebo for the MVA62B vaccine is Sodium Chloride for Injection USP, 0.9%.

4.4 Additional GeoVax vaccine product information

The GeoVax DNA and rMVA vaccines are unique among HIV/AIDS vaccines that have progressed in human trials in expressing native, transmembrane-bound Env (Figure 4-2).The inclusion of Env is important because preclinical studies have demonstrated improved protection in regimens that include Env as well as Gag-Pol immunogens [35,36].

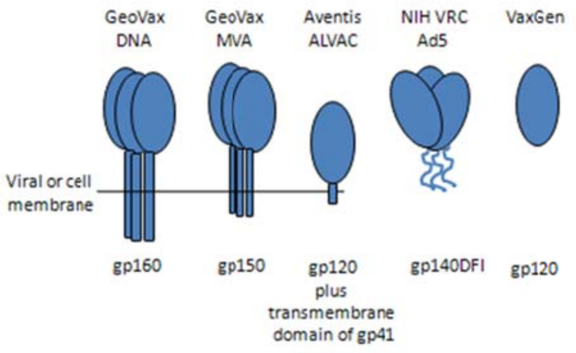

Figure 4-2 Expression of native Env by the GeoVax DNA and MVA vaccines compared with forms of Env expressed by other vaccines. From left to right, the schematic portrays (1) the gp160 trimeric membrane–bound full length form of Env expressed by the GeoVax DNA. Each member of the trimer consists of a globular gp120 subunit and a transmembrane gp41 subunit. (2) The gp150 trimeric membrane bound form of Env expressed by the GeoVax rMVA. In the gp150, the gp41 protein subunit is truncated for its 115 C-terminal amino acids. (3) The membrane-anchored monomeric gp120 form of Env expressed in the recombinant canarypox vector (ALVAC) tested in the RV144 trial in Thailand. The gp120 is fused to the transmembrane domain of gp41. (4) The trimeric secreted gp140DFI form of Env expressed by the NIH Vaccine Research Center (VRC) adenovirus vaccine being tested in HVTN 505. (5) The monomeric gp120 protein subunit used in the VaxGen vaccine trials in the US and Thailand and as a boost for the Aventis ALVAC vaccine in the RV144 trial in Thailand.

GeoVax holds the hypothesis that Ab elicited by native trimeric Env that is bound to a membrane can recognize Env on virions and infected cells. If this Ab binds tightly enough, it can initiate fragment crystallizable (Fc)-mediated mechanisms of protection such as complement (C')-mediated lysis, opsonization, antibody-dependent cellular cytotoxicity (ADCC), and antibody-dependent cell-mediated viral inhibition (ADCVI) [37]. In preclinical trials of SIV and SHIV prototypes of the GeoVax HIV vaccine using high dose challenges, the avidity of the anti-Env Ab response for gp160 Env has correlated with reduced levels of peak viremia. In the different trials, the reductions in peak viremia have varied from 100 to 10,000-fold, with the extent of the reduction

reflecting the relationship of the Envs in the immunogens and challenge viruses (Table 4-1). In repeat moderate dose challenge studies, the avidity of the anti-Env Ab for native Env has correlated with prevention of infection [13].

In macaques, the avidity of elicited Ab to Env by the JS7/MVA62B vaccine regimen has had good breadth for the Envs of incident isolates [24]. This breadth does not extend across clades, but does include incident isolates within clade B. The breadth in the avidity of the GeoVax-elicited sera for Envs of incident isolates is consistent with the previously demonstrated breadth of C'-mediated lysis [38,39], ADCC [40-42], and ADCVI [43] activities in patient sera for patient isolates.

Table 4-1 Summary of association between avidity and protection[1]

Vaccine Env / Challenge Env	Relationship of Envs	Type of challenge	Route	Avidity (range)	Protection	Correlation between avidity and protection
ADA/162P3 [24]	85% homology, HIV-1 Envs	Single high dose	IR	30-60	Up to 100X reduction in peak viremia	r=-0.8, p<0.001
89.6/89.6P [23]	89.6P is a neutralization resistant mutant of 89.6, HIV-1 Envs	Single high dose	IR	20-50	Up to 1000X reduction in peak viremia	r=-0.8, p<0.0001
239/251 [44]	Closely related neutralization resistant Envs, SIV Envs	Single high dose	IR	15-45	Up to 10,000x reduction in peak viremia	r=-0.6, p<0.01
239/E660 [13]	83% homology, SIV Envs	Repeat moderate dose	IR	30-50	70% Prevention of infection	R=0.9. p<0.0001

[1]Avidity is measured in duplicate enzyme-linked immunosorbent assays (ELISAs), one subjected to a 1.5 M sodium thiocyanate (NaSCN) wash, and one to a PBS wash. The avidity index is the dilution at which the NaSCN-washed sample has an optical density (OD) of 0.5 divided by the dilution at which the PBS-washed sample has an OD of 0.5 x 100.

Prior examples of vaccine for which the avidity of an Ab response has been found to be important for protection include the conjugate vaccines against Haemophilus influenzae type B (Hib) [45] and Streptococcus pneumonia (pneumococcus) [46]. These vaccines convert T-cell–independent to T-cell–dependent immunogens and allow Ab stimulated by polysaccharides to undergo affinity maturation in children under two years of age. The avidity of the Ab responses elicited by these vaccines is key to their protective activities. Thus, preclinical studies using SIV and SHIV prototypes of the GeoVax vaccines are not the only studies to show that Ab avidity is important in vaccine-mediated protection. The measurement of avidity for HIV-1 immunogens may be of particular importance because

of the slow maturation of Ab avidity to the highly glycosylated Env [47]. For most viral targets, avidity maturation is sufficiently rapid that it has not needed measurement.

Neutralizing Ab has been the gold standard assay for Ab for an HIV vaccine. However, studies with neutralizing monoclonals have suggested that these Abs can have higher *in vivo* protective activity than predicted from their *in vitro* neutralizing activity [48-50]. These findings are consistent with *in vivo* Ab-mediated protection being augmented by factors that are not operative in *in vitro* neutralization assays. GeoVax suggests that these *in vivo* factors involve mechanisms of virolysis and cytolysis that are initiated by Ab binding to and tagging virus and infected cells using Fc region-mediated mechanisms of destruction that include C'-mediated lysis, opsonization, ADCC, and ADCVI [37].The efficiency with which these mechanisms work is likely tied to the avidity with which an Ab binds to Env on virus and cells. Notably the GeoVax vaccines are the only vaccines in phase 2a clinical development that express Env as a membrane-bound trimer.

4.5 Trial design rationale

HVTN 065 tested injection of 3 mg of JS7 DNA followed by $1x10^8$ $TCID_{50}$ of MVA62B in a DDMM or DMM regimen and MVA62B alone (without DNA) in the MMM regimen (see Table 4-4). As shown in Section 4.9.1, the results of HVTN 065 indicate that two DNA primes are needed for maximal T cell responses. Temporal studies on Ab responses showed the third MVA in the MMM regimen increased anti-Env Ab titers by about 4-fold compared to the DDMM group whereas after two MVA immunizations, both the DDMM and the MMM regimens had similar titers of anti-Env Ab [7]. HVTN 065 also demonstrated that higher antibody responses occurred in both the DMM and MMM groups, which had a 4 month interval between the last two MVA doses, compared to the DDMM group that had only a 2 month interval between all vaccinations. Anti-Env binding antibody and neutralization (to MN) response rates and titers were highest in the MMM group, lowest in the DDMM group and intermediate in the DMM group (see Figure 4-13). The results of HVTN 065 suggest that three MVA injections may be needed for optimal Ab responses. They also suggest that a longer (4 month) interval between the last two MVA vaccinations may improve antibody responses.

Furthermore, recent data from influenza clinical trials [51] have indicated that a longer interval between the DNA priming and monovalent inactivated vaccine (MIV) boosting (or heterologous MIV priming and boosting, [52]) results in significantly higher antibody response rates and titers (HAI and neutralization) compared to a shorter interval (24 versus 4 weeks). Additionally, the kinetics of the influenza study VRC 310 demonstrate that antibody responses begin improving at 3 months, with functional activity (avidity) beginning to improve at 4 months and epitope breadth at 6 months (Barney Graham, VRC personal communication). Earlier studies of envelope subunit HIV vaccines [1,2] and pneumococcal vaccine [3] have also demonstrated that a rest period is needed to maximize the humoral immune response to these vaccines.

The elicitation of higher anti-Env Ab titers is desirable because of the likely importance of Ab in the enhanced protection against infection elicited by the GM-CSF adjuvanted SIV DNA/MVA vaccine regimen and the presumed importance of antibody in the protection seen in the ALVAC prime gp120 protein boost RV144 Thai trial [53]. Therefore, this trial will compare the antibody responses generated by 2 compared to 3 MVA boosts, as well as the effect of a longer interval between the second-to-last and last MVA dose.

4.5.1 Dose (amount and number)

In Group 1 the GEO-D03 DNA will be dosed at 0.3 mg which is 1/10th of the full DNA dose as this is a first in human dosing for this particular DNA product. Group 2 and Group 3 participants will be injected with the full dose of 3 mg. The rationale for the dosing choice of DNA is supported from data with the parent product JS7 DNA which was dosed at 0.3 mg first in human, then 3 mg thereafter. This dose was found to be well tolerated and immunogenic in participants in HVTN 065 and Part A of HVTN 205.

The dose selected for MVA62B is identical to the full dose used in HVTN 065 and HVTN 205 which was shown to be safe, well tolerated, and immunogenic.

4.5.2 Schedule

In Group 1 the vaccines will be administered in 2 month intervals, following the precedent established in the clinical trials HVTN 065 and HVTN 205. The GEO-D03 DNA vaccine or placebo will be injected at 0 and 2 months and then the MVA62B vaccine boosts or placebo will occur at 4, 6 and 8 months. In HVTN 065, the low dose vaccine group used one-tenth doses of both the DNA and the MVA. In this trial, only the DNA vaccine is initially injected with the lower dose – both for safety purposes (the full MVA dose safety profile has already been established), and for relative immunogenicity comparisons.

In version 1.0 of this protocol, Group 2 was also designed with the administration of all vaccines occurring at 2 month intervals. Subsequently, reconsideration of the potential beneficial effect of a longer interval between the second-to-last and last MVA vaccines on antibody responses, as well as an interest in evaluating the effects of 2 versus 3 MVA vaccines with a longer dosing interval in both groups, led to a redesign of the study schema and dosing schedule. In version 2.0, participants who enrolled into the original Group 2 will be split into a revised Group 2 and Group 3. In both groups, the GEO-D03 DNA or placebo will have been injected, under version 1.0 of the protocol, at 0 and 2 months. MVA62B or placebo will then be injected at 4, 6 and 10 months in Group 2 and at 4 and 8 months in Group 3, thus creating a 4 month interval between the last two doses in each group. Some participants in Groups 2 and 3 may receive the Month 4 injection under version 1.0 of the protocol.

4.5.3 Choice of placebo

The placebo product for both the GEO-D03 DNA vaccine and the MVA62B vaccine is Sodium Chloride for Injection USP, 0.9%.

4.6 Plans for future product development and testing

Based on the results of HVTN 065, the full dose DDMM GeoVax regimen and subsequently the MMM regimen were chosen to move forward into a phase 2a study (HVTN 205), which is currently in progress at 11 US and 2 Peruvian sites. In the meantime, additional macaque challenge data suggest that the GM-CSF-adjuvanted vaccine provides protection against infection and virologic control among vaccinated animals with breakthrough infection. Therefore, if this prime-boost comparison proves to be clinically safe and immunogenic, this combination prime-boost strategy would be

GeoVax's primary regimen to take forward into an efficacy trial. Future decisions would be made about the relative merits of MVA versus other poxvirus candidates (eg, ALVAC, NYVAC). However, this MVA product is the only poxvirus product that is currently being developed for use in clade B regions of the world.

4.7 Preclinical safety studies

Toxicology testing of the GEO-D03 plasmid DNA vaccine alone or in combination with MVA62B vaccine was not required by the FDA due to a number of factors. First there exist extensive toxicology studies performed in rabbits with the precursor DNA vaccine (JS7) and MVA62B vaccine. Second, there is a substantial body of safety data on the prior use of GM-CSF in humans as a recombinant protein, in tumor cell vaccines expressing GM-CSF and as a DNA vaccine. Third, the compilation of the human safety data for the JS7/MVA62B vaccine regimen has also been favorable. Finally, studies in rhesus macaques using the SIV prototype vaccine co-expressing GM-CSF demonstrated an excellent safety profile as well and these data are described here.

4.7.1 Preclinical safety of data in rhesus macaques

In preclinical studies in rhesus macaques, GM-CSF co-expressing and non-GM-CSF co-expressing prototypes of the JS7/MVA62B and GEO-D03/MVA62B were tested for the ability to prevent infection by a repeated moderate dose intrarectal challenge with SIVE660.

4.7.1.1 Hematology and clinical chemistry values

Standard hematology and serum chemistry analyses in conjunction with physical exams were performed on macaques receiving two unadjuvanted DNA followed by two MVA immunizations (DDMM regimen) or two GM-CSF-co-expressing DNA immunizations followed by two MVA immunizations (DgDgMM regimen). Serum chemistry and hematology analyses were completed at multiple timepoints with a specific focus on the hematology testing, which was completed three times within the 14 day period following each vaccination. The emphasis on hematology testing was deemed most reasonable because GM-CSF affects the *in vivo* production of multiple types of leukocytes. Serum chemistry and hematology analyses were also completed for animals that became infected with SIV to determine if the administration of the vaccines predisposed animals to any unanticipated pathogenesis.

All animals, except one that was euthanized due to self mutilation, remained healthy throughout the vaccine portion of the study and gained body weight. Analysis of the hematology data did not reveal any trends associated with the use of the GM-CSF supplemented vaccine. With the exception of a limited number of individual timepoints, values were generally observed to be within normal ranges throughout the study for both groups of animals. Declines in red blood cell counts, and other associated analytes early in the study for both groups were observed but levels recovered later in the study. It is believed that the erythrocyte reductions early in the study were caused by the larger amounts of blood, relative to body weights, drawn during this phase of the study. A more limited analyses of serum chemistry data provided similar results. With the exception of serum glucose and phosphorous levels, which decreased and increased, respectively, values were generally observed to be within normal ranges throughout the study for both groups of animals. The decrease over time in serum glucose levels is thought to reflect variable processing of serum samples. The increased levels of phosphorous may be

related to vaccine administration in general or the immune responses that were induced. However, the responses were observed in both vaccine groups (with and without the GM-CSF component) and returned to starting levels when the vaccine phase was completed and as such appears unlikely to be associated with administration of GM-CSF (in-house Macaque Technical Report).

4.7.1.2 Measurement of antibodies specific to GM-CSF in rhesus macaques

Rhesus macaques were tested for the presence of GM-CSF-specific antibodies to determine if an immune response was induced to the GM-CSF protein expressed by the plasmid. Antibodies to the cytokine were measured using an ELISA. Serum samples were obtained prior to the first immunization, 2 weeks after the administration of the DNA vectored vaccines and at week 36, prior to the infectious challenge part of the study. The tests did not reveal detectable levels of GM-CSF-specific antibody suggesting that the co-expressed GM-CSF had not elicited an antibody response.

4.7.2 Preclinical safety studies for JS7 and MVA62B vaccines in rabbits and guinea pigs

Preclinical safety studies on the JS7 precursor for GEO-D03 DNA have been conducted in New Zealand White (NZW) rabbits both in the absence and presence of a MVA62B boost. MVA62B has also undergone standard toxicology testing in rabbits. These studies are summarized in Table 4-2 and described in more detail in the Investigator’s Brochure.

Table 4-2 Summary of toxicology studies using JS7 DNA and MVA62B vaccines

Study	Test Article	Lot used		Dose	Immunization Schedule	Sacrifice
		DNA	MVA			
6-week IM toxicity in NZW rabbits with 4-week recovery	JS7 DNA (20 rabbits or 10/sex) vs. Control (20 rabbits or 10/sex)	BD030021-B	NA	JS7 DNA: 4.02 mg; Resuspension buffer: 0 mg	SD 1, 15, 29, 43 IM (0.75 mL at 2 sites for total of 1.5 mL)	SD 44, 71
9-week IM toxicity in NZWrabbits	MVA62B alone	NA	1129.02	1x10^8 PFU	SD 1,22,43,64	SD 66, 78
13-week IM toxicity in NZW rabbits with 2-week recovery	1) JS7 DNA prime and MVA62B boost<br><br>2) MVA62B alone	BD030021-C	1129.02	1) JS7 DNA: 2.9 mg<br>2) MVA62B: 1x$10^{8.3}$ $TCID_{50}$	1) JS7 DNA on SD 1, 22 IM MVA62B on SD 43, 64, 85<br>2) MVA62B on SD 1, 22, 43, 64 IM	1) SD 87 (M), 88 (F), 99<br>2) SD 66, 78
60-day Biodistribution in NZW rabbits<br><br>Injection site integration	JS7 DNA (30 rabbits or 15/sex) vs. Control (6 rabbits or 3/sex)<br><br>Integration analysis included in study	BD030021-B	NA	JS7 DNA: 4.02 mg; Formulation buffer:0 mg	SD 1 (0.5 mL IM at 3 sites/animal)	SD 8,28,60
Toxicity in guinea pigs	JS7 DNA (9 animals, 3 males per group)	BD030021-B<br>BD030021-A	NA	0 mg (buffer), 1.5 mg, or 15 mg	SD 1	SD 8

SD = Study Day
PFU = plaque forming units
vs. = versus

4.7.3 Preclinical safety studies of JS7 DNA vaccine

4.7.3.1 Toxicology study of JS7 DNA vaccine in NZW rabbits

A study was performed to determine the potential toxicity of the JS7 DNA vaccine when administered IM 4 times during 6 weeks to male and female NZW rabbits with a 4-week recovery period. Two groups (10/sex/group) received IM injections of resuspension buffer control or 4.02 mg/dose of JS7 DNA on Days 1, 15, 29, and 43. Animals were sacrificed on Day 44 or Day 71. Parameters evaluated included mortality, clinical signs of toxicity, and dermal Draize observations for skin irritation, body weights, body temperature, food consumption, ophthalmologic parameters, clinical pathology (hematology, serum chemistry, coagulation, and urinalysis) values, immunogenicity, organ weights, bone marrow evaluation, gross pathology, and histopathology.

Treatment with the JS7 DNA vaccine did not cause mortality or affect the general health of the animals. There were no abnormal clinical observations or observations in rabbit skin irritations, and there were no treatment-related effects on body weights, food consumption, body temperature, or ophthalmologic parameters. There was no apparent test article-related effect on measured clinical pathology with the exception of white blood cell count (WBC) in female but not male rabbits.

The treatment group females tended to have lower WBC values mostly due to lower numbers of circulating lymphocytes. While this effect was not seen in all of the females, it was most likely due to the vaccine causing redistribution of the circulating lymphocytes

in the females; however, it was not considered to be an adverse effect since these rabbits were essentially healthy with no clinical signs or significant histopathological findings other than the observed bone marrow response and some minimal inflammation at the injection site. The lower WBC values in the females were considered to be an incidental finding without any biological significance.

There was a possible test-article-related effect on the bone marrow. The myeloid: erythroid (M:E) ratio was significantly lower in the treated females on Day 44, which correlated with the lower WBC observed in the peripheral blood. The lower M:E ratio in this group was due to 2 of the 5 rabbits having myeloid cell counts lower than the lowest value for the control values. It is unknown if this is a test article-related effect or due to individual animal variation, as it was not observed in the male rabbits or upon repeat testing on Day 71. All subjectively had normal to low normal numbers of megakaryocytes, except one male rabbit that subjectively had decreased numbers of megakaryocytes on Day 44 only.

Based on the results of this study, IM administration of the JS7 DNA vaccine at 4.02 mg/dose 4 times during 6 weeks to male and female NZW rabbits was well tolerated. The only possible treatment-related effect was lower WBC count and M:E ratio in treated females. The effect on M:E ratio was resolved after a 4-week recovery period.

4.7.3.2 Biodistribution study of JS7 DNA vaccine in NZW rabbits (single dose)

A 60-day IM biodistribution study of the JS7 DNA vaccine in NZW rabbits was performed. A total of 36 animals were inoculated. Fifteen animals per sex were assigned to the test article (JS7 DNA vaccine) group and 3 animals per sex were assigned to the control (resuspension buffer) group. Animals received 0.5 mL in 3 IM injection sites (4.02 mg/mL [calculated]) injection of test article or control article in the upper and lower right hind leg and the upper left leg.

The animals were observed daily for mortality, morbidity, general health, signs of toxicity, and food consumption. Treatment with the JS7 DNA vaccine did not cause mortality or affect the general health of the animals. There were no treatment-related effects on clinical observations, body weights, or food consumption.

Results of the polymerase chain reaction analysis determined that the JS7 DNA vaccine remained localized in the muscle and skin at the site of injection and was not distributed to other organs of the body. The plasmid vaccine did persist at the injection site throughout the 60-day study, but the plasmid copy level decreased markedly over the course of the study from Days 8 through 60. All other tissues tested were negative at all timepoints, except one sample of popliteal lymph nodes contained a quantifiable number of copies of the plasmid vaccine at Day 29. No plasmid was detected in popliteal lymph nodes collected at Day 60.

Subsequently an injection-site muscle integration study was undertaken which concluded that there was no detectable integration of the plasmid into the genomic DNA of these animals.

4.7.3.3 Injection site muscle integration study of JS7 DNA vaccine

Twelve injection-site muscle samples with quantifiable plasmid copy levels and 2 control samples taken at the 60-day timepoint of the biodistribution study were analyzed.

All samples tested below the limit of detection for integration. Therefore, it is concluded that there was no detectable integration of the plasmid into the genomic DNA of these animals.

4.7.4 Preclinical safety studies of MVA/HIV48 and MVA/HIV62B

4.7.4.1 Safety of prototype vaccines JS2 DNA and MVA/HIV48 in NZW rabbits

The JS2 DNA and MVA/HIV48 prototype vaccines for the JS7 DNA and MVA/HIV62B vaccines were studied in a prime-boost toxicity trial and are described further in the Investigator's Brochure.

All animals survived until their scheduled necropsy. Some MVA-related effects included a decrease in erythropoietic parameters(red blood cells, hemoglobin, and hematocrit), an increase in WBC and corresponding lymphocyte and/or monocyte count, and increase in globulin and a decrease in albumin and albumin/globulin ration, and an increase in creatinine phosphokinase (CPK).

Other effects seen in animals vaccinated with 5×10^{8} PFU MVA/HIV48 IM were (1) decreased body weights (males only), which correlated to decreased food consumption (males and females) on the days of IM injection; (2) enlarged lymph nodes and increased spleen hemorrhage; and/or (3) lymphoid hyperplasia occurred in MVA-treated animals. An increase of injection site irritation was observed in animals vaccinated intradermal (ID) and was supported by microscopic evaluation.

The maximum tolerated dose for MVA/HIV48 administered IM in rabbits once every 3 weeks for 5 injections or once every 3 weeks for 3 injections following 3 priming injections with 4 mg plasmid JS2 DNA is considered to be at least 5×10^{8} PFU/injection. The maximum tolerated dose for MVA/HIV48 administered ID in rabbits once every 3 weeks for 5 injections is considered to be a least 1×10^{8} PFU/injection. The No-Observed-Adverse-Effect-Level (NOAEL) was not determined because only 1 dose level in each regimen was tested for toxicity in this study.

4.7.4.2 Safety of JS7 DNA and MVA/HIV62B vaccines in NZW rabbits

A toxicology study of JS7 DNA vaccine prime and MVA/HIV62B vaccine boost was conducted. The purpose of the study was (1) to determine the potential toxicity of the JS7 DNA vaccine when administered twice by IM injection followed by 3 IM injections of MVA/HIV62B during a 12-week period and (2) to determine the potential toxicity of MVA/HIV62B alone when administered 4 times by IM injection during an approximately 9-week period to male and female NZW rabbits. The persistence, reversibility, or delayed onset of effect was assessed during a 2-week, no-treatment recovery period. Two control groups (10/sex/group) received IM injections of matching placebos using the identical dosing schedule (Table 4-3).

Table 4-3 Study design for 13-week IM toxicity study of MVA/HIV62B and JS7 DNA vaccines in rabbits

Group	Treatment	Dose level	Dosing schedule	Sacrifice		Number of rabbits	
						Male	Female
1	Control for MVA/HIV62B	0	Day 1, 22, 43 and 64	Day 66	Day 78	10	10
2	MVA/HIV62B	1×10^8 $TCID_{50}$	Day 1, 22, 43 and 64	Day 66	Day 78	10	10
3	Resuspension buffer	0	Day 1 and 22	Day 87	Day 99	10	10
	Control for MVA/HIV62B	0	Day 43, 64 and 85				
4	JS7 DNA	3 mg	Day 1 and 22	Day 87	Day 99	10	10
	MVA/HIV62B	1×10^8 $TCID_{50}$	Day 43, 64 and 85				

Main-phase animals were sacrificed on Day 66 or 78 whereas the respective recovery-phase animals were sacrificed on Day 87 or 99. Parameters evaluated included mortality, clinical signs of toxicity, body weights, food consumption, and dermal Draize observations of the injection sites, ophthalmology, body temperature, clinical pathology (hematology, serum chemistry, coagulation, and urinalysis), organ weights, bone marrow evaluation, gross pathology, histopathology, and immunogenicity.

Administration of the JS7 DNA and MVA/HIV62B vaccines did not cause mortality or affect the general health of the animals. There were no abnormal clinical or Draize observations or treatment-related effects on body weights, food consumption, body temperature, or ophthalmology.

There was no apparent test-article-related effect on measured clinical pathology variables following administration of MVA/HIV62B vaccine only. However, clinical pathology results after administration of JS7 DNA vaccine followed by MVA/HIV62B vaccine were unclear, in that lower numbers of platelets and reticulocytes, and, indirectly, red blood cells because of lower hemoglobin and hematocrit, were noted in both sexes following the first injection of MVA/HIV62B but not at later timepoints. The changes were not considered to be adverse events because they were minimal and within the laboratory's historical range. In addition, concurrent changes in various clinical chemistry variables were noted. These changes, which generally affected electrolytes, proteins, and triglycerides, were observed in females more often than in males. At the recovery timepoint, the changes were not observed in females but some changes were observed in males. These changes also were not considered to be adverse events because they were minimal and within the laboratory's historical range.

There was no apparent test-article-related effect on bone marrow following administration of MVA/HIV62B vaccine alone or following administration of JS7 DNA vaccine prime and MVA/HIV62B vaccine boost. On Day 66 and 87, there were no statistical differences in the M:E ratio between control and treated rabbits. The rabbits had ordered synchronous maturation of both myeloid and erythroid cells. On Day 78, male recovery-phase animals given MVA/HIV62B vaccine had a statistically significant higher M:E ratio than control rabbits because the recovery-phase animals had a higher number of myeloid cells and a lower number of erythroid cells than the control animals. On Day 99, the M:E ratio between control and treated rabbits in the recovery group was not statistically different.

There were no apparent treatment-related effects on organ weights or on gross or microscopic pathology. There was minimal inflammation, with an occasional mild degree

of inflammation at injection sites in rabbits administered the MVA/HIV62B vaccine. The inflammation was less severe following a 2-week recovery period, which is indicative of a healing process. A few minimal to mild chronic or chronic/active inflammatory lesions were present at injection sites in treated rabbits dosed with both vaccines. These lesions at injection sites were considered to be typical host responses to a foreign body and not test-article related.

4.7.4.3 Safety of JS7 DNA plasmid vaccine in guinea pigs (USP88 Test)

This study was designed to determine the potential toxicity of the JS7 DNA plasmid vaccine when administered once by intraperitoneal (IP) injection to male guinea pigs as a release test for the vaccine.

Parameters evaluated included mortality, clinical signs of toxicity, and body weights.

The safety test result was deemed satisfactory as all animals survived the test period, no signs of toxicity were observed and all animals weighed more at the end of the test period than at the time of the injection.

4.7.5 Summary of pharmacology and toxicology studies

Toxicology studies completed using NZW rabbits revealed no significant treatment-related effects which failed to resolve during the recovery period. Biodistribution and integration studies revealed persisting DNA only at the site of injection with no evidence for genomic integration.

4.8 Preclinical immunogenicity studies

4.8.1 Immunogenicity of SIV prototypes of GEO-D03/MVA62B

Simian immunodeficiency virus (SIV) prototypes of GEO-DO3, both co-expressing and not co-expressing rhesus GM-CSF, have undergone safety, immunogenicity and challenge testing in rhesus macaques. Immunizations included 3 mg of DNA delivered at weeks 0 and 8 and $1x10^8$ PFU of MVA administered at weeks 16 and 24. Animals were tested for safety throughout the immunization and challenge phases of the trial and for the effects of the co-expressed GM-CSF on immunogenicity and susceptibility to infection. All animals remained healthy through the vaccination portion of the study and gained body weight. Furthermore, in the challenge phase of the study, the GM-CSF-adjuvanted vaccine had beneficial (not adverse) effects on protection against infection. Analysis of hematology data collected throughout the different phases of the study did not reveal any trends associated with the use of the GM-CSF supplemented vaccine. With the exception of a limited number of individual timepoints, values were observed to be within normal ranges throughout the study for both GM-CSF-adjuvanted and non-GM-CSF-adjuvanted groups of animals. Analysis of serum chemistry data showed no differences between groups. These data indicate that co-expression of the rhesus GM-CSF in the DNA prime as an adjuvant was safe in macaques, a species in which the expressed GM-CSF was bioactive. Furthermore, the data indicate that the co-expressed, bioactive GM-CSF was highly beneficial (no adverse effects) for an immunodeficiency virus challenge.

At 6 months post the last vaccination, a repeat moderate dose intrarectal challenge (Monkey Infectious Dose $(MID)_{30}$) with the heterologous SIVsmE660 was administered. SIVsmE660 has 91% Gag homology and 83% Env homology with the immunogen, a

genetic relationship that is overall similar to that of intraclade isolates within the current pandemic [54,55]. Twelve weekly challenges were given with challenges being stopped once animals scored as infected. 71% of the group primed with the GM-CSF co-expressing DNA were protected against infection whereas only 25% of the group primed with the non-adjuvanted DNA were protected (Figure 4-3 A).The two animals that became infected in the GM-CSF adjuvanted group achieved good post challenge control of *in vivo* viral replication (Figure 4-3 B). Therefore, the GM-CSF, when encoded within the DNA plasmid vaccine, increased the ability of the prototype SIV DNA/MVA vaccine to induce immune responses that prevented infection with a heterologous rectal viral challenge.

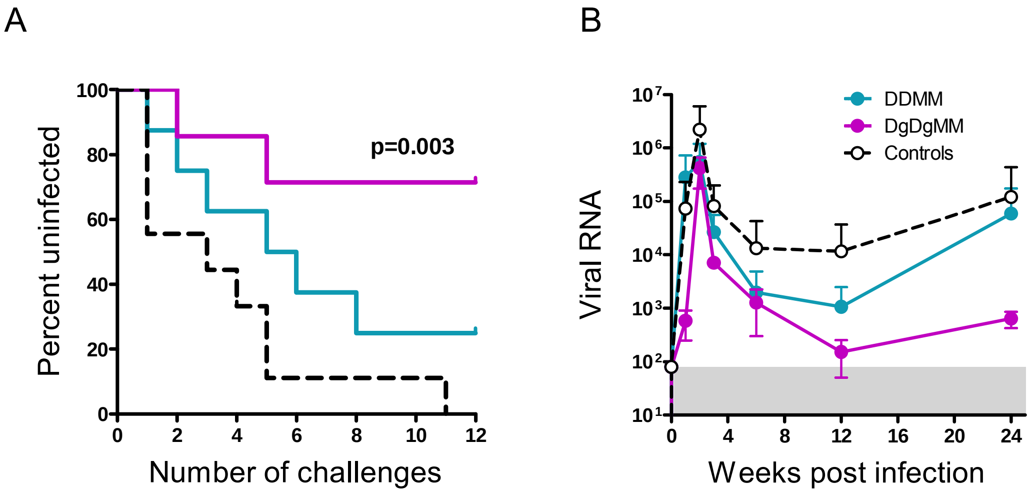

Figure 4-3 Protection against a repeated moderate dose rectal challenge. (A) Prevention of infection, (B) control of post challenge viremia in animals that became infected. Macaques primed with DNA in the presence (n=8, all A*01$^-$, one B*08$^+$ and one B*17$^+$) or absence (n=7, all A*01$^-$, one B*08$^+$ and one B*17$^+$) of co-expressed GM-CSF and unvaccinated controls (n=9, all A*01$^-$, B*08$^-$ and B*17$^-$) were given 12 weekly challenges with MID$_{30}$ of SIVsmE660. DNA vaccinations were administered at weeks 0 and 8 and MVA vaccinations at weeks 16 and 24. Challenge was 6 months later. D, SIV239 DNA, Dg, SIV239 DNA co-expressing rhesus GM-CSF. M, SIV239 MVA. P=0.003, difference between infection in the GM-CSF adjuvanted and unvaccinated group (log-rank Mantel-Cox) test. In B, Copies of viral RNA are given for the first 6 months post infection. To allow comparison of levels of virus in different animals, the last week at which virus was below detection, is considered as week 0. The background for detection of viral RNA was 80 copies per mL. Data are means.

Analysis of immune responses elicited by the DDMM and DgDgMM vaccine regimens revealed no differences in the magnitudes of anti-HIV CD4+ or CD8+ T-cell responses (data not shown) or the titers of Env-specific IgG (Figure 4-4A). However, the co-expressed GM-CSF did show a trend for eliciting enhanced Env-specific IgA responses in rectal secretions (Figure 4-4B). The co-expressed GM-CSF also enhanced the avidity of the Env-specific IgG response (Figure 4-4C), the neutralizing activity of the elicited antibody for easy to neutralize variants of E660 (Figure 4-4D) from the genetically complex E660 challenge stock, and antibody-dependent cellular cytotoxicity activity (ADCC) (Figure 4-4E) of the elicited Env-specific IgG.

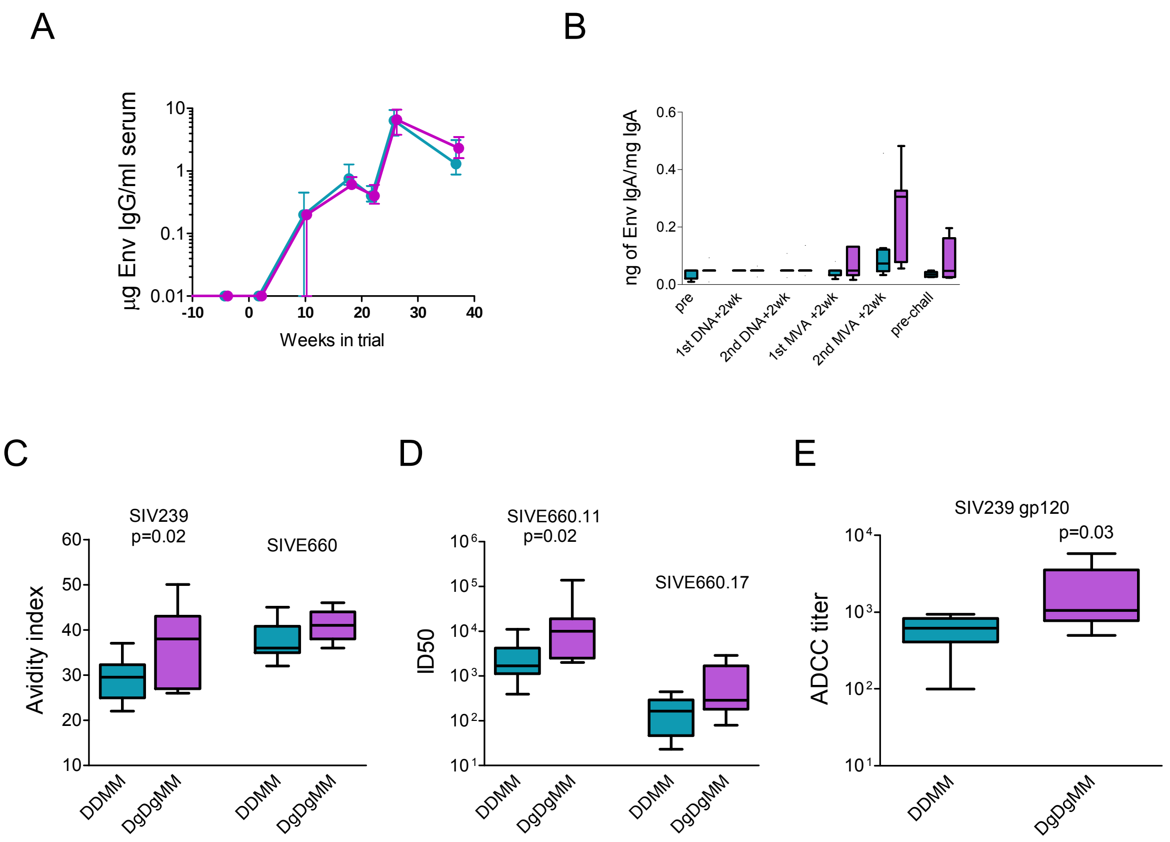

Figure 4-4 Humoral immune responses elicited by the GM-CSF-adjuvanted and non-adjuvanted SIV239 DNA/MVA vaccines. DNA priming immunizations were administered at weeks 0 and 8 and MVA booster immunizations at weeks 16 and 24. (A) Env-specific IgG responses measured in serum at preimmunization, 2, 10, 18, 21, 26, and 37 weeks in the trial. Micrograms of IgG are estimated relative to a standard curve of rhesus IgG. Values are medians $\pm$ interquartile ranges. (B) Tukey plots presenting Env-specific IgA responses in rectal secretions at preimmunization, 2 weeks after the indicated immunizations and prechallenge. IgA is presented as Env-specific IgA divided by total IgA. (C) Avidity indices for elicited IgG for the SIV239 Env of the immunogen and the SIVE660 Env of the challenge measured at 2 weeks after the second MVA immunization. Avidity indices increased with time in the trial and further increased post infection. (D) Neutralization titers for pseudotypes with two Envs molecularly cloned from the genetically diverse SIVE660 stock. Titers for SIVE660.11 were determined at 2 weeks post the second MVA boost; and, for SIVE660.17, at 13 weeks after the second MVA boost. Differences in overall titers for the two tested isolates reflect differences in the susceptibility of different isolates from the E660 quasispecies to neutralization and differences in the timing of assays. Titers are the reciprocal for the dilution of serum achieving an inhibitory dose 50 (ID_{50}) in the TZM-bl assay. (E) ADCC titers for SIVmac239 gp120 coated CEM.NKR_{CCR5} cells at two weeks following the second MVA boost. In panels *C* - *E*, Boxplots present median and 25th and 75th percentiles for responses. Target Envs and the significance for differences between the DDMM and DgDgMM regimens are indicated above boxplots. Statistical comparisons were made using a two-sided Wilcoxon's rank-sum test. Data for the DDMM regimen are presented in turquoise, and those for the DgDgMM regimen in fuchsia.

In earlier preclinical trials using prototypes of the GeoVax DNA and MVA vaccines, GM-CSF also enhanced Ab responses. In these trials, which used high dose rectal challenges, the avidity of anti-Env IgG correlated with reductions in peak post challenge viremia [23,24]. In one study that was followed long term, the GM-CSF adjuvant

correlated with fewer transient re-emergences (blips) of the vaccine controlled infection [56].

The avidity of the Env-specific IgG for the SIVE660 Env of the challenge virus correlated with the number of challenges to infection (r=0.9, p<0.0001, Spearman test) (Figure 4-5A). In contrast, titers of neutralizing and ADCC antibody activities and the presence of anti-Env IgA, which were higher in the GM-CSF-adjuvanted group, did not correlate with protection. Elicited T-cell responses also did not correlate with the number of challenges to infection. The correlation with avidity was specific for the Env of the challenge virus and was not observed for the Env of the SIV239 immunogen (Table 4-1). Protection did not correlate with the TRIM5α genetic restriction (Figure 4-5B).

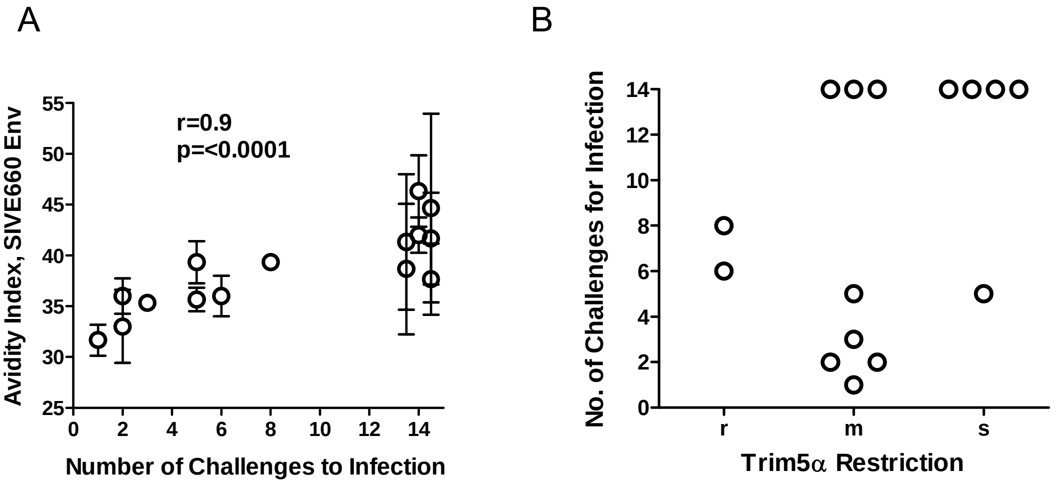

Figure 4-5 Avidity of the vaccine-elicited IgG for the Env of the challenge virus correlates with protection. (A) Significant correlation between avidity of the elicited IgG for the SIVE660 Env of the challenge virus and the number of challenges to infection. Data are presented as the mean + one standard deviation for 3 independent assays. Correlations were done using the two sided Spearman rank order statistical analysis. (*B)* The *TRIM5α* genotype of vaccinated rhesus macaques does not restrict the number of challenges to infection r, restrictive *TRIM5α* genotype (homozygous or heterozygous for *TRIM5αTFP* or *CYPA*); s, susceptible genotype (homozygous for *TRIM5αQ*); m, moderately susceptible (heterozygous for a restrictive and permissive allele). Animals that were not infected by the 12 challenges are plotted at challenge 14.

Following the last challenge, the 5 uninfected DgDgMM animals and 2 uninfected DDMM animals were regularly tested for evidence of infection, as measured by the presence of viral RNA in blood and anamnestic antibody responses to Env in serum. No evidence of infection was observed (Figure 4-6 and data not shown). At one year after the completion of the first series of SIVE660 challenges the animals were reboosted with MVA in preparation for a second series of SIVE660 challenges.

The third MVA boost transiently increased the magnitudes of both Ab and T cell responses. Following the third boost, magnitudes of both Ab and T cell responses expanded and then contracted (Figure 4-6). Peak titers of binding Ab were about 2-fold higher than those following the second MVA boost. At 13 weeks after the boost, a period during which the contraction of the Ab response had largely occurred, binding Ab responses remained about 2-fold higher than the contracted response after the second MVA boost. After the initial contraction, titers of binding Ab appeared to be stable with

time. In contrast to titers of binding Ab that showed sharp expansions and contractions with boosts, antibody avidity showed an overall slow rise with time. Following the third MVA boost, the avidity index of the Ab response was 11 units higher than after the second MVA boost. Env-specific IgA in rectal secretions underwent a sharp expansion and contraction reaching peak and contracted levels similar to those after the second MVA boost. CD4+ and CD8+ T cell responses also showed sharp expansions and contractions. Peak responses for these were the same (CD4+) or lower (CD8+) than after the second MVA boost. However, at 13 weeks after the boost, CD4+ responses showed less contraction, remaining 2 to3-fold higher than after the second MVA boost.

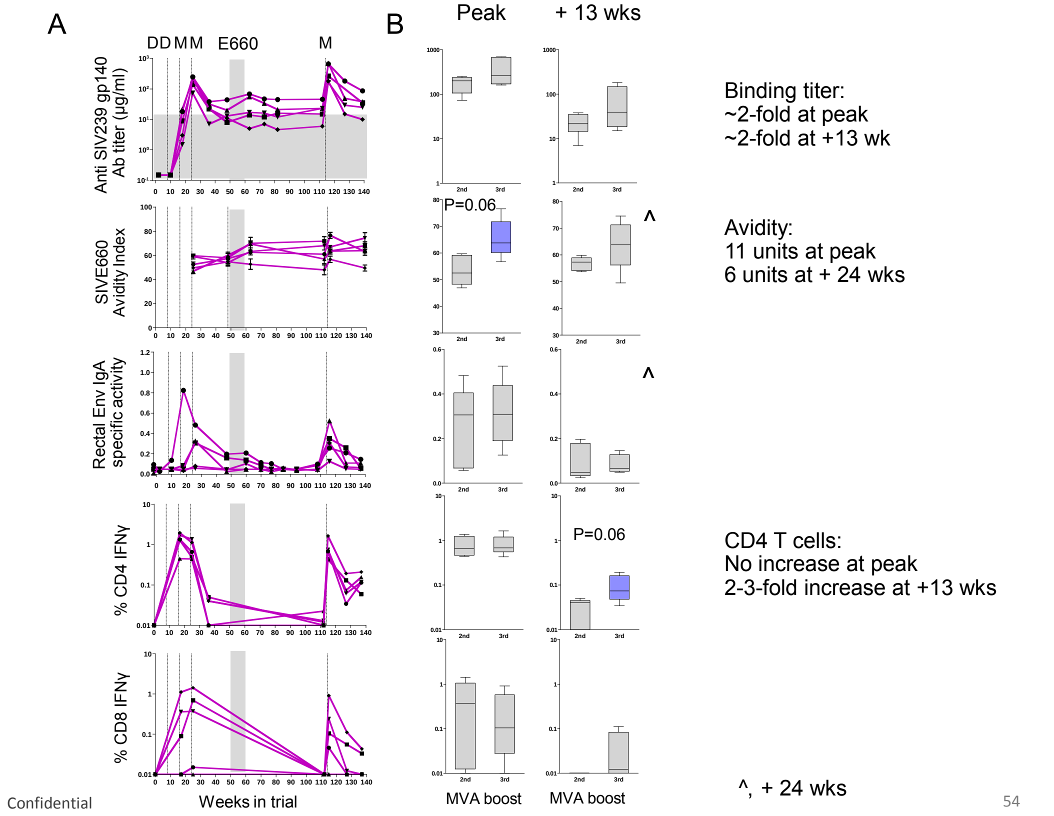

Figure 4-6 Patterns of immune responses up to the second series of challenges. Immune responses are shown for the five DgDgMM vaccinated animals that were not infected by the first series of exposures to SIV E660. (A) Temporal immune responses. The response being measured is shown on the y axis and the weeks in the trial, on the x axis. All data are medians. (B) Comparison of heights of responses at 2 and 13 or 24 (indicated with an ^) weeks after the second and third MVA boosts. Boxplots present median, 25th and 75th percentiles for responses. P values for responses with strong trends for significance are given in box plots. Statistical comparisons were made using a two-sided Wilcoxon's rank-sum test. Comments are for responses that the third boost increased to higher levels than elicited by the second boost.

Following the third MVA boost, animals were rested for 6 months and then subjected to a second series of 12 intrarectal SIVE660 challenges (Figure 4-7). With one exception, all animals were protected against the second series of 12 challenges. The exception was the DgDgMMM animal that had shown slowly falling titers of binding Ab and slowly falling avidity following the first series of E660 challenges (see Figure 4-6). This animal became infected at the tenth exposure.

After an approximately 3 month rest, survivors of the second series of E660 challenge were subjected to 5 to 7 low dose intrarectal SIV 251 (120 $TCID_{50}$) exposures followed by 12 high dose intrarectal SIV251 (650 $TCID_{50}$) exposures (Figure 4-7). All of the previously protected animals remained protected against the low dose SIV251 challenges. However, all became infected by the high dose challenges. One DDMMM animal and one DgDgMMM animal were infected by the fourth high dose challenge. The other DDMMM animal was infected by the tenth high dose challenge. Of the remaining three DgDgMMM animals, one was infected by the eleventh challenge and two by the twelfth challenge (Figure 4-8). The dose of SIV251 was raised because the low dose had infected only 1 of 5 unvaccinated controls in 7 exposures (data not shown). The high dose of SIV251 infected the four remaining uninfected controls within 4 exposures.

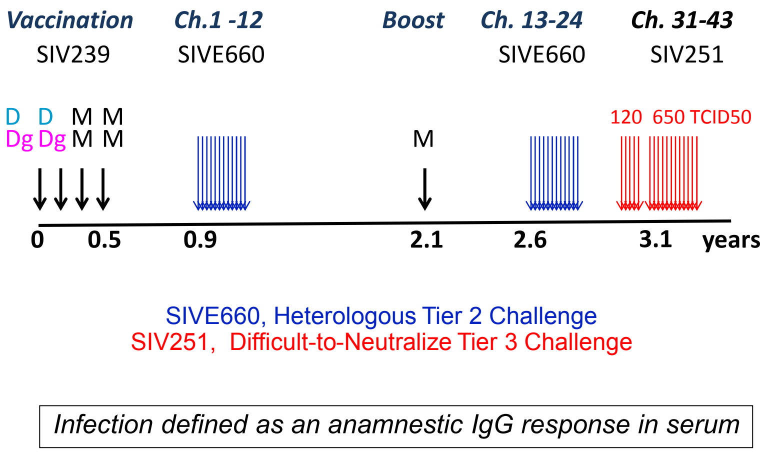

Figure 4-7 Schematic for serial intrarectal challenges for DgDgMMM and DDMMM vaccinated animals. Animals receiving the unadjuvanted DNA prime are presented in turquoise, and those receiving the GM-CSF co-expressing DNA prime, in fuchsia. SIVE660 challenges are indicated with blue arrows, and SIV251 challenges with red arrows. Low and higher dose SIV251 challenges are indicated in $TCID_{50}$ above their respective exposure arrows. All challenges were weekly, for 12 weeks; except for the low dose SIV251 which was administered between 5 and 7 times depending on the macaque. An animal was considered infected if sera contained an anamnestic Ab response to Env.

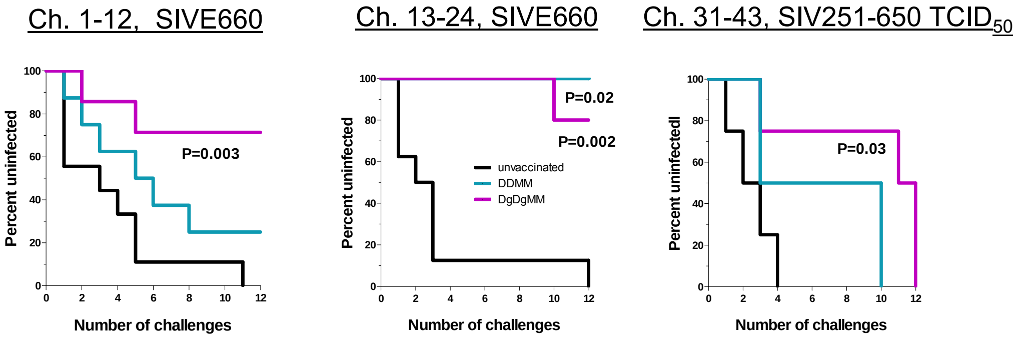

Figure 4-8 Results of serial challenges of DDMMM and DgDgMMM vaccinated animals. The series of challenges and time of initiation of the challenges are indicated above schematics**.** Statistics for the Kaplan-Meier curves use the log-rank Mantel-Cox test.

Analysis of temporal viremia in SIV251 infected animals revealed one animal in the DgDgMMM controlling its infection to below the level of detection (Figure 4-9). The other animals, in both vaccinated groups, showed approximately 10-fold lower medians for peak levels of viral RNA and 5-fold lower medians for setpoint levels of viral RNA (Figure 4-9) compared to unvaccinated controls. These results suggest that the elicited vaccine response had conferred the ability to exert partial control over break through infections.

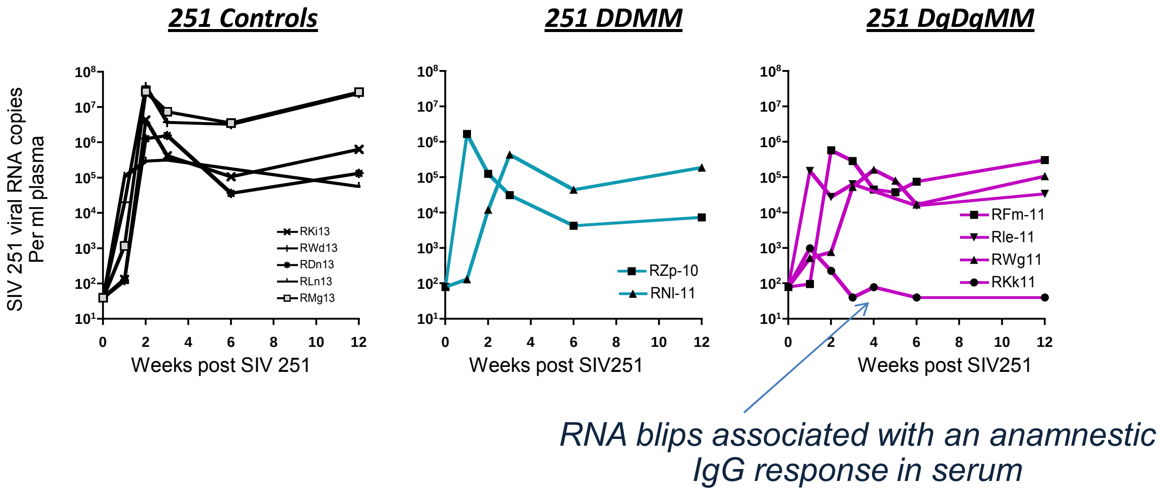

Figure 4-9 Temporal viremia in SIV251-infected animals. Copies of viral RNA are given for the first 3 months post infection. To allow comparison of levels of virus in different animals, the last week at which virus was below detection, is considered as week 0.

Avidity of Env-specific Ab. The avidity of Env-specific Ab increased with time in the trial (Figure 4-10). Just before the initial series of E660 challenges, the median avidity for the E660 Env was 35. Just before the second series of E660 challenges, it was 62 for the E660 Env. By the time of initiation of the high dose SIV251 challenge, the median avidity index was 78 for the SIV239 Env (an Env closely related to the swarm in the SIV251 stock). For both E660 challenges, avidity appeared to play a role in protection. However, for the difficult to neutralize 251 challenge, avidity did not appear to play the same protective role it had played for SIV E660.

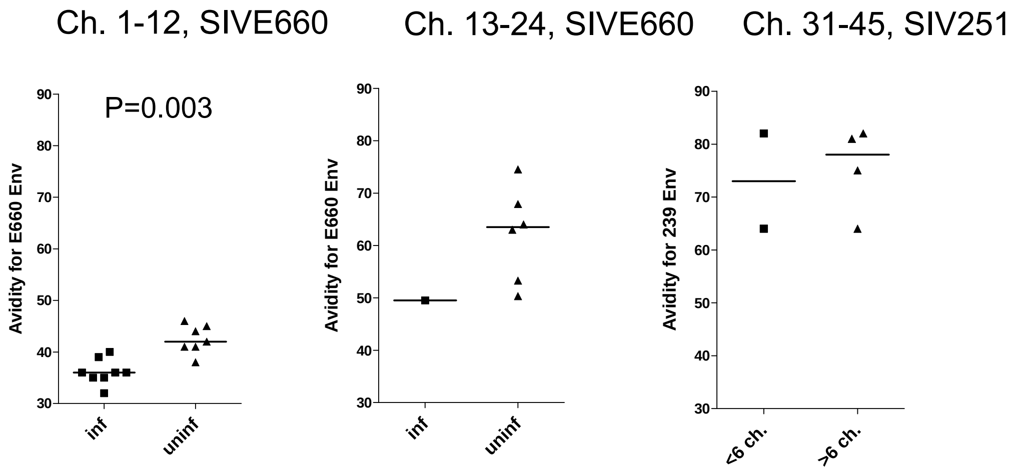

Figure 4-10 Avidity of Env-specific Ab at the times for the initiation of the different series of challenges. Within graphs, each symbol represents data for one animal. The horizontal lines are medians. The challenge series and virus are indicated above graphs. Statistics use the Mann-Whitney test. Ch. = challenges; inf = infected; uninf = uninfected.

4.8.2 Summary of preclinical immunogenicity and protection studies

Results from a heterologous DNA prime-MVA boost study using a SIV prototype of the GEO-D03 vaccine that co-expresses GM-CSF showed an impressive increased vaccine-induced prevention of infection from 25% in the non-GM-CSF to 71% in the GM-CSF co-expressing vaccine group. Co-expression of GM-CSF in the DNA prime enhanced the avidity of elicited IgG for SIV envelope glycoproteins, the titers of neutralizing antibody for easy to neutralize SIV isolates and antibody-dependent cellular cytotoxicity. The prevention of infection showed a strong correlation with the avidity of the elicited Env-specific antibody for the Env of the SIVsmE660 challenge virus ($r=0.9$, $p<0.0001$).

A late boost and further challenges of the protected animals revealed protection against a second series of E660 challenges and a substantial delay in infection by a fourth series of higher dose SIV251 challenges. The correlation between avidity and prevention of E660 infection that had been observed for the first series of E660 challenges, also appeared to apply to the second series of E660 challenges. However, avidity, which slowly rose over the course of the trial from indices of 30-50 at the initiation of the first series of

challenges to indices of 60-85 at the initiation of the fourth series of challenges (higher dose 251), did not appear to correlate with protection against the hard to neutralize SIV251 challenge.

4.8.3 Predictive value of SIV E660 challenge model

The predictive value of the SIVE660 and SIV251 challenge models for future clinical trials is not known. However the genetic relatedness of SIVE660 to the SIV239 vaccine are reasonable mimics of transmitted infections [54,55]. The SIVE660 challenge stock has neutralization characteristics of tier 2 isolates of HIV-1 [57]. However, in other preclinical vaccine trials using SIVE660 exposures, most breakthrough infections have been classified as tier 1. When one examines transmitted clade B HIV-1 isolates, transmitted isolates predominantly have tier 2 (not tier 1) characteristics [57]. SIV251 has proven to be very difficult to prevent. This is thought to reflect the high resistance of this virus to neutralizing Ab, a level of resistance that is rarely seen for HIV-1 [57].

4.9 Clinical studies

No previous clinical studies of the GEO-DO3 vaccine have been performed to date. Summarized here are the safety and immunogenicity results of prior clinical studies with the earlier generation DNA vaccine (JS7) and the MVA vaccine (MVA62B) that will be used in this trial.

4.9.1 Clinical studies of JS7/MVA62B

On January 26, 2006, NIAID submitted protocol HVTN 065 to a new BB-IND to evaluate the GeoVax JS7/MVA62B vaccine regimen for the prevention of HIV-1 infection (later assigned as BB-IND 12930). HVTN 065 was a randomized, double blinded, placebo controlled trial conducted at six clinical sites in the United States in adults aged 18-50 years (Table 4-4). The study was designed with 10 participants receiving one-tenth doses of the vaccines; specifically 0.3 mg of the JS7 DNA (D) and 10^7 $TCID_{50}$ of MVA62B (M) at 8 week intervals in the DDMM schedule. After a safety review of the 1/10th dose DNA vaccinations, 30 participants were randomized to receive full doses of the vaccines (3 mg and 10^8 $TCID_{50}$, respectively) in the DDMM sequence. Once the full dose DDMM regimen was demonstrated to be adequately safe and immunogenic, part B of the trial was started. This included the enrollment of 30 participants to receive full dose vaccines in the DMM or MMM sequences with immunizations administered at 0, 8 and 24 weeks. The placebo product used for all groups was saline and placebo participants were enrolled at the ratio of 1:5, placebo recipients: vaccines.

Table 4-4 HVTN 065 schema

			Dose		Vaccination schedule in weeks (days)			
					Prime		Boost	
Part	Group	N	JS7 DNA	MVA 62B	0 (0)	8 (56)	16 (112)	24 (168)
A	T1	10	0.3 mg	1×10^7 $TCID_{50}$	DNA	DNA	MVA	MVA
	P1	2	—	—	placebo	placebo	placebo	placebo
	T2	30	3 mg	1×10^8 $TCID_{50}$	DNA	DNA	MVA	MVA
	P2	6	—	—	placebo	placebo	placebo	placebo
B	T3	30	3 mg	1×10^8 $TCID_{50}$	DNA	MVA	—	MVA
	P3	6	—	—	placebo	placebo	—	placebo
	T4	30	—	1×10^8 $TCID_{50}$	MVA	MVA	—	MVA
	P4	6	—	—	placebo	placebo	—	placebo

Total: 120 = 100 vaccinees + 20 placebos

T = treatment
P = placebo

The vaccines were safe and well tolerated at both doses and using all schedules without severe reactogenicity (Figure 4-11). Local reactogenicity (pain, tenderness, erythema and induration at the injection site) and systemic reactogenicity (fever, malaise/fatigue, myalgia, headache, nausea, vomiting, chills, and arthralgia) with onset within 72 hours following vaccination were none to moderate. Axillary pain and lymphadenopathy were the most common adverse events (AE) considered to be related to the vaccines, with four cases of each occurring during the study. However, no pattern of systemic AE emerged during the study and no serious adverse events (SAE) related to the study vaccines were observed (one Grade 4 CPK elevation was probably not related; all other SAEs were deemed not related). The vaccines were well-tolerated, with an acceptable safety profile in this trial. There have been no cardiac events, including myopericarditis attributable to MVA vaccine in HVTN 065.

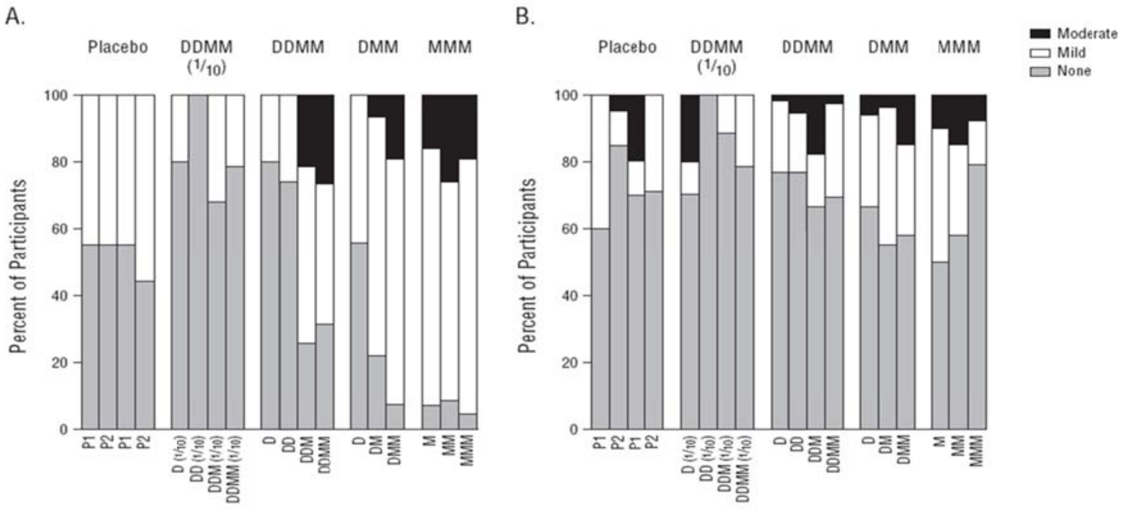

Figure 4-11 Reactogenicity of study vaccine regimens. The percent of participants with local pain and/or tenderness (A) or any systemic symptom (B) following each vaccine dose is shown. Reactions were graded as none, mild, moderate, or severe. The vaccine groups are given at the top of the schematics and the immunization status of groups at the bottom. D = DNA; M = MVA; P = placebo. The number of Ds and Ms indicate the number of immunizations, for example, DDM means two DNA and one MVA immunization.

Immunogenicity studies in HVTN 065 revealed that the low and high dose DDMM regimens elicited the highest frequency of T cell responders, whereas the MMM regimen elicited the highest titers and frequencies of Ab responses (Figure 4-12). Both antibody and T-cell responses were not detected following the DNA priming immunizations. However, following the MVA booster immunizations, subjects on the full dose DDMM regimen achieved a maximal CD4+ response rate of 77%, compared to a 43% response rate in the MMM arm. DNA also primed maximal response rates for CD8+ T cells, with the DDMM and MMM arms eliciting 42% and 17% CD8+ T cell responders, respectively. Subjects who received DDMM elicited maximal CD8+ T-cell response rates after the final vaccination (second MVA vaccination), compared to the MMM arm, in which maximal T-cell response rates occurred after the second MVA vaccination. A single DNA prime was not as effective as two DNA primes for eliciting maximal T-cell responses. The highest frequencies and titers of anti-Env Ab responses were elicited by the MMM regimen. Ab response rates for Env scored higher in the sera obtained from MMM immunized individuals in 5 different assays. These included 3 binding assays, a neutralization assay for HIV-1 MN and a commercial western blot.

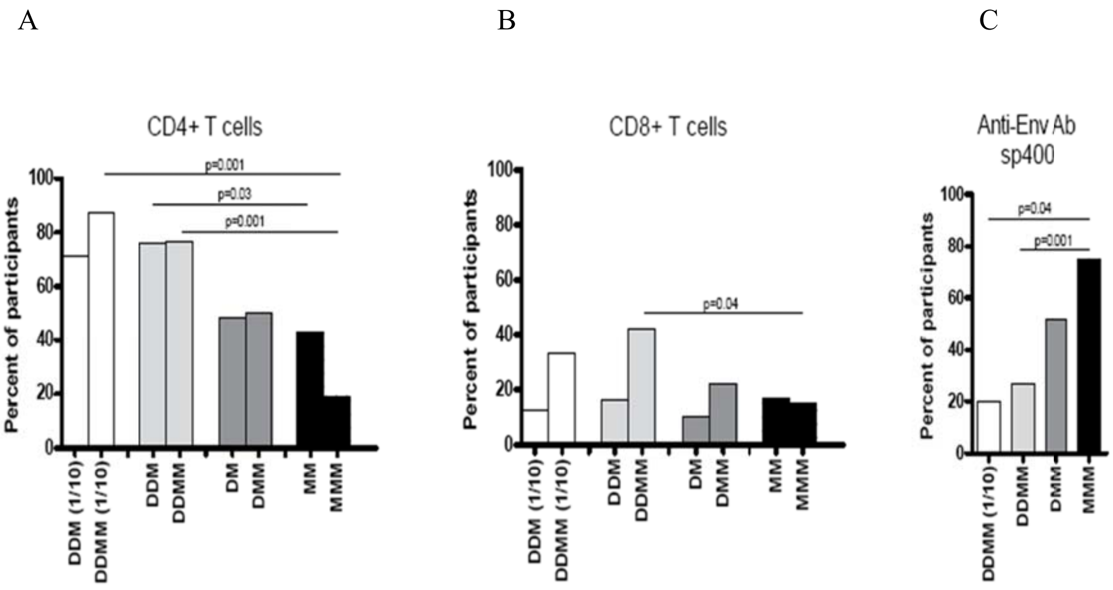

Figure 4-12 Immune response rates determined in HVTN endpoint assays. Response rates for CD4+T cells (A), CD8+T cells (B) and anti-Env Ab (C). Responses for CD4+and CD8+T cells are for responses to Gag, Env, or Pol measured as IFN-γ or IL-2 producing cells scored using intracellular cytokine staining (ICS) following stimulation with potential T cell epitope (PTE) peptide pools. Response rate for anti-Env Ab were measured using an ELISA for the sp400 peptide, a peptide representing the immunodominant region of gp41. Lymphocytes and sera for determining response rates were harvested at 2 weeks following immunizations. All assays were performed in HVTN laboratories on frozen samples. Letters at the bottom of schematics indicate group, and the immunization status of groups (see legend to Figure 4-11 for designations).

Analysis of titers of anti-Env Ab elicited by the different regimens revealed that the MMM regimen had elicited the highest titer of anti-Env binding Ab (Figure 4-13). This titer was about 4-times higher than elicited in the DDMM regimen. Interestingly after the final dose of MVA62B (the second injection) in the DDMM regimen, titers of anti-Env Ab were similar to those elicited after the middle dose of MVA (also the second injection) in the MMM regimen. This suggests that a DDMMM regimen might enhance anti-Env Ab titers over those elicited by the DDMM regimen.

In addition, compared to the full dose DNA/MVA regimen, the low dose regimen elicited similar rates of CD4+ and CD8+ T-cell responses, but lower rates of antibody responses. To maximize antibody responses, the full dose is the preferred regimen for this study.

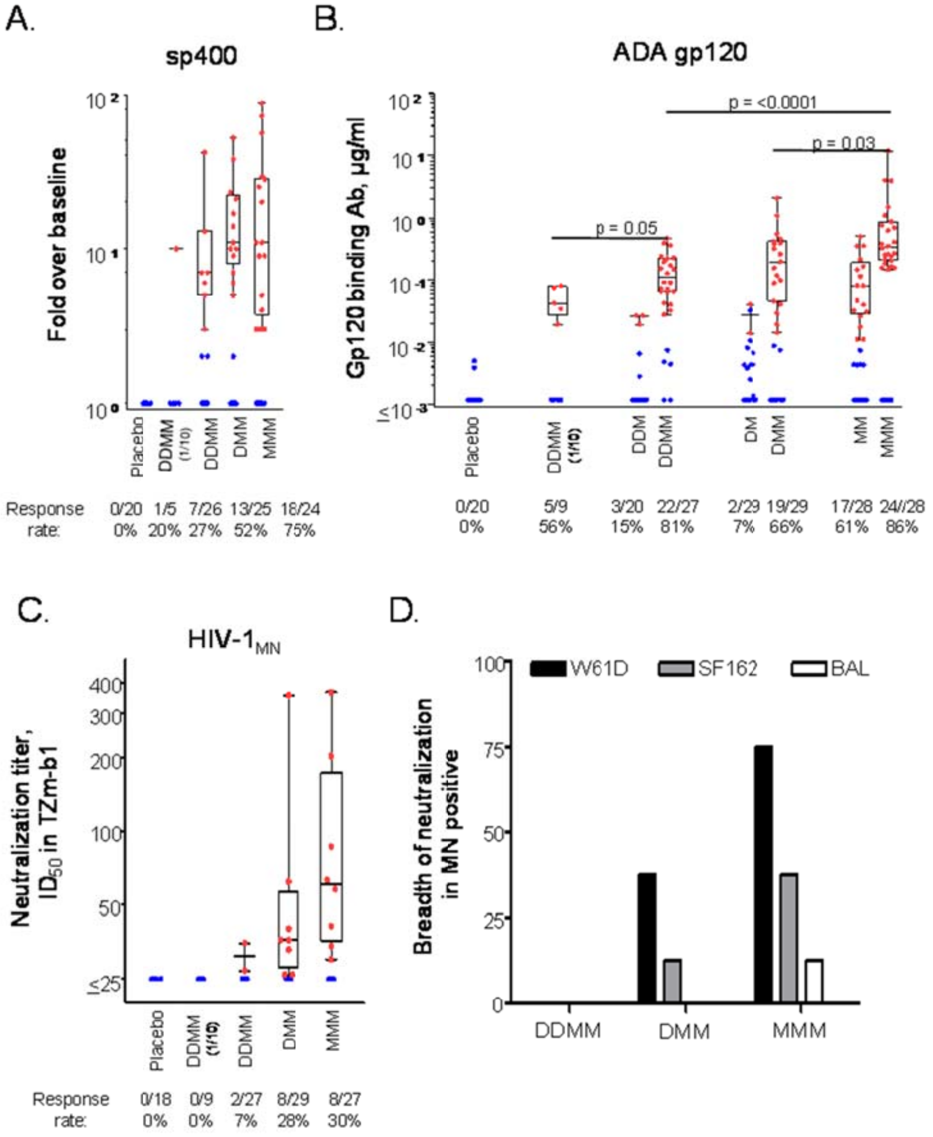

Figure 4-13 Magnitudes and response rates of Env binding and neutralizing antibodies. (A) Binding Ab for sp400, a peptide representing the immunodominant region of gp41. (B) Binding Ab for ADA gp120. (C) Neutralizing Ab for HIV-1_{MN}. Designations below schematics indicate groups and response rates (see legend to Figure 4-11 for details). The boxplots show median and 25th and 75th percentiles for positive data (indicated by red points). Blue points indicate negative data. Data for determining P values include only positive data. (D) Percent of positive MN neutralization responses also neutralizing other tier 1 isolates. Seventeen of the samples demonstrating neutralization against HIV-1_{MN} were evaluated further. The tier 1 isolates are shown including HIV-1_{SF162}, HIV-1_{W61D} (T-cell lab-adapted strain), and HIV-$1_{BAL.}$

The affinity maturation of the vaccine-elicited antibody responses was measured by an avidity index assay (Binding Antibody Multiplex Assay- Avidity Index (BAMA- AI). The binding antibody multiplex assay has been previously described [58]. Avidity index was measured with the BAMA assay with the inclusion of a denaturation step (treating the samples with 0.1 M Na-citrate, pH 3.0) and comparing the mean fluorescence intensity (MFI) in the treated well vs. MFI in the untreated well. Analysis of Avidity Index at Visit 12 indicated that DMM and MMM vaccine schedules elicited antibodies

with a higher avidity index than either the DDMM (low) or DDMM (high) responses. No significant difference in antibody avidity index was seen between the DMM and MMM regimens. Furthermore, antibody avidity remained durable in all vaccine groups through Visit 14 (6 months post boost) [59].

4.9.2 Phase 2a, HVTN 205

Based on the results of HVTN 065, the full dose DDMM regimen and subsequently the MMM regimen were chosen to move forward into a phase 2a study (HVTN 205), which is currently near completion (Table 4-5). The study originally included one vaccine arm (DDMM regimen) to assess the safety and tolerability of the prime-boost regimen of JS7/MVA62B in 225 healthy, uninfected volunteers, aged 18 to 50 years (150 vaccine recipients, 75 placebo recipients). Due to the attractiveness of a homologous prime/boost regimen and the generation of higher antibody responses in the MMM arm in HVTN 065, the HVTN 205 protocol was amended to provide an additional arm of 75 healthy, HIV-1--uninfected subjects who received three full doses of MVA62B (MMM regimen) on a 0, 8 and 24 week schedule. The primary objective was to assess the safety and tolerability of the prime-boost regimen of JS7 DNA and MVA62B (DDMM) vaccines and the regimen of MVA62B alone (MMM). The secondary objectives was to obtain more immunogenicity data, specifically, the CD4+ and CD8+ T cell and antibody response rates, for the two regimens.

All immunizations are now completed for HVTN 205. Overall, the vaccines have been well tolerated. Most reactogenicity symptoms are mild. In part A, there was one severe local reactogenicity symptom each of pain and tenderness to MVA or placebo. Also for the systemic reactogenicity symptoms, there was one event of severe malaise to DNA or placebo and one event of severe malaise to MVA or placebo. In Part B, thus far, there were two severe reactogenicity symptom of tenderness to DNA or MVA or placebo, one severe reactogenicity symptom of pain to MVA or placebo, one participant with severe increased temperature to MVA or placebo, and one participant has had a severe allergic reaction to one of the study products (DNA or MVA or placebo). Two grade 3 lab abnormalities deemed probably not related to study product include transient decreased lymphocyte count and hypoglycemia. There have been no other grade 3 or higher related AE’s, no related SAEs, and no cardiac events, including myopericarditis related to study product.

Table 4-5 HVTN 205 schema

		Dose		Vaccination Schedule in months (days)			
Group	Number	DNA (mg)	MVA ($TCID_{50}$)	0 (0)	2 (56)	4 (112)	6 (168)
Part A							
Group 1	120	3	$1x10^8$	DNA	DNA	MVA	MVA
Group 2	60	0	0	Placebo	Placebo	Placebo	Placebo
Part B							
Group 3	30	3	$1x10^8$	DNA	DNA	MVA	MVA
Group 4	75	0	$1x10^8$	MVA	MVA	Placebo	MVA
Group 5	15	0	0	Placebo	Placebo	Placebo	Placebo

Part A: Total 180 = 120 vaccinees + 60 placebos
Part B: Total 120 = 105 vaccinees + 15 placebos
Total for study: 300 = 225 vaccinees + 75 placebos

4.9.3 Prior experience with GM-CSF in humans

Human GM-CSF (Leukine® – trade name) delivered as a recombinant protein, has been approved by the FDA for the following human uses:

- To stimulate production of white blood cells after autologous bone marrow transplantation in patients with non-Hodgkin's lymphoma, acute lymphocytic leukemia, or Hodgkin's disease (March, 1991)

- To treat fungal infections and for the acceleration of white blood cells following chemotherapy (November, 1996)

4.9.4 Vaccine trials incorporating GM-CSF as an adjuvant

At least 6 cancer vaccine studies have used cells expressing GM-CSF [60-65] and at least one malaria vaccine study [66] has used DNA encoding GM-CSF as an adjuvant. In the cancer vaccine studies, the secreted amount of expressed GM-CSF protein has ranged from 20 to 1400 ng/10^6 cells/24 hours. The estimated 250 ng/10^6 cells/48 hours of GM-CSF expressed by 293T cells transiently transfected with the GEO-D03 DNA plasmid is midway in this range. In six published studies in which GM-CSF-expressing tumor cells were administered and toxicities were described, the majority of toxicities were Grade 1 or 2 injection site reactions, such as erythema, induration, and pruritis. Subjects on only one study experienced systemic toxicities such as fatigue or flu-like symptoms [65]. The one report for a GM-CSF encoded DNA plasmid vaccine study did not include toxicities [66]. GeoVax expects the local effects seen with ID and subcutaneous (SC) administration in the cancer vaccine studies may be diminished somewhat in the proposed study by the use of a DNA vaccine and the IM route of injection.

Other vaccine studies have delivered recombinant GM-CSF as a protein administered ID or SC for cancer or infectious disease indications [67]. These studies have included doses up to 500 mcg per dose. Provenge®, an approved cancer immunotherapy, uses GM-CSF

incorporated in a fusion protein *ex vivo* to stimulate autologous T cells to treat prostate cancer [68,69].

4.10 Potential risks of study products and administration

4.10.1 Anti-GM-CSF binding antibodies

Anti-GM-CSF antibodies are known to be present in healthy people, however at significantly lower levels than persons with clinical diseases related to GM-CSF autoantibody production, which is manifested as pulmonary alveolar proteinosis (PAP) [70]. Levels found in most healthy individuals are typically 1mcg/ml. The lower threshold for levels associated with PAP is 10.4 – 19 mcg/ml; although the average levels for patients with PAP were nearly 60 mcg/mL [70]. Of patients who received Leukine® 250ug 3 times per week IV or SC for 28 to 84 days in multiple courses, anti-GM-CSF autoantibodies were found in 5/214 patients and none were symptomatic (Leukine® prescribing information). The exposure to externally derived GM-CSF from the GEO-D03 vaccine will be orders of magnitude lower than the doses of Leukine® given to clinical patients. Therefore, there is no expectation that GEO-D03 would elicit clinically significant anti-GM-CSF autoantibodies. Anti-GM-CSF binding antibodies will be measured in this study at baseline, and after each dose of GEO-D03 to evaluate whether there is any effect of this vaccine on GM-CSF autoantibodies. The PSRT will be monitoring the AE reports throughout the study for any signs or symptoms of PAP that may require further evaluation. The batched anti-GM-CSF Ab reports will also be monitored by the PSRT for potentially concerning patterns or unusual values to determine if changes to the study protocol or unblinding for further evaluation is warranted.

4.10.2 GM-CSF levels

GM-CSF in very low levels is nearly ubiquitous. Very high levels have been associated with specific autoimmune and inflammatory diseases, and deficiencies in GM-CSF production have been associated with morbidity and mortality from microbial infections and PAP. The GEO-D03 vaccine given in this study is not expected to increase the total GM-CSF levels above baseline but will be measured after GEO-D03 administration for informative purposes.

Table 4-6 Summary of potential risks of study products and administration

Common	• Mild to moderate injection site pain, tenderness, erythema, or swelling/induration/edema<br>• Malaise/fatigue, myalgia, or headache in the first few days following injection<br>• A vaccine-induced positive HIV antibody test result<br>• Axillary or cervical lymph node swelling, pain, or tenderness
Less common	• Severe injection site pain or tenderness<br>• Other mild to moderate injection site reactions (local paresthesia, pruritis, warmth or burning sensation)<br>• Fever, chills, flu-like syndrome, arthralgia, rash, nausea, vomiting, or dizziness in the first few days following injection<br>• Vasovagal reaction/lightheadedness/dizziness related to the injection procedure<br>• Transient changes in clinical laboratory values<br>• Injection site hematoma, bruising/ecchymosis, laceration, other transient lesions, or bleeding related to the injection procedure
Uncommon or rare	• Severe localized injection site reaction, such as sterile abscess or secondary bacterial infection<br>• Allergic reaction, including rash, urticaria, angioedema, bronchospasm, or anaphylaxis<br>• Prolonged QT interval<br>• Dyspnea<br>• Decreased neutrophil or hemoglobin count
Unknown frequency or theoretical risks	• Muscle damage at the injection site<br>• Myo/pericarditis<br>• Autoimmune disease or cancer<br>• Effects on a participant's response to an approved HIV vaccine administered in the future<br>• Effects on susceptibility to HIV, if the participant is exposed to HIV<br>• Effects on the course of HIV infection/disease, if the participant is infected with HIV<br>• Effects on the fetus and on pregnancy<br>• Elevated CPK<br>• Pulmonary alveolar proteinosis

5 Objectives and endpoints

5.1 Primary objectives and endpoints

Primary objective:

- To assess the safety and tolerability of a heterologous prime-boost regimen consisting of two injections of GEO-D03 DNA vaccine and two or three injections of modified vaccinia Ankara (MVA)/HIV62B (MVA62B) with dose escalation of the GEO-D03 DNA vaccine from 0.3 mg to 3 mg

Primary endpoints:

- Frequency and severity of local injection site reactogenicity signs and symptoms: pain, tenderness, erythema, induration, and maximum severity of pain and/or tenderness; frequency and severity of systemic reactogenicity signs and symptoms: fever, malaise/fatigue, myalgia, headache, nausea, vomiting, chills, arthralgia, and maximum severity of systemic symptoms within the initial 72 hour period following vaccination

- Distribution of values of safety laboratory measures: CBC, ALT, AST, alkaline phosphatase, creatinine

- Frequency of AEs categorized by MedDRA System Organ Class,and MedDRA Preferred Term, severity and assessed relationship to study products; detailed description of all AE meeting DAIDS criteria for expedited reporting

- Report of the number of participants with early discontinuation of vaccinations, by treatment group and reason for discontinuation

5.2 Secondary objectives and endpoints

Secondary objective 1:

- To compare the Env-specific antibody responses after 3 doses of MVA in Group 2 with those after 2 doses of MVA in Group 3 at 2 weeks after the last vaccination

Secondary endpoints 1:

- Frequency and magnitude of HIV-1 Env-specific binding antibody and isotypes

- Avidity indices for Env-specific binding antibody

- Frequency of neutralizing antibody responses to HIV-1

- In individuals with neutralizing antibodies to HIV-1, neutralizing antibody titers (magnitude) and breadth of neutralizing activity

Secondary objective 2:

- To compare the Env-specific antibody responses two weeks after 2 doses of MVA spaced 2 months apart in Group 2 to those after 2 doses of MVA spaced 4 months apart in Group 3

Secondary endpoints 2:

- Endpoints as indicated in secondary endpoints 1

Secondary objective 3:

- To compare the elicitation of Env-specific antibody responses 2 weeks after the second and third MVA boosts in Group 2

Secondary endpoints 3:

- Endpoints as indicated in secondary endpoints 1

Secondary objective 4:

- To compare the elicitation of HIV-specific T cells between Groups 2 and 3

Secondary endpoints 4:

- Frequency and magnitude of HIV-1 specific CD4+ and CD8+ T cell responses as measured by ICS at 2 weeks after the second vaccination for Groups 2 and 3
- Frequency and magnitude of HIV-1 specific CD4+ and CD8+ T cell responses as measured by ICS at 2 weeks after the last vaccination for Groups 2 and 3

Secondary objective 5

- To compare the cytokine profile elicited by the vaccine regimen for Groups 2 and 3

Secondary endpoint 5

- Determine concentrations of secreted cytokines and chemokines by multiplex analysis of stimulated cell supernatants following peptide stimulation of PBMC 2 weeks after the last vaccination for Groups 2 and 3

Secondary objective 6

- To characterize innate immune responses in treatment Groups 2 and 3

Secondary endpoints 6

- Blood concentrations of lymphocyte populations (T, B and NK cells), dendritic cells, monocytes and granulocytes
- Concentrations of cytokines and chemokines in serum and/or plasma samples

5.3 Exploratory objectives

Exploratory objective 1:

- To measure the elicitation of humoral and cellular immune responses of Group 1 and compare responses between the active arms of treatment Groups 1, 2, and 3 at two weeks after the final MVA dose and potentially compare responses between the active arms of treatment Groups 1, 2, and 3 at other timepoints

Exploratory endpoints 1:

- Frequency and magnitude of HIV-1 Env-specific binding antibody and isotypes
- Frequency of neutralizing antibody responses to HIV-
- In individuals with neutralizing antibodies to HIV-1, neutralizing antibody titers (magnitude) and breadth of neutralizing
- Frequency and magnitude of HIV-1 specific CD4+ and CD8+ T cell responses

Exploratory objective 2:

- To further characterize B cell immune responses

Exploratory endpoint 2:

- Titers of ADCC antibodies

Exploratory objective 3:

- To further characterize T-cell immune responses

Exploratory endpoints 3:

- Determine concentrations of secreted cytokines and chemokines by multiplex analysis of stimulated cell supernatants following peptide stimulation of PBMC
- Assess the functional and phenotypic profiles of antigen specific CD4+ and CD8+ T-cell responses by multiparameter polyfunctional flow cytometry

Exploratory objective 4:

- To further characterize innate immune responses in treatment Groups 2 and 3

Exploratory endpoint 4:

- Changes in PBMC gene expression relative to prevaccine levels of key genes expected to change

Exploratory objective 5:

- To measure GM-CSF and GM-CSF specific antibodies for Groups 1, 2, and 3

Exploratory endpoints 5:

- Serum concentrations of GM-CSF after DNA vaccination
- Serum concentrations of GM-CSF specific antibody after DNA vaccination

6 Statistical considerations

6.1 Accrual and sample size calculations

Recruitment will target 48 healthy, HIV-uninfected vaccinia-naïve adult participants.

Groups 1 and 2 were enrolled sequentially according to dose escalation rules outlined in the protocol. All enrollments and safety reviews for dose escalation occurred during Version 1.0 of the protocol. Participants were randomized within each group to receive either DNA prime or placebo followed by MVA boost or placebo in a vaccine:placebo ratio of 10:2 in Group 1 and 30:6 in Group 2.

In order to accommodate the splitting of the original Group 2 from Version 1.0 into the new Groups 2 and 3 of Version 2.0, the first 18 participants (who were originally randomized into blocks of 5:1 active to placebo) enrolled into the original Group 2 will follow the new Version 2.0 Group 2 schedule. The last 18 participants enrolled into the original Group 2 will follow the new Version 2.0 Group 3 schedule. This will ensure the integrity of randomization and blinding within each group.

Since enrollment is concurrent with receiving the first study vaccination, all participants will provide some safety data. Hence, sample size calculations for safety in Section 6.1.1 are based on the target sample sizes. It is possible, however, for immunogenicity data to be missing; previous HVTN and AIDS Vaccine Evaluation Group (AVEG) studies suggest 10% is a reasonable estimate for the rate of missing data. For this reason, the sample size calculations in Section 6.1.2 account for 10% of enrolled participants having missing data for the primary immunogenicity endpoints.

6.1.1 Sample size calculations for safety

The goal of the safety evaluation for this study is to identify safety concerns associated with product administration. The ability of the study to detect serious adverse events (SAEs) can be expressed by the true event rate above which at least 1 SAE would likely be observed and the true event rate below which no events would likely be observed. Specifically, for the vaccine arm in Group 1 (n =10), there is a 90% chance of observing at least 1 event if the true rate of such an event is 21% or more; and there is a 90% chance of observing no events if the true rate is 1% or less. For the vaccine arm in Groups 2 and 3 (n = 15), there is a 90% chance of observing at least 1 event if the true rate of such an event is 14.2% or more; and there is a 90% chance of observing no events if the true rate is 0.7% or less. For the 3.0mg GEOD03 DNA vaccine, the data from Groups 2 and 3 could be pooled (n = 30) such that there is a 90% chance of observing at least 1 event if the true rate of such an event is 7% or more; and there is a 90% chance of observing no events if the true rate is 0.35% or less. For the combined 3.0mg GEOD03 DNA plus MVA2B vaccines, the data could either be pooled as above or analyzed separately by group. In the latter case (n=15), there is a 90% chance of observing at least 1 event if the true rate of such an event is 14.2% or more; and there is a 90% chance of observing no events if the true rate is 0.7% or less. In HVTN vaccine trials from December 2000 through September 2010, about 4% of participants who received placebos experienced an SAE.

Probabilities of observing 0, 1 or more, and 2 or more events among arms of size 10, 15, and 30 are presented in Table 6-1 for a range of possible true adverse event rates. These calculations provide a more complete picture of the sensitivity of this study design to identify potential safety problems with the vaccine.

Table 6-1 Probability of observing 0 events, 1 or more events, and 2 or more events, among arms of size 10, 15, and 30, for different true event rates

Event rate (%)	Pr (0/10)	Pr (1+/10)	Pr (2+/10)	Pr (0/15)	Pr (1+/15)	Pr (2+/15)	Pr (0/30)	Pr (1+/30)	Pr (2+/30)
1.0	90.4	9.6	0.4	86.0	14.0	1.0	74.0	26.0	3.6
3.5	70 .0	30.0	4.6	58.6	41.4	9.5	34.3	65.7	28.3
5.0	59.9	40.1	8.6	46.3	53.7	17.1	21.5	78.5	44.6
10.0	34.9	65.1	26.4	20.6	79.4	45.1	4.2	95.8	81.6
20.0	10.7	89.3	62.4	3.5	96.5	83.3	0.1	99.9	98.9
30.0	2.8	97.2	85.1	0.5	99.5	96.5	<0.1	>99.9	>99.9
40.0	0.6	99.4	95.4	<0.1	>99.9	99.5	<0.1	>99.9	>99.9
50.0	<0.1	>99.9	98.9	<0.1	>99.9	>99.9	<0.1	>99.9	>99.9
60.0	<0.1	>99.9	99.8	<0.1	>99.9	>99.9	<0.1	>99.9	>99.9

An alternative way of describing the statistical properties of the study design is in terms of the 95% confidence interval for the true rate of an adverse event based on the observed data. Table 6-2 shows the 2-sided 95% confidence intervals for the probability of an event based on a particular observed rate. Calculations are done using the score test method [71]. If none of the 40 participants receiving the vaccine regimen in any arm of any group experience a safety event, the 95% 2-sided upper confidence bound for the true rate of such events in the total vaccinated population is 9%. For the vaccine arm of size 15, if no participants experiences a safety event, the 95% 2-sided upper confidence bound for the rate of such events overall is 20%. For the vaccine arm of size 10, the 2-sided upper confidence bound for this rate is 28%.

Table 6-2 Two-sided 95% confidence intervals based on observing a particular rate of safety endpoints for arms of size 10, 15, 30, and 40

Observed proportion	95% CI
0/10	(0.0, 27.8)
1/10	(1.8, 40.4)
2/10	(5.7, 51.0)
0/15	(0.0, 20.4)
1/15	(1.2, 29.8)
2/15	(3.7, 37.9)
0/30	(0.0, 11.4)
1/30	(0.6, 16.7)
2/30	(1.8, 21.3)
0/40	(0.0, 8.8)
1/40	(0.4, 12.9)
2/40	(1.4, 16.5)

6.1.2 Sample size calculations for immunogenicity

The main goals of this trial regarding immunogenicity outcomes are to assess whether, and the degree to which, immune responses in vaccinated subjects are different following 1) Second MVA boost in Group 2 vs. third MVA boost in Group 2; 2) second MVA boost in Group 2 vs. second MVA boost in Group 3; and 3) third MVA boost in Group 2 vs. second MVA boost in Group 3. The first objective assesses change in response as a result of an additional vaccination from 2 to 3 MVA boosts, the second assesses change in response for a 2 vs. a 4 month interval between the first and second MVA boosts, and the third assesses change in response for the 2 MVA boosts with a 4 month interval vs. 3 MVA boosts with a 4 month interval prior to last vaccination. The immunogenicity measurements of interest are binding antibody, binding avidity, neutralizing antibody, and intracellular cytokine staining assays among vaccinees. No adjustment for multiple comparisons will be made for the use of multiple assays or multiple objectives. For binary immune response endpoints such as status of MN neutralization, McNemar's test will be used to assess an increase in the positive response rate for comparisons within an individual (second MVA vs. third MVA in Group 2). Figure 6-1 shows statistical power for comparing various differences in response rates at the two time-points in Group 2 for a sample size of 13, assuming a 10% loss to follow-up. The figure assumes that individuals who respond at the second MVA will also respond at the third MVA, such that the only change in response is due to an increase in the number of individuals who respond at the second time point. For example, if we expect a 7% MN neutralization response rate after the second MVA similar to HVTN 065 Group 2, then there is 81% power to detect a 47% additive elevation in the response rate (e.g., from 7% to 54%). In addition, if we expect a 27% binding antibody response rate after the second MVA, then there is 81% power to detect a 47% additive elevation in the response rate (e.g., from 27% to 74%).

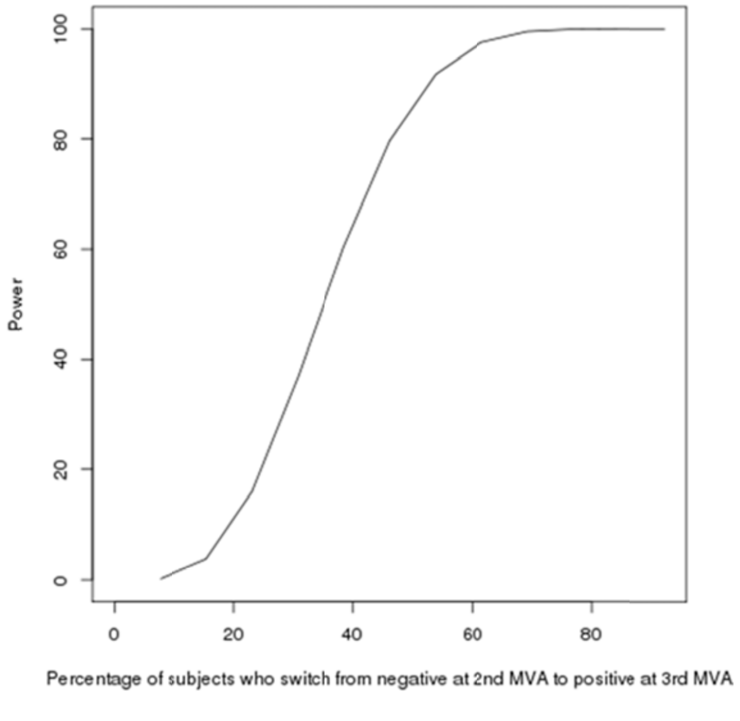

Figure 6-1 Power to detect a non-zero difference in response rates by one-sided unconditional McNemar's test at the 0.025 level following 2 and 3 MVA boosts in Group 2 vaccinees (n=13)

For comparisons between groups of individuals (i.e., second MVA in Group 2 vs. second MVA in Group 3 or third MVA in Group 2 vs. second MVA in Group 3), Fisher's exact tests will be used to assess differences in the response rates. Table 6-3 illustrates the minimum response rates that could be detected between vaccine groups (n=13) with statistical power of 80% and 90% for an exact 2-sided test with a Type I error rate of 0.05.

Table 6-3 Minimum response rates required to rule out a non-zero difference between 2 groups of size 13

	Minimum detectable response rate second group (%)	
True response rate first group (%)	80% power	90% power
10	65	72
20	76	82
30	85	91
40	93	97
50	99	>99

For subjects with a positive response for a given assay, various quantitative readouts will be assessed; for example for MN neutralization, these readouts would include MN 50% inhibition neutralization titer and magnitude-and-breadth of neutralization to panels of Tier 1 and Tier 2 isolates, summarized as areas under the magnitude-breadth curve. Similarly, for subjects with a positive binding response, various quantitative readouts will be assessed, such as binding titer, avidity level, and the magnitude-and-breadth of binding to panels of Tier 1 and Tier 2 isolates, summarized as areas under the magnitude-breadth curve.

For quantitative readouts where the comparison is between vaccinations in Group 2, an exact Wilcoxon signed-rank test will be used for assessing whether the location centers of the readouts increase from after the second boost to after the third boost, in the subset of Group 2 vaccinated subjects with a positive response.

For the scenario where the true positive response rate for vaccine recipients is 50% after the second boost and 90% after the third boost, Figure 6-2 shows statistical power for comparing various mean response levels, where we set the standard deviation (SD) of the readouts at each time-point to 1, such that the results are interpretable in terms of power to detect a one SD increase in the mean readout. The calculations are performed by simulation assuming a 90% probability of response after the third MVA vaccination among those with a response after the second MVA vaccination, and the readouts were generated from a bivariate normal distribution with linear correlation of 0.7. These power calculations are based on 10,000 simulated data-sets, where, for each simulated trial, an exact Wilcoxon signed-rank test is applied to the subgroup of subjects with a positive response after the second MVA vaccination. Figure 6-2 shows that there is at most 60% power to detect a 3-sd increase in mean response magnitude after the third MVA vaccination under this scenario.

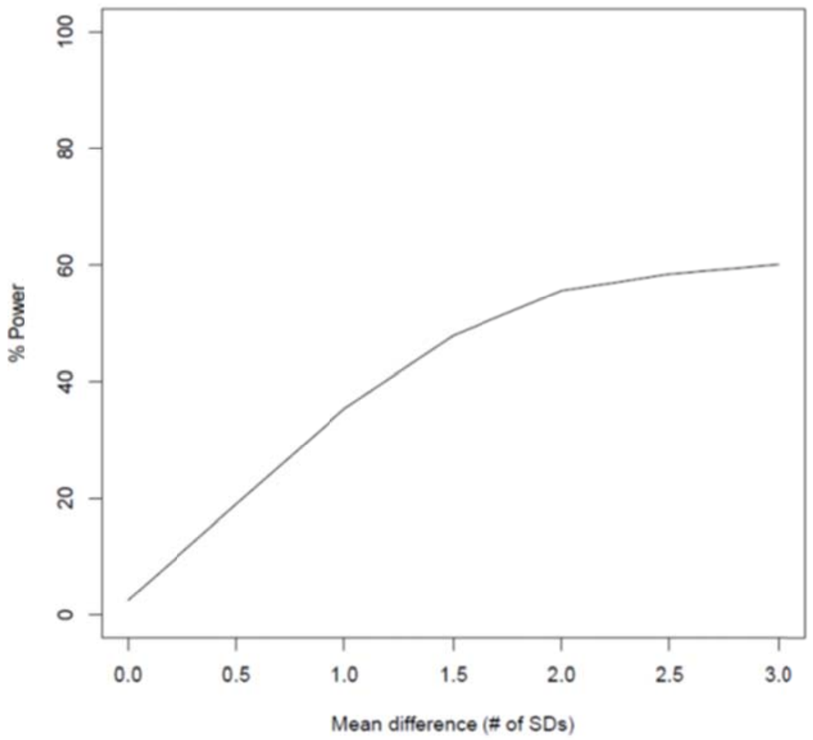

Figure 6-2 Power to detect various differences in mean magnitudes between a second MVA response of 50% and a third MVA response of 90% with a 90% probability of response at the third MVA given a response at the second MVA and 70% correlation between magnitudes

For quantitative readouts where the comparison is between groups, an exact Wilcoxon rank sum/Mann Whitney test will be used for assessing whether the location centers of the readouts are different. Figure 6-3 shows the power for detecting a difference in magnitude for assays (i.e., binding antibody) where the majority of participants have a detectable response to the vaccine. Ten thousand trials were simulated, each with size 13 per group, where the data had a normal distribution with a common standard deviation. The mean of 3.74 and standard deviation of 0.48 were chosen to be equal to the observed log-transformed net fluorescence intensity mean and SD for IgG binding antibody responses to ConS in participants receiving a DNA-prime, MVA-boost in HVTN205. Power was based on rejecting the null hypothesis of no difference for a 2-sided Mann-Whitney test with an alpha level of 0.05. The geometric mean ratio and difference of the observed MVA alone to DNA-MVA means for the IgG ConS in HVTN 205 was 1.07 and 0.27, respectively, which would not be rejected in this scenario. A difference of at least 4 fold would be required in order to have 80% power to reject the null of no difference.

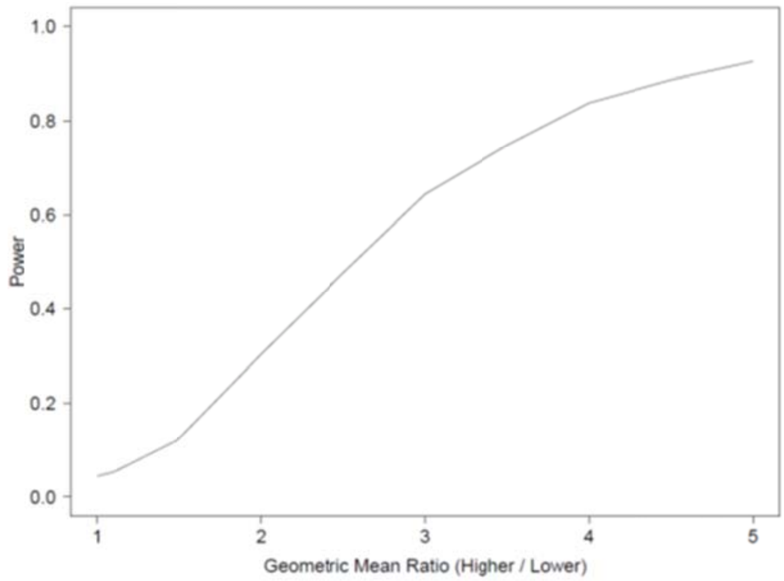

Figure 6-3 Power to detect a non-zero difference in binding antibody magnitude between groups by two-sided Mann-Whitney test at the 0.05 level (n=13 per group)

Another way of choosing between regimens is to select the regimen with the higher estimated mean or median. Figure 6-4 shows the probability of observing a higher estimated mean given that the true mean is higher for assays (i.e., binding antibody) where the majority of participants have a detectable response to the vaccine. Data were simulated as described above, where power was calculated as the probability of correctly selecting the regimen with a higher true mean. The geometric mean ratio of the observed MVA alone to DNA-MVA for the IgG Con S in VTN 205 was 1.07, which corresponds to a 60% probability that the MVA alone would have a higher estimated mean if that were the true ratio of the regimens. A difference of about 1.8 fold would be required in order to have 90% probability of observing a higher estimated mean if the mean were truly higher.

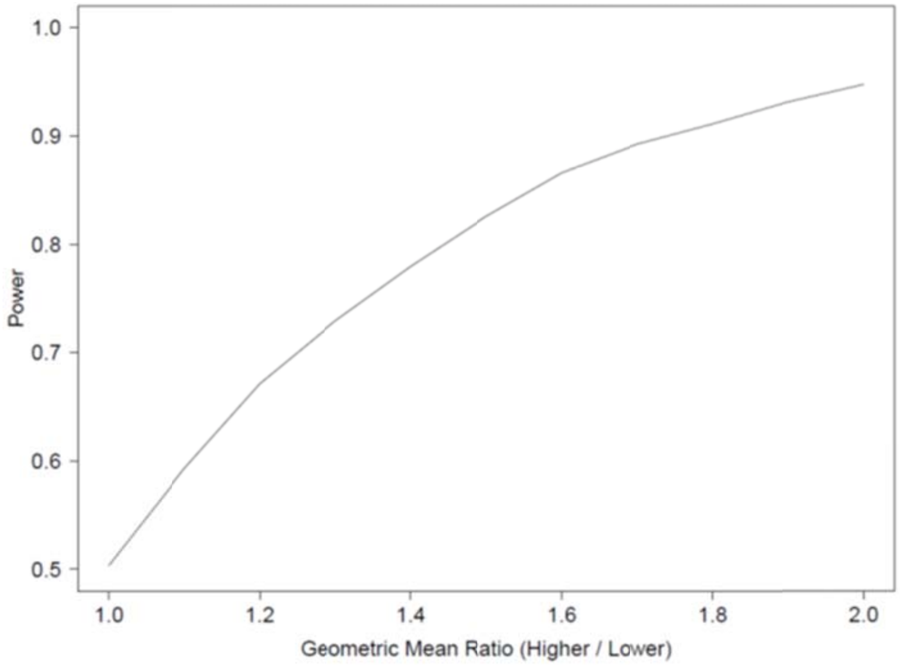

Figure 6-4 Probability of observing a higher estimated mean binding antibody response if the mean is truly higher (n=13 per group)

Figure 6-5 shows the power for detecting a difference in magnitude for assays (ie, avidity) where a proportion of participants have a detectable response to the vaccine. Data was simulated for 10,000 trials each of size 13 per group where the data had a mixed binary and normal distribution with a common standard deviation. The response rate of 61%, mean among positive responders of 53, and standard deviation among positive responders of 20 was chosen to be equal to the observed avidity index values for the IgG binding avidity responses to the immunodominant gp41 peptide in participants receiving a DNA-prime, MVA-boost in VTN205. Power was calculated for rejecting the null hypothesis of no difference using a 2-sided Mann-Whitney test with an alpha level of 0.05. The MVA alone group in VTN205 had approximately a 10% higher response rate and a 6 unit increase in magnitude over the DNA-prime group, which would not be rejected in this scenario. A 30% increase in response rate (i.e., all participants have measurable avidity to the immunodominant peptide) with an avidity increase of at least 20 to 30 units would be required in order to have 80-90% power to reject the null hypothesis of no difference.

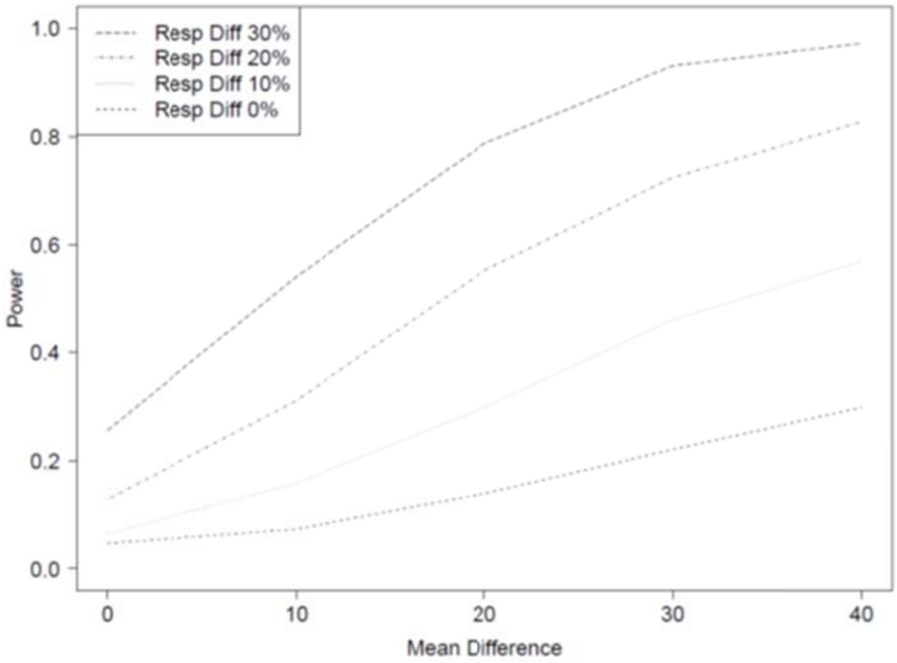

Figure 6-5 Power to detect a non-zero difference in avidity magnitude between groups by two-sided Mann-Whitney at the 0.05 level (n=13 per group)

Figure 6-6 shows the probability of observing a higher estimated mean given that the mean is truly higher for assays (i.e., avidity) where a proportion of participants have a detectable response to the vaccine. Data were simulated as described above, where power was calculated as the probability of correctly selecting the regimen with a higher true mean. The MVA alone group in VTN205 had approximately a 10% higher response rate and a 6 unit increase in magnitude over the DNA-prime group, so if we assume these are the true values of the distribution, then there is approximately a 73% probability of this regimen having a higher observed mean in a trial of size 13 per group.

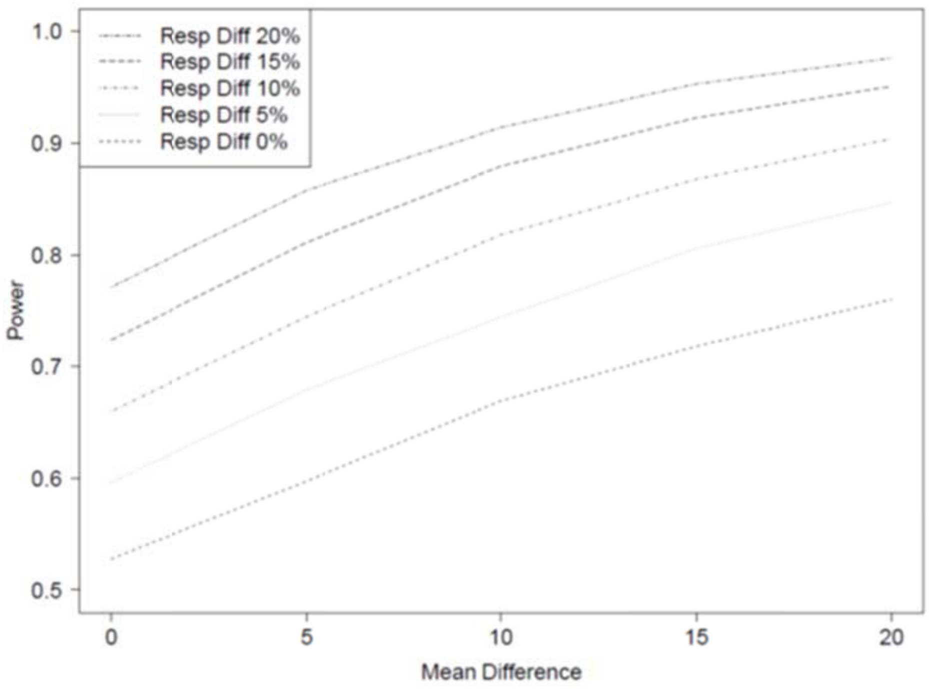

Figure 6-6 Probability of observing a higher estimated mean avidity response if the mean is truly higher (n=13 per group)

6.2 Randomization

The randomization sequence will be obtained by computer-generated random numbers and provided to each HVTN CRS through the SDMC's Web-based randomization system. The randomization will be done in blocks to ensure balance across arms. At each institution, the pharmacist with primary responsibility for dispensing study products is charged with maintaining security of the treatment assignments.

The SDMC will send to the Pharmacist of Record information and instructions regarding the modified treatment assignment to participants of Group 2 (version 2) and Group 3, at their respective CRS.

6.3 Blinding

Participants and site staff (except for site pharmacists) will be blinded as to participant treatment arm assignments (eg, vaccine or placebo). Study product assignments are accessible to those HVTN CRS pharmacists, DAIDS protocol pharmacists and contract monitors, and SDMC staff who are required to know this information in order to ensure proper trial conduct. Any discussion of study product assignment between pharmacy staff and any other HVTN CRS staff is prohibited. The HVTN SMB members also are unblinded to treatment assignment in order to conduct review of trial safety.

When a participant leaves the trial prior to study completion, the participant will be told he or she must wait until all participants are unblinded to learn his or her treatment assignment.

Emergency unblinding decisions will be made by the site investigator. If time permits, the HVTN 094 PSRT should be consulted before emergency unblinding occurs.

6.4 Statistical analysis

This section describes the final study analysis, unblinded as to treatment arm assignment. All data from enrolled participants will be analyzed according to the initial randomization assignment regardless of how many vaccinations they received. The analysis is a modified intent-to-treat analysis in that individuals who are found to have been HIV infected at enrollment or who are randomized but not enrolled do not contribute data and hence are excluded. Because of blinding and the brief length of time between randomization and enrollment—typically no more than 4 working days—very few such individuals are expected.

Analyses for primary endpoints will be performed using SAS and R. All other descriptive and inferential statistical analyses will be performed using SAS, StatXact, or R statistical software.

No formal multiple comparison adjustments will be employed for multiple safety endpoints, multiple primary immunogenicity endpoints, or secondary endpoints. However, multiplicity adjustments will be made for certain immunogenicity assays, as discussed below, when the assay endpoint is viewed as a collection of hypotheses (eg, testing multiple peptide pools to determine a positive response).

6.4.1 Analysis variables

The analysis variables consist of baseline participant characteristic, safety, and immunogenicity for primary- and secondary-objective analyses.

6.4.2 Baseline comparability

Treatment arms will be compared for baseline participant characteristics using descriptive statistics.

6.4.3 Safety/tolerability analysis

Since enrollment is concurrent with receiving the first vaccination, all participants will have received at least 1 vaccination and therefore will provide some safety data.

6.4.3.1 Reactogenicity

The number and percentage of participants experiencing each type of reactogenicity sign or symptom will be tabulated by severity and treatment arm and the percentages displayed graphically by arm. For a given sign or symptom, each participant's reactogenicity will be counted once at the maximum severity for all injection visits. In addition, to the individual types of events, the maximum severity of local pain or tenderness, induration or erythema, and of systemic symptoms will be calculated. Kruskal-Wallis tests will be used to test for differences in severity between arms.

6.4.3.2 AEs and SAEs

AEs will be summarized using MedDRA System Organ Class and Preferred Terms. Tables will show by treatment arm the number and percentage of participants

experiencing an AE within a System Organ Class and Preferred Term by severity or by relationship to study product. For the calculations in these tables, a participant with multiple AEs within a category will be counted once at the maximum severity or the strongest recorded causal relationship to study product. Formal statistical testing comparing arms is not planned since interpretation of differences must rely heavily upon clinical judgment.

A listing of SAEs reported to the DAIDS Regulatory Support Center (RSC) Safety Office will provide details of the events including severity, relationship to study product, time between onset and last vaccination, and number of vaccinations received. A similar listing will be provided on an annual basis for autoimmune disorders, which are AEs of special interest (AESI). A sample listing of AESI is provided in Appendix O.

6.4.3.3 Local laboratory values

Boxplots of local laboratory values will be generated for baseline values and for values measured during the course of the study by treatment arm and visit. Each boxplot will show the first quartile, the median, and the third quartile. Outliers (values outside the boxplot) will also be plotted. If appropriate, horizontal lines representing boundaries for abnormal values will be plotted.

6.4.3.4 Reasons for vaccination discontinuation and early study termination

The number and percentage of participants who discontinue vaccination and who terminate the study early will be tabulated by reason and treatment arm.

6.4.4 Immunogenicity analysis

6.4.4.1 General approach

For the statistical analysis of immunogenicity endpoints, data from enrolled participants will be used according to the initial randomization assignment regardless of how many injections they received. Additional analyses may be performed, limited to participants who received all scheduled injections per protocol. Assay results that are unreliable, from specimens collected outside of the visit window, or from HIV-infected participants postinfection are excluded. Since the exact date of HIV infection is unknown, any assay data from blood draws 4 weeks prior to an infected participant's last seronegative sample and thereafter may be excluded. If an HIV-infected participant does not have a seronegative sample postenrollment, then all data from that participant may be excluded from the analysis.

Qualitative assay data (eg, response rates) will be analyzed by tabulating the frequency of positive response for each assay by antigen and treatment arm at each timepoint for which an assessment is performed. Crude response rates will be presented with their corresponding 95% confidence interval estimates calculated using the score test method.[71] Because of the small numbers of placebo participants in each group, no adjustment will be made to the vaccine arm estimates for the false positive rates in the placebo arms. Fisher's exact tests will be used to compare response rates between the vaccine arms of Groups 2 and 3, with a significant difference declared if the 2-sided p-value is ≤ 0.05. The key time-points for these assessments are two weeks after the third MVA vaccination and/or two weeks after the second MVA vaccination.

In addition to assessing response rates at each time-point, the difference between response rates after the second and third MVA doses in Group 2 will be compared using

McNemar's test. Moreover, the maximum likelihood estimate and exact 95% confidence interval for the difference in these response rates will be reported.

For continuous assay data (eg, area-under-the-magnitude-breadth curve for neutralizing antibody responses or percentage of positive cells from the ICS assay), graphical and tabular summaries of the distributions by antigen, treatment arm, and time-point will be made. The difference between continuous endpoints after the second and third MVA doses for Group 2 will be evaluated within the subgroup of subjects with a positive response for the endpoint after the second MVA injection. An exact Wilcoxon signed rank test will be used for testing the null hypothesis that the difference in location centers is zero versus the alternative hypothesis that this difference departs from zero. The difference between continuous endpoints between groups will be evaluated using a Mann-Whitney test. The test statistic will be inverted to provide a Hodges-Lehmann point and 95% confidence interval estimate for the difference in location centers.

More sophisticated analyses employing repeated measures methodology (for example, repeated measures analysis of variance [ANOVA] or generalized estimating equations) may be utilized to incorporate immune responses over several timepoints and to test for differences over time. However, inference from such analyses would be limited by the small sample size of this study. All statistical tests will be 2-sided and will be considered statistically significant if $p \leq 0.05$.

Based upon previous AIDS Vaccine Evaluation Group (AVEG) and HVTN trials, missing 10% of immunogenicity results for a specific assay is common due to study participants terminating from the study early, problems in shipping specimens, or low cell viability of processed peripheral blood mononuclear cells (PBMCs). To achieve unbiased statistical estimation and inferences with the planned methods for estimation and testing, missing data need to be missing completely at random (MCAR). MCAR assumes that the probability of an observation being missing does not depend upon the observed responses or upon any unobserved covariates but may depend upon covariates included in the model (eg, missing more among whites than non-whites). When missing data are minimal (specifically if no more than 20% of participants are missing any values), then the planned procedures are expected to be approximately valid. If not, then the same methods will be used (because the sample sizes are too small to use more sophisticated methods that make a missing at random assumption), and the results will be reported with stated caveats about potential bias due to missing data.

6.4.4.2 Specific approach for the secondary endpoints

Secondary endpoint 1: Env-specific antibody responses as measured by binding and neutralizing antibody assays after second and third MVA boost of Group 2 or between vaccinations for Group 2 vs. Group 3. The analysis of Env-specific antibody response rates as measured by binding and neutralizing antibody assays will be evaluated and compared between the vaccine arms as described for frequency data in the general approach section. A positivity call will be based on a laboratory-specified threshold. The magnitude of responses will also be analyzed as described for the continuous data in the general approach section. Graphs will be used to display the background-subtracted magnitudes for each participant at each timepoint, with a box plot of data from positive responders superimposed on the individual data values. Statistical testing comparing the magnitudes will be based on positive responders only.

If there is sufficient response to a Tier 1 HIV-1 isolate panel, the magnitude-breadth curves of individual participants will be plotted, for each time-point [72]. In that case, for

each of the vaccine arms and time-points the group-averaged magnitude-breadth curves will be computed and included on the plots. At each time-point the individual areas under the magnitude-breadth curves (AUC-MBs) may be compared between the two vaccine arms using the methods for quantitative readouts described in the general approach section. In addition, these AUC-MBs may be compared between the second MVA injection and third MVA injection time-points for Group 2 or between Groups 2 and 3 by the methods described above. For the neutralizing and binding antibody assays, responses to Tier 2 isolate panels may also be measured.

Secondary endpoint 2: Analysis of CD4+ and CD8+ T-cell response as measured by the ICS assay after second and third MVA boosts for Group 2 or between vaccinations for Group 2 vs. Group 3. The analysis of CD4+ and CD8+ T-cell response rates as measured by the ICS assay will be evaluated and compared as described under the general approach. In determining the positivity call for each peptide pool, a multiple comparison adjustment will be made for the two T-cell subsets and the number of peptide pools used in the assay using the discrete Bonferroni adjustment. The magnitude of response will be analyzed as described for continuous data in the general approach section. For each T-cell subset, graphs will be used to display the background-subtracted magnitudes for each participant by protein, treatment arm and time-point, with a box plot of data from positive responders superimposed on the individual data values. Statistical testing comparing the magnitudes will be based on positive responders only. The main analyses will focus on T-cells expressing at least one of the two cytokines IFN-γ or IL-2. Additional analyses will consider T-cells expressing polyfunctionality degree 1, 2, or 3, as well as considering T-cells expressing particular patterns of the three cytokines.

6.4.5 Analyses prior to end of study

6.4.5.1 Safety

During the course of the trial, unblinded analyses of safety data will be prepared approximately every 4 months for review by the SMB. Ad hoc safety reports may also be prepared for SMB review at the request of the HVTN 094 PSRT. The HVTN leadership must approve any other requests for unblinded safety data prior to the end of the study.

6.4.5.2 Immunogenicity

An unblinded statistical analysis by treatment assignment of an immunogenicity endpoint may be performed when the Laboratory Program has completed testing at least 80% of samples from the primary immunogenicity visit and all participants have completed the visit. The Laboratory Program will review the analysis report prior to distribution to the protocol chairs, DAIDS, vaccine developer, and other key HVTN members and investigators. Distribution will be limited to those with a need to know for the purpose of informing future trial-related decisions. The HVTN leadership must approve any other requests for HVTN immunogenicity analyses prior to the end of the study. Any analyses conducted prior to the end of the study should not compromise the integrity of the trial in terms of participant retention or safety or immunogenicity endpoint assessments.

7 Selection and withdrawal of participants

Participants will be healthy, HIV-1–uninfected (seronegative) adults who comprehend the purpose of the study and have provided written informed consent. Volunteers will be recruited and screened; those determined to be eligible, based on the inclusion and exclusion criteria, will be enrolled in the study. Final eligibility determination will depend on results of laboratory tests, medical history, physical examinations, and answers to self-administered and/or interview questions.

Investigators should always use good clinical judgment in considering a volunteer's overall fitness for trial participation. Some volunteers may not be appropriate for enrollment even if they meet all inclusion/exclusion criteria. Medical, psychiatric, occupational, or other conditions may make evaluation of safety and/or immunogenicity difficult, and some volunteers may be poor candidates for retention.

Administration of unattenuated vaccinia (DryVax) for smallpox vaccination has been recognized to be possibly associated with the occurrence of myo/pericarditis. This occurrence has been uncommon among vaccinia recipients. However, due to key differences such as that DryVax actively replicates in human cells as compared to the much more highly attenutated MVA which undergoes abortive replication in human cells, MVA is far less virulent than DryVax [73]. Myo/pericarditis has not been reported with MVA, a highly attenuated form of vaccinia. Within HVTN protocols approximately 400 volunteers have received MVA vaccinations and there have been no MVA associated cases of myo/pericarditis. However, this protocol will continue to include cardiac surveillance measures to ascertain any association of myo/pericarditis from MVA vaccination. Specific inclusion and exclusion criteria have been included in this protocol to minimize the chances of enrolling anyone with certain pre-existing cardiac conditions which may interfere with the assessments for myo/pericarditis or cardiovascular disease after MVA vaccination.

Determination of eligibility, taking into account all inclusion and exclusion criteria, must be made within 56 days prior to enrollment unless otherwise noted in Sections 7.1 and 7.2.

7.1 Inclusion criteria

1. **Age** of 18 to 50 years

2. **Access to a participating HVTN CRS** and willingness to be followed for the planned duration of the study

3. Ability and willingness to provide **informed consent**

4. **Assessment of understanding**: volunteer demonstrates understanding of this study and completes a questionnaire prior to first vaccination with verbal demonstration of understanding of all questionnaire items answered incorrectly

5. Willingness to receive **HIV test results**

6. Willingness to discuss **HIV infection risks**, amenable to **HIV risk reduction counseling**, and committed to maintaining behavior consistent with low risk of HIV exposure through the last required protocol clinic visit

7. **Willing to be contacted annually** after completion of scheduled clinic visits for a total of 3 years following initial study injection.

8. **Agrees not to enroll in another study** of an investigational research agent prior to completion of last required protocol clinic visit (excludes annual contacts for safety surveillance)

9. **Good general health** as shown by medical history, physical exam, and screening laboratory tests

10. Assessed by the clinic staff as being at **"low risk" for HIV infection**

11. **Hemoglobin** ≥ 11.0 g/dL for volunteers who were born female, ≥ 13.0 g/dL for volunteers who were born male

12. **White blood cell (WBC) count** = 3300 to 12,000 cells/mm3

13. **Total lymphocyte count** ≥ 800 cells/mm3

14. **Remaining differential** either within institutional normal range or with site physician approval

15. **Platelets** = 125,000 to 550,000/mm3

16. **Chemistry panel**: Alanine aminotransferase (ALT), aspartate aminotransferase (AST), alkaline phosphatase, and creatinine values ≤ institutional upper limits of normal

17. **Negative HIV-1 and -2 blood test**: US participants must have a negative FDA-approved immunoassay

18. **Negative Hepatitis B surface antigen (HBsAg)**

19. **Negative anti-Hepatitis C virus antibodies (anti-HCV)**, or negative HCV polymerase chain reaction (PCR) if the anti-HCV is positive

20. **Normal urine**:

    - Negative urine glucose, and

    - Negative or trace urine protein, and

    - Negative or trace urine hemoglobin (if trace hemoglobin is present on dipstick, a microscopic urinalysis for RBCs within institutional normal range).

21. **Volunteers who were born female**: negative serum or urine beta human chorionic gonadotropin (β-HCG) pregnancy test performed prior to vaccination on the day of initial vaccination

22. **Reproductive status**: A volunteer who was born female must:

- Agree to consistently use effective contraception (see Appendix B) for sexual activity that could lead to pregnancy from at least 21 days prior to enrollment through the last required protocol clinic visit. Effective contraception is defined as using any of the following methods:
    - Condoms (male or female) with or without a spermicide,
    - Diaphragm or cervical cap with spermicide,
    - Intrauterine device (IUD),
    - Hormonal contraception, or
    - Successful vasectomy in the male partner (considered successful if a volunteer reports that a male partner has [1] documentation of azoospermia by microscopy, or [2] a vasectomy more than 2 years ago with no resultant pregnancy despite sexual activity postvasectomy);
- Or not be of reproductive potential, such as having reached menopause (no menses for 1 year) or having undergone hysterectomy, bilateral oophorectomy, or tubal ligation;
- Or be sexually abstinent.

23. **Volunteers who were born female must also agree not to seek pregnancy through alternative methods**, such as artificial insemination or *in vitro* fertilization until after the last required protocol clinic visit

7.2 Exclusion criteria

1. **Vaccinia (smallpox) vaccine** determined by (1) clinical evidence of vaccinia scarification; (2) self-reported history of vaccinia vaccination; (3) date of birth; or (4) US military service prior to 1989 or after December 2002 (Not excluded: a participant born before 1975, or with past US military service, who self reports he/she did not receive vaccinia [smallpox] vaccine and has no evidence of scarification.)

2. **Untreated or incompletely treated syphilis infection**

3. **HIV vaccine(s)** received in a prior HIV vaccine trial. For potential participants who have received control/placebo in an HIV vaccine trial, the HVTN 094 PSRT will determine eligibility on a case-by-case basis.

4. **Non-HIV experimental vaccine(s)** received within the last 5 years in a prior vaccine trial. Exceptions may be made for vaccines that have subsequently undergone licensure by the FDA. For potential participants who have received control/placebo in an experimental vaccine trial, the HVTN 094 PSRT will determine eligibility on a case-by-case basis. For potential participants who have received an experimental vaccine(s) greater than 5 years ago, eligibility for enrollment will be determined by the HVTN 094 PSRT on a case-by-case basis.

5. **Immunosuppressive medications** received within 168 days before first vaccination. (Not excluded: [1] corticosteroid nasal spray for allergic rhinitis; [2] topical

corticosteroids for mild, uncomplicated dermatitis; or [3] oral/parenteral corticosteroids given for non-chronic conditions not expected to recur [length of therapy 10 days or less with completion at least 30 days prior to enrollment].)

6. **Blood products** received within 120 days before first vaccination

7. **Immunoglobulin** received within 60 days before first vaccination

8. **Live attenuated vaccines** other than influenza vaccine received within 30 days before first vaccination or scheduled within 14 days after injection (eg, measles, mumps, and rubella [MMR]; oral polio vaccine [OPV]; varicella; yellow fever)

9. **Investigational research agents** received within 30 days before first vaccination

10. **Intent to participate in another study** of an investigational research agent during the planned duration of the HVTN 094 study

11. **Influenza vaccine or any vaccines that are not live attenuated vaccines** and were received within 14 days prior to first vaccination (eg, tetanus, pneumococcal, Hepatitis A or B)

12. **Allergy treatment with antigen injections** within 30 days before first vaccination or that are scheduled within 14 days after first vaccination

13. **Current anti-tuberculosis (TB) prophylaxis or therapy**

14. **Clinically significant medical condition**, physical examination findings, clinically significant abnormal laboratory results, or past medical history with clinically significant implications for current health. A clinically significant condition or process includes but is not limited to:

    - A process that would affect the immune response,
    - A process that would require medication that affects the immune response,
    - Any contraindication to repeated injections or blood draws,
    - A condition that requires active medical intervention or monitoring to avert grave danger to the participant's health or well-being during the study period,
    - A condition or process for which signs or symptoms could be confused with reactions to vaccine, or
    - Any condition specifically listed among the exclusion criteria below.

15. **Any medical, psychiatric, occupational, or other condition** that, in the judgment of the investigator, would interfere with, or serve as a contraindication to, protocol adherence, assessment of safety or reactogenicity, or a participant's ability to give informed consent

16. **Serious adverse reactions to vaccines** including anaphylaxis and related symptoms such as hives, respiratory difficulty, angioedema, and/or abdominal pain. (Not excluded: a participant who had a nonanaphylactic adverse reaction to pertussis vaccine as a child.)

17. **Hypersensitivity to eggs or egg products**

18. **History of myocarditis, pericarditis, cardiomyopathy, congestive heart failure with permanent sequelae, clinically significant arrhythmia** (including any arrhythmia requiring medication, treatment, or clinical follow-up).

19. **ECG with clinically significant findings**, or features that would interfere with the assessment of myo/pericarditis, as determined by a contract ECG Lab or cardiologist, including any of the following: (1) conduction disturbance (complete left or complete right bundle branch block or nonspecific intraventricular conduction disturbance with QRS ≥120 ms, PR interval≥220ms, any 2nd or 3rd degree AV block, or QTc prolongation (>450ms)); (2) repolarization (ST segment or T wave) abnormality that will interfere with the assessment of myo/pericarditis; (3) significant atrial or ventricular arrhythmia; (4) frequent atrial or ventricular ectopy (eg, frequent premature atrial contractions, 2 premature ventricular contractions in a row); (5) ST elevation consistent with ischemia; (6) evidence of past or evolving myocardial infarction

20. **Autoimmune disease**

21. **Immunodeficiency**

22. **Asthma** other than mild, well-controlled asthma. Exclude a participant who:

    - Generally uses a bronchodilator (beta$_2$ agonist) daily, or

    - In the past year, has (any of the following):

        - Had > 1 exacerbation of symptoms treated with oral steroids (Note: oral/parenteral steroid use for asthma is exclusionary within 168 days before first vaccination.);

        - Routinely used moderate to high dose inhaled corticosteroids (eg, more than the equivalent of 250 mcg fluticasone; 400 mcg budesonide; 500 mcg beclomethasone; or 1000 mcg triamcinolone/flunisolide, as a daily dose) or theophylline for asthma; or

        - Needed emergency care, urgent care, hospitalization, or intubation for asthma.

23. **Diabetes mellitus** type 1 or type 2, including cases controlled with diet alone. (Not excluded: history of isolated gestational diabetes.)

24. **Thyroidectomy, or thyroid disease** requiring medication during the last 12 months

25. History of hereditary **angioedema**, acquired angioedema, or idiopathic angioedema.

26. **Hypertension**:

    - If a person has been found to have elevated blood pressure or hypertension during screening or previously, exclude for blood pressure that is not well controlled. Well-controlled blood pressure is defined as consistently ≤ 140 mm Hg systolic and ≤ 90 mm Hg diastolic, with or without medication, with only isolated, brief instances of higher readings, which must be ≤ 150 mm Hg systolic and ≤ 100 mm Hg diastolic. For these participants, blood pressure must be ≤ 140 mm Hg systolic and ≤ 90 mm Hg diastolic at enrollment.

- If a person has NOT been found to have elevated blood pressure or hypertension during screening or previously, exclude for systolic blood pressure ≥ 150 mm Hg at enrollment or diastolic blood pressure ≥ 100 mm Hg at enrollment.

27. **Body mass index (BMI)** ≥ 40; or BMI ≥ 35 with 2 or more of the following: age > 45, systolic blood pressure > 140 mm Hg, diastolic blood pressure > 90 mm Hg, current smoker, known hyperlipidemia

28. **Bleeding disorder** diagnosed by a doctor (eg, factor deficiency, coagulopathy, or platelet disorder requiring special precautions)

29. **Malignancy** (Not excluded: a participant with a surgical excision and subsequent observation period that in the investigator's estimation has a reasonable assurance of sustained cure or is unlikely to recur during the period of the study.)

30. **Seizure disorder** (Not excluded: a participant with a history of seizures who has not required medications or had a seizure within the past 3 years.)

31. **Asplenia**: any condition resulting in the absence of a functional spleen

32. **Psychiatric condition that precludes compliance with the protocol**. Specifically excluded are persons with psychoses within the past 3 years, ongoing risk for suicide, or history of suicide attempt or gesture within the past 3 years.

33. **Pregnant or breastfeeding**

7.3 Participant departure from vaccination schedule or withdrawal

This section concerns an individual participant's departure from the vaccination schedule. Pause rules for the trial as a whole are described in Section 11.3.1.

7.3.1 Delaying vaccinations for a participant

Under certain circumstances, a participant's scheduled vaccination will be delayed. The factors to be considered in such a decision include but are not limited to the following:

- Live attenuated vaccines other than influenza vaccine received within 30 days prior to any vaccination,

- Influenza vaccine or any vaccines that are not live attenuated vaccines (eg, pneumococcal) and were received within 14 days prior to any vaccination,

- Allergy treatment with antigen injections within 30 days prior to any study injection,

- Blood products or immunoglobulin received within 45 days prior to any vaccination, or

- Prevaccination abnormal vital signs or clinical symptoms that may mask assessment of vaccine reaction.

Vaccinations should not be administered outside the visit window period specified in the HVTN 094 Study Specific Procedures.

In order to avoid vaccination delays and missed vaccinations, participants who plan to receive licensed vaccines, allergy treatments, or other investigational research agents should be counseled to schedule receipt of these substances outside the intervals indicated above, when possible. Because their effects on safety and immunogenicity assessments and their interactions with study vaccines are unknown, if circumstances allow, these substances should also be avoided in the 2 week interval between a study vaccination and completion of the scheduled 2 week postvaccination follow-up visit.

7.3.2 Participant departure from vaccination schedule

Every effort should be made to follow the vaccination schedule per the protocol. If a participant misses a vaccination and the visit window period for the vaccination has passed, that vaccination cannot be given. The participant should be asked to continue study visits. The participant should resume the vaccination schedule with the next vaccination unless there are circumstances that require further delay or permanent discontinuation of vaccination (see Sections 7.3.1 and 7.3.3).

7.3.3 Discontinuing vaccination for a participant

Under certain circumstances, an individual participant's vaccinations will be permanently discontinued. Specific events that will result in stopping a participant's vaccination schedule include:

- Co-enrollment in a study with an investigational research agent (rare exceptions allowing for the continuation of vaccinations may be granted with the unanimous consent of the HVTN 094 PSRT).

- Clinically significant condition (ie, a condition that affects the immune system or for which continued vaccinations and/or blood draws may pose additional risk), including but not limited to the following:
  - Pregnancy (regardless of outcome);
  - Any grade 4 local or systemic reactogenicity symptom, lab abnormality, or AE that is subsequently considered to be related to vaccination;
  - Any grade 3 lab abnormality or other clinical AE (exception: fever or vomiting and subjective local and systemic symptoms) that is subsequently considered to be related to vaccination; or
  - Clinically significant type 1 hypersensitivity reaction associated with study vaccination. Consultation with the HVTN 094 PSRT is required prior to subsequent vaccinations following any type 1 hypersensitivity reaction associated with study vaccination; or

- Investigator determination in consultation with Protocol Team leadership (eg, for repeated nonadherence to study staff instructions).

Such participants should be counseled on the importance of continuing with the study and strongly encouraged to participate in follow-up visits and protocol-related procedures (unless medically contraindicated) per the protocol for the remainder of the trial.

In addition, vaccinations will be stopped for participants diagnosed with HIV infection. HIV-infected participants will not continue in the trial (see Sections 9.6).

7.3.4 Participant termination from the study

Under certain circumstances, an individual participant may be terminated from participation in this study. Specific events that will result in early termination include:

- Participant refuses further participation,
- Participant relocates and remote follow-up or transfer to another HVTN CRS is not possible,
- HVTN CRS determines that the participant is lost to follow-up,
- Participant becomes HIV infected, or
- Investigator decides, in consultation with Protocol Team leadership, to terminate participation (eg, if participant exhibits inappropriate behavior toward clinic staff).

8 Study product preparation and administration

CRS pharmacists should consult the Pharmacy Guidelines and Instructions for DAIDS Clinical Trials Networks for standard pharmacy operations. The protocol schema is shown in Table 3-1. See the Investigator's Brochures for further information about study products.

8.1 Vaccine regimen

The schedule of vaccination is shown in Section 3 and additional information is given below.

Group 1

Treatment 1 (T1): Plasmid DNA GEO-D03 vaccine 0.3 mg to be administered as 1 mL IM in either deltoid at Months 0 and 2.

AND

MVA/HIV62 vaccine $1x10^8$ $TCID_{50}$ to be administered as 1 mL IM in either deltoid at Months 4, 6, and 8.

MVA/HIV62 vials are labeled as $1x10^{7.5}$ $TCID_{50}$, actual average $1x10^{8.0}$ $TCID_{50}$

Placebo 1 (P1): Placebo for Plasmid DNA GEO-D03 (administered as Sodium Chloride for Injection USP, 0.9%) to be administered as 1 mL IM in either deltoid at Months 0 and 2.

AND

Placebo for MVA/HIV62 (administered as Sodium Chloride for Injection USP, 0.9%) to be administered as 1mL IM in either deltoid at Months 4, 6, and 8

Group 2

Participants (Group 2) in Version 1.0 were randomized to treatment 2 (T2) or placebo 2 (P2)

Treatment 2 (T2): Plasmid DNA GEO-D03 vaccine 3mg to be administered as 1mL IM in either deltoid at Months 0 and 2.

AND

MVA/HIV62 vaccine $1x10^8$ $TCID_{50}$ to be administered as 1mL IM in either deltoid at Months 4, 6, and 8.

MVA/HIV62B vials are labeled as $1x10^{7.5}$ $TCID_{50}$, actual average $1x10^{8.0}$ $TCID_{50}$

Placebo 2 (P2): Placebo for Plasmid DNA GEO-D03 (administered as Sodium Chloride for Injection USP, 0.9%) to be administered as 1 mL IM in either deltoid at Months 0 and 2.

AND

Placebo for MVA/HIV62 administered as Sodium Chloride for Injection USP, 0.9%) to be administered as 1mL IM in either deltoid at Months 4, 6, and 8.

<u>Participants (original Group 2) consented for Version 2.0 will have their vaccination schedule changed as follows:</u>

The first 18 PTIDS originally enrolled in Group 2 (Version 1.0) will continue in Group 2 (Version 2.0) but will no longer receive a Month 8 injection. They will receive MVA or Placebo at Month 10 instead:

Treatment 2 (T2): Plasmid DNA GEO-D03 vaccine 3mg to be administered as 1mL IM in either deltoid at Months 0 and 2 (already administered).

AND

MVA/HIV62 vaccine $1x10^8$ $TCID_{50}$ to be administered as 1mL IM in either deltoid at Months 4, 6, and 10.

MVA/HIV62B vials are labeled as $1x10^{7.5}$ $TCID_{50}$, actual average $1x10^{8.0}$ $TCID_{50}$

Placebo 2 (P2): Placebo for Plasmid DNA GEO-D03 (administered as Sodium Chloride for Injection USP, 0.9%) to be administered as 1 mL IM in either deltoid at Months 0 and 2 (already administered).

AND

Placebo for MVA/HIV62 administered as Sodium Chloride for Injection USP, 0.9%) to be administered as 1mL IM in either deltoid at Months 4, 6, and 10.

The last 18 PTIDS originally enrolled in Group 2 (Version 1.0) will be switched to Group 3 (Version 2.0) and will no longer receive a Month 6 injection. They will continue to receive all of the other injections they were originally randomized to under Version 1.0.

Treatment 3 (T3): Plasmid DNA GEO-D03 vaccine 3mg to be administered as 1mL IM in either deltoid at Months 0 and 2 (already administered under T2 (Version 1.0)).

AND

MVA/HIV62 vaccine 1x10^8 $TCID_{50}$ to be administered as 1mL IM in either deltoid at Months 4 and 8.

MVA/HIV62B vials are labeled as 1x$10^{7.5}$ $TCID_{50}$, actual average 1x$10^{8.0}$ $TCID_{50}$

Placebo 3 (P3): Placebo for Plasmid DNA GEO-D03 (administered as Sodium Chloride for Injection USP, 0.9%) to be administered as 1 mL IM in either deltoid at Months 0 and 2 (already administered under P2 (Version 1.0)).

AND

Placebo for MVA/HIV62 administered as Sodium Chloride for Injection USP, 0.9%) to be administered as 1mL IM in either deltoid at Months 4 and 8.

For all participants originally enrolled in Group 2, a new prescription must be written and sent to the pharmacy once the participant has consented under Version 2.0 with the appropriate vaccination schedule for the MVA or placebo. This will serve as a double check for pharmacy that they have the correct information in the participant files in the pharmacy.

8.2 Study product formulation

See the Investigator's Brochure (IB) for further information about the study products.

GEO-D03 DNA vaccine, 3 mg DNA/mL (labeled as GEO-D03 Plasmid DNA)
The GEO-D03 DNA vaccine (labeled as GEO-D03 Plasmid DNA, 3 mg DNA/mL) is supplied as an opalescent to clear, colorless solution in single-dose vials containing a volume to deliver 1 mL of 3 mg/mL DNA. The DNA is formulated in a formulation buffer consisting of phosphate-buffered saline (PBS), EDTA (ethylenediamine tetraacetic acid), and ethanol. The vials containing study product must be stored frozen at −70°C or colder.

Formulation Buffer for GEO-D03 DNA (labeled as Diluent)
The Buffer for GEO-D03 DNA (labeled as Diluent) is supplied as a clear, colorless solution in a single dose vial consisting of phosphate-buffered saline (PBS), EDTA (ethylenediamine tetraacetic acid), and ethanol. Each vial contains 4.5 mL of buffer. The vials containing study product must be stored frozen at −70°C or colder.

Placebo for GEO-D03 DNA vaccine
Sodium Chloride for Injection USP, 0.9% will be used as the placebo for GEO-D03 DNA and must be stored as directed by the manufacturer.

MVA/HIV62 vaccine (MVA62B vaccine, labeled as MVA/HIV62 10 ^7.5 $TCID_{50}$/mL**)**
The MVA/HIV62 vaccine to be used for preparation of the 1×10^8 $TCID_{50}$ dose is supplied as an opalescent to off-white suspension in single-dose vials containing a volume to deliver 1 mL of $1\times10^{8\pm0.5}$ $TCID_{50}$. The vaccine is formulated in a formulation buffer consisting of PBS and 7.5% sucrose. The lot was manufactured by BioReliance Ltd., Glasgow, Scotland. The vials containing study product must be stored frozen at −70°C or colder.

Placebo for MVA/HIV62 vaccine
Sodium Chloride for Injection USP, 0.9% will be used as the placebo for MVA/HIV62 and must be stored as directed by the manufacturer.

8.3 Preparation of study products

8.3.1 GEO-D03 0.3mg

One vial of GEO-D03 3 mg/mL (labeled as GEO-D03 Plasmid DNA, 3 mg DNA/mL) and one vial of Formulation Buffer for GEO-D03 DNA (labeled as Diluent) will be needed to prepare this dose. Prior to injection, the Pharmacist will remove both vials from the freezer and allow it to thaw in a 2° to 8°C refrigerator for 2 to 18 hours. During this 2 to 18 hour period, if the products are not completely thawed and a volunteer is ready to be vaccinated, the pharmacist may hold the vials in his/her hand to complete the thawing process. The thawed vaccine and buffer can be held at 2° to 8°C for up to 5 days (120 hours) after removal from the freezer. THEY MUST NOT BE STORED at room temperature.

Immediately prior to preparation, the pharmacist should remove both vials from the refrigerator. The pharmacist will take both vials and invert them gently several times. DO NOT SHAKE. Using aseptic technique, the pharmacist should withdraw 0.5 mL of GEO-D03 3mg/mL into a 1 mL syringe. The contents of this syringe will then be slowly injected into the vial containing 4.5 mL of *BUFFER*. The pharmacist should avoid creating bubbles. To prevent aerosolization, the pharmacist should, before removing the needle from the vial, bring the needle above the level of the liquid and allow excess air to rise into the syringe. The pharmacist will then remove the needle from the vial and discard into a sharps container. The pharmacist will then invert this vial several times. The final product will contain 0.3mg/mL of GEO-D03. Using a new 3 or 5mL syringe, the pharmacist will withdraw 1 mL of this final product. The syringe containing the product must not be held at room temperature. Once drawn into the syringe, the vaccine should be kept cold (2° to 8°C) until it is ready to be injected into the participant. The syringe should be labeled as *GEO-D03 vaccine 0.3 mg DNA or Placebo*. The syringe should also be labeled with a 24 hour expiration date from the time product was withdrawn from the admixture into the syringe. (Note: If the vaccine is not withdrawn from the vial until 96 hours or more after removal from the freezer, then the actual expiration date/time should be used).

Any unused portion of entered vials and expired prefilled syringes should be disposed of as biohazardous waste in accordance with institutional or pharmacy policy for a biological safety level RG1 product.

8.3.2 Placebo for GEO-D03 0.3mg

One vial of Placebo for GEO-D03 0.3 mg (labeled as Sodium Chloride for Injection USP, 0.9%) will be needed to prepare this dose. Prior to injection, the Pharmacist will remove the vial from storage and place it in a 2° to 8°C refrigerator for 2 to 18 hours.

Immediately prior to withdrawing a 1 mL dose into a 3 or 5 mL syringe, the vial should be removed from the refrigerator. Using aseptic technique, the pharmacist should withdraw the entire contents of the vial, up to 1 mL, into a 3 or 5 mL syringe. The syringe

containing the product must not be held at room temperature. Once drawn into the syringe, the placebo should be kept cold (2° to 8°C) until it is ready to be injected into the participant. The syringe should be labeled as *GEO-D03 vaccine 0.3 mg DNA or Placebo*. The syringe should also be labeled with a 24 hour expiration date from the time product was withdrawn from the refrigerated vial into the syringe. (Note: If the placebo is not withdrawn from the vial until 96 hours or more after placing in the refrigerator, then the actual expiration date/time should be used).

8.3.3 GEO-D03 3 mg

One vial of GEO-D03 3 mg/mL (labeled as Plasmid DNA GEO-D03, 3 mg DNA/mL) will be needed to prepare this dose. Prior to injection, the Pharmacist will remove the 3 mg DNA/mL vial of vaccine from the freezer and allow it to thaw in a 2° to 8°C refrigerator for 2 to 18 hours. During this 2 to 18 hour period, if the vaccine is not completely thawed and a volunteer is ready to be vaccinated, the pharmacist may hold the vial in his/her hand to complete the thawing process. The thawed vaccine can be held at 2° to 8°C for up to 5 days (120 hours) after removal from the freezer. IT MUST NOT BE STORED at room temperature.

Immediately prior to withdrawing a 1 mL dose into a 3 or 5 mL syringe, the vial should be removed from the refrigerator and inverted gently several times. DO NOT SHAKE. Using aseptic technique, the pharmacist should withdraw the entire contents of the vial, up to 1 mL, into a 3 or 5 mL syringe. The syringe containing the product must not be held at room temperature. Once drawn into the syringe, the vaccine should be kept cold (2° to 8°C) until it is ready to be injected into the participant. The syringe should be labeled as *GEO-D03 vaccine 3 mg DNA or Placebo*. The syringe should also be labeled with a 24 hour expiration date from the time product was withdrawn from the thawed vial into the syringe. (Note: If the vaccine is not withdrawn from the vial until 96 hours or more after removal from the freezer, then the actual expiration date/time should be used).

Any unused portion of entered vials and expired prefilled syringes should be disposed of as biohazardous waste in accordance with institutional or pharmacy policy for a biological safety level RG1 product.

8.3.4 Placebo for GEO-D03 3 mg

One vial of Placebo for GEO-D03 3 mg/mL (labeled as Sodium Chloride for Injection USP, 0.9%) will be needed to prepare this dose. Prior to injection, the Pharmacist will remove the vial from storage and place it in a 2° to 8°C refrigerator for 2 to 18 hours.

Immediately prior to withdrawing a 1 mL dose into a 3 or 5 mL syringe, the vial should be removed from the refrigerator. Using aseptic technique, the pharmacist should withdraw the entire contents of the vial, up to 1 mL, into a 3 or 5 mL syringe. The syringe containing the product must not be held at room temperature. Once drawn into the syringe, the placebo should be kept cold (2° to 8°C) until it is ready to be injected into the participant. The syringe should be labeled as *GEO-D03 vaccine 3 mg DNA or Placebo*. The syringe should also be labeled with a 24 hour expiration date from the time product was withdrawn from the refrigerated vial into the syringe. (Note: If the placebo is not withdrawn from the vial until 96 hours or more after placing in the refrigerator, then the actual expiration date/time should be used).

8.3.5 MVA/HIV62

One vial of MVA/HIV62 (labeled as MVA/HIV62 $1\times10^{7.5}$ $TCID_{50}$/mL) will be needed to prepare this dose. Prior to injection, the pharmacist will remove the vial from the freezer and allow it to thaw in a 2° to 8° C refrigerator for 2 to 18 hours. During this 2 to 18 hour period, if the vaccine is not completely thawed and a volunteer is ready to be vaccinated, the pharmacist may hold the vial in his/her hand to complete the thawing process. The thawed MVA/HIV62 vaccine can be held at 2° to 8° C for up to 5 days (120 hours) after removal from the freezer. Do not STORE at room temperature.

Immediately prior to withdrawing a 1 mL dose into a 3 or 5 mL syringe, the vial should be removed from the refrigerator and inverted gently several times. DO NOT SHAKE. Using aseptic technique, the pharmacist should withdraw the entire contents of the vial, up to 1 mL, into a 3 or 5 mL syringe. The syringe containing the product must not be held at room temperature. Once drawn into the syringe, the vaccine should be kept cold (2° to 8°C) until it is ready to be injected into the participant. The syringe should be labeled as *MVA/HIV62 vaccine* $1x10^8$ $TCID_{50}$ *or Placebo*. The syringe should also be labeled with a 24 hour expiration date from the time product was withdrawn from the thawed vial into the syringe. (Note: If the vaccine is not withdrawn from the vial until 96 hours or more after removal from the freezer, then the actual expiration date/time should be used).

Any unused portion of entered vials and expired prefilled syringes should be disposed of as biohazardous waste in accordance with institutional or pharmacy policy for a biological safety level RG1 product.

8.3.6 Placebo for MVA/HIV62

One vial of Placebo for MVA/HIV62 (labeled as Sodium Chloride for Injection USP, 0.9%) will be needed to prepare this dose. Prior to injection, the Pharmacist will remove the vial from storage and place it in a 2° to 8°C refrigerator for 2 to 18 hours.

Immediately prior to withdrawing a 1 mL dose into a 3 or 5 mL syringe, the vial should be removed from the refrigerator. Using aseptic technique, the pharmacist should withdraw the entire contents of the vial, up to 1 mL, into a 3 or 5 mL syringe. The syringe containing the product must not be held at room temperature. Once drawn into the syringe, the placebo should be kept cold (2° to 8°C) until it is ready to be injected into the participant. The syringe should be labeled as *MVA/HIV62 vaccine* $1x10^8$ $TCID_{50}$ *or Placebo*. The syringe should also be labeled with a 24 hour expiration date from the time product was withdrawn from the refrigerated vial into the syringe. (Note: If the placebo is not withdrawn from the vial until 96 hours or more after removal from storage, then the actual expiration date/time should be used).

8.4 Administration

Prior to administration, the study product in the syringe should be removed from the refrigerator. It may be warmed to room temperature or warmer by holding in one’s hand for several minutes at the participant’s side immediately prior to administration.

The GEO-D03 or placebo and the MVA/HIV62 or placebo vaccinations are to be given intramuscularly into the deltoid muscle after preparation of the site with alcohol.

When preparing a dose in a syringe and administering the dose, consideration should be given to the volume of solution in the needle before and after the dose is administered. Particularly, if the needle used to withdraw the product is replaced prior to vaccine administration, consideration should be given to conserving the full dose of product. The pharmacy and clinic staff members are encouraged to work together to administer the dose specified in the protocol.

At sites where registered pharmacists are legally authorized to administer drug, the HVTN CRS may choose to have the HVTN CRS pharmacist administer the vaccinations.

8.5 Acquisition of study products

Plasmid DNA GEO-D03, Formulation Buffer (labeled as Diluent), and MVA/HIV62 will be provided by GeoVax, Inc. Sodium Chloride for Injection USP, 0.9% must be purchased by the site.

Once an HVTN CRS is protocol registered, the pharmacist can obtain study products from the NIAID Clinical Research Products Management Center (CRPMC) by following the ordering procedures given in Pharmacy Guidelines and Instructions for DAIDS Clinical Trials Networks.

8.6 Pharmacy records

The HVTN CRS pharmacist is required to maintain complete records of all study products. As the Sodium Chloride for Injection USP, 0.9% will be used from site supplies, the continuous inventory is not required, but all other information must be completed (including the lot number for the vial/ampule/IV solution used). The pharmacist of record is responsible for maintaining randomization codes and randomization confirmation notices for each participant in a secure manner.

8.7 Final disposition of study products

All unused study products must be returned to the CRPMC after the study is completed or terminated unless otherwise instructed by the CRPMC. The procedures and relevant form are included in the Pharmacy Guidelines and Instructions for DAIDS Clinical Trials Networks.

9 Clinical procedures

The schedule of clinical procedures is shown in Appendix J, Appendix K, and Appendix L. Group 2 includes additional visits.

9.1 Informed consent

Informed consent is the process of ensuring that participants fully understand what will and may happen to them while participating in a research study. The HVTN informed consent form documents that a participant (1) has been informed about the potential risks, benefits, and alternatives to participation, and (2) is willing to participate in an HVTN study. Informed consent encompasses all written or verbal study information HVTN CRS staff provide to the participant, before and during the trial. HVTN CRS staff will obtain informed consent of participants according to HVTN policies and procedures.

The informed consent process continues throughout the study. Key study concepts should be reviewed periodically with the participant and the review should be documented. At each study visit, HVTN CRS staff should consider reviewing the procedures and requirements for that visit and for the remaining visits. Additionally, if any new information is learned that might affect the participants' decisions to stay in the trial, this information will be shared with trial participants. If necessary, participants will be asked to sign revised informed consent forms.

An HVTN CRS may employ recruitment efforts prior to the participant consenting. For example, some HVTN CRSs use a telephone script to prescreen people before they come to the clinic for a full screening visit. Participants must sign a screening or protocol-specific consent before any procedures are performed to determine eligibility. HVTN CRSs must submit recruitment and prescreening materials to IRBs/ECs for human subjects protection review and approval.

9.1.1 Screening consent form

Some HVTN CRSs have approval from their local IRB or EC to use a general screening consent form that allows screening for an unspecified HIV vaccine trial. In this way, HVTN CRS staff can continually screen potential participants and, when needed, proceed quickly to obtain protocol-specific enrollment consent. Sites conducting IRB/EC–approved general screening or prescreening may use the results from this screening to determine eligibility for this protocol, provided the tests are conducted within the time periods specified in the eligibility criteria. Without a general screening consent, screening for a specific study cannot take place until the site is activated by HVTN Regulatory Affairs.

9.1.2 Protocol-specific consent forms

The protocol-specific consent form describes the study products to be used and all aspects of protocol participation, including screening and enrollment procedures. A sample protocol-specific consent form for the main study is located in Appendix A.

Each HVTN CRS is responsible for developing a protocol-specific consent form for local use, based on the sample protocol-specific consent form in Appendix A. The consent

form must be developed in accordance with local IRB/EC requirements and the principles of informed consent as described in Title 45, Code of Federal Regulations (CFR) Part 46 and Title 21 CFR, Part 50, and in the International Conference on Harmonisation (ICH) E6, Good Clinical Practice: Consolidated Guidance 4.8.

Study sites are strongly encouraged to have their local CABs review their sites-specific consent forms. This review should include, but should not be limited to, issues of cultural competence, local language considerations, and the level of understandability.

The sample informed consent form includes instructions throughout the document for developing specific content.

Sites should follow the instructions in the Protocol-specific Official Memo distributed along with this protocol regarding when they may begin using their site-specific protocol consent forms.

Regarding protocol registration, sites should follow procedures outlined in the current version of the DAIDS Protocol Registration Manual.

9.1.3 VISP registry consent form

Experimental HIV vaccines may induce antibody production to HIV antigens, producing reactive results on commercially available HIV test kits. This is called “vaccine-induced seropositivity” (VISP) (see Section 9.6.1). In order to provide poststudy HIV testing to distinguish between VISP and HIV infection, and to mitigate potential social harms resulting from VISP in HIV vaccine recipients who are not infected with HIV, the HVTN has created a VISP registry. Following study unblinding, the registry will allow HVTN counselors to verify that an individual has received an HIV vaccine, and therefore has the potential for VISP. Information in the VISP registry will not be used for research. Rather, the registry exists to support provision of poststudy testing and counseling services to HIV vaccine recipients.

The VISP registry consent form describes the purpose of the VISP registry, the participant information to be included in the registry, confidentiality protections, and risks and benefits associated with inclusion in the registry. The VISP registry consent form is contained in Appendix C.

The VISP Registry consent form will be presented to all participants. It is recommended to be presented no later than the last scheduled vaccination visit.

9.1.4 Assessment of Understanding

Study staff are responsible for ensuring that participants fully understand the study before enrolling them. This process involves reviewing the informed consent form with the participant, allowing time for the participant to reflect on the procedures and issues presented, and answering all questions completely.

An Assessment of Understanding is used to document the participant’s understanding of key concepts in this HIV vaccine trial. The participant must complete the Assessment of Understanding before enrollment. Staff may provide assistance in reading and understanding the questions and responses, if necessary. Participants must verbalize understanding of all questions answered incorrectly. This process and the participant’s understanding of the key concepts should be recorded in source documentation at the site.

IRBs/ECs may require that a participant has signed either a screening or protocol-specific consent document prior to administering the Assessment of Understanding. The consent process (including the use of the Assessment of Understanding) should be explained thoroughly to the IRB/EC, whose recommendations should be followed.

9.2 Pre-enrollment procedures

Screening may occur over the course of several contacts/visits, up to and including before vaccination on Day 0. All inclusion and exclusion criteria must be assessed within 56 days before enrollment, unless otherwise specified in the eligibility criteria (or below in this section).

After the appropriate informed consent has been obtained and before enrollment, the following procedures are performed:

- Medical history, documented in the case history record;
- Assessment of whether the volunteer is at low risk for HIV infection;
- Complete physical examination, including height, weight, vital signs, and clinical assessments of head, ears, eyes, nose, and throat; neck; lymph nodes; heart; chest; abdomen; extremities; neurological function; and skin;
- Assessment of concomitant medications the volunteer is taking, including prescription and nonprescription drugs, vitamins, topical products, alternative/complementary medicines (eg, herbal and health food supplements), recreational drugs, vaccinations, and allergy shots (record the complete generic name for all medications);
- 12-lead ECG with interpretation (see Section 9.4.1.3);
- Laboratory tests as defined in the inclusion and exclusion criteria, including:
  - Screening HIV test,
  - HBsAg,
  - Anti-HCV,
  - Syphilis test,
  - Complete blood count (CBC) with differential and platelets,
  - Chemistry panel (ALT, AST, alkaline phosphatase, and creatinine),
  - Urine dipstick (as described in Section 9.8), and
  - Urine or serum pregnancy test (participants who were born female).
- Administration of behavioral risk assessment questionnaire;
- Obtaining of volunteer demographics in compliance with the NIH Policy on Reporting Race and Ethnicity Data: Subjects in Clinical Research, Aug. 8, 2001 (available at http://grants.nih.gov/grants/guide/notice-files/NOT-OD-01-053.html);

- Counseling on HIV testing and risk reduction, performed in compliance with the US Centers for Disease Control and Prevention (CDC)'s current guidelines or other local guidelines for HIV counseling, testing, and referral as described in Section 9.6; and

- Discussion of pregnancy prevention. A pregnant or breastfeeding person may not be enrolled in this trial. Specific criteria and assessment of contraception and pregnancy status are described in study inclusion criteria. Discussion of pregnancy prevention includes advising a participant who was born female and who reports no current sexual activity that could lead to that participant becoming pregnant to have a plan to begin adequate birth control. This plan would be put to use if, during the study, the participant becomes sexually active in a way that could lead to that participant becoming pregnant.

9.2.1 Use of screening results from another HVTN study

If a participant screens for an HVTN study at the same HVTN CRS but then does not join that study, screening results from that effort may be applied to the screening for this protocol, as long as the screening was done under participant consent, the participant has signed a consent form to begin screening for this study, and the tests were conducted within the time periods specified in the eligibility criteria (see Sections 7.1 and 7.2).

9.3 Enrollment and vaccination visits

Enrollment is simultaneous with first vaccination. The time interval between randomization and enrollment should not exceed 4 working days. The HVTN CRS registers the participant by scheduling the day 0 visit (enrollment) via the Web-based randomization system, and requests the randomization assignment. Circumstances may require a participant's enrollment visit to be changed. This may exceed the 4-day randomization time limit.

At all vaccination visits, the following procedures are performed before vaccination:

- Abbreviated physical examination, including weight, vital signs, and a symptom-directed evaluation by history and/or appropriate physical exam based on participant self-reported symptoms or complaints;

- Assessment of baseline reactogenicity parameters;

- Assessment of concomitant medications (as described in Section 9.2);

- Assessment of any new or unresolved AEs/intercurrent illnesses; and

- Urine or serum pregnancy test (for participants who were born female).

Following completion of all procedures in the preceding list and results indicate that vaccination may proceed, vaccination is prepared and administered (see Sections 8.3 and 8.4).

Immediately following vaccination, the participant remains in the clinic for observation. An initial reactogenicity assessment is made at a target of 30 minutes after injection, with an acceptable range of 25-60 minutes. Before leaving the clinic, the participant is given

the postvaccination symptom log and is instructed on how to complete it. The site will make arrangements to obtain daily reports of reactogenicity events from the participant during the reactogenicity period (as described in Section 9.9).

The following procedures will be performed at all vaccination visits. These procedures may be performed prior to or following vaccination:

- Risk reduction counseling (as described in Section 9.6);
- Pregnancy prevention assessment (as described in Section 9.2 and 9.7); and
- Assessment of new or unresolved social impacts (site staff will ask participant about the status of any unresolved social impacts and if s/he has experienced any new social impacts as a result of the trial participation).

Additional procedures will be performed at scheduled visits as specified in Appendix J, Appendix K, and Appendix L:

- HIV infection assessment, including pretest counseling. A subsequent follow-up contact is conducted to provide post-test counseling and to report results to participant;
- Confirm that participants received HIV test results from previous visit. If not, provide test results and post-test counseling as appropriate; and
- Specimen collection (should be completed prior to vaccination)

9.4 Follow-up visits

The following procedures are performed at all scheduled follow-up visits:

- Assessment of new or continuing concomitant medications (as described in Section 9.2);
- Assessment of new or unresolved AEs/intercurrent illnesses; and

Additional procedures will be performed at scheduled follow-up visits as specified in Appendix J, Appendix K, and Appendix L:

- Risk reduction counseling (as described in Section 9.6);
- Pregnancy prevention assessment (as described in Section 9.2 and 9.7); and
- Assessment of new or unresolved social impacts (site staff will ask participant about the status of any unresolved social impacts and if s/he has experienced any new social impacts as a result of the trial participation);
- Administration of the social impact assessment questionnaire (types of impacts assessed involve personal relationships, health insurance, life insurance, educational or employment opportunities, housing, immigration, or travel);

- Administration of a questionnaire that asks the participant about any HIV testing he or she may have received outside of the study. Participants will also be asked whether they believe they received the active vaccine or the placebo;

- HIV infection assessment including pretest counseling. A subsequent follow-up contact is conducted to provide post-test counseling and to report results to participant;

- Confirm that participants received HIV test results from previous visit. If not, provide test results and post-test counseling as appropriate;

- Cardiac symptoms assessment (as described in Section 9.4.1)

- Abbreviated physical examination including weight, vital signs, and a symptom-directed evaluation by history and/or appropriate physical exam based on participant self-reported symptoms or complaints;

- Complete physical examination, including weight, vital signs, and clinical assessments of head, ears, eyes, nose, and throat; neck; lymph nodes; heart; chest; abdomen; extremities; neurological function; and skin;

- Specimen collection;

- Clinical laboratory tests including:
  - CBC with differential and platelet count,
  - Chemistry panel (see Section 9.2), and
  - Urine dipstick (urinalysis if appropriate; see Section 9.8); and

- Urine or serum pregnancy test (for participants who were born female).

9.4.1 Cardiac safety monitoring

Myo/pericarditis has been observed in recipients of vaccinia vaccinations used to protect against smallpox. It has been an *uncommon* occurrence in vaccinia recipients. Myo/pericarditis has not been documented to occur with MVA. Within HVTN protocols approximately 400 volunteers have received MVA vaccinations and there have been no MVA associated cases of myo/pericarditis. However, the safety of trial participants is the major priority for the HVTN and the Protocol Team, and thus enhanced cardiac safety monitoring has been added to this protocol to detect potential cardiac effects of MVA vaccine.

ECGs are performed at screening to establish a baseline and to exclude participants with pre-existing ECG findings which may interfere with post vaccination cardiac asssessments and as clinically indicated. ECG interpretation will be centralized, and provided by a contract ECG laboratory staffed by a cardiologist who has ongoing experience with interpreting ECGs from several other vaccinia and MVA trials. This should provide consistency in the application of ECG screening criteria for enrollment, in the quality of the ECG data, and in assessing ECG changes as in other MVA studies [74].

9.4.1.1 Cardiac symptoms assessment

At all MVA/placebo vaccination visits and at subsequent visits as indicated in Appendix J, Appendix K, and Appendix L, participants will be questioned specifically about symptoms and signs suggestive of myo/pericarditis or other cardiovascular complications as listed below:

- Shortness of breath,
- Chest pain/discomfort,
- Palpitations,
- Unexplained fatigue, and
- Fever, chills, myalgia/arthralgias.

If cardiopulmonary symptoms are reported, the study staff will perform a cardiopulmonary evaluation. Any report of these or any other signs and symptoms suggestive of any new cardiovascular condition will prompt an appropriate diagnostic evaluation as medically indicated.

9.4.1.2 Cardiac troponin

Testing for cTnI or cTnT, biomarkers for cardiac tissue injury, is performed for specific symptoms after enrollment as specified in Section 9.4.1.4. cTnI or cTnT tests will be performed at local laboratories. Sites should consult their local laboratories for information on specific handling requirements.

Any cTnT or cTnI result above the institutional upper limit of normal should be reported by phone or email to the SDMC Clinical Affairs staff within 24 hours (contact information listed in *HVTN 094 Study Specific Procedures, Key Resource Guide*), and repeated as soon as possible along with a CK-MB, and an additional ECG.

9.4.1.3 ECG testing

A 12-lead ECG is required at screening, and as clinically indicated. ECG equipment will be provided by the HVTN or accessed locally by the sites. ECG interpretation will be provided by a contract ECG laboratory.

9.4.1.4 Evaluation of suspected myo/pericarditis

The classic presentation of myo/pericarditis may not always be apparent with very early involvement. Since apparently benign symptoms may be suggestions of or mimic myo/pericarditis, there should be a low threshold for additional investigation of chest sensation or symptoms referable to the chest. As with evaluation for all potentially serious health problems in study participants, the protocol team recommends that the clinic physician be involved in the evaluation and clinical decision making associated with cardiac symptoms.

Any participant who develops symptoms suggestive of possible myo/pericarditis (such as chest pain, dyspnea, palpitations, congestive heart failure) following MVA/placebo vaccination will be evaluated with an ECG, cTnT or cTnI, and CK-MB by study staff as

long as performing these tests in the research setting does not interfere with prompt medical care of the participant. Symptoms or findings that lead to a cardiac evaluation or referral for suspected myo/pericarditis should be reported by phone or email to the SDMC Clinical Affairs staff within 24 hours (contact information listed in *HVTN 094 Study Specific Procedures, Key Resource Guide*).

The participant with symptoms and cardiac enzyme findings and/or ECG findings consistent with suspected or probable myo/pericarditis related to vaccine according to the CDC case definition [75], attached as Appendix N to this protocol, will be referred to a cardiologist for consultation and care. The site will communicate a request to the cardiologist that the initial evaluation include any of the following tests that have not been done previously for evaluation of that specific cardiac event: an ECG, cTnT or cTnI, CK-MB, and echocardiography. The site will request permission from the participant for access to medical records related to the evaluation. An AE of myo/pericarditis with a suspected causal relationship to vaccine would be followed by study staff until resolution and the participant will be contacted 1 year after the event to complete follow-up of the AE.

Any episode of myo/pericarditis at any grade must be reported to SDMC Clinical Affairs immediately and reported as an SAE, as described in Section 11.2.3. Study staff will follow any AE of myo/pericarditis until resolution.

9.5 Annual health contacts

Participants will be contacted annually for a total of 3 years following initial study injection (see Appendix M). At these contacts, CRS staff will collect the information listed below. Clinic visits will only be required if HIV confirmatory testing is necessary (see Section 9.6.1); however, a clinic visit may be arranged for other reasons.

- Confirmation of vital status; if deceased, attempt to learn cause and date of death;
- If participant is alive, record the participant's responses to questions regarding any occurrence of the following events since the last HVTN study contact:
    - Life-threatening adverse experiences;
    - Persistent or significant disability/incapacity;
    - Hospitalizations and reasons;
    - Other important medical events that may jeopardize the participant or may require intervention to prevent 1 of the other outcomes listed above;
    - New chronic conditions requiring more than 30 days of medical intervention or medication;
    - AESI's
    - New diagnosis of HIV infection; and
    - Pregnancies and outcomes, including congenital anomalies/birth defects.

All such events will be recorded, and adverse events will be assessed for relationship to study product(s).

9.5.1 Interim contacts

CRSs may report safety information obtained at a contact other than the annual contact. These contacts are reported as interim visits.

9.6 HIV counseling and testing

HIV counseling will be performed in compliance with the CDC's guidelines or other local guidelines for HIV counseling and referral. HIV testing will be performed in accordance with the current HVTN HIV testing algorithm following enrollment.

Participants will be counseled routinely during the trial on the avoidance of HIV infection and on the potential negative social impacts of testing antibody positive due to the vaccine. They will also be counseled on the risks of HIV antibody testing outside of the HVTN CRSs and will be discouraged from doing so during study participation and/or during any period of vaccine-induced positive serology.

Study staff will take particular care to inform study participants of the likelihood of routine HIV testing being offered or performed outside the study CRS at emergency rooms, clinics, and medical offices. Such testing has become more likely due to the CDC's revised guidelines for HIV counseling and testing, as well as policy changes in many countries to make HIV testing more frequent and routine. CRS staff should inform participants of their right to opt out of HIV testing outside the study site. CRS staff should inform study participants if local and/or state policies and regulations permit medical providers to perform HIV testing without first informing patients. If this is the case, then CRS staff should advise study participants that they may decline testing preemptively. CRS staff should also inform participants if positive results must be reported to local public health authorities. CRS staff should also inform participants of the need to maintain study blinding by getting HIV testing only at the study CRS. CRS staff should provide participants with CRS contact information and should encourage participants to ask medical providers to contact the CRS. The CRS can verify that the participant is in an HIV vaccine clinical trial and should only be tested at the study CRS.

Potential participants identified as being HIV infected during screening are not enrolled. All participants who become HIV infected during the study will be terminated from this study. Potential and enrolled participants identified as HIV infected will be referred for medical treatment, counseling, and management of the HIV infection. These individuals may also be referred to appropriate ongoing clinical trials or observational studies.

9.6.1 Distinguishing intercurrent HIV infection from vaccine-induced positive serology

The study product may elicit an antibody response to HIV proteins. Therefore, vaccine-induced positive serology may occur in this study. Several precautionary measures will be taken to distinguish intercurrent HIV infection from vaccine-induced positive serology. These precautionary measures include:

- Participants will have physical examinations at visits specified in Appendix J, Appendix K, and Appendix L. Signs or symptoms of an acute HIV infection syndrome, an intercurrent illness consistent with HIV-1 infection, or probable HIV exposure would prompt a diagnostic workup per the HVTN algorithm for Recent Exposure/Acute Infection Testing to determine HIV infection.

- HIV testing will be performed at multiple timepoints throughout the study (see Appendix J, Appendix K, and Appendix L). The Laboratory Program (or approved diagnostic laboratory) will follow the HVTN HIV testing algorithm (as described in the HVTN Site Lab Reference Manual), which is able to distinguish vaccine-induced antibody responses from actual HIV infections.

- All participants can receive HIV-1 diagnostic testing from the site following their last scheduled visit until they are told that they did not receive an HIV vaccine or that they do not have vaccine-induced seropositivity.

- All participants who received vaccine product and who have vaccine-induced positive or indeterminate HIV-1 serology (as measured by the standard anti-HIV antibody screening tests) at or after the study is unblinded will be offered poststudy HIV-1 diagnostic testing (per the HVTN poststudy HIV-1 testing algorithm) periodically and free of charge as medically/socially indicated (approximately every 6 months).

9.7 Contraception status

Contraception status is assessed and documented at every scheduled clinic visit for a participant who was born female and who is sexually active in a way that could cause that participant to become pregnant. Prior to enrollment and throughout the study, staff will ask participants to verbally confirm their use of adequate contraceptive methods. A participant who was born female and is sexually active in a way that could cause that participant to become pregnant should be reminded at all scheduled clinic visits of the importance of using contraception and should be referred to specific counseling, information, and advice as needed. (Specific contraception requirements are listed in Section 7.1.) This reminder should be documented in the participant's study record.

Self-reported infertility—including having reached menopause (no menses for 1 year) or having undergone hysterectomy, bilateral oophorectomy, or tubal ligation—must be documented in the participant's study record.

9.8 Urinalysis

Dipstick testing may be performed in the clinic or the lab, as long as the required elements (glucose, protein, and hemoglobin) are tested. The examination is performed on urine obtained by clean catch.

If the screening dipstick is transiently abnormal due to menses or infection, document this issue in the participant's source documentation. For infection, provide appropriate treatment and/or referral. Following resolution, repeat the dipstick and, if within the eligibility limits specified in the protocol, the participant may be enrolled.

Follow-up urinalysis should be deferred if a participant is menstruating, but should be performed as soon as possible. If a follow-up dipstick is abnormal due to a participant's menstrual period, document in the comment section of the case report form (CRF) and repeat the dipstick once the participant is no longer menstruating. In this case, a micro-urinalysis is not required.

9.9 Assessments of reactogenicity

For all participants, baseline assessments are performed before and reactogenicity assessments are performed after each vaccination. All reactogenicity symptoms are followed until resolution and graded according to the Division of AIDS Table for Grading the Severity of Adult and Pediatric Adverse Events (DAIDS AE Grading Table), Version 1.0, December 2004 (Clarification August 2009).

The reactogenicity assessment period is 3 full days following each vaccination per the assessment schedule shown in Table 9-1. Participants are instructed to record symptoms using a postvaccination symptom log and to contact the site daily during the assessment period. Clinic staff will follow new or unresolved reactogenicity symptoms present at day 3 to resolution. Participants are instructed to contact the clinic for events that arise during the period between vaccination and the next scheduled visit. In general, a participant who self-reports any postvaccination reaction greater than mild is seen by a clinician within 48 hours after onset, unless the reaction is improving and/or has completely resolved.

Reactogenicity events are reported using CRFs that correspond to the time of assessment in Table 9-1. Reactogenicity assessments include assessments of systemic and local symptoms, vaccine-related lesions, and lymph nodes. Events not listed on a CRF, or with an onset after the reactogenicity assessment period (day of vaccination and 3 full days after), or those meeting SAE/adverse events requiring expedited reporting to DAIDS criteria, are recorded on an adverse experience log form.

Table 9-1 Schedule of reactogenicity assessments

Day	Time	Performed by
0[a]	Baseline: before vaccination	HVTN CRS staff
	Early: 25-60 minutes after vaccination	HVTN CRS staff
	Between early assessment and 11:59pm day 0	HVTN CRS staff or participant
1	Between 12:00am and 11:59pm day 1	HVTN CRS staff or participant
2	Between 12:00am and 11:59pm day 2	HVTN CRS staff or participant
3[b]	Between 12:00am and 11:59pm day 3	HVTN CRS staff or participant

[a] Day of vaccination
[b] New or unresolved reactogenicity symptoms present on day 3 are followed until resolution

9.9.1 Assessment of systemic and local symptoms

Systemic symptoms include increased body temperature, malaise and/or fatigue, myalgia, headache, chills, arthralgia, nausea, and vomiting. Local symptoms include pain and/or tenderness proximal to the injection site. The daily maximum severity reached for each symptom during the assessment period is reported.

Body temperature is measured by oral or infrared thermometry and reported in degrees Celsius. If temperature is measured in Fahrenheit, the conversion to Celsius should be documented in the participant's chart note. A measurement is taken once daily during the assessment period and should be repeated if participant is feeling feverish.

9.9.2 Assessment of injection site

Typical injection site reactions are erythema/induration/swelling/edema. The maximum horizontal and maximum vertical measurements for all injection site reactions are recorded.

All injection site reactions are monitored until resolution. Areas greater than 25 cm^2 are followed daily; otherwise, the frequency of follow-up is based on clinician judgment.

9.9.3 Assessment of lymph nodes

This assessment is required only when reactogenicity assessments are performed by HVTN CRS staff, not by the participant.

Only the proximally draining lymph nodes are assessed (eg, axillary nodes on the same side of the body for injections given in the deltoid). Lymph nodes are first evaluated for enlargement and tenderness. If they are found to be enlarged, measurements are taken to determine the size (widest diameter) of the enlarged node(s).

9.10 Visit windows and missed visits

Visit windows are defined in HVTN 094 Study Specific Procedures. For a visit not performed within the window period, a Missed Visit form is completed. If the missed visit is one that required safety assessments or local safety labs, HVTN CRS staff should attempt to bring the participant in for an interim visit as soon as possible.

Procedures performed at an interim visit are usually toxicity/safety assessments (including local safety labs) and HIV testing. With the exception of HIV testing, these procedures are performed only if they were required at the missed visit or if clinically indicated. HIV testing may be performed as deemed appropriate by the study staff. Blood samples for immunogenicity assays are not typically collected at interim visits.

If a missed visit required vaccination, please refer to Sections 7.3.2 and 7.3.3 for resolution.

9.11 Early termination visit

In the event of early participant termination, site staff should consider if the following assessments are appropriate: a final physical examination, clinical laboratory tests (including urine dipstick, CBC with differential, platelet count, and chemistry panel), pregnancy testing, social impact assessment, and HIV test.

9.12 Pregnancy

If a participant becomes pregnant during the course of the study, no more injections of study product will be given, but remaining visits and study procedures should be completed unless medically contraindicated. If the participant terminates from the study prior to the pregnancy outcome, the site should make every effort to keep in touch with the participant in order to ascertain the pregnancy outcome.

10 Laboratory

10.1 HVTN CRS laboratory procedures

The HVTN Site Lab Reference Manual provides further guidelines for operational issues concerning the clinical and processing laboratories. The manual includes guidelines for general specimen collection, special considerations for phlebotomy; specimen labeling; whole blood processing; HIV screening/diagnostic testing; and general screening and safety testing.

Tube types for blood collection are specified in Appendix G, Appendix H, and Appendix I. For tests performed locally, the local lab may assign appropriate tube types.

In specific situations, the blood collection tubes will be redirected to another laboratory or will require study-specific processing techniques. In these cases, laboratory special instructions will be posted on the protocol-specific section of the HVTN website.

10.2 Total blood volume

Required blood volumes per visit differ for Groups 1, 2, and 3 and their respective blood draw schedules are shown in Appendix G, Appendix H, and Appendix I. The HVTN Laboratory Program will further specify the tube type and collection volumes in special instructions posted to the protocol-specific section of the HVTN website. Not shown is any additional blood volume that would be required if a safety lab needs to be repeated, or if a serum pregnancy test needs to be performed. The additional blood volume would likely be minimal. The total blood volume drawn for each participant will not exceed 500 mL in any 56-day (8-week) period.

10.3 Primary immunogenicity timepoint

The primary immunogenicity timepoint(s) in this study for Group 1 are Day 182 and Day 238 (ie, 2 weeks after the fourth and fifth vaccination visits); for Group 2 are Day 182 and Day 317 (ie, 2 weeks after the fourth and fifth vaccination visits); and for Group 3 is Day 238 (ie, 2 weeks after the fourth vaccination visit). Endpoint assays for humoral and cellular responses are performed on all participants at the primary immunogenicity timepoints and may be performed at baseline. Depending on the number of responders observed, assays for humoral and cellular responses may be performed on all participants at other timepoints; the schedule is shown in Appendix G, Appendix H, and Appendix I.

10.4 Endpoint assays: cellular

10.4.1 Flow cytometry

Flow cytometry will be used to examine vaccine-specific CD4+ and CD8+ T-cell responses following stimulation of PBMCs with synthetic HIV peptides that span the proteins encoded by the vaccine. ICS parameters will include cytokines such as IFN-γ, IL-2, and TNF-α, and may include other cytokines to identify T cells of specific

functionality (such as Th2 and Th17). Markers of cytotoxic potential (e.g., Granzyme B, and CD57, CD107a, MIP-1beta, CD154) may also be included. Data will be reported as percentages of CD4+ or CD8+ T cells responding to a specific peptide pool. Additional cell surface markers, cytokines, or functional markers may also be analyzed.

10.4.2 Cytokine multiplex bead array

Cryopreserved PBMCs will be stimulated with synthetic HIV peptides that span the proteins encoded by the vaccine. The multiplex assay will be used to measure concentrations of cytokines and functional markers present in the supernatant of the stimulated PBMCs (eg IFN-γ, IL-2, TNF-α, IL-4, IL-10, IL-6, IL-13, and IL-21). Other analytes may also be included.

10.5 Endpoint assays: humoral

10.5.1 HIV-1 multiplex antibody assay

Total binding IgG (IgG1, IgG2, IgG3, IgG4) and IgA antibodies to the immunodominant gp41 epitope, gp140 Env and Gag will be assessed on plasma samples from all study participants taken at the primary immunogenicity timepoints and baseline. Specimens from other timepoints as well as other HIV antigens may also be assayed based on the results of the initial assay. Data will be reported as concentration of Igs.

10.5.2 Neutralizing antibody assay

HIV-1–specific neutralizing antibody assays will be performed on serum samples from all study participants taken at the primary immunogenicity timepoints. Specimens from the baseline and other timepoints may also be analyzed. The tier 1 assays will test neutralization of the highly neutralization-sensitive tier 1 viruses (MN.3, SF162.LS, MP03.13, Bal.26 and MW965.26). Tier 2 assays may be conducted to test neutralization of a panel of primary subtype B isolates starting with an initial panel consisting of 6 subtype B strains chosen among the recommended reference strains [76]. If positive neutralization (ID_{50} titer >10, or >50% neutralization in a 1:10 screening assay) is detected greater than 10% of the time in vaccine recipients compared to placebo recipients, we will assay against an additional 6 tier 2 reference strains to increase the statistical power. Our benchmark is a conservative estimate based on the responses from a previous study [77] and may be modified as results are obtained on case control samples from more recent trials. Neutralization of additional isolates may be assessed.

10.5.3 Antibody avidity

Antibody avidity will be measured utilizing the binding antibody multiplex assay (BAMA) with the addition of a dissociation step to calculate the antibody avidity index. Antigen specific IgG avidity in each participant’s serum will be measured against a consensus Clade B gp41 immunodominant epitope. Avidity index will be expressed as the percent binding (MFI) in the treated well as compared to the non-treated well. Plasma samples collected from all study participants will be assessed at the primary immunogenicity timepoints and baseline. Specimens from other timepoints may also be assayed based on the results of the initial assay.

10.6 Innate immunity assays

As exploratory analyses, innate immunity studies will be performed on samples from Group 2 and Group 3 participants.

10.6.1 Soluble factors in serum

Multiplex and/or enzyme-linked immunosorbent assay (ELISA) may be used to measure soluble cytokines, chemokines, and other immunomodulatory factors in the serum. Analytes may include IFN-γ, IL-6, TNF- α, IL-10, IP-10, and/or MCP-1. Other analytes may also be included.

10.6.2 Enumeration and phenotyping of cell populations

Phenotyping of DCs, monocytes, NK cells, B cells, T cells or other leukocytes for lineage, maturation and activation markers may be performed on cryopreserved PBMC. Absolute cell counts will be determined on all participants by obtaining a CBC including a differential cell count from a clinical laboratory. Cryopreserved PBMC from the same blood draw will be examined by flow cytometry and absolute counts of cell types of interest will be determined based on the absolute lymphocyte count derived from the CBC. In addition, Trucount tubes with whole blood will be used when possible for direct enumeration of major leukocyte populations in the blood, including DC, by flow cytometry. Data will be reported as cell concentrations per microliter of blood and as percent of cells positive for each maker at the various timepoints.

10.6.3 RNA gene expression

Bulk PBMC will be cryopreserved in an RNA protection reagent. RNA may be isolated and used for microarray analysis and/or real-time PCR. Signatures of gene expression changes for different cell types will be analyzed over time after vaccination. Data will be reported as fold change over baseline expression.

10.7 Genotyping

Molecular human leukocyte antigen (HLA) typing may be performed on enrolled participants using cryopreserved PBMC collected at baseline, initially on specimens from participants who demonstrate vaccine-induced T-cell responses at postvaccination timepoints. Other participants (including placebo recipients) may be HLA-typed to support future studies of immunological interest at the discretion of the HVTN Laboratory Program. Other markers, such as genes associated with immune responses or HIV-1 may also be performed.

10.8 Exploratory studies

Samples may be used for other testing and research related to furthering the understanding of HIV immunology or vaccines. In addition, cryopreserved samples may be used to perform additional assays to support standardization and validation of existing or newly developed methods.

10.8.1 ADCC

As an exploratory analysis, the induction of antibodies capable of mediating ADCC may be investigated. The ADCC assay will use either gp120-coated cells or HIV-1 infected cells as targets (eg, CEM.NK.CCR5 cells) and either cryopreserved PBMC or a suitable cell line as effector cells. Percent specific ADCC activity in plasma will be based on cleavage of a cell-permeable fluorogenic peptide substrate containing a sequence recognizable by the serine protease Granzyme B (GzB). If reactivity is detected against the vaccine strain, then the breadth of responses may be evaluated with additional strains of either gp120 or infectious virus. Due to its exploratory nature, the performance of the ADCC assay will be at the discretion of the HVTN Laboratory Program; sample allocation for the ADCC assay has therefore been grouped with the serum storage specimens.

10.8.2 Anti-GM-CSF binding antibodies

As an exploratory analysis, serum GM-CSF antibody (GMAb) levels in each participant's serum may be determined by a GMAb sandwich ELISA. An authentic, GM-CSF affinity-purified GMAb isolated via affinity chromatography from serum of patients with autoimmune pulmonary alveolar proteinosis will be used as the standard. The GMAb levels will be measured by Rare Lung Disease Network Central Laboratory at Cincinnati Children's Hospital Research Foundation. Serum samples for this analysis will be collected at baseline, 2 weeks after the first vaccination visit, and 2 and 10 weeks after the second DNA vaccination visit.

10.8.3 GM-CSF levels

As an exploratory analysis, serum GM-CSF levels may be determined by a commercially-available ELISA, Quantikine HS GM-CSF Immunoassay, per manufacturer's instructions. Serum GM-CSF levels will be measured by Rare Lung Disease Network Central Laboratory at Cincinnati Children's Hospital Research Foundation. Serum samples for this analysis will be collected on the day of each DNA vaccination (prior to vaccination) as well as days 3 and 7 following each DNA vaccination for all study participants.

10.9 Ancillary studies

10.9.1 Antibody avidity studies

Additional antibody avidity assays may be performed by Duke University and GeoVax to conduct a comparison study of different antigens and avidity assay methods.

10.10 Other use of stored specimens

The HVTN aims not only to test vaccine candidates but also to continue to explore the correlates of immunity to HIV. In order to do so, the HVTN intends to store blood samples from participants. These samples will be used for other testing and research related to furthering the understanding of virology, immunology, or vaccinology to the extent authorized in each study site's informed consent form, or as otherwise authorized

under applicable law. Other testing on specimens will only occur, at a minimum, after review and approval by the HVTN and the IRB/EC of the researcher requesting the specimens.

The protocol sample informed consent form is written so that the participant either explicitly allows or does not allow sample storage for other research when he or she signs the form. Participants who initially agree to other use of their samples may rescind their approval once they enter the study; such participants will still remain in this study. If a participant decides against allowing other research using his or her samples, or at any time rescinds prior approval for such other use, the study site investigator or designee must notify HVTN Regulatory Affairs in writing. In either case, after database lock, the HVTN Laboratory Program will request that the repository destroy all specimens with the participant identification numbers (PTIDs) of all participants who do not agree to other use of their samples. HVTN Core will report the destruction of relevant specimens to the participants' site Principal Investigators (PIs).

Study sites must notify HVTN Regulatory Affairs if institutional or local governmental requirements pose a conflict with or impose restrictions on the use of stored specimens.

10.11 Biohazard containment

As the transmission of HIV and other blood-borne pathogens can occur through contact with contaminated needles, blood, and blood products, appropriate precautions will be employed by all personnel in the drawing of blood and shipping and handling of all specimens for this study, as currently recommended by the CDC and the NIH or other locally appropriate agencies.

All dangerous goods materials, including Biological Substances, Category A or Category B, must be transported according to instructions detailed in the International Air Transport Association Dangerous Goods Regulations.

11 Safety monitoring and safety review

11.1 Safety monitoring and oversight

11.1.1 HVTN 094 PSRT

The HVTN 094 PSRT is composed of the following members:

- DAIDS Medical Officer representative,
- Protocol Chair and Cochair,
- Protocol Team Leader,
- Core Medical Monitor, and
- SDMC Clinical Affairs Safety Associate.

The clinician members of HVTN 094 PSRT are responsible for decisions related to participant safety.

The Protocol Team clinic coordinator, project manager, vaccine developer representative, clinical trial manager, and others may also be included in HVTN 094 PSRT meetings.

11.1.2 HVTN SMB

The SMB is a multidisciplinary group consisting of biostatisticians, clinicians, and experts in HIV vaccine research that, collectively, has experience in the conduct and monitoring of vaccine trials. Members of the SMB are not directly affiliated with the protocols under review.

The SMB reviews safety data, unblinded as to treatment arm, approximately every 4 months. The reviews consist of evaluation of cumulative reactogenicity events, AE, laboratory safety data, and individual reports of adverse events requiring expedited reporting to DAIDS. To increase the sensitivity for detecting potential safety problems, the SMB will review safety data aggregated across multiple protocols that use the same or similar vaccine candidates. The SMB conducts additional special reviews at the request of the HVTN 094 PSRT.

Study sites will receive SMB summary minutes and are responsible for forwarding them to their local IRB/ECs.

11.1.3 SDMC roles and responsibilities in safety monitoring

The roles and responsibilities of the SDMC in relation to safety monitoring include:

- Maintaining a central database management system for HVTN clinical data;
- Providing reports of clinical data to appropriate groups such as the HVTN 094 PSRT and HVTN SMB (see Section 11.1.2);

- Daily monitoring of clinical data for events that meet the safety pause and HVTN 094 PSRT AE review criteria (see Section 11.3.1);

- Notifying HVTN CRSs and other groups when safety pauses or planned holds are instituted and lifted (see Section 11.3.1);

- Querying HVTN CRSs for additional information regarding reported clinical data; and

- Providing support to the HVTN 094 PSRT.

11.2 Safety reporting

11.2.1 Submission of safety forms to SDMC

Sites must submit all safety forms (eg, reactogenicity, adverse experience, urinalysis, local lab results, cardiac assessment, and concomitant medications) before the end of the next business day after receiving the information. The forms should not be held in anticipation of additional information at a later date. If additional information is received at a later date, the forms should be updated and refaxed before the end of the next business day after receiving the new information.

11.2.2 AE reporting

An AE is any untoward medical occurrence in a clinical investigation participant administered a study product/procedure(s) and which does not necessarily have a causal relationship with this treatment. An AE can therefore be any unfavorable and unintended sign (including an abnormal laboratory finding), symptom, or disease temporally associated with the use of an investigational study product/procedure(s), whether or not related to the investigational study product/procedure(s). All AEs are graded according to the Division of AIDS Table for Grading the Severity of Adult and Pediatric Adverse Events (DAIDS AE Grading Table), Version 1.0, December 2004 (Clarification dated August 2009), available on the RSC website at http://rsc.tech-res.com/safetyandpharmacovigilance/, except:

- unintentional weight loss of less than 10% loss in body weight from baseline is not required to be reported as an adverse event;

- PR interval ≤ 0.219 sec will not be reported as an AE; and

- asymptomatic increase in QTc interval < 0.06 sec above baseline, with a QTc ≤ 0.45 sec, will not be reported as an AE.

The definition of Grade 1 mild prolonged PR interval (Adult > 16 years) that will be used is:

- 0.22 - 0.25 sec.

The criteria for prolonged QTc interval that will be used are:

- Grade 1, mild: asymptomatic, QTc interval 0.45–0.47 sec;

- Grade 2, moderate: asymptomatic, QTc interval 0.48–0.49 sec;
- Grade 3 severe: asymptomatic, QTc interval ≥ 0.50 sec OR increase in interval ≥ 0.06 sec above baseline; and
- Grade 4, potentially life-threatening: life-threatening consequences (eg, Torsade de pointes or other associated serious ventricular dysrhythmia).

All AEs are reported to the SDMC on the appropriate CRF. Clinic staff should evaluate every AE to determine if (1) the AE meets the requirements for expedited reporting to DAIDS (Section 11.2.3) and (2) if the AE meets the criteria for a safety pause/prompt AE review (Section 11.3.1).

Autoimmune disorders are AEs of special interest (AESI). A sample list of AESI is provided in Appendix O. AESI's must be reported during the entire period of participation including during annual health contacts.

Sites are expected to notify SDMC Clinical Affairs staff of any serious safety concern requiring their attention (see Table 11-1). Telephone numbers and email addresses are listed in the Key Resource Guide of the HVTN 094 Study Specific Procedures. Concerns requiring immediate attention should be communicated by calling the SDMC Clinical Affairs safety phone.

In the case of email notification, SDMC Clinical Affairs staff will reply during working hours (US Pacific Time) to confirm that the email has been received and reviewed. If email service is not available, the HVTN CRS should notify SDMC Clinical Affairs of the event by telephone, then submit CRFs.

In addition, site investigators are required to submit AE information in accordance with local regulatory agencies' or other local authorities' requirements.

11.2.3 Expedited reporting of adverse events to DAIDS

Requirements, definitions and methods for expedited reporting of AEs are outlined in Version 2.0 (January 2010) of the *Manual for Expedited Reporting of Adverse Events* to DAIDS (DAIDS EAE Manual), which is available on the RSC website at http://rsc.tech-res.com/safetyandpharmacovigilance/. The SAE Reporting Category will be used for this study.

The internet-based DAIDS Adverse Event Reporting System (DAERS) must be used for expedited AE reporting to DAIDS. In the event of system outages or technical difficulties, expedited AE reports may be submitted via the DAIDS EAE Form. For questions about DAERS, please contact DAIDS-ES at DAIDS-ESSupport@niaid.nih.gov or from within the DAERS application itself.

Sites where DAERS has not been implemented will submit expedited AE reports by documenting the information on the current DAIDS EAE Form. This form is available on the RSC website: http://rsc.tech-res.com/safetyandpharmacovigilance/. For questions about expedited AE reporting, please contact the RSC (DAIDSRSCSafetyOffice@tech-res.com).

The study products for which expedited reporting are required are:

- GEO-D03 vaccine
- Placebo for GEO-D03
- MVA62B vaccine
- Placebo for MVA62B

While the participant is in the main study reporting period (See Section 3), the SAE Reporting Category will be used.

If the participant has completed the main study and is in the "annual health contacts reporting period" (see Section 3) the suspected unexpected serious adverse reactions (SUSAR) Reporting Category will be used. (In addition, please note, per Section 9.5, all adverse events that are serious are collected on the LTFU Event Log and reported to SCHARP.)

After the participant has completed the annual health contacts period and is off study, sites must report SUSARs if the study site staff becomes aware of the events on a passive basis (eg, from publicly available information).

The NIAID/DAIDS will report all unexpected SAEs related to the study products observed in this clinical trial to the FDA in accordance with 21 CFR 312.32 (IND Safety Reports). However, because safety is a primary study endpoint, the Sponsor Medical Officer will not be unblinded to study treatment assignment when there is an assessment of relatedness of the SAE with the study products; and the safety report will be sent to the FDA based on the blinded attribution assessment.

If the PSRT believes unblinding of the site PI to treatment assignment will assist with the clinical management of the SAE, the PSRT will consult the independent HVTN SMB for a recommendation. In the event the HVTN SMB determines that unblinding is indicated, the SMB will inform the site physician of the participant's treatment assignment in such a manner as to maintain the study blind of the PSRT and study team. For additional impact and management of SAEs on the study, refer to section 11.4.

11.3 Safety reviews

11.3.1 Initial safety evaluation and considerations for dose escalation

All enrollments and safety reviews for dose escalation occurred during Version 1.0 of the protocol

Enrollment will begin with Group 1 participants. The first six subjects across all sites will be enrolled at a rate of no more than one per day. Prior to enrolling any additional participants into Group 1, the HVTN 094 PSRT will review and approve the reactogenicity and safety data of the first 6 participants through day 14 after the initial GEO-D03 DNA injection. If the reactogenicity and safety data are deemed acceptable, Group 1 will continue to enroll until completion. Prior to beginning enrollment into Group 2, the HVTN 094 PSRT will review and approve the reactogenicity and safety data of all Group 1 participants through day 14 after the initial GEO-D03 DNA injection. If the reactogenicity and safety data are deemed acceptable, Group 2 may begin enrollment. The first six Group 2 subjects across all sites will be enrolled at a rate of no

more than one per day. Prior to enrolling any additional participants into Group 2, the HVTN 094 PSRT will review and approve the reactogenicity and safety data of the first 6 participants through day 14 after the initial GEO-D03 DNA injection. If the reactogenicity and safety data are deemed acceptable, Group 2 will continue to enroll until completion.

11.4 Safety pause and prompt PSRT AE review

When a trial is placed on safety pause, all enrollment and vaccination with the product related to the event that triggered the pause will be held until further notice. The AEs that will lead to a safety pause or prompt HVTN 094 PSRT AE review are summarized in Table 11-1. Vaccinations may be suspended for safety concerns other than those described in the table, or before pause rules are met, if, in the judgment of the HVTN 094 PSRT, participant safety may be threatened. Criteria for an individual participant's departure from the schedule of vaccinations are listed in Section 7.3.

Table 11-1 AE notification and safety pause/AE review rules

Event and relationship to study products/procedure	Severity	HVTN CRS action	SDMC action
SAE, related	Grade 5 or Grade 4	Phone immediately, email and fax forms immediately[a]	Immediate pause
SAE, not related	Grade 5	Phone immediately, email and fax forms immediately	Immediate HVTN 094 PSRT notification
SAE, related	Grade 3	Email and fax forms immediately	Prompt HVTN 094 PSRT AE review to consider pause
AE[b], related	Grade 4 or 3	Email and fax forms immediately	Prompt HVTN 094 PSRT AE review to consider pause

[a] Phone numbers and email addresses are listed in HVTN 094 Study Specific Procedures, Key Resource Guide.
[b] Does not include subjective reactogenicity symptoms (injection site pain, tenderness, fatigue/malaise, myalgia, arthralgia, chills, headache, nausea).

For all safety pauses, the SDMC Clinical Affairs staff notifies the HVTN 094 PSRT, HVTN Regulatory Affairs, DAIDS Pharmaceutical Affairs Branch (PAB), DAIDS Regulatory Affairs Branch (RAB), DAIDS Safety and Pharmacovigilance Team (SPT), and participating HVTN CRSs. When an immediate safety pause is triggered, the SDMC Clinical Affairs staff also notifies the HVTN SMB.

Once a trial is paused, the HVTN 094 PSRT reviews safety data and decides whether the pause can be lifted or permanent discontinuation of vaccination is appropriate, consulting the SMB if necessary. SDMC Clinical Affairs staff notifies the participating HVTN CRSs, HVTN Regulatory Affairs, DAIDS PAB, DAIDS RAB, and DAIDS SPT of the decision regarding resumption or discontinuation of study vaccinations. Based on the HVTN 094 PSRT assessment, DAIDS RAB notifies the FDA as needed.

If an immediate HVTN 094 PSRT notification or prompt HVTN 094 PSRT AE review is triggered, the SDMC Clinical Affairs staff notifies the HVTN 094 PSRT as soon as possible during working hours (US Pacific Time)—or, if the information was received during off hours, by the morning of the next work day. If a prompt HVTN 094 PSRT AE review cannot be completed within 72 hours of SDMC notification (excluding weekends and US federal holidays), an automatic safety pause occurs.

Each HVTN CRS is responsible for submitting to its IRB/EC and any local regulatory authority protocol-related safety information (such as IND safety reports, notification of vaccine holds due to the pause rules, etc), as required.

In addition, all other AEs are reviewed routinely by the HVTN 094 PSRT (see Section 11.5.2).

11.5 Review of cumulative safety data

Routine safety review occurs at the start of enrollment and then throughout the study.

Reviews proceed from a standardized set of protocol-specific safety data reports. These reports are produced by the SDMC and include queries to the HVTN CRSs. Events are tracked by internal reports until resolution.

11.5.1 Daily review

Blinded daily safety reviews are routinely conducted by the SDMC Clinical Affairs staff for events requiring expedited reporting to DAIDS, and events that meet safety pause criteria or prompt HVTN 094 PSRT AE review criteria.

11.5.2 Weekly review

During the injection phase of the trial, the SDMC Clinical Affairs staff and the HVTN 094 PSRT review clinical safety reports on a weekly basis and conduct calls to review the data as appropriate. After the injections and the final 2-week safety visits are completed, less frequent reporting and safety reviews may be conducted at the discretion of the HVTN 094 PSRT. The SDMC Clinical Affairs staff reviews reports of clinical and laboratory AEs. Events identified during the review that are considered questionable, inconsistent, or unexplained are referred to the HVTN CRS clinic coordinator for verification.

11.6 Study termination

This study may be terminated early by the determination of the HVTN 094 PSRT, HVTN SMB, FDA, NIH, Office for Human Research Protections (OHRP), or vaccine developer. In addition, the conduct of this study at an individual HVTN CRS may be terminated by the determination of the local IRB or EC, or of the appropriate local or national regulatory authority.

12 Protocol conduct

This protocol and all actions and activities connected with it will be conducted in compliance with the principles of GCP (ICHe6), and according to DAIDS and HVTN policies and procedures as specified in the *HVTN Manual of Operations,* DAIDS Clinical Research Policies and Standard Procedures Documents including procedures for the following:

- Protocol registration, activation, and implementation;
- Informed consent, screening, and enrollment;
- Study participant reimbursement;
- Clinical and safety assessments;
- Safety monitoring and reporting;
- Data collection, documentation, transfer, and storage;
- Participant confidentiality;
- Study follow-up and close-out;
- Unblinding of staff and participants;
- Quality control;
- Protocol monitoring and compliance;
- Advocacy and assistance to participants regarding negative social impacts associated with the vaccine trial;
- Risk reduction counseling; and
- Specimen collection, processing, and analysis.

Any policies or procedures that vary from DAIDS and HVTN standards or require additional instructions (eg, instructions for randomization specific to this study) will be described in the HVTN 094 *Study Specific Procedures.*

12.1 Social impacts

Participants in this study risk experiencing discrimination or other personal problems, resulting from the study participation itself or from the development of VISP. The HVTN CRS is obliged to provide advocacy for and assistance to participants regarding these negative social impacts associated with the vaccine trial. If HVTN CRS staff have questions regarding ways to assist a participant dealing with a social impact, a designated NIAID or HVTN Core representative can be contacted.

Social harms are tabulated by the SDMC and are subjected to descriptive analysis. The goal is to reduce their incidence and enhance the ability of study staff to mitigate them when possible.

Summary tables of social impact events will be generated weekly, and made available for review by the protocol chairs, protocol team leader, and the designated NIAID representative.

12.2 Compliance with NIH guidelines for research involving products containing recombinant DNA

Because this study is evaluating products containing recombinant DNA, it must comply with regulations set forth in the NIH's *Guidelines for Research Involving Recombinant DNA Molecules.* Information about the study must be submitted to site Institutional Biosafety Committees (IBC) and must be approved before participants are enrolled at the respective institution. Investigators at each site are responsible for obtaining IBC approval and periodic review of the research per NIH guidelines *section IV-B07-b-(6)* and *section IV-B-2-b.* IBC review and approval must be documented by the investigator and submitted as part of initial protocol registration for this trial. If this protocol is amended, investigators should follow the requirements of their IBC.

The NIH guidelines also require that human gene transfer trials conducted at or sponsored by institutions that receive NIH funds must be submitted to the NIH Office of Biotechnology Activities (OBA) for review by the Recombinant DNA Advisory Committee (RAC). The NIH guidelines create exceptions to RAC review, but the HVTN 094 Protocol Team determined that the exceptions did not apply. Therefore, the Protocol Team, jointly with GeoVax, Inc., submitted the application with the study concept proposal for RAC review and responded to RAC comments. The application will follow the guidance provided in the NIH Guidelines.

The HVTN and DAIDS will ensure that reporting requirements to RAC, as outlined in *Appendix M-I-C-1.Initiation of the Clinical Investigation*, *Appendix M-I-C-3.Annual Reports*, and *Appendix M-I-C-4.Safety Reporting* are satisfied per the NIH Guidelines.

12.3 Emergency communication with study participants

As in all clinical research, this study may generate a need to reach participants quickly to avoid imminent harm, or to report study findings that may otherwise concern their health or welfare.

When such communication is needed, the CRS will request that its IRB/EC expedite review of the message. If this review cannot be completed in a timeframe consistent with the urgency of the required communication, the site should contact the participant first, and then notify the IRB/EC of the matter as soon as possible.

13 Version history

The Protocol Team may modify the original version of the protocol. Modifications are made to HVTN protocols via clarification memos, letters of amendment, or full protocol amendments.

The table below describes the version history of, and modifications to, Protocol HVTN 094.

Protocol history and modifications

Date	Protocol version	Protocol modification	Comment
7-JAN-2013	Version 2	Full Protocol Amendment	
13-JUN-2012	Version 1	Clarification Memo3	
1-MAR-2012	Version 1	Clarification Memo 2	
7-FEB-2012	Version 1	Clarification Memo 1	
20-DEC-2011	Version 1	Original protocol	

14 Document references (other than literature citations)

Other documents referred to in this protocol, and containing information relevant to the conduct of this study, include:

- Assessment of Understanding. Accessible through the HVTN protocol-specific website.

- Current CDC Guidelines. Revised Recommendations for HIV Testing of Adults, Adolescents, and Pregnant Women in Health-Care Settings. Available at http://www.cdc.gov/mmwr/PDF/rr/rr5514.pdf.

- Division of AIDS (DAIDS) Clinical Research Policies and Standard Procedures Documents. Available at http://www3.niaid.nih.gov/research/resources/DAIDSClinRsrch/

- Division of AIDS Protocol Registration Manual. Available at http://www.niaid.nih.gov/LabsAndResources/resources/DAIDSClinRsrch/Documents/prmanual.pdf

- Division of AIDS Table for Grading the Severity of Adult and Pediatric Adverse Events. Version 1.0, December 2004. (Clarification dated August 2009) Available at http://rsc.tech-res.com/safetyandpharmacovigilance

- The Manual for Expedited Reporting of Adverse Events to DAIDS. Version 2.0, January 2010. Available at http://rsc.tech-res.com/safetyandpharmacovigilance HVTN Certificate of Confidentiality. Accessible through the HVTN website.

- HVTN 094 Special Instructions. Accessible through the HVTN protocol-specific website.

- HVTN 094 Study Specific Procedures. Accessible through the HVTN protocol-specific website.

- HVTN Site Lab Reference Manual. Accessible through the HVTN website.

- HVTN Manual of Operations. Accessible through the HVTN website.

- HVTN algorithm for diagnosis of HIV infections. Part of the HVTN Site Lab Reference Manual (see above).

- International Conference on Harmonisation (ICH) E6 (R1), Guideline for Good Clinical Practice: Section 4.8, Informed consent of trial subjects. Available at http://www.emea.europa.eu/pdfs/human/ich/013595en.pdf.

- Participants' Bill of Rights and Responsibilities. Accessible through the HVTN website.

- NIH Guidelines for Research Involving Recombinant DNA Molecules. Available at http://oba.od.nih.gov/rdna/nih_guidelines_oba.html.

- NIH Policy on Reporting Race and Ethnicity Data: Subjects in Clinical Research. Available at http://grants1.nih.gov/grants/guide/notice-files/NOT-OD-01-053.html.

- Pharmacy Guidelines and Instructions for DAIDS Clinical Trials Networks, July 2008.

- Requirements for Source Documentation in DAIDS Funded and/or Sponsored Clinical Trials. Available at http://www3.niaid.nih.gov/research/resources/DAIDSClinRsrch/ClinicalSite.htm

- Title 21, Code of Federal Regulations, Part 50. Available at http://www.access.gpo.gov/nara/cfr/waisidx_08/21cfrv1_08.html.

- Title 45, Code of Federal Regulations, Part 46. Available at http://www.access.gpo.gov/nara/cfr/waisidx_07/45cfrv1_07.html.

See section 16 for literature cited in the background and statistics sections of this protocol.

15 Acronyms and abbreviations

Ab	antibody
Ad	adenovirus
AE	adverse event
AESI	AEs of special interest
ALT	alanine aminotransferase
ANOVA	analysis of variance
ART	antiretroviral therapy
AST	aspartate aminotransferase
AVEG	AIDS Vaccine Evaluation Group
β-HCG	beta human chorionic gonadotropin
BMI	body mass index
CAB	Community Advisory Board
CBC	complete blood count
CCHRF	Cincinnati Children's Hospital Research Foundation
CDC	US Centers for Disease Control and Prevention
CFR	Code of Federal Regulations
CIOMS	Council for International Organizations of Medical Sciences
CI	confidence intervals
CRF	case report form
CRPMC	NIAID Clinical Research Products Management Center
CRS*	clinical research site
CTL	cytotoxic T lymphocyte
DAERS	DAIDS Adverse Event Reporting System
DAIDS	Division of AIDS (US NIH)
DHHS	US Department of Health and Human Services
DSMB	NIAID Data and Safety Monitoring Board
EAE	adverse events requiring expedited reporting to DAIDS
EC	Ethics Committee
EIA	enzyme immunoassay
ELISA	enzyme-linked immunosorbent assay
ELISpot	enzyme-linked immunospot
FDA	US Food and Drug Administration
FHCRC	Fred Hutchinson Cancer Research Center
FPR	false positive rate
GCP	Good Clinical Practice
GEE	generalized estimating equation
HAART	highly active antiretroviral therapy
HBsAg	hepatitis B surface antigen
HCV	hepatitis C virus
HIPAA	Health Insurance Portability and Accountability Act
HIV	human immunodeficiency virus

HLA	human leukocyte antigen
HVTN	HIV Vaccine Trials Network
IB	Investigator's Brochure
IBC	Institutional Biosafety Committee
ICH	International Conference on Harmonisation
ICS	intracellular cytokine staining
IFN-γ	interferon gamma
IND	Investigational New Drug
IRB	Institutional Review Board
IUD	intrauterine device
LTFU	loss to follow-up
MAR	missing at random
MCAR	missing completely at random
MFI	mean fluorescence intensity
MMR	measles, mumps, and rubella
MVA	modified vaccinia Ankara
nAb	neutralizing antibody
NHP	nonhuman primate
NIAID	National Institute of Allergy and Infectious Diseases (US NIH)
NICD	National Institute for Communicable Diseases (Johannesburg, South Africa)
NIH	US National Institutes of Health
OBA	NIH Office of Biotechnology Activities
OHRP	US Office for Human Research Protections
OPV	oral polio vaccine
PAB	DAIDS Pharmaceutical Affairs Branch
PBMC	peripheral blood mononuclear cell
PBS	phosphate-buffered saline
PCR	polymerase chain reaction
PI	Principal Investigator
PSRT	Protocol Safety Review Team
PTE	potential T-cell epitope
RAB	DAIDS Regulatory Affairs Branch
RAC	NIH Recombinant DNA Advisory Committee
RSC	DAIDS Regulatory Support Center
SAE	serious adverse event
SCHARP	Statistical Center for HIV/AIDS Research and Prevention
SDMC	statistical and data management center
SFC	spot-forming cell
SFU	spot-forming unit
SIV	simian immunodeficiency virus
SMB	Safety Monitoring Board
SPT	DAIDS Safety and Pharmacovigilance Team
SUSAR	suspected unexpected serious adverse reactions

TB	tuberculosis
UW-VSL	University of Washington Virology Specialty Laboratory
VISP	Vaccine induced seropositivity
VRC	Vaccine Research Center (NIAID)
vs.	versus
WBC	white blood cell

* CRSs were formerly referred to as HIV Vaccine Trial Units (HVTUs). Conversion to use of the term CRS is in process, and some HVTN documents may still refer to HVTUs.

16 Literature cited

1. Gorse GJ, Schwartz DH, Graham BS, Matthews TJ, Stablein DM, Frey SE, Belshe RB, Clements ML, Wright PF, Eibl M, . HIV-1 recombinant gp160 vaccine given in accelerated dose schedules. NIAID AIDS Vaccine Clinical Trials Network. Clin Exp Immunol **1994**;98:178-84.

2. Graham BS, Keefer MC, McElrath MJ, Gorse GJ, Schwartz DH, Weinhold K, Matthews TJ, Esterlitz JR, Sinangil F, Fast PE. Safety and immunogenicity of a candidate HIV-1 vaccine in healthy adults: recombinant glycoprotein (rgp) 120. A randomized, double-blind trial. NIAID AIDS Vaccine Evaluation Group. Ann Intern Med **1996**;125:270-9.

3. Rose MA, Gruendler M, Schubert R, Kitz R, Schulze J, Zielen S. Safety and immunogenicity of sequential pneumococcal immunization in preschool asthmatics. Vaccine **2009**;27:5259-64.

4. UNAIDS. Ethical considerations in biomedical HIV prevention trials. **2007**.

5. The National Commission for the Protection of Human Subjects of Biomedical and Behavioral Research. The Belmont Report: Ethical Principles and Guidelines for the Protection of Human Subjects of Research. **1979**.

6. Council for International Organizations of Medical Sciences (CIOMS). International ethical guidelines for biomedical research involving human subjects. Bull Med Ethics **2002**;17-23.

7. Goepfert PA, Elizaga ML, Sato A, Qin L, Cardinali M, Hay CM, Hural J, Derosa SC, Defawe OD, Tomaras GD, Montefiori DC, Xu Y, Lai L, Kalams SA, Baden LR, Frey SE, Blattner WA, Wyatt LS, Moss B, Robinson HL. Phase 1 Safety and Immunogenicity Testing of DNA and Recombinant Modified Vaccinia Ankara Vaccines Expressing HIV-1 Virus-like Particles. J Infect Dis **2011**;203:610-9.

8. Rissoan MC, Soumelis V, Kadowaki N, Grouard G, Briere F, de Waal MR, Liu YJ. Reciprocal control of T helper cell and dendritic cell differentiation. Science **1999**;283:1183-6.

9. Kopf M, Herren S, Wiles MV, Pepys MB, Kosco-Vilbois MH. Interleukin 6 influences germinal center development and antibody production via a contribution of C3 complement component. J Exp Med **1998**;188:1895-906.

10. Burdin N, Galibert L, Garrone P, Durand I, Banchereau J, Rousset F. Inability to produce IL-6 is a functional feature of human germinal center B lymphocytes. J Immunol **1996**;156:4107-13.

11. Xiao P, Zhao J, Patterson LJ, Brocca-Cofano E, Venzon D, Kozlowski PA, Hidajat R, Demberg T, Robert-Guroff M. Multiple vaccine-elicited nonneutralizing antienvelope antibody activities contribute to protective

efficacy by reducing both acute and chronic viremia following simian/human immunodeficiency virus SHIV89.6P challenge in rhesus macaques. J Virol **2010**;84:7161-73.

12. Zhou T, Georgiev I, Wu X, Yang ZY, Dai K, Finzi A, Kwon YD, Scheid JF, Shi W, Xu L, Yang Y, Zhu J, Nussenzweig MC, Sodroski J, Shapiro L, Nabel GJ, Mascola JR, Kwong PD. Structural basis for broad and potent neutralization of HIV-1 by antibody VRC01. Science **2010**;329:811-7.

13. Lai L, Kwa S, Kozlowski PA, Montefiori DC, Ferrari G, Johnson WE, Hirsch V, Villinger F, Chennareddi L, Earl PL, Moss B, Amara RR, Robinson HL. Prevention of infection by a granulocyte-macrophage colony-stimulating factor co-expressing DNA/modified vaccinia Ankara simian immunodeficiency virus vaccine. J Infect Dis **2011**;204:164-73.

14. Yokota A, Takeuchi H, Maeda N, Ohoka Y, Kato C, Song SY, Iwata M. GM-CSF and IL-4 synergistically trigger dendritic cells to acquire retinoic acid-producing capacity. Int Immunol **2009**;21:361-77.

15. Iwata M, Hirakiyama A, Eshima Y, Kagechika H, Kato C, Song SY. Retinoic acid imprints gut-homing specificity on T cells. Immunity **2004**;21:527-38.

16. Mora JR, Iwata M, Eksteen B, Song SY, Junt T, Senman B, Otipoby KL, Yokota A, Takeuchi H, Ricciardi-Castagnoli P, Rajewsky K, Adams DH, von Andrian UH. Generation of gut-homing IgA-secreting B cells by intestinal dendritic cells. Science **2006**;314:1157-60.

17. Faith A, McDonald J, Peek E, Richards D, Caulfield J, Chevretton E, Roberts D, Lee T, Corrigan C, Hawrylowicz C. Functional plasticity of human respiratory tract dendritic cells: GM-CSF enhances T(H)2 development. J Allergy Clin Immunol **2005**;116:1136-43.

18. Stumbles PA, Thomas JA, Pimm CL, Lee PT, Venaille TJ, Proksch S, Holt PG. Resting respiratory tract dendritic cells preferentially stimulate T helper cell type 2 (Th2) responses and require obligatory cytokine signals for induction of Th1 immunity. J Exp Med **1998**;188:2019-31.

19. Bonecchi R, Bianchi G, Bordignon PP, D'Ambrosio D, Lang R, Borsatti A, Sozzani S, Allavena P, Gray PA, Mantovani A, Sinigaglia F. Differential expression of chemokine receptors and chemotactic responsiveness of type 1 T helper cells (Th1s) and Th2s. J Exp Med **1998**;187:129-34.

20. Sallusto F, Palermo B, Hoy A, Lanzavecchia A. The role of chemokine receptors in directing traffic of naive, type 1 and type 2 T cells. Curr Top Microbiol Immunol **1999**;246:123-8.

21. Douek DC, Brenchley JM, Betts MR, Ambrozak DR, Hill BJ, Okamoto Y, Casazza JP, Kuruppu J, Kunstman K, Wolinsky S, Grossman Z, Dybul M, Oxenius A, Price DA, Connors M, Koup RA. HIV preferentially infects HIV-specific CD4+ T cells. Nature **2002**;417:95-8.

22. Filipazzi P, Valenti R, Huber V, Pilla L, Canese P, Iero M, Castelli C, Mariani L, Parmiani G, Rivoltini L. Identification of a new subset of myeloid suppressor cells in peripheral blood of melanoma patients with modulation by a granulocyte-macrophage colony-stimulation factor-based antitumor vaccine. J Clin Oncol **2007**;25:2546-53.

23. Lai L, Vodros D, Kozlowski PA, Montefiori DC, Wilson RL, Akerstrom VL, Chennareddi L, Yu T, Kannanganat S, Ofielu L, Villinger F, Wyatt LS, Moss B, Amara RR, Robinson HL. GM-CSF DNA: An adjuvant for higher avidity IgG, rectal IgA, and increased protection against the acute phase of a SHIV-89.6P challenge by a DNA/MVA immunodeficiency virus vaccine. Virology **2007**;369:153-67.

24. Zhao J, Lai L, Amara RR, Montefiori DC, Villinger F, Chennareddi L, Wyatt LS, Moss B, Robinson HL. Preclinical studies of human immunodeficiency virus/AIDS vaccines: inverse correlation between avidity of anti-Env antibodies and peak postchallenge viremia. J Virol **2009**;83:4102-11.

25. Pantaleo G, Esteban M, Jacobs B, Tartaglia J. Poxvirus vector-based HIV vaccines. Curr Opin HIV AIDS **2010**;5:391-6.

26. Gomez CE, Najera JL, Jimenez V, Bieler K, Wild J, Kostic L, Heidari S, Chen M, Frachette MJ, Pantaleo G, Wolf H, Liljestrom P, Wagner R, Esteban M. Generation and immunogenicity of novel HIV/AIDS vaccine candidates targeting HIV-1 Env/Gag-Pol-Nef antigens of clade C. Vaccine **2007**;25:1969-92.

27. Harenberg A, Guillaume F, Ryan EJ, Burdin N, Spada F. Gene profiling analysis of ALVAC infected human monocyte derived dendritic cells. Vaccine **2008**;26:5004-13.

28. Mooij P, Balla-Jhagjhoorsingh SS, Koopman G, Beenhakker N, van Haaften P, Baak I, Nieuwenhuis IG, Kondova I, Wagner R, Wolf H, Gomez CE, Najera JL, Jimenez V, Esteban M, Heeney JL. Differential CD4+ versus CD8+ T-cell responses elicited by different poxvirus-based human immunodeficiency virus type 1 vaccine candidates provide comparable efficacies in primates. J Virol **2008**;82:2975-88.

29. Delaloye J, Roger T, Steiner-Tardivel QG, Le Roy D, Knaup RM, Akira S, Petrilli V, Gomez CE, Perdiguero B, Tschopp J, Pantaleo G, Esteban M, Calandra T. Innate immune sensing of modified vaccinia virus Ankara (MVA) is mediated by TLR2-TLR6, MDA-5 and the NALP3 inflammasome. PLoS Pathog **2009**;5:e1000480.

30. Rerks-Ngarm S, Pitisuttithum P, Nitayaphan S, Kaewkungwal J, Chiu J, Paris R, Premsri N, Namwat C, de Souza M, Adams E, Benenson M, Gurunathan S, Tartaglia J, McNeil JG, Francis DP, Stablein D, Birx DL, Chunsuttiwat S, Khamboonruang C, Thongcharoen P, Robb ML, Michael NL, Kunasol P, Kim JH. Vaccination with ALVAC and AIDSVAX to Prevent HIV-1 Infection in Thailand. N Engl J Med **2009**.

31. Harari A, Bart PA, Stohr W, Tapia G, Garcia M, Medjitna-Rais E, Burnet S, Cellerai C, Erlwein O, Barber T, Moog C, Liljestrom P, Wagner R, Wolf

H, Kraehenbuhl JP, Esteban M, Heeney J, Frachette MJ, Tartaglia J, McCormack S, Babiker A, Weber J, Pantaleo G. An HIV-1 clade C DNA prime, NYVAC boost vaccine regimen induces reliable, polyfunctional, and long-lasting T cell responses. J Exp Med **2008**;205:63-77.

32. Barouch DH, O'Brien KL, Simmons NL, King SL, Abbink P, Maxfield LF, Sun YH, La Porte A, Riggs AM, Lynch DM, Clark SL, Backus K, Perry JR, Seaman MS, Carville A, Mansfield KG, Szinger JJ, Fischer W, Muldoon M, Korber B. Mosaic HIV-1 vaccines expand the breadth and depth of cellular immune responses in rhesus monkeys. Nat Med **2010**;16:319-23.

33. Smith JM, Amara RR, Campbell D, Xu Y, Patel M, Sharma S, Butera ST, Ellenberger DL, Yi H, Chennareddi L, Herndon JG, Wyatt LS, Montefiori D, Moss B, McClure HM, Robinson HL. DNA/MVA vaccine for HIV type 1: effects of codon-optimization and the expression of aggregates or virus-like particles on the immunogenicity of the DNA prime. AIDS Res Hum Retroviruses **2004**;20:1335-47.

34. Wyatt LS, Earl PL, Vogt J, Eller LA, Chandran D, Liu J, Robinson HL, Moss B. Correlation of immunogenicities and in vitro expression levels of recombinant modified vaccinia virus Ankara HIV vaccines. Vaccine **2008**;26:486-93.

35. Letvin NL, Huang Y, Chakrabarti BK, Xu L, Seaman MS, Beaudry K, Korioth-Schmitz B, Yu F, Rohne D, Martin KL, Miura A, Kong WP, Yang ZY, Gelman RS, Golubeva OG, Montefiori DC, Mascola JR, Nabel GJ. Heterologous envelope immunogens contribute to AIDS vaccine protection in rhesus monkeys. J Virol **2004**;78:7490-7.

36. Amara RR, Patel K, Niedziela G, Nigam P, Sharma S, Staprans SI, Montefiori DC, Chenareddi L, Herndon JG, Robinson HL, McClure HM, Novembre FJ. A combination DNA and attenuated simian immunodeficiency virus vaccine strategy provides enhanced protection from simian/human immunodeficiency virus-induced disease. J Virol **2005**;79:15356-67.

37. Huber M, Trkola A. Humoral immunity to HIV-1: neutralization and beyond. J Intern Med **2007**;262:5-25.

38. Aasa-Chapman MM, Holuigue S, Aubin K, Wong M, Jones NA, Cornforth D, Pellegrino P, Newton P, Williams I, Borrow P, McKnight A. Detection of antibody-dependent complement-mediated inactivation of both autologous and heterologous virus in primary human immunodeficiency virus type 1 infection. J Virol **2005**;79:2823-30.

39. Sullivan BL, Knopoff EJ, Saifuddin M, Takefman DM, Saarloos MN, Sha BE, Spear GT. Susceptibility of HIV-1 plasma virus to complement-mediated lysis. Evidence for a role in clearance of virus in vivo. J Immunol **1996**;157:1791-8.

40. Ahmad A, Menezes J. Antibody-dependent cellular cytotoxicity in HIV infections. FASEB J **1996**;10:258-66.

41. Blumberg RS, Paradis T, Hartshorn KL, Vogt M, Ho DD, Hirsch MS, Leban J, Sato VL, Schooley RT. Antibody-dependent cell-mediated cytotoxicity against cells infected with the human immunodeficiency virus. J Infect Dis **1987**;156:878-84.

42. Shepp DH, Chakrabarti S, Moss B, Quinnan GV, Jr. Antibody-dependent cellular cytotoxicity specific for the envelope antigens of human immunodeficiency virus. J Infect Dis **1988**;157:1260-4.

43. Forthal DN, Landucci G, Keenan B. Relationship between antibody-dependent cellular cytotoxicity, plasma HIV type 1 RNA, and CD4+ lymphocyte count. AIDS Res Hum Retroviruses **2001**;17:553-61.

44. Kannanganat S, Nigam P, Velu V, Earl PL, Lai L, Chennareddi L, Lawson B, Wilson RL, Montefiori DC, Kozlowski PA, Moss B, Robinson HL, Amara RR. Preexisting vaccinia virus immunity decreases SIV-specific cellular immunity but does not diminish humoral immunity and efficacy of a DNA/MVA vaccine. J Immunol **2010**;185:7262-73.

45. Schlesinger Y, Granoff DM. Avidity and bactericidal activity of antibody elicited by different Haemophilus influenzae type b conjugate vaccines. The Vaccine Study Group. JAMA **1992**;267:1489-94.

46. Anttila M, Eskola J, Ahman H, Kayhty H. Avidity of IgG for Streptococcus pneumoniae type 6B and 23F polysaccharides in infants primed with pneumococcal conjugates and boosted with polysaccharide or conjugate vaccines. J Infect Dis **1998**;177:1614-21.

47. Parekh BS, Pau CP, Kennedy MS, Dobbs TL, McDougal JS. Assessment of antibody assays for identifying and distinguishing recent from long-term HIV type 1 infection. AIDS Res Hum Retroviruses **2001**;17:137-46.

48. von Bubnoff A. 2G12 revisisted. IAVI Research Report **2009**;13:21-2.

49. Hessell AJ, Rakasz EG, Poignard P, Hangartner L, Landucci G, Forthal DN, Koff WC, Watkins DI, Burton DR. Broadly neutralizing human anti-HIV antibody 2G12 is effective in protection against mucosal SHIV challenge even at low serum neutralizing titers. PLoS Pathog **2009**;5:e1000433.

50. Mascola JR, Stiegler G, VanCott TC, Katinger H, Carpenter CB, Hanson CE, Beary H, Hayes D, Frankel SS, Birx DL, Lewis MG. Protection of macaques against vaginal transmission of a pathogenic HIV-1/SIV chimeric virus by passive infusion of neutralizing antibodies. Nat Med **2000**;6:207-10.

51. Ledgerwood JE, Wei CJ, Hu Z, Gordon IJ, Enama ME, Hendel CS, McTamney PM, Pearce MB, Yassine HM, Boyington JC, Bailer R, Tumpey TM, Koup RA, Mascola JR, Nabel GJ, Graham BS. DNA priming and influenza vaccine immunogenicity: two phase 1 open label randomised clinical trials. Lancet Infect Dis **2011**;11:916-24.

52. Belshe RB, Frey SE, Graham I, Mulligan MJ, Edupuganti S, Jackson LA, Wald A, Poland G, Jacobson R, Keyserling HL, Spearman P, Hill H, Wolff M.

Safety and immunogenicity of influenza A H5 subunit vaccines: effect of vaccine schedule and antigenic variant. J Infect Dis **2011**;203:666-73.

53. Haynes BF, Gilbert PB, McElrath MJ, Zolla-Pazner S, Tomaras GD, Alam SM, Evans DT, Montefiori DC, Karnasuta C, Sutthent R, Liao HX, DeVico AL, Lewis GK, Williams C, Pinter A, Fong Y, Janes H, DeCamp A, Huang Y, Rao M, Billings E, Karasavvas N, Robb ML, Ngauy V, de Souza MS, Paris R, Ferrari G, Bailer RT, Soderberg KA, Andrews C, Berman PW, Frahm N, De Rosa SC, Alpert MD, Yates NL, Shen X, Koup RA, Pitisuttithum P, Kaewkungwal J, Nitayaphan S, Rerks-Ngarm S, Michael NL, Kim JH. Immune-correlates analysis of an HIV-1 vaccine efficacy trial. N Engl J Med **2012**;366:1275-86.

54. Reynolds MR, Weiler AM, Weisgrau KL, Piaskowski SM, Furlott JR, Weinfurter JT, Kaizu M, Soma T, Leon EJ, MacNair C, Leaman DP, Zwick MB, Gostick E, Musani SK, Price DA, Friedrich TC, Rakasz EG, Wilson NA, McDermott AB, Boyle R, Allison DB, Burton DR, Koff WC, Watkins DI. Macaques vaccinated with live-attenuated SIV control replication of heterologous virus. J Exp Med **2008**;205:2537-50.

55. Yeh WW, Jaru-Ampornpan P, Nevidomskyte D, Asmal M, Rao SS, Buzby AP, Montefiori DC, Korber BT, Letvin NL. Partial protection of Simian immunodeficiency virus (SIV)-infected rhesus monkeys against superinfection with a heterologous SIV isolate. J Virol **2009**;83:2686-96.

56. Robinson HL, Montefiori DC, Villinger F, Robinson JE, Sharma S, Wyatt LS, Earl PL, McClure HM, Moss B, Amara RR. Studies on GM-CSF DNA as an adjuvant for neutralizing Ab elicited by a DNA/MVA immunodeficiency virus vaccine. Virology **2006**;352:285-94.

57. Seaman M, Janes H, Hawkins N, randpre L, Devoy C, Giri A, Coffey R, Harris L, Wood B, Daniels M, Bhattacharya T, Lapedes A, Polonis V, McCutchan F, Gilbert P, Self S, Korber B, Montefiori D, Mascola J. Tiered Categorization of a Diverse Panel of HIV-1 Env Pseudoviruses for Assessment of Neutralizing Antibodies. J Virol **2010**;84:1439-52.

58. Tomaras GD, Yates NL, Liu P, Qin L, Fouda GG, Chavez LL, Decamp AC, Parks RJ, Ashley VC, Lucas JT, Cohen M, Eron J, Hicks CB, Liao HX, Self SG, Landucci G, Forthal DN, Weinhold KJ, Keele BF, Hahn BH, Greenberg ML, Morris L, Karim SS, Blattner WA, Montefiori DC, Shaw GM, Perelson AS, Haynes BF. Initial B-cell responses to transmitted human immunodeficiency virus type 1: virion-binding immunoglobulin M (IgM) and IgG antibodies followed by plasma anti-gp41 antibodies with ineffective control of initial viremia. J Virol **2008**;82:12449-63.

59. Seaton K, Lan A, Goepfert P, Alam S, Parekh B, Robinson H, Tomaras GD. Increased Avidity Index to Immunodominant Epitope of HIV01 gp41 Env with MVA prime/boost vs. DNA prime MVA/boost in Human HIV-1 Vaccine Trial (HVTN 065). Keystone Symposium on HIV Vaccine, **2012**.

60. Simons JW, Jaffee EM, Weber CE, Levitsky HI, Nelson WG, Carducci MA, Lazenby AJ, Cohen LK, Finn CC, Clift SM, Hauda KM, Beck LA, Leiferman KM, Owens AH, Jr., Piantadosi S, Dranoff G, Mulligan RC,

Pardoll DM, Marshall FF. Bioactivity of autologous irradiated renal cell carcinoma vaccines generated by ex vivo granulocyte-macrophage colony-stimulating factor gene transfer. Cancer Res **1997**;57:1537-46.

61. Simons JW, Mikhak B, Chang JF, DeMarzo AM, Carducci MA, Lim M, Weber CE, Baccala AA, Goemann MA, Clift SM, Ando DG, Levitsky HI, Cohen LK, Sanda MG, Mulligan RC, Partin AW, Carter HB, Piantadosi S, Marshall FF, Nelson WG. Induction of immunity to prostate cancer antigens: results of a clinical trial of vaccination with irradiated autologous prostate tumor cells engineered to secrete granulocyte-macrophage colony-stimulating factor using ex vivo gene transfer. Cancer Res **1999**;59:5160-8.

62. Jaffee EM, Hruban RH, Biedrzycki B, Laheru D, Schepers K, Sauter PR, Goemann M, Coleman J, Grochow L, Donehower RC, Lillemoe KD, O'Reilly S, Abrams RA, Pardoll DM, Cameron JL, Yeo CJ. Novel allogeneic granulocyte-macrophage colony-stimulating factor-secreting tumor vaccine for pancreatic cancer: a phase I trial of safety and immune activation. J Clin Oncol **2001**;19:145-56.

63. Salgia R, Lynch T, Skarin A, Lucca J, Lynch C, Jung K, Hodi FS, Jaklitsch M, Mentzer S, Swanson S, Lukanich J, Bueno R, Wain J, Mathisen D, Wright C, Fidias P, Donahue D, Clift S, Hardy S, Neuberg D, Mulligan R, Webb I, Sugarbaker D, Mihm M, Dranoff G. Vaccination with irradiated autologous tumor cells engineered to secrete granulocyte-macrophage colony-stimulating factor augments antitumor immunity in some patients with metastatic non-small-cell lung carcinoma. J Clin Oncol **2003**;21:624-30.

64. Soiffer R, Hodi FS, Haluska F, Jung K, Gillessen S, Singer S, Tanabe K, Duda R, Mentzer S, Jaklitsch M, Bueno R, Clift S, Hardy S, Neuberg D, Mulligan R, Webb I, Mihm M, Dranoff G. Vaccination with irradiated, autologous melanoma cells engineered to secrete granulocyte-macrophage colony-stimulating factor by adenoviral-mediated gene transfer augments antitumor immunity in patients with metastatic melanoma. J Clin Oncol **2003**;21:3343-50.

65. Simons JW, Sacks N. Granulocyte-macrophage colony-stimulating factor-transduced allogeneic cancer cellular immunotherapy: the GVAX vaccine for prostate cancer. Urol Oncol **2006**;24:419-24.

66. Wang R, Richie TL, Baraceros MF, Rahardjo N, Gay T, Banania JG, Charoenvit Y, Epstein JE, Luke T, Freilich DA, Norman J, Hoffman SL. Boosting of DNA vaccine-elicited gamma interferon responses in humans by exposure to malaria parasites. Infect Immun **2005**;73:2863-72.

67. Parmiani G, Castelli C, Pilla L, Santinami M, Colombo MP, Rivoltini L. Opposite immune functions of GM-CSF administered as vaccine adjuvant in cancer patients. Ann Oncol **2007**;18:226-32.

68. Small EJ, Fong L. Developing immunotherapy as legitimate therapy for patients with prostate cancer. J Clin Oncol **2010**;28:1085-7.

69. Small EJ, Schellhammer PF, Higano CS, Redfern CH, Nemunaitis JJ, Valone FH, Verjee SS, Jones LA, Hershberg RM. Placebo-controlled phase III trial of immunologic therapy with sipuleucel-T (APC8015) in patients with metastatic, asymptomatic hormone refractory prostate cancer. J Clin Oncol **2006**;24:3089-94.

70. Uchida K, Nakata K, Suzuki T, Luisetti M, Watanabe M, Koch DE, Stevens CA, Beck DC, Denson LA, Carey BC, Keicho N, Krischer JP, Yamada Y, Trapnell BC. Granulocyte/macrophage-colony-stimulating factor autoantibodies and myeloid cell immune functions in healthy subjects. Blood **2009**;113:2547-56.

71. Agresti A, Coull BA. Approximate is better than "exact" for interval estimation of binomial proportions. Am Stat **1998**;52:119-26.

72. Huang Y, Gilbert P, Montefiori D, Self S. Simultaneous evaluation of the magnitude and breadth of a left- and right-censored multivariate response, with application to HIV vaccine development. Statistics in Biopharmaceutical Research **2009**;1:81-91.

73. Blanchard TJ, Alcami A, Andrea P, Smith GL. Modified vaccinia virus Ankara undergoes limited replication in human cells and lacks several immunomodulatory proteins: implications for use as a human vaccine. J Gen Virol **1998**;79:1159-67.

74. Sano J, Chaitman BR, Swindle J, Frey SE. Electrocardiography screening for cardiotoxicity after modified Vaccinia Ankara vaccination. Am J Med **2009**;122:79-84.

75. Update: cardiac-related events during the civilian smallpox vaccination program--United States, 2003. MMWR Morb Mortal Wkly Rep **2003**;52:492-6.

76. Li M, Gao F, Mascola JR, Stamatatos L, Polonis VR, Koutsoukos M, Voss G, Goepfert P, Gilbert P, Greene KM, Bilska M, Kothe DL, Salazar-Gonzalez JF, Wei X, Decker JM, Hahn BH, Montefiori DC. Human immunodeficiency virus type 1 env clones from acute and early subtype B infections for standardized assessments of vaccine-elicited neutralizing antibodies. J Virol **2005**;79:10108-25.

77. Gilbert P, Wang M, Wrin T, Petropoulos C, Gurwith M, Sinangil F, D'Souza P, Rodriguez-Chavez IR, DeCamp A, Giganti M, Berman PW, Self SG, Montefiori DC. Magnitude and breadth of a nonprotective neutralizing antibody response in an efficacy trial of a candidate HIV-1 gp120 vaccine. J Infect Dis **2010**;202:595-605.

Appendix A Sample informed consent form

Title: A phase 1 placebo controlled clinical trial to evaluate the safety and immunogenicity of a prime-boost vaccine regimen of GEO-D03 DNA and MVA/HIV62B vaccines in healthy, HIV-1-uninfected vaccinia naïve adult participants

Short title: HVTN 094

Site: [Insert site name]

Thank you for your interest in our research study. Please read this consent form or ask someone to read it to you. If you decide to join the study, we will ask you to sign this form. We will offer you a copy to keep. We will ask you questions to see if we have explained everything clearly. You can also ask us questions about the study. This will help you decide whether or not to join, or whether to continue in the study.

Research is not the same as treatment or medical care. The purpose of a research study is to answer scientific questions.

About the study

The HIV Vaccine Trials Network (HVTN) and GeoVax, Inc. are conducting a research study to test two HIV vaccines used in combination. HIV is the virus that causes AIDS.

About 48 people will take part in this study at multiple sites. The researcher in charge of this study at this clinic is [Insert name of site PI]. The US National Institutes of Health (NIH) is paying for the study.

1. **We are doing this study to answer several questions.**

- Are the study vaccines safe to give to people?
- Are people able to take the study vaccines without becoming too uncomfortable?
- How do people's immune systems respond to the study vaccines? (Your immune system protects you from disease.)
- Do different doses or schedules of the vaccines have different effects?

2. **The study vaccines cannot give you HIV.**

The study vaccines are not made from actual HIV. It is impossible for the study vaccines to give you HIV. Also, they cannot cause you to give HIV to someone else.

3. **We do not know if the study vaccines will decrease, increase, or not change your chance of becoming infected with HIV if you are exposed to the virus.**

Sites: Any change to the language in this section requires approval from HVTN Regulatory Affairs.

Several studies have tested whether HIV vaccines can reduce the risk of getting HIV from another person. In some studies, people who got the vaccine seemed to have the

same risk of getting HIV as people who did not get the vaccine. In one study, people who got the vaccine seemed to have a *lower* risk of getting HIV than people who did not get the vaccine. In another study, some men who got the vaccine had a *higher* risk of getting HIV than men who did not get the vaccine.

This study differs from the studies in which people who got the vaccine had a higher or lower risk of getting HIV. The study staff can tell you about the differences.

We do not know whether the vaccines in this study will affect your risk of getting HIV from another person. The risk could be higher, lower, or unchanged. It's very important to avoid exposure to HIV during and after the study. We will tell you how to avoid HIV.

4. These study vaccines are experimental.

The study vaccines are called GEO-D03 DNA and MVA/HIV62B. From here on, we will call them DNA and MVA or the "study vaccines." They are experimental HIV vaccines. That means we do not know whether the vaccines will be safe to use in people, or whether they will work to prevent HIV infection. These vaccines are used only in research studies.

The vaccines were developed by GeoVax, Inc.

DNA vaccine

DNA is a natural substance found in all living things, including people and viruses. DNA instructs cells to make proteins. In this study, the DNA vaccine will tell your body to make a small amount of some proteins that are found in HIV. Your body's immune system might recognize these proteins and prepare itself to fight HIV. This is called an immune response. The DNA vaccine is similar to natural DNA, but it was made in a laboratory. You cannot become infected with HIV or AIDS from the DNA vaccine or from these proteins.

This vaccine also contains DNA that will tell your body to make a protein called "granulocyte-macrophage colony-stimulating factor" (GM-CSF). GM-CSF is found naturally in your own body. It helps your bone marrow make the white blood cells in your body which fight infections. As part of this vaccine it is expected to alert your body's immune system against the HIV proteins. This may help the vaccine to work better. GM-CSF is approved by the FDA as a drug to help people who have had cancer treatments recover their white blood cells faster. In cancer treatment it is given in much larger amounts than will be given in this study. The DNA vaccine has not been given to people before. This vaccine is almost identical to a previous GeoVax DNA vaccine called pGA2/JS7 which has been used before in people. The difference between the previous DNA vaccine and the DNA vaccine in this study is the addition of the GM-CSF DNA described above. A similar version of GEO-D03 DNA that was made for monkey studies has been found to be safe in monkeys. Even if something looks like it is safe or works in animals, it may not be true for people.

MVA vaccine

The MVA vaccine was made from a virus called Modified Vaccinia Ankara virus, which was developed to protect against smallpox. The virus in the vaccine has been changed so that it will not spread in your body. Also, it cannot spread from you to other people. The MVA vaccine will instruct the body to make the same proteins as the DNA vaccine,

proteins that are found in HIV. These proteins may cause the body to have an immune response.

The MVA vaccine and the previous DNA vaccine have been given to people before in other HVTN studies. The first study of these vaccines in people involved 120 participants. In that study, the DNA and MVA vaccines did not cause health problems and were well tolerated. Another HVTN study is ongoing with the same vaccines as above. It will involve about 300 people.

Joining the study

5. It is completely up to you whether or not to join the study.

Take your time in deciding. If it helps, talk to people you trust, such as your doctor, friends or family. If you decide not to join this study, or if you leave it after you have joined, your other care at this clinic and the benefits or rights you would normally have will not be affected.

If you join this study, you may not be allowed to join other HIV vaccine or HIV prevention studies now or in the future. Also during the study, you should not donate blood or tissue.

If you choose not to join this study, you could join another study if one is available and you are eligible. If you are interested, we can tell you about other HIV vaccine and prevention studies that we know of.

Site: Remove item 6 if you use a separate screening consent that covers these procedures.

6. If you decide to join the study, we will screen you to see if you are eligible.

Screening involves a physical exam, HIV test and health history. A physical exam may include, but is not limited to:

- Checking your weight, temperature and blood pressure
- Looking in your mouth and throat
- Listening to your heart and lungs
- Feeling your abdomen (stomach and liver)

We will also do blood and urine tests. These tests tell us about some key aspects of your health, such as how healthy your kidneys, liver, and immune system are. We will also test you for these sexually transmitted diseases: syphilis, Hepatitis B, and Hepatitis C. We will ask you about medications you are taking. We will ask you about behaviors that might put you at risk for getting HIV. If you were born female, we will test you for pregnancy.

To see if you can join the study, we will do a heart test called an electrocardiogram (ECG, also known as EKG). For the ECG, we will place leads (suction cups, or stickers) on your chest, arms and legs and you will need to lie still for several seconds. This test will also let us see if there are any changes in your heart health later in the study.

We will review the screening results with you, and offer you counseling and referral if you need medical care. The screening results may show you are not eligible to join the study, even if you want to. You cannot be in another research study where you receive a study product and be enrolled in this study.

If you are allergic to eggs or egg products, you cannot be in this study.

If you ever received the smallpox vaccine, you cannot be in this study. The smallpox vaccine might interfere with the way your body responds to the MVA vaccine.

We will ask you about your history of drug use. Using some drugs may mean that you cannot join this study.

7. If you were born female and could become pregnant, you must agree to use birth control to join this study.

Site: List approved birth control methods here if you do not want to hand out the separate Approved Birth Control Methods sheet.

You must agree to use effective birth control from three weeks before your first injection until after your last clinic visit. We will talk to you about effective birth control methods. They are listed on a handout that we will give to you. *Site: Delete the preceding sentence if you list approved birth control methods in this consent form.* If you join the study, we will test you for pregnancy at some visits, including before each study injection.

Being in the study

If you are eligible to join the study after screening, and still want to participate, here is what will happen:

8. There are different groups in this study with different visit schedules. You will come to the clinic for about 18 – 21 scheduled visits over 14-16 months. This is explained further in Section 12.

Site: Edit number of visits and range of visit lengths if necessary. (There is site-specific variation in screening protocols and in the number of possible follow-up visits between protocol-mandated visits.)

Visits can last from [#] to [#] hours.

You may have to come for more visits if you have a laboratory or health issue.

We may contact you after the main study ends (eg, to tell you about the study results).

9. After you finish your clinic visits, we will contact you annually to ask about your health.

After the clinic visits are completed, we will contact you once each year to check on your health. These annual health contacts will continue until 3 years after you received your first study injection.

We will talk more about this part of the study in section 25 of this form.

10. We will give you [Site: Insert compensation] for each study visit you complete.

This amount is to cover the costs of [Site: Insert text]

Site: Insert any costs to participants (eg, birth control costs for female participants who could become pregnant).

Payments you receive for being in the study may be taxable. This happens if we pay you more than $600 between January 1 and December 31 of the same year. The clinic staff may need to ask you for your Social Security number for tax reasons.

11. We will give you either the study vaccines or a placebo.

Not everyone in this study will get the study vaccines. Some people will get a placebo, a substance that does not contain vaccine. We will compare the results from people who got the placebo with results from people who got the study vaccines. In this study, the placebo is sterile salt water.

You have a 5-in-6 chance of getting the study vaccines.

Site: Modify the randomization metaphor in the below paragraph as appropriate to your local culture.

Whether you get the study vaccines or the placebo is completely random, like rolling a dice.

The reason we are testing the study vaccines is because we do not know whether they work or are safe. That means we do not know whether it is better to get the vaccine or to get the placebo. In either case, you need to take steps to protect yourself from HIV infection.

The clinic staff have no say in whether you get the study vaccines or the placebo. They will not know which one you are getting, and neither will you. Only the pharmacist at your site will have this information while the study is going on, and he or she will keep it a secret.

You will have to wait until all participants complete their final study visits to find out whether you got the study vaccines or the placebo. This could be several years. But, if you have a serious medical problem and need to know what you got before the end of the study, we can tell you.

12. We will give you the study products on a schedule.

You will be in one of 3 groups. Which group you are in will depend on when you joined the study. We will tell you which group you are in. The groups test different doses and schedules of the study vaccines, as shown in the table below.

You will get 4 or 5 injections during the study by needle in the upper arm.

HVTN 094 Participant Injection Schedule

	First Injection Visit	2 Months later	4 Months later	6 Months later	8 Months later	10 Months later
Group 1	DNA vaccine (low dose) or Placebo	DNA vaccine (low dose) or Placebo	MVA vaccine or Placebo	MVA vaccine or Placebo	MVA vaccine or Placebo	--------
Group 2	DNA vaccine (full dose) or Placebo	DNA vaccine (full dose) or Placebo	MVA vaccine or Placebo	MVA vaccine or Placebo	--------	MVA vaccine or Placebo
Group 3	DNA vaccine (full dose) or Placebo	DNA vaccine (full dose) or Placebo	MVA vaccine or Placebo	--------	MVA vaccine or Placebo	--------

You will have to wait in the clinic for about a half hour after each injection to see if there are any problems. Then for that night and for three more days, you will need to write down your symptoms and tell us how you are feeling. Contact the clinic staff if you have any issues or concerns after receiving an injection. If you have a problem, we will continue to check on you until it goes away.

Your last visit is 6 months after your last injection. If you are in Group 2, this means your last visit will be 16 months after your first injection visit. If you are in Groups 1 or 3, your last visit will be 14 months after your first injection.

13. In addition to giving you the study products, we will perform these procedures:

- Regular HIV testing, as well as counseling on your results and on how to avoid getting HIV;
- Physical exams;
- Collection of blood and urine samples;
- Pregnancy tests if you were born female;
- Questions about your health, including medications you may be taking; and about any symptoms that could be early signs of heart problems;
- Personal questions about your HIV risk, including sexual behavior and drug use; and
- Questions about any personal problems or benefits you may have from participating in the study.

Site: Paste table of procedures in this section or distribute it as a separate sheet if it is helpful to your study participants.

We will review the results of these procedures and tests with you at your next visit, or sooner if necessary. We will also offer you counseling and referral for needed care.

14. We will test your samples for reactions to the study products.

In this study, we will need to take blood from you with a needle on several occasions. The amount will depend on the lab tests we need to do. It will be some amount between 8.5 mL and 200 mL (about 2 teaspoons to 1 cup). Your body will make new blood to replace the blood we take out.

Site: You may want to add a sentence to the end of the previous paragraph contextualizing the blood volumes described (eg, "To compare, people who donate blood in the US can give a total of about 500 mL in an 8-week period."). Modify the example for cultural relevance and alter blood volumes as necessary.

We will use some of your samples to see if you have side effects from the study products. We will share these results with you.

We also will send your samples (without your name) to a lab to see how your immune system responds to the study products. This may include genetic tests. These tests are for research purposes only. The lab will not give the results to you or this clinic, and the results will not become part of your study record.

After this testing, we will continue to store your samples in case we need to repeat any tests for this study.

15. When we take samples from you for this study, we take extra samples in case we have to repeat tests. If the samples are no longer needed for this study, the HVTN wants to keep them for use in other studies.

These other studies are likely to be about HIV and the immune system. However, they could also help researchers understand other diseases.

Below we will ask if you agree to donate your extra samples combined with limited information. It is your decision. What you decide will not affect your study participation or any care you receive here. If you do not agree, the HVTN will make sure that your samples are destroyed when they are no longer needed for this study. If you agree, there is no limit on how long your samples will be stored. You can change your mind at any time and your samples will be destroyed.

The HVTN will not sell your samples or information.

What information might be shared with the samples? <u>We will not share any information that would make it easy for anyone to identify you.</u> However, some information may be personal, such as your race, ethnicity, sex, and health, including HIV status. Other information may be what product you received and how your body responded to the product.

What type of studies might be done with the extra samples and information? We cannot guess exactly how your extra samples and information will be used. To use them, any researcher must have his or her institutional review board (IRB) or ethics committee (EC) review the use of the samples. The IRB/EC protects the rights and well-being of research participants.

The studies may include limited genetic testing. Your genes are passed to you from your birth parents. They affect how you look and how your body works. Limited genetic

testing involves only some of your genes, not all of your genes (your genome). The researchers will not look at all of your genes, only the genes related to the immune system and diseases.

If you agree, your extra samples and information could also be used for genome wide studies. In these studies, researchers will look at all of your genes (your genome). The researchers compare the genomes of many people, looking for common patterns of genes that could help them understand diseases.

The researchers may put the information from the genome wide studies into a protected database so that other researchers can access it. Usually, no one would be able to look at your genome and link it to you as a person. However, if another database exists that also has information on your genome and your name, someone might be able to compare the databases and identify you. The risk of this is very small.

Will I see the results of the studies? The researchers will not report their results to you, this clinic, or your doctor. The results will not be in your medical record. These other studies will not benefit you personally and they are not necessary for your medical care. Instead, the studies might help the public through new scientific discoveries.

Please write your initials or make your mark in the box next to the option you choose.

☐ I agree to donate my extra samples combined with limited information for other studies related to HIV, the immune system and other diseases. This may include limited genetic testing.

OR

☐ I agree to the option above and also to donate my extra samples combined with limited information for use in genome wide studies. I understand my genome and limited information may be put into a protected genome wide database.

OR

☐ I do not agree to donate my extra samples combined with limited information for use in other studies.

16. We will do our best to protect your private information.

Sites: Check HIPAA authorization for conflicts with this section.

Your study records and samples will be kept in a secure location. We will label all of your samples and most of your records with a code number, not your name or other personal information. However, it is possible to identify you, if necessary. We will not share your name with the lab that does the tests on your samples, or with anyone else who does not need to know your name.

Clinic staff will have access to your study records. Your records may also be reviewed by groups who watch over this study to see that we are protecting your rights, keeping you safe, and following the study plan. These groups include:

- The US National Institutes of Health and its study monitors,
- The US Food and Drug Administration,
- [Insert name of local IBC],
- [Insert name of local IRB/IEC],
- [Insert name of local and/or national regulatory authority as appropriate],
- GeoVax, Inc. and people who work for them,
- The HVTN and people who work for them,
- The HVTN Safety Monitoring Board or the NIAID Data and Safety Monitoring Board, and
- The US Office for Human Research Protections.

All reviewers will take steps to keep your records private.

We cannot guarantee absolute privacy. At this clinic, we have to report the following information:

Site: Include any public health or legal reporting requirements. Bulleted examples should include all appropriate cases (reportable communicable disease, risk of harm to self or others, etc.).

- [Item 1]
- [Item 2]
- [Item 3]

US sites: Include the following paragraph verbatim.

We have a Certificate of Confidentiality from the US government, to help protect your privacy. With the certificate, we do not have to release information about you to someone who is not connected to the study, such as the courts or police. Sometimes we can't use the certificate. Since the US government funds this research, we cannot withhold information from it. Also, you can still release information about yourself and your study participation to others.

Researchers who use your stored samples and limited information for other research will also do their best to protect your private information. The samples and limited information they receive will be labeled with a code number. They will not have your name or any personal information. Any reviewers of those studies will take steps to keep your records private.

The results of this study, and other studies that use the samples or information you agree to donate, may be published. No publication will use your name or identify you personally.

After the study is completed, information collected from the study may be made publically available to other researchers. If this is done, your name, personal information, and the code number used to identify you in our records will not be made available.

17. We may stop your injections or take you out of the study at any time.

This may happen if:

- you do not follow instructions,
- the researcher thinks that staying in the study might harm you,
- you get HIV,
- you enroll in a different research study where you receive another study product, or
- the study is stopped for any reason.

We may stop your injections or take you out of the study even if you want to continue and even if you were scheduled for additional injections. If we stop your injections, we may ask you to stay in the study to complete other study procedures.

18. If you become pregnant during the study, we will continue with some procedures but not injections.

We will do this for as long as it is safe for you and your developing baby.

If you leave the study while you are still pregnant, we will contact you after your due date to ask some questions about your pregnancy and delivery.

19. If you get infected with HIV during the study, we will help you get care and support.

You will not be able to stay in this study. We will counsel you about your HIV infection and about telling your partner(s). We will tell you where you can get support and medical care, and about other studies you may want to join. We will not provide or pay for any of your HIV care directly.

Risks

20. There are risks to being in this study.

This section describes the risks and restrictions we know about. There may also be unknown risks, even serious ones. We will tell you if we learn anything new that may affect your willingness to stay in the study.

Risks of routine medical procedures:

In this study, we will do some routine medical procedures. These are taking blood and giving injections. These procedures can cause bruising, pain, fainting, soreness, redness, swelling, itching, tingling, muscle damage, and (rarely) infection where the needle was inserted. Taking blood can cause a low blood cell count (anemia), making you feel tired.

General risks of vaccines:

Rarely, a vaccine can cause an allergic reaction, including a rash, hives, or difficulty breathing. **Allergic reactions can be life-threatening**. You should tell us if you have ever had a bad reaction to any injection or vaccine.

All vaccines can cause fever, chills, rash, aches and pains, nausea, headache, dizziness, and feeling tired. Most people can still do their planned activities after getting a vaccine. Rarely, people experience side effects that limit their normal activities or make them go to the doctor.

Very rarely, a vaccine causes an autoimmune disease in a person, or makes an autoimmune disease worse. An autoimmune disease happens when your immune system attacks your own body, instead of attacking an infection.

Risks of the DNA vaccine:

The DNA vaccine in this study has been tested in monkeys without causing injury. Even if something looks like it is safe in animals, the same may not be true for people. We do not know the risks of the study vaccine because it has not been given to people before. People in 2 other HVTN studies have been given the previous DNA vaccine and so far there have been no serious side effects. We do not expect these products to cause health problems.

Possible risks related to DNA vaccines include muscle damage and insertion of the vaccine DNA into the body's DNA, leading to cancer. We think the risk of these things happening is low. More than 1000 people have been given other DNA vaccines being tested against HIV and none of these things has happened so far.

Since 1995, thousands of people have received experimental DNA vaccines for diseases such as hepatitis, human papilloma virus (HPV, also known as genital warts), and HIV. In these people, the DNA vaccines also have not caused serious side effects.

We expect the risks of the DNA vaccine in this study to be similar to those of other DNA vaccines. However, there may be new side effects that we don't know about.

Risks of GM-CSF

The side effects experienced by people who got GM-CSF along with other vaccines were similar to the general risks of vaccines including mild or moderate arm pain / tenderness at the injection site, and symptom such as tiredness, fatigue/feeling unwell, muscle aches, headaches, joint pains, chills or nausea, in the first few days after an injection.

Some people can make antibodies against GM-CSF. Many healthy people have very low levels of these antibodies that their body has made against their own naturally occurring GM-CSF. Some people have a rare disease where they make very high levels of these antibodies and then develop problems with breathing and infections. The very low levels

of GM-CSF made by this vaccine are not expected to increase antibodies against GM-CSF. However, to learn more about this vaccine we will test for these antibodies.

Risks of the MVA vaccine:

The MVA vaccine is similar to the smallpox vaccine that has been used worldwide to protect against smallpox. The smallpox vaccine may cause certain heart problems in some people. The smallpox vaccine has caused swelling of the heart tissues in about 1 in every 175 people who received the vaccine. This result has not been seen in any trial using an MVA vaccine in healthy people. The MVA vaccine in this study is much weaker than smallpox vaccine, and is designed to avoid these problems. In other earlier studies, different experimental MVA vaccines against HIV have been given to over 300 healthy people and have not caused any heart problems or serious side effects. Even so, although it is very unlikely, it is possible that MVA vaccines, including this one, could be associated with heart problems. If you join the study, we will ask you about symptoms related to heart problems. Let us know right away if you are having any problems like extreme tiredness, chest pain, or difficulty breathing.

If we suspect a heart problem during the study, we may do additional testing to check your heart. We may refer you to a heart doctor for diagnosis and treatment. We will keep in close touch with you until the problem is over. We will ask you to sign a form to allow us to review your medical records for the heart problem.

Risks of DNA and MVA vaccines given together

The MVA vaccine used in this study has also been tested with a similar DNA vaccine to the one in this study in HVTN 065 and HVTN 205. No serious health problems have occurred. In earlier studies of the previous DNA vaccine and this MVA vaccine, most participants experienced only mild to moderate pain and/or tenderness at the injection site in the first few days after an injection. About half of all participants who got vaccine or placebo reported at least one symptom in the first few days after an injection, such as tiredness, fatigue/feeling unwell, muscle aches, headaches, joint pains, or chills, nausea or vomiting. Around 7% of participants—about 1 out of every 14 people—experienced lymph node (gland) swelling, pain or tenderness in the neck or armpit area closest to the injection site. These symptoms were mild or moderate and usually improved or went away within a few days. About 1% of people in HVTN 205 felt a severe reaction of pain or tenderness, fever, or ill feeling from an injection of vaccine or placebo. These symptoms went away completely. Two people had severe temporary lab changes (low white blood cell count and low blood sugar) that were probably not related to the injections. The low white blood cell count resolved within 7 days and the low blood sugar resolved within 10 days. No serious illnesses related to the injections have been reported. This study is still ongoing and if we get new information that is important for you to know, we will tell you.

This study will include an extra MVA injection that was not given in the previous studies. It is expected to improve the immune response. The risks of this extra injection are unknown.

Other changes in blood tests such as a lower white blood cell count or hemoglobin level or a higher muscle enzyme (called CPK) may possibly occur from a study vaccine.

Personal problems/discrimination/testing HIV antibody positive:

About 10 to 20% of HVTN participants report personal problems or discrimination because of joining an HIV vaccine study. Family or friends may worry, get upset or angry, or assume that you are infected with HIV or at high risk and treat you unfairly as a result. Rarely, a participant has lost a job because the study took too much time away from work, or because their employer thought they had HIV.

The study vaccines may cause you to test positive on some types of HIV tests. This means that after you get the study vaccines, a routine HIV test done outside this clinic may say you have HIV, even if you don't. For this reason, you should plan to get HIV tests only at this clinic during the study. Our tests can tell the difference between true HIV infection and a positive result that is caused by the study vaccines.

If you receive a positive test result caused by the study vaccines at any time, we can provide you with free HIV testing for as long as you need it. If this happens, we do not know how long you will test positive due to the study vaccines. If you receive a positive HIV test result and we determine it is because you have HIV, we will refer you for follow-up care.

It is unlikely, but you could test negative at the end of the study and positive some time later, even though you don't have HIV. This could happen if different HIV tests come into use. We will give you a phone number to call for more information.

If someone believes you are infected with HIV even if you are not, you could face discrimination and other problems. For example, you could be denied medical or dental care, employment, insurance, a visa, or entry into the military. If you do have a positive HIV antibody test caused by the study vaccines, you will not be able to donate blood or organs. Your family and friends may treat you differently. We will give you a brochure that tells you more about testing HIV positive because of an HIV vaccine, and how you can avoid some of these problems.

Site: Modify the preceding paragraph if applicable.

Embarrassment/anxiety:

You may feel embarrassed when we ask about your HIV risks, such as having sex and using drugs. Also, waiting for your HIV test results or other health test results could make you feel anxious. You could feel worried if your test results show that you are infected with HIV. If you feel embarrassed or anxious, please tell us and we will try to help you.

Risks of disclosure of your personal information:

We will take several steps to protect your personal information. Although the risk is very low, it is possible that your personal information could be given to someone who should not have it. If that happened, you could face discrimination, stress, and embarrassment. We can tell you more about how we will protect your personal information if you would like it.

Unknown risks:

We do not know if the study vaccines will increase, decrease, or not change your risk of becoming infected with HIV if exposed. If you get infected with HIV, we do not know

how the study vaccines might affect your HIV infection or how long it takes to develop AIDS.

We do not know if getting these study vaccines will affect how you respond to any future approved HIV vaccine. It could be that a future HIV vaccine may not work as well for you because you got the study vaccines. Currently, no HIV vaccine has been approved for use.

We do not know how the study vaccines will affect a pregnant participant or a developing baby.

Benefits

21. The study may not benefit you.

We do not know whether getting the study vaccines might benefit you in any way. However, being in the study might still help you in some ways. The counseling that you get as part of the study may help you avoid getting HIV. The lab tests and physical exams that you get while in this study might detect health problems you don't yet know about.

This study may help in the search for a vaccine to prevent HIV. However, if the study vaccines later become approved and sold, there are no plans to share any money with you. You will also not receive any money if you decide to donate your extra samples and limited information for other research, even if this research leads to a new product or discovery.

Your rights and responsibilities

22. If you join the study, you have rights and responsibilities.

As a participant, you have many rights that we respect. You also have responsibilities. We will give you the Participant's Bill of Rights and Responsibilities (PBORR). It describes your rights and responsibilities as a study participant.

Leaving the study

23. Tell us if you decide to leave the study.

You are free to leave the study at any time and for any reason. Your care at this clinic and your legal rights will not be affected, but it is important for you to let us know.

We will ask you to come back to the clinic one last time for a physical exam, and we may ask to take some blood and urine samples. We will also ask about any personal problems or benefits you have experienced from being in the study. We believe these steps are important to protecting your health, but it is up to you whether to complete them.

Injuries

24. If you get sick or injured during the study, contact us immediately.

Your health is important to us. We will help you get the medical care you need.

When someone gets sick or injured in an HVTN study, the HVTN decides whether the injury is probably related to the study products/procedures. If the HVTN decides it was more likely due to the study products or procedures than any other cause, then the HVTN and/or the vaccine developer will use their funds to pay for treatment. If the HVTN decides otherwise, then you and your health insurance *(Sites: insert locale- appropriate medical insurance language in the preceding paragraph)* would be responsible for treatment costs. You may disagree with the decision the HVTN makes about your injuries. At your request the HVTN will ask experts who are not connected with the HVTN to review its decision.

In this study, the vaccine developer will pay the cost of medical expenses that arise from injuries caused by the study products.

For injuries caused by study procedures, the HVTN has limited funds to cover the cost of medical treatment.

No matter what, you still have the right to use the court system to address payment for your injuries if you are not satisfied.

Some injuries are not physical. For example, someone might be harmed psychologically or emotionally by being in an HIV vaccine study. Or they might lose wages from injuries because they could not go to work. No funds have been set aside to pay for nonphysical injuries, even if they are related to participation in the study.

Annual health contacts

25. After your clinic visits end, we will contact you once a year until 3 years after your first injection.

We will contact you by phone or email *[Site: Modify mode of contact as appropriate]* once a year to ask questions about your health. If you prefer to answer these questions in person, an appointment with the study clinic can be arranged.

If we have any concerns about your health, we may need to have additional contact with you. You are also welcome to contact us at any time if you have concerns about your health related to your study participation.

If we ask you to come to the clinic, we will give you [Site: Insert compensation amount] for each visit. This amount is to cover the costs of [Site: Insert text].

If someone outside this study clinic told you that you are infected with HIV, we will ask you to come back to the clinic for another HIV test. We will draw about 15 mL (1 tablespoon) of blood. We may ask you to come back more than once for this testing.

Because we will want to contact you once a year, please tell us if your contact information changes, if you are moving away, or if you do not want us to contact you anymore.

You can tell us at any time that you don’t want any more annual health contacts. If you do so, you will not lose any benefits or rights you would normally have.

All other information that is discussed earlier in this consent also applies to the annual health contacts.

Questions

26. If you have questions or problems at any time during your participation in this study, use the following important contacts.

If you have questions about this study, contact
[name and telephone number of the investigator or other study staff].

If you have any symptoms that you think may be related to this study, contact
[name and telephone number of the investigator or other study staff].

If you have questions about your rights as a research participant, or problems or concerns about how you are being treated in this study, contact
[name/title/phone of person on IRB or other appropriate organization].

If you want to leave this study, contact
[name and telephone number of the investigator or other study staff].

Your signature

27. Before you sign this consent form, make sure of the following:

- You have read this consent form, or someone has read it to you.
- You feel that you understand what the study is about and what will happen to you if you join. You understand what the possible risks and benefits are.
- You have had your questions answered and know that you can ask more.
- You agree to join this study.

You will not be giving up any of your rights by signing this consent form.

Participant's name (print)	Participant's signature or mark	Date	Time
Clinic staff conducting consent discussion (print)	Clinic staff signature	Date	Time

For participants who are unable to read or write, a witness should complete the signature block below:

______________________	______________________	__________	__________
Witness's name (print)	Witness's signature	Date	Time

*Witness is impartial and was present for the consent process.

Appendix B Approved birth control methods (for sample informed consent form)

You should not become pregnant during the study because we do not know how the study vaccines could affect the developing baby.

If you were born female and are sexually active in a way that could lead you to get pregnant, you must agree to use effective birth control, starting at least 3 weeks before you get your first injection of study vaccines or placebo and continuing until after your last clinic visit as specified in the informed consent.

Effective birth control means using any of the following methods every time you have sex:

- Birth control drugs that prevent pregnancy—given by pills, shots, patches, vaginal rings, or inserts under the skin;
- Male or female condoms, with or without a cream or gel that kills sperm;
- Diaphragm or cervical cap with a cream or gel that kills sperm;
- Intrauterine device (IUD); or
- Any other contraceptive method approved by the researchers.

You do not have to use birth control if:

- You are only having sex with a partner or partners who have had a vasectomy. (We will ask you some questions to confirm that the vasectomy was successful.);
- You have reached menopause, with no menstrual periods for one year;
- You have had a hysterectomy (your uterus removed);
- You have had your ovaries removed;
- You have a tubal ligation (your "tubes tied") or confirmed successful placement of a product that blocks the fallopian tubes;
- You are having sex only with a female partner or partners;
- You only have oral sex; or,
- You are sexually abstinent (no sex at all).

Remember: If you are having sex, you need to use male or female condoms to protect yourself from HIV infection.

Appendix C HVTN VISP registry consent

The HIV Vaccine Trials Network (HVTN) would like your permission to enter your name and link it to information about you in a computer registry (the “VISP registry”). By having your name and vaccine study information in the VISP registry, trained HVTN staff can quickly help you if you have problems with VISP.

About VISP

The body makes antibodies to prevent infection. Most vaccines cause the body to make antibodies as a way of preventing infection. Your body may make antibodies to HIV because you received an HIV vaccine. Some HIV test results could come back positive even if you are not infected with HIV. This is called a VISP (vaccine-induced seropositive) test result. We do not know who will have VISP test results or how long these test results may last.

People with VISP test results need specific HIV tests. These tests can tell whether a test result is VISP or a real HIV infection. These people may need help explaining their VISP situation if someone outside the study wants to test them for HIV. VISP test results may cause problems in several areas like insurance, job applications, the military, prison, visa applications, emigration/immigration, and blood and tissue donation.

We are asking you for your permission to enter your name in the registry now in case you have VISP test results later. The registry will not be used for any other purpose.

What are the benefits of the registry?

Your study site will help you with problems related to VISP test results. If you are unable to go to your original study site, an HVTN counselor will help you with these problems. The HVTN counselor will need to verify your study participation and if you received an HIV vaccine. The registry gives the HVTN counselor quick access to this information.

If you choose not to have your name entered in the registry, HVTN counselors still will do their best to help you. However, it will take longer to get that information. If your study site is no longer doing HIV vaccine studies, your records may be stored securely off site. It is possible your records may not be found.

What information does the registry contain and how is it protected?

The registry contains the following information:

- Your participant ID (the code used for you instead of your name at your study site)
- The study network and study you were in
- The site where you began the study
- The date you began the study

- Your date of birth or age
- If you received an HIV vaccine that may cause you to test VISP

We are asking for your permission to enter your name into the registry and link it to the information above.

The registry will NOT contain:

- Your HIV test results
- Your phone number or any other way to contact you

Any other personal information that you discuss with the HVTN counselor will be kept separate from the registry. We will keep your name in the registry until you tell us you want it removed.

All people who work with your registry information sign agreements to keep the information confidential.

The registry is a secured computer database. It can only be accessed with a password.

What are the risks?

The only risk to having your name entered and linked to the other pieces of information in the registry is that someone who is not authorized might see your information. The risk of this happening is low because of the security protections in place. However, we cannot guarantee this will never happen.

What if I have more questions about the registry?

Please talk to your study site or call the phone number they provided if you have any questions about the registry now or in the future.

If I agree now, can I change my mind later?

Yes. You can contact your study site or call the number you have been given anytime to tell us that you would like your name to be deleted from the registry. We will send you confirmation that we deleted it. Your decision will not affect your participation in the main HIV vaccine study.

By signing this form, you do not give up any legal rights.

Please write your initials or make your mark in the box next to the option you choose.

☐ I **AGREE** to allow my name to be entered and linked to the information in the HVTN VISP registry.

☐ I **DO NOT AGREE** to allow my name to be entered and linked to the information in the HVTN VISP registry.

Please sign or make your mark below.

Participant's name (print)	Participant's signature or mark	Date	Time
Study staff conducting consent discussion (print)	Study staff signature	Date	Time

For participants who are unable to read or write, a witness should complete the signature block below:

Witness's name (print)[#]	Witness's signature	Date	Time

Witness is impartial and was present for the consent process.

Appendix D Table of procedures for Group 1 (for sample informed consent form)

	Visits																
	Screening	**1st injection** (Month 0)	3 days after 1st injection	7 days after 1st injection	2 weeks after 1st injection	**2nd injection** (Month 2)	3 days after 2nd injection	1 week after 2nd injection	2 weeks after 2nd injection	**3rd injection** (Month 4)	2 weeks after 3rd injection	**4th injection** (Month 6)	2 weeks after 4th injection	**5th injection** (Month 8)	2 weeks after 5th injection	3 months after 5th injection	6 months after 5th injection
**Procedure**	Visit 1	Visit 2	Visit 4	Visit 5	Visit 6	Visit 7	Visit 8	Visit 9	Visit 10	Visit 11	Visit 15	Visit 16	Visit 17	Visit 18	Visit 19	Visit 22	Visit 24
Injection		√				√				√		√		√			
Medical history	√																
Complete physical	√																√
Brief physical		√		√	√	√		√	√	√	√	√	√	√	√	√	
ECG+	√																
Urine sample	√				√										√		
Blood drawn	√	√	√	√	√	√	√	√	√		√		√		√	√	√
Pregnancy test (participants born female)	√	√				√				√		√		√		√	
HIV testing / counseling	√	√				√		√			√	√			√	√	√
Interview / questionnaire	√	√	√	√	√	√	√	√	√	√	√	√	√	√	√	√	√
Risk reduction counseling	√	√		√	√	√		√	√	√	√	√	√	√	√	√	√

+ECG may be repeated at later visits in some cases

***Note*: Visits 3, 12, 13, 14, 20, 21, 23, and 25 not applicable to Group 1 and are not shown on this table.**

Not shown in this table is a time after all study participants have completed their last scheduled visit when you can find out what products you received

Appendix E Table of procedures for Group 2 (for sample informed consent form)

	Visits																				
	Screening	**1st injection** (Month 0)	1 day after 1st injection	3 days after 1st injection	7 days after 1st injection	2 weeks after 1st injection	**2nd injection** (Month 2)	3 days after 2nd injection	1 week after 2nd injection	2 weeks after 2nd injection	**3rd injection** (Month 4)	1 day after 3rd injection	3 days after 3rd injection	1 week after 3rd injection	2 weeks after 3rd injection	**4th injection** (Month 6)	2 weeks after 4th injection	**5th injection** (Month 10)	2 weeks after 5th injection	3 months after 5th injection	6 months after 5th injection
**Procedure**	Visit 1	Visit 2	Visit 3	Visit 4	Visit 5	Visit 6	Visit 7	Visit 8	Visit 9	Visit 10	Visit 11	Visit 12	Visit 13	Visit 14	Visit 15	Visit 16	Visit 17	Visit 20	Visit 21	Visit 23	Visit 25
Injection		√					√				√					√		√			
Medical history	√																				
Complete physical	√																				√
Brief physical		√			√	√	√		√	√	√			√	√	√	√	√	√	√	
ECG[+]	√																				
Urine sample	√					√													√		
Blood drawn	√	√	√	√	√	√	√	√	√	√	√	√	√	√	√		√		√	√	√
Pregnancy test (participants born female)	√	√					√				√					√		√		√	
HIV testing / counseling	√	√					√		√						√	√	√		√	√	√
Interview / questionnaire	√	√	√	√	√	√	√	√	√	√	√	√	√	√	√	√	√	√	√	√	√
Risk reduction counseling	√	√			√	√	√		√	√	√			√	√	√	√	√	√	√	√

Grayed out columns = visits not required for designated CRS

[+]ECG may be repeated at later visits in some cases

***Note*: Visits 18, 19, 22, and 24 not applicable to Group 2 and are not shown on this table.**

Not shown in this table is a time after all study participants have completed their last scheduled visit when you can find out what products you received

Appendix F Table of procedures for Group 3 (for sample informed consent form)

	Visits																		
	Screening	**1st injection** (Month 0)	1 day after 1st injection	3 days after 1st injection	7 days after 1st injection	2 weeks after 1st injection	**2nd injection** (Month 2)	3 days after 2nd injection	1 week after 2nd injection	2 weeks after 2nd injection	**3rd injection** (Month 4)	1 day after 3rd injection	3 days after 3rd injection	1 week after 3rd injection	2 weeks after 3rd injection	**4th injection** (Month 8)	2 weeks after 4th injection	3 months after 4th injection	6 months after 4th injection
**Procedure**	Visit 1	Visit 2	Visit 3	Visit 4	Visit 5	Visit 6	Visit 7	Visit 8	Visit 9	Visit 10	Visit 11	Visit 12	Visit 13	Visit 14	Visit 15	Visit 18	Visit 19	Visit 22	Visit 24
Injection		√					√				√					√		√	
Medical history	√																		
Complete physical	√																		√
Brief physical		√			√	√	√		√	√	√			√	√	√	√	√	
ECG+	√																		
Urine sample	√					√											√		
Blood drawn	√	√	√	√	√	√	√	√	√	√	√	√	√	√	√		√	√	√
Pregnancy test (participants born female)	√	√					√				√					√		√	
HIV testing / counseling	√	√					√		√						√	√	√	√	√
Interview / questionnaire	√	√	√	√	√	√	√	√	√	√	√	√	√	√	√	√	√	√	√
Risk reduction counseling	√	√			√	√	√		√	√	√			√	√	√	√	√	√

Grayed out columns = visits not required for designated CRS
+ECG may be repeated at later visits in some cases

***Note*: Visits 16, 17, 20, 21, 23, and 25 not applicable to Group 3 and are not shown on this table**

Not shown in this table is a time after all study participants have completed their last scheduled visit when you can find out what products you received

Appendix G Laboratory procedures for Group 1

					Tube volume (mL)																									
				Visit:	1	2	3	4	5	6	7	8	9	10	11	12	13	14	15	16	17	18	19	20	21	22	23	24	25	
				Day:	Screening	D0	D1	D3	D7	D14	D56	D59	D63	D70	D112	D113	D115	D119	D126	D168	D182	D224	D238	D303	D317	D334	D394	D425	D485	
				Month:	visit[4]	M0			M0.25	M0.5	M2		M2.25	M2.5	M4			M4.25	M4.5	M6	M6.5	M8	M8.5	M10	M10.5	M11	M13	M14	M16	
						VAC1					VAC2				VAC3					VAC4		VAC5								
**Description**	**Ship to[1,2]**	**Assay location[2]**	**Tube[5]**	**Tube size (vol capacity)[5]**		DNA					DNA				MVA					MVA		MVA								**Total**
BLOOD COLLECTION																														
Screening or diagnostic assays																														
Screening HIV test	Local lab	Local lab	SST/EDTA	5mL	5	—	—	—	—	—	—	—	—	—	—	—	—	—	—	—	—	—	—	—	—	—	—	—	—	**5**
HBsAg/anti-HCV/Syphilis	Local lab	Local lab	SST	8.5mL	8.5	—	—	—	—	—	—	—	—	—	—	—	—	—	—	—	—	—	—	—	—	—	—	—	—	**8.5**
HIV in-study diagnostic test[10]	UW-VSL	UW-VSL	EDTA	10mL	—	—	—	—	—	—	10	—	—	—	—	—	—	—	10	—	—	—	10	—	—	10	—	20	—	**60**
Safety labs																														
CBC/ Diff/ platelets	Local lab	Local lab	EDTA	5mL	5	5	—	5	5	5	5	5	5	5	—	—	—	—	5	—	5	—	5	—	—	5	—	—	—	**65**
Chemistry Panel[6]	Local lab	Local lab	SST	5mL	5	—	—	—	—	5	—	—	—	5	—	—	—	—	5	—	5	—	5	—	—	5	—	—	—	**35**
Cardiac Troponin[3]	Local lab	Local lab	Hep	5mL	—	—	—	—	—	—	—	—	—	—	—	—	—	—	—	—	—	—	—	—	—	—	—	—	—	**0**
GM-CSF Assays																														
GM-CSF levels	CSR	CCHRF	SST	3.5mL	—	w	—	3.5	3.5	—	3.5	3.5	3.5	—	—	—	—	—	—	—	—	—	—	—	—	—	—	—	—	**17.5**
Anti-GM-CSF binding Ab assay	CSR	CCHRF	SST	3.5mL	—	w	—	—	—	3.5	—	—	—	w	—	—	—	—	w	—	—	—	—	—	—	—	—	—	—	**3.5**
Immunogenicity assays[7]																														
HLA typing[8]	CSR	FHCRC	ACD	8.5mL	—	17	—	—	—	—	—	—	—	—	—	—	—	—	—	—	—	—	—	—	—	—	—	—	—	**17**
Humoral Assays																														
HIV binding Ab assay	CSR	Duke	EDTA	10mL	—	***10***	—	—	—	—	—	—	—	10	—	—	—	—	10	—	***10***	—	***10***	—	—	—	—	10	—	**60**
HIV neut Ab assay	CSR	Duke	SST	8.5mL	—	8.5	—	—	—	—	—	—	—	8.5	—	—	—	—	8.5	—	***8.5***	—	***8.5***	—	—	—	—	8.5	—	**51**
HIV Ab avidity assay	CSR	Duke	SST	—	—	v	—	—	—	—	—	—	—	—	—	—	—	—	—	—	***v***	—	***v***	—	—	v	—	v	—	**0**
Cellular Assays																														
HIV-specific ICS	CSR	FHCRC	ACD	8.5mL	—	34	—	—	—	—	—	—	—	34	—	—	—	—	34	—	***34***	—	***34***	—	—	—	—	34	—	**204**
Cytokine multiplex bead array	CSR	FHCRC	ACD	8.5mL	—	—	—	—	—	—	—	—	—	—	—	—	—	—	—	—	***8.5***	—	***8.5***	—	—	—	—	—	—	**17**
Storage																														
Serum storage (incl ADCC)	CSR	—	SST	5mL or 8.5mL	—	17	—	—	—	—	—	—	—	17	—	—	—	—	13.5	—	17	—	17	—	—	17	—	17	—	**115.5**
Plasma storage	CSR	—	EDTA	10mL	—	20	—	—	—	—	—	—	—	10	—	—	—	—	10	—	20	—	20	—	—	—	—	10	—	**90**
Plasma storage	CSR	—	ACD	8.5mL	—	z	—	—	—	—	—	—	—	z	—	—	—	—	z	—	z	—	z	—	—	—	—	z	—	**0**
PBMC storage	CSR	—	ACD	8.5mL	—	68	—	—	—	—	—	—	—	51	—	—	—	—	76.5	—	59.5	—	59.5	—	—	—	—	51	—	**365.5**
**Maximum Total**					**23.5**	**179.5**	**0**	**8.5**	**8.5**	**13.5**	**18.5**	**8.5**	**8.5**	**140.5**	**0**	**0**	**0**	**0**	**172.5**	**0**	**167.5**	**0**	**177.5**	**0**	**0**	**37**	**0**	**150.5**	**0**	**1114.5**
**Maximum 56-Day total**					**23.5**	**203**	**203**	**211.5**	**220**	**234**	**228.5**	**57.5**	**57.5**	**189.5**	**176**	**140.5**	**0**	**0**	**313**	**172.5**	**340**	**167.5**	**345**	**0**	**0**	**37**	**0**	**150.5**	**0**	
URINE COLLECTION																														
Urinalysis	Local lab	Local lab			X	—	—	—	—	X	—	—	—	—	—	—	—	—	—	—	—	—	X	—	X	—	—	—	—	
Pregnancy test[9]	Local lab	Local lab			X	X	—	—	—	—	X	—	—	—	X	—	—	—	—	X	—	X	—	X	—	X	X	—	—	

Grayed out columns = visits not applicable to Group 1.
v = 1mL of serum will be taken out of serum storage to perform HIV Ab avidity assays; no separate blood draw is needed
w = 0.5mL of serum will be taken out of storage to perform GM-CSF assays; no separate blood draw is needed
z = up to 5mL of plasma will be harvested during ACD blood PBMC processing; no separate blood draw is needed

[1] CSR = central specimen repository

[2] HVTN Laboratory Program includes laboratories at UW-VSL, Duke, and FHCRC = University of Washington Virology Specialty Laboratory (Seattle, Washington, USA); Duke = Duke University Medical Center (Durham, North Carolina, USA); FHCRC = Fred Hutchinson Cancer Research Center (Seattle, Washington, USA).

Non-HVTN laboratories: CCHRF. CCHRF=Cincinnati Children's Hospital Research Foundation (Cincinnati, OH, USA)

[3] Cardiac troponin to be drawn if clinically indicated.

[4] Screening may occur over the course of several contacts/visits up to and including day 0 prior to vaccination.

[5] Local labs may assign appropriate alternative tube types for locally performed tests.

[6] Chemistry panels are defined in section 9.2 (pre-enrollment) and section 9.4 (postenrollment).

[7] Based on the number of responders observed at the primary immunogenicity timepoints (visits 2 (only for binding Ab assay), 17, and 19), lab assays may be performed on all participants for humoral and cellular responses at other timepoints

[8] Genotyping may be performed on enrolled participants using cryopreserved PBMC collected at baseline, initially in participants who demonstrate vaccine-induced T-cell responses at post-vaccination timepoints.

[9] Pregnancy tests may be performed on blood specimens.

[10] At an early termination visit for a withdrawn or terminated participant (see section 9.11), blood should be drawn for HIV diagnostic testing, as shown for visit 24 above.

Appendix H Laboratory procedures for Group 2

Description	Ship to[1,2]	Assay location[2]	Tube[5]	Tube size (vol capacity)[5]	Tube volume (mL)																									Total
				Visit:	1	2	3	4	5	6	7	8	9	10	11	12	13	14	15	16	17	18	19	20	21	22	23	24	25	
				Day:	Screening	D0	D1	D3	D7	D14	D56	D59	D63	D70	D112	D113	D115	D119	D126	D168	D182	D224	D238	D303	D317	D334	D394	D425	D485	
				Month:	visit[4]	M0			M0.25	M0.5	M2		M2.25	M2.5	M4			M4.25	M4.5	M6	M6.5	M8	M8.5	M10	M10.5	M11	M13	M14	M16	
						VAC1					VAC2				VAC3					VAC4				VAC5						
						DNA					DNA				MVA					MVA				MVA						
BLOOD COLLECTION																														
Screening or diagnostic assays																														
Screening HIV test	Local lab	Local lab	SST/EDTA	5mL	5	—	—	—	—	—	—	—	—	—	—	—	—	—	—	—	—	—	—	—	—	—	—	—	—	**5**
HBsAg/anti-HCV/Syphilis	Local lab	Local lab	SST	8.5mL	8.5	—	—	—	—	—	—	—	—	—	—	—	—	—	—	—	—	—	—	—	—	—	—	—	—	**8.5**
HIV in-study diagnostic test[10]	UW-VSL	UW-VSL	EDTA	10mL	—	—	—	—	—	—	10	—	—	—	—	—	—	—	10	—	10	—	—	—	10	—	10	—	20	**70**
Safety labs																														
CBC/ Diff/ platelets	Local lab	Local lab	EDTA	5mL	5	5	5	5	5	5	5	5	5	5	5	5	5	5	5	—	5	—	—	—	5	—	5	—	—	**90**
Chemistry Panel[6]	Local lab	Local lab	SST	5mL	5	—	—	—	—	5	—	—	—	5	—	—	—	—	5	—	5	—	—	—	5	—	5	—	—	**35**
Cardiac Troponin[3]	Local lab	Local lab	Hep	5mL	—	—	—	—	—	—	—	—	—	—	—	—	—	—	—	—	—	—	—	—	—	—	—	—	—	**0**
GM-CSF Assays																														
GM-CSF levels	CSR	CCHRF	SST	3.5mL	—	w	—	3.5	3.5	—	3.5	3.5	3.5	—	—	—	—	—	—	—	—	—	—	—	—	—	—	—	—	**17.5**
Anti-GM-CSF binding Ab assay	CSR	CCHRF	SST	3.5mL	—	w	—	—	—	3.5	—	—	—	w	—	—	—	—	w	—	—	—	—	—	—	—	—	—	—	**3.5**
Immunogenicity assays[7]																														
HLA typing[8]	CSR	FHCRC	ACD	8.5mL	—	17	—	—	—	—	—	—	—	—	—	—	—	—	—	—	—	—	—	—	—	—	—	—	—	**17**
Humoral Assays																														
HIV binding Ab assay	CSR	Duke	EDTA	10mL	—	***10***	—	—	—	—	—	—	—	10	—	—	—	—	10	—	***10***	—	—	—	***10***	—	—	—	10	**60**
HIV neut Ab assay	CSR	Duke	SST	8.5mL	—	8.5	—	—	—	—	—	—	—	8.5	—	—	—	—	8.5	—	***8.5***	—	—	—	***8.5***	—	—	—	8.5	**51**
HIV Ab avidity assay	CSR	Duke	SST	—	—	v	—	—	—	—	—	—	—	—	—	—	—	—	—	—	***v***	—	—	—	***v***	—	v	—	v	**0**
Cellular Assays																														
HIV-specific ICS	CSR	FHCRC	ACD	8.5mL	—	34	—	—	—	—	—	—	—	34	—	—	—	—	34	—	***34***	—	—	—	***34***	—	—	—	34	**204**
Cytokine multiplex bead array	CSR	FHCRC	ACD	8.5mL	—	—	—	—	—	—	—	—	—	—	—	—	—	—	—	—	***8.5***	—	—	—	***8.5***	—	—	—	—	**17**
Innate Immunity Assays[11]																														
RNA gene expression	CSR	FHCRC	ACD	8.5mL	—	8.5	8.5	8.5	—	8.5	8.5	—	—	8.5	8.5	8.5	8.5	8.5	8.5	—	—	—	—	—	—	—	—	—	—	**93.5**
Trucount	FHCRC	FHCRC	ACD	—	—	y	y	y	—	y	y	—	—	y	y	y	y	y	y	—	—	—	—	—	—	—	—	—	—	**0**
Serum cytokines	CSR	FHCRC	SST	3.5 or 5mL	—	5	3.5	3.5	—	3.5	3.5	—	—	3.5	3.5	3.5	3.5	3.5	3.5	—	—	—	—	—	—	—	—	—	—	**40**
Storage																														
Serum storage (incl ADCC)	CSR	—	SST	5mL or 8.5mL	—	17	5	—	—	—	5	5	5	17	5	5	5	5	8.5	—	17	—	—	—	17	—	17	—	17	**150.5**
Plasma storage	CSR	—	EDTA	10mL	—	20	—	—	—	—	—	—	—	10	—	—	—	—	10	—	20	—	—	—	20	—	—	—	10	**90**
Plasma storage	CSR	—	ACD	—	—	z	z	z	—	z	z	z	z	z	z	z	z	z	z	—	z	—	—	—	z	—	—	—	z	**0**
PBMC storage	CSR	—	ACD	8.5mL	—	68	25.5	25.5	—	25.5	25.5	17	17	51	17	17	17	17	68	—	59.5	—	—	—	59.5	—	—	—	51	**561**
**Maximum Total**					**23.5**	**193**	**47.5**	**46**	**8.5**	**51**	**61**	**30.5**	**30.5**	**152.5**	**39**	**39**	**39**	**39**	**171**	**0**	**177.5**	**0**	**0**	**0**	**177.5**	**0**	**37**	**0**	**150.5**	**1513.5**
**Maximum 56-Day total**					**23.5**	**216.5**	**264**	**310**	**318.5**	**369.5**	**407**	**197**	**181.5**	**325.5**	**313.5**	**230.5**	**117**	**117**	**479.5**	**327**	**348.5**	**177.5**	**177.5**	**0**	**177.5**	**0**	**37**	**0**	**150.5**	
URINE COLLECTION																														
Urinalysis	Local lab	Local lab			X	—	—	—	—	X	—	—	—	—	—	—	—	—	—	—	—	—	X	—	X	—	—	—	—	
Pregnancy test[9]	Local lab	Local lab			X	X	—	—	—	—	X	—	—	—	X	—	—	—	—	X	—	X	—	X	—	X	X	—	—	

Dark grey shading with hashes = visits not applicable to Group 2

Light grey shading = visits not required for designated CRS.

v = 1mL of serum will be taken out of serum storage to perform HIV Ab avidity assays; no separate blood draw is needed

w = 0.5mL of serum will be taken out of storage to perform GM-CSF assays; no separate blood draw is needed

y = Whole blood will be taken from an ACD tube already drawn during PBMC processing of Trucount; no separate blood draw is needed.

z = up to 5mL of plasma will be harvested during ACD blood PBMC processing; no separate blood draw is needed

[1] CSR = central specimen repository

[2] HVTN Laboratory Program includes laboratories at UW-VSL, Duke, and FHCRC. UW-VSL = University of Washington Virology Specialty Laboratory (Seattle, Washington, USA); Duke = Duke University Medical Center (Durham, North Carolina, USA); FHCRC = Fred Hutchinson Cancer Research Center (Seattle, Washington, USA)

Non-HVTN laboratories include: CCHRF=Cincinnati Children's Hospital Research Foundation (Cincinnati, OH, USA)

[3] Cardiac troponin to be drawn if clinically indicated.

[4] Screening may occur over the course of several contacts/visits up to and including day 0 prior to vaccination.

[5] Local labs may assign appropriate alternative tube types for locally performed tests.

[6] Chemistry panels are defined in section 9.2 (pre-enrollment) and section 9.4 (postenrollment)

[7] Based on the number of responders observed at the primary immunogenicity time points (visits 2 (only for binding Ab assay) 17, and 21), lab assays may be performed on all participants for humoral and cellular responses at other time points

[8] Genotyping may be performed on enrolled participants using cryopreserved PBMC collected at baseline, initially in participants who demonstrate vaccine-induced T-cell responses at post-vaccination time points.

[9] Pregnancy tests may be performed on blood specimens.

[10] At an early termination visit for a withdrawn or terminated participant (see section 9.11), blood should be drawn for HIV diagnostic testing, as shown for visit 25 above.

[11] Innate Immunity Assays not required for designated CRS.

Appendix I Laboratory procedures for Group 3

					Tube volume (mL)																									
				Visit:	**1**	**2**	**3**	**4**	**5**	**6**	**7**	**8**	**9**	**10**	**11**	**12**	**13**	**14**	**15**	**16**	**17**	**18**	**19**	**20**	**21**	**22**	**23**	**24**	**25**	
				Day:	Screening	D0	D1	D3	D7	D14	D56	D59	D63	D70	D112	D113	D115	D119	D126	D168	D182	D224	D238	**D303**	**D317**	D334	D392	D425	D485	
				Month:	visit[4]	M0			M0.25	M0.5	M2		M2.25	M2.5	M4			M4.25	M4.5	M6	M6.5	M8	M8.5	M10	M10.5	M11	M13	M14	M16	
						**VAC1**					**VAC2**				**VAC3**							**VAC4**								
**Description**	**Ship to[1,2]**	**Assay location[2]**	**Tube[5]**	**Tube size (vol capacity)[5]**		DNA					DNA				MVA							MVA								**Total**
BLOOD COLLECTION																														
Screening or diagnostic assays																														
Screening HIV test	Local lab	Local lab	SST/EDTA	5mL	5	—	—	—	—	—	—	—	—	—	—	—	—	—	—	—	—	—	—	—	—	—	—	—	—	**5**
HBsAg/anti-HCV/Syphilis	Local lab	Local lab	SST	8.5mL	8.5	—	—	—	—	—	—	—	—	—	—	—	—	—	—	—	—	—	—	—	—	—	—	—	—	**8.5**
HIV in-study diagnostic test[10]	UW-VSL	UW-VSL	EDTA	10mL	—	—	—	—	—	—	10	—	—	—	—	—	—	—	10	—	—	—	10	—	—	10	—	20	—	**60**
Safety labs																														
CBC/ Diff/ platelets	Local lab	Local lab	EDTA	5mL	5	5	5	5	5	5	5	5	5	5	5	5	5	5	5	—	—	—	5	—	—	5	—	—	—	**85**
Chemistry Panel[6]	Local lab	Local lab	SST	5mL	5	—	—	—	—	5	—	—	—	5	—	—	—	—	5	—	—	—	5	—	—	5	—	—	—	**30**
Cardiac Troponin[3]	Local lab	Local lab	Hep	5mL	—	—	—	—	—	—	—	—	—	—	—	—	—	—	—	—	—	—	—	—	—	—	—	—	—	**0**
GM-CSF Assays																														
GM-CSF levels	CSR	CCHRF	SST	3.5mL	—	w	—	3.5	3.5	—	3.5	3.5	3.5	—	—	—	—	—	—	—	—	—	—	—	—	—	—	—	—	**17.5**
Anti-GM-CSF binding Ab assay	CSR	CCHRF	SST	3.5mL	—	w	—	—	—	3.5	—	—	—	w	—	—	—	—	w	—	—	—	—	—	—	—	—	—	—	**3.5**
Immunogenicity assays[7]																														
HLA typing[8]	CSR	FHCRC	ACD	8.5mL	—	17	—	—	—	—	—	—	—	—	—	—	—	—	—	—	—	—	—	—	—	—	—	—	—	**17**
Humoral Assays																														
HIV binding Ab assay	CSR	Duke	EDTA	10mL	—	***10***	—	—	—	—	—	—	—	10	—	—	—	—	10	—	—	—	***10***	—	—	—	—	10	—	**50**
HIV neut Ab assay	CSR	Duke	SST	8.5mL	—	8.5	—	—	—	—	—	—	—	8.5	—	—	—	—	8.5	—	—	—	***8.5***	—	—	—	—	8.5	—	**42.5**
HIV Ab avidity assay	CSR	Duke	SST	—	—	v	—	—	—	—	—	—	—	—	—	—	—	—	—	—	—	—	***v***	—	—	v	—	v	—	**0**
Cellular Assays																														
HIV-specific ICS	CSR	FHCRC	ACD	8.5mL	—	34	—	—	—	—	—	—	—	34	—	—	—	—	34	—	—	—	***34***	—	—	—	—	34	—	**170**
Cytokine multiplex bead array	CSR	FHCRC	ACD	8.5mL	—	—	—	—	—	—	—	—	—	—	—	—	—	—	—	—	—	—	***8.5***	—	—	—	—	—	—	**8.5**
Innate Immunity Assays[11]																														
RNA gene expression	CSR	FHCRC	ACD	8.5mL	—	8.5	8.5	8.5	—	8.5	8.5	—	—	8.5	8.5	8.5	8.5	8.5	8.5	—	—	—	—	—	—	—	—	—	—	**93.5**
Trucount	FHCRC	FHCRC	ACD	—	—	y	y	y	—	y	y	—	—	y	y	y	y	y	y	—	—	—	—	—	—	—	—	—	—	**0**
Serum cytokines	CSR	FHCRC	SST	3.5 or 5mL	—	5	3.5	3.5	—	3.5	3.5	—	—	3.5	3.5	3.5	3.5	3.5	3.5	—	—	—	—	—	—	—	—	—	—	**40**
Storage																														
Serum storage (incl ADCC)	CSR	—	SST	5mL or 8.5mL	—	17	5	—	—	—	5	5	5	17	5	5	5	5	8.5	—	—	—	17	—	—	17	—	17	—	**133.5**
Plasma storage	CSR	—	EDTA	10mL	—	20	—	—	—	—	—	—	—	10	—	—	—	—	10	—	—	—	20	—	—	—	—	10	—	**70**
Plasma storage	CSR	—	ACD	—	—	z	z	z	—	z	z	z	z	z	z	z	z	z	z	—	—	—	z	—	—	—	—	z	—	**0**
PBMC storage	CSR	—	ACD	8.5mL	—	68	25.5	25.5	—	25.5	25.5	17	17	51	17	17	17	17	68	—	—	—	59.5	—	—	—	—	51	—	**501.5**
**Maximum Total**					**23.5**	**193**	**47.5**	**46**	**8.5**	**51**	**61**	**30.5**	**30.5**	**152.5**	**39**	**39**	**39**	**39**	**171**	**0**	**0**	**0**	**177.5**	**0**	**0**	**37**	**0**	**150.5**	**0**	1336
**Maximum 56-Day total**					**23.5**	**216.5**	**264**	**310**	**318.5**	**369.5**	**407**	**197**	**181.5**	**325.5**	**313.5**	**230.5**	**117**	**117**	**479.5**	**327**	**171**	**0**	**177.5**	**0**	**0**	**37**	**0**	**150.5**	**0**	
URINE COLLECTION																														
Urinalysis	Local lab	Local lab			X	—	—	—	—	X	—	—	—	—	—	—	—	—	—	—	—	—	X	—	X	—	—	—	—	
Pregnancy test[9]	Local lab	Local lab			X	X	—	—	—	—	X	—	—	—	X	—	—	—	—	X	—	X	—	X	—	X	X	—	—	

Dark grey shading with hashes = visits not applicable to Group 3; Light grey shading = visits not required for designated CRS.
v = 1mL of serum will be taken out of serum storage to perform HIV Ab avidity assays; no separate blood draw is needed
w = 0.5mL of serum will be taken out of storage to perform GM-CSF assays; no separate blood draw is needed
y = Whole blood will be taken from an ACD tube already drawn during PBMC processing of Trucount; no separate blood draw is needed.
z = up to 5mL of plasma will be harvested during ACD blood PBMC processing; no separate blood draw is needed

[1] CSR = central specimen repository

[2] HVTN Laboratory Program includes laboratories at UW-VSL, Duke, and FHCRC. UW-VSL = University of Washington Virology Specialty Laboratory (Seattle, Washington, USA); Duke = Duke University Medical Center (Durham, North Carolina, USA); FHCRC = Fred Hutchinson Cancer Research Center (Seattle, Washington, USA)

Non-HVTN laboratories include: CCHRF = Cincinnati Children's Hospital Research Foundation (Cincinnati, OH, USA)

[3] Cardiac troponin to be drawn if clinically indicated.

[4] Screening may occur over the course of several contacts/visits up to and including day 0 prior to vaccination.

[5] Local labs may assign appropriate alternative tube types for locally performed tests.

[6] Chemistry panels are defined in section 9.2 (pre-enrollment) and section 9.4 (postenrollment)

[7] Based on the number of responders observed at the primary immunogenicity time point (visits 2 (only for binding Ab assay) and 19), lab assays may be performed on all participants for humoral and cellular responses at other time points

[8] Genotyping may be performed on enrolled participants using cryopreserved PBMC collected at baseline, initially in participants who demonstrate vaccine-induced T-cell responses at post-vaccination time points.

[9] Pregnancy tests may be performed on blood specimens.

[10] At an early termination visit for a withdrawn or terminated participant (see section 9.11), blood should be drawn for HIV diagnostic testing, as shown for visit 24 above.

[11] Innate Immunity Assays not required for designated CRS

Appendix J Procedures at HVTN CRS for Group 1

Visit:	1[a]	2	3	4	5	6	7	8	9	10	11	12	13	14	15	16	17	18	19	20	21	22	23	24	25	Post
Day:		D0	D1	D3	D7	D14	D56	D59	D63	D70	D112	D113	D115	D119	D126	D168	D182	D224	D238	D303	D317	D334	D394	D425	D485	
Month:		M0			M0.25	M0.5	M2		M2.25	M2.5	M4			M4.25	M4.5	M6	M6.5	M8	M8.5	M10	M10.5	M11	M13	M14	M16	
**Procedure**	Screening visit	**VAC1**					**VAC2**				**VAC3**					**VAC4**		**VAC5**								
**Study procedures[b]**																										
Signed screening consent (if used)	X	—	—	—	—	—	—	—	—	—	—	—	—	—	—	—	—	—	—	—	—	—	—	—	—	—
Assessment of understanding	X	—	—	—	—	—	—	—	—	—	—	—	—	—	—	—	—	—	—	—	—	—	—	—	—	—
Signed protocol consent	X	—	—	—	—	—	—	—	—	—	—	—	—	—	—	—	—	—	—	—	—	—	—	—	—	—
Medical history	X	—	—	—	—	—	—	—	—	—	—	—	—	—	—	—	—	—	—	—	—	—	—	—	—	—
Complete physical exam	X	—	—	—	—	—	—	—	—	—	—	—	—	—	—	—	—	—	—	—	—	—	—	X	—	—
Abbreviated physical exam	—	X	—	—	X	X	X	—	X	X	X	—	—	—	X	X	X	X	X	—	—	X	—	—	—	—
Risk reduction counseling	X	X	—	—	X	X	X	—	X	X	X	—	—	—	X	X	X	X	X	—	—	X	—	X	—	—
ECG[c]	X	—	—	—	—	—	—	—	—	—	—	—	—	—	—	—	—	—	—	—	—	—	—	—	—	—
Cardiac smyptoms assessment[c, g]	—	—	—	—	—	—	—	—	—	—	X	—	—	—	X	X	X	X	X	—	—	X	—	X	—	—
Pregnancy prevention assessment[d]	X	X	—	—	X	X	X	—	X	X	X	—	—	—	X	X	X	X	X	—	—	X	—	X	—	—
Behavioral risk assessment	X	—	—	—	—	—	—	—	—	—	—	—	—	—	—	—	—	—	—	—	—	—	—	—	—	—
Confirm eligibility, obtain demographics, randomize	X	—	—	—	—	—	—	—	—	—	—	—	—	—	—	—	—	—	—	—	—	—	—	—	—	—
Social impact assessment	—	X	—	—	X	X	X	—	X	X	X	—	—	—	X	X	X	X	X	—	—	X	—	X	—	—
Social impact assessment questionnaire	—	—	—	—	—	—	—	—	—	X	—	—	—	—	—	X	—	—	—	—	—	—	—	X	—	—
Outside testing and belief questionnaire	—	—	—	—	—	—	—	—	—	—	—	—	—	—	—	X	—	—	—	—	—	—	—	X	—	—
Concomitant medications	X	X	—	X	X	X	X	X	X	X	X	—	—	—	X	X	X	X	X	—	—	X	—	X	—	—
Intercurrent illness/adverse experience	—	X	—	X	X	X	X	X	X	X	X	—	—	—	X	X	X	X	X	—	—	X	—	X	—	—
HIV infection assessment[e]	X	—	—	—	—	—	X	—	—	—	—	—	—	—	X	—	—	—	X	—	—	X	—	X	—	—
Confirm HIV test results provided to participant	—	X	—	—	—	—	—	—	X	—	—	—	—	—	—	X	—	—	—	—	—	X	—	X	—	—
**Local lab assessment**																										
Urine dipstick	X	—	—	—	—	X	—	—	—	—	—	—	—	—	—	—	—	—	X	—	—	—	—	—	—	—
Pregnancy (urine or serum HCG)[f]	X	X	—	—	—	—	X	—	—	—	X	—	—	—	—	X	—	X	—	—	—	X	—	—	—	—
CBC, differential, platelet	X	X	—	X	X	X	X	X	X	X	X	—	—	—	X	—	X	—	X	—	—	X	—	—	—	—
Chemistry panel	X	—	—	—	—	X	—	—	—	X	—	—	—	—	X	—	X	—	X	—	—	X	—	—	—	—
Syphilis, Hepatitis B, Hepatitis C	X	—	—	—	—	—	—	—	—	—	—	—	—	—	—	—	—	—	—	—	—	—	—	—	—	—
**Vaccination procedures**																										
Vaccination[h]	—	X	—	—	—	—	X	—	—	—	X	—	—	—	—	X	—	X	—	—	—	—	—	—	—	—
Reactogenicity assessments[i]	—	X	—	—	—	—	X	—	—	—	X	—	—	—	—	X	—	X	—	—	—	—	—	—	—	—
**Poststudy**																										
Unblind participant	—	—	—	—	—	—	—	—	—	—	—	—	—	—	—	—	—	—	—	—	—	—	—	—	—	X

Grayed out columns = visits not applicable for Group 1.

[a] Screening may occur over the course of several contacts/visits up to and including day 0 prior to vaccination.

[b] For specimen collection requirements, see Appendix G.

[c] ECG is required at screening and may also be performed at additional timepoints as clinically indicated.

[d] Pregnancy prevention compliance occurs only with participants who were born female and are capable of becoming pregnant.

[e] Includes pretest counseling. A subsequent follow-up contact is conducted to provide post-test counseling and to report results to participant.

[f] For a participant who was born female, pregnancy test must be performed on the day of vaccination prior to vaccination. Pregnancy test to determine eligibility may be performed at screening or on day 0 prior to first vaccination. Serum pregnancy tests may be used to confirm the results of, or substitute for, a urine pregnancy test.

[g] Cardiac troponin and CK-MB to be drawn if clinically indicated

[h] Blood draws required at vaccination visits must be performed prior to administration of study product; however, it is not necessary to have results prior to administration. Lab tests may be drawn within the 3 days prior to vaccination.

[i] Reactogenicity assessments performed daily for at least 3 days postvaccination (see section 9.9)

Appendix K Procedures at HVTN CRS for Group 2

Visit:	1[a]	2	3	4	5	6	7	8	9	10	11	12	13	14	15	16	17	18	19	20	21	22	23	24	25	Post
Day:		D0	D1	D3	D7	D14	D56	D59	D63	D70	D112	D113	D115	D119	D126	D168	D182	D224	D238	D303	D317	D334	D394	D425	D485	
Month:		M0			M0.25	M0.5	M2		M2.25	M2.5	M4			M4.25	M4.5	M6	M6.5	M8	M8.5	M10	M10.5	M11	M13	M14	M16	
**Procedure**	Screening visit	**VAC1**					**VAC2**				**VAC3**					**VAC4**				**VAC5**						
**Study procedures**[b]																										
Signed screening consent (if used)	X	—	—	—	—	—	—	—	—	—	—	—	—	—	—	—	—	—	—	—	—	—	—	—	—	—
Assessment of understanding	X	—	—	—	—	—	—	—	—	—	—	—	—	—	—	—	—	—	—	—	—	—	—	—	—	—
Signed protocol consent	X	—	—	—	—	—	—	—	—	—	—	—	—	—	—	—	—	—	—	—	—	—	—	—	—	—
Medical history	X	—	—	—	—	—	—	—	—	—	—	—	—	—	—	—	—	—	—	—	—	—	—	—	—	—
Complete physical exam	X	—	—	—	—	—	—	—	—	—	—	—	—	—	—	—	—	—	—	—	—	—	—	—	X	—
Abbreviated physical exam	—	X	—	—	X	X	X	—	X	X	X	—	—	X	X	X	X	—	—	X	X	—	X	—	—	—
Risk reduction counseling	X	X	—	—	X	X	X	—	X	X	X	—	—	X	X	X	X	—	—	X	X	—	X	—	X	—
ECG[c]	X	—	—	—	—	—	—	—	—	—	—	—	—	—	—	—	—	—	—	—	—	—	—	—	—	—
Cardiac smyptoms assessment[c, g]	—	—	—	—	—	—	—	—	—	—	X	—	—	—	X	X	X	—	—	X	X	—	X	—	X	—
Pregnancy prevention assessment[d]	X	X	—	—	X	X	X	—	X	X	X	—	—	X	X	X	X	—	—	X	X	—	X	—	X	—
Behavioral risk assessment	X	—	—	—	—	—	—	—	—	—	—	—	—	—	—	—	—	—	—	—	—	—	—	—	—	—
Confirm eligibility, obtain demographics, randomize	X	—	—	—	—	—	—	—	—	—	—	—	—	—	—	—	—	—	—	—	—	—	—	—	—	—
Social impact assessment	—	X	—	—	X	X	X	—	X	X	X	—	—	X	X	X	X	—	—	X	X	—	X	—	X	—
Social impact assessment questionnaire	—	—	—	—	—	—	—	—	—	X	—	—	—	—	—	X	—	—	—	—	—	—	—	—	X	—
Outside testing and belief questionnaire	—	—	—	—	—	—	—	—	—	—	—	—	—	—	—	X	—	—	—	—	—	—	—	—	X	—
Concomitant medications	X	X	X	X	X	X	X	X	X	X	X	X	X	X	X	X	X	—	—	X	X	—	X	—	X	—
Intercurrent illness/adverse experience	—	X	X	X	X	X	X	X	X	X	X	X	X	X	X	X	X	—	—	X	—	—	X	—	X	—
HIV infection assessment[e]	X	—	—	—	—	—	X	—	—	—	—	—	—	—	X	—	X	—	—	—	X	—	X	—	X	—
Confirm HIV test results provided to participant	—	X	—	—	—	—	—	—	X	—	—	—	—	—	—	X	—	—	—	X	—	—	X	—	X	—
**Local lab assessment**																										
Urine dipstick	X	—	—	—	—	X	—	—	—	—	—	—	—	—	—	—	—	—	—	—	X	—	—	—	—	—
Pregnancy (urine or serum HCG)[f]	X	X	—	—	—	—	X	—	—	—	X	—	—	—	—	X	—	—	—	X	—	—	X	—	—	—
CBC, differential, platelet	X	X	X	X	X	X	X	X	X	X	X	X	X	X	X	—	X	—	—	—	X	—	X	—	—	—
Chemistry panel	X	—	—	—	—	X	—	—	—	X	—	—	—	—	X	—	X	—	—	—	X	—	X	—	—	—
Syphilis, Hepatitis B, Hepatitis C	X	—	—	—	—	—	—	—	—	—	—	—	—	—	—	—	—	—	—	—	—	—	—	—	—	—
**Vaccination procedures**																										
Vaccination[h]	—	X	—	—	—	—	X	—	—	—	X	—	—	—	—	X	—	—	—	X	—	—	—	—	—	—
Reactogenicity assessments[i]	—	X	—	—	—	—	X	—	—	—	X	—	—	—	—	X	—	—	—	X	—	—	—	—	—	—
**Poststudy**																										
Unblind participant	—	—	—	—	—	—	—	—	—	—	—	—	—	—	—	—	—	—	—	—	—	—	—	—	—	X

Dark gray columns = visits not applicable for Group 2

Light gray columns = visits not required for designated CRS.

[a] Screening may occur over the course of several contacts/visits up to and including day 0 prior to vaccination.

[b] For specimen collection requirements, see Appendix H

[c] ECG is required at screening and may also be performed at additional timepoints as clinically indicated.

[d] Pregnancy prevention compliance occurs only with participants who were born female and are capable of becoming pregnant.

[e] Includes pretest counseling. A subsequent follow-up contact is conducted to provide post-test counseling and to report results to participant.

[f] For a participant who was born female, pregnancy test must be performed on the day of vaccination prior to vaccination. Pregnancy test to determine eligibility may be performed at screening or on day 0 prior to first vaccination. Serum pregnancy tests may be used to confirm the results of, or substitute for, a urine pregnancy test.

[g] Cardiac troponin and CK-MB to be drawn if clinically indicated

[h] Blood draws required at vaccination visits must be performed prior to administration of study product; however, it is not necessary to have results prior to administration. Lab tests may be drawn within the 3 days prior to vaccination.

[i] Reactogenicity assessments performed daily for at least 3 days postvaccination (see section 9.9)

Appendix L Procedures at HVTN CRS for Group 3

Visit:	1[a]	2	3	4	5	6	7	8	9	10	11	12	13	14	15	16	17	18	19	20	21	22	23	24	25	Post
Day:		D0	D1	D3	D7	D14	D56	D59	D63	D70	D112	D113	D115	D119	D126	D168	D182	D224	D238	D303	D317	D334	D394	D425	D485	
Month:		M0			M0.25	M0.5	M2		M2.25	M2.5	M4			M4.25	M4.5	M6	M6.5	M8	M8.5	M10	M10.5	M11	M13	M14	M16	
**Procedure**	Screening visit	**VAC1**					**VAC2**				**VAC3**							**VAC4**								
**Study procedures[b]**																										
Signed screening consent (if used)	X	—	—	—	—	—	—	—	—	—	—	—	—	—	—	—	—	—	—	—	—	—	—	—	—	—
Assessment of understanding	X	—	—	—	—	—	—	—	—	—	—	—	—	—	—	—	—	—	—	—	—	—	—	—	—	—
Signed protocol consent	X	—	—	—	—	—	—	—	—	—	—	—	—	—	—	—	—	—	—	—	—	—	—	—	—	—
Medical history	X	—	—	—	—	—	—	—	—	—	—	—	—	—	—	—	—	—	—	—	—	—	—	—	—	—
Complete physical exam	X	—	—	—	—	—	—	—	—	—	—	—	—	—	—	—	—	—	—	—	—	—	—	X	—	—
Abbreviated physical exam	—	X	—	—	X	X	X	—	X	X	X	—	—	X	X	—	—	X	X	—	—	X	—	—	—	—
Risk reduction counseling	X	X	—	—	X	X	X	—	X	X	X	—	—	X	X	—	—	X	X	—	—	X	—	X	—	—
ECG[c]	X	—	—	—	—	—	—	—	—	—	—	—	—	—	—	—	—	—	—	—	—	—	—	—	—	—
Cardiac smyptoms assessment[c, g]	—	—	—	—	—	—	—	—	—	—	X	—	—	—	X	—	—	X	X	—	—	X	—	X	—	—
Pregnancy prevention assessment[d]	X	X	—	—	X	X	X	—	X	X	X	—	—	X	X	—	—	X	X	—	—	X	—	X	—	—
Behavioral risk assessment	X	—	—	—	—	—	—	—	—	—	—	—	—	—	—	—	—	—	—	—	—	—	—	—	—	—
Confirm eligibility, obtain demographics, randomize	X	—	—	—	—	—	—	—	—	—	—	—	—	—	—	—	—	—	—	—	—	—	—	—	—	—
Social impact assessment	—	X	—	—	X	X	X	—	X	X	X	—	—	X	X	—	—	X	X	—	—	X	—	X	—	—
Social impact assessment questionnaire	—	—	—	—	—	—	—	—	—	X	—	—	—	—	—	—	—	—	—	—	—	—	—	X	—	—
Outside testing and belief questionnaire	—	—	—	—	—	—	—	—	—	—	—	—	—	—	—	—	—	—	—	—	—	—	—	X	—	—
Concomitant medications	X	X	X	X	X	X	X	X	X	X	X	X	X	X	X	—	—	X	X	—	—	X	—	X	—	—
Intercurrent illness/adverse experience	—	X	X	X	X	X	X	X	X	X	X	X	X	X	X	—	—	X	X	—	—	X	—	X	—	—
HIV infection assessment[e]	X	—	—	—	—	—	X	—	—	—	—	—	—	—	X	—	—	—	X	—	—	X	—	X	—	—
Confirm HIV test results provided to participant	—	X	—	—	—	—	—	—	X	—	—	—	—	—	—	—	—	X	—	—	—	X	—	X	—	—
**Local lab assessment**																										
Urine dipstick	X	—	—	—	—	X	—	—	—	—	—	—	—	—	—	—	—	—	X	—	—	—	—	—	—	—
Pregnancy (urine or serum HCG)[f]	X	X	—	—	—	—	X	—	—	—	X	—	—	—	—	—	—	X	—	—	—	X	—	—	—	—
CBC, differential, platelet	X	X	X	X	X	X	X	X	X	X	X	X	X	X	X	—	—	—	X	—	—	X	—	—	—	—
Chemistry panel	X	—	—	—	—	X	—	—	—	X	—	—	—	—	X	—	—	—	X	—	—	X	—	—	—	—
Syphilis, Hepatitis B, Hepatitis C	X	—	—	—	—	—	—	—	—	—	—	—	—	—	—	—	—	—	—	—	—	—	—	—	—	—
**Vaccination procedures**																										
Vaccination[h]	—	X	—	—	—	—	X	—	—	—	X	—	—	—	—	—	—	X	—	—	—	—	—	—	—	—
Reactogenicity assessments[i]	—	X	—	—	—	—	X	—	—	—	X	—	—	—	—	—	—	X	—	—	—	—	—	—	—	—
**Poststudy**																										
Unblind participant	—	—	—	—	—	—	—	—	—	—	—	—	—	—	—	—	—	—	—	—	—	—	—	—	—	X

Dark gray columns = visits not applicable for Group 3.
Light gray columns = visits not required for designated CRS.
[a] Screening may occur over the course of several contacts/visits up to and including day 0 prior to vaccination.
[b] For specimen collection requirements, see Appendix I.
[c] ECG is required at screening and may also be performed at additional timepoints as clinically indicated.
[d] Pregnancy prevention compliance occurs only with participants who were born female and are capable of becoming pregnant.
[e] Includes pretest counseling. A subsequent follow-up contact is conducted to provide post-test counseling and to report results to participant.
[f] For a participant who was born female, pregnancy test must be performed on the day of vaccination prior to vaccination. Pregnancy test to determine eligibility may be performed at screening or on day 0 prior to first vaccination. Serum pregnancy tests may be used to confirm the results of, or substitute for, a urine pregnancy test.
[g] Cardiac troponin and CK-MB to be drawn if clinically indicated
[h] Blood draws required at vaccination visits must be performed prior to administration of study product; however, it is not necessary to have results prior to administration. Lab tests may be drawn within the 3 days prior to vaccination.
[i] Reactogenicity assessments performed daily for at least 3 days postvaccination (see section 9.9)

Appendix M Procedures at CRS for annual health contacts

Contact[a] Day	728	1092
Month	24	36
**Procedures**		
Vital status and health events[b]	X	X

[a] Clinic visits are not required, except that any participant reporting a diagnosis of HIV infection will be asked to come to the clinic so that HIV status can be confirmed.
[b] see section 9.5.

Appendix N Case definition of myo/pericarditis for use in adverse events monitoring

Myo/pericarditis

Myo/pericarditis is defined as a spectrum of disease caused by inflammation of the myocardium and/or pericardium. Patients might have symptoms and signs consistent with myocarditis, pericarditis, or both. For the purpose of surveillance reporting, patients with myocarditis or pericarditis will be reported as having myo/pericarditis. These categories are intended for surveillance purposes and not for use in individual diagnosis or treatment decisions.

Case Definition for Acute Myocarditis

A suspected case of acute myocarditis is defined by the following criteria and the absence of evidence of any other likely cause of symptoms or findings below:

- Presence of dyspnea, palpitations, or chest pain of probable cardiac origin in a patient with either one of the following:
  - — Electrocardiogram (ECG) abnormalities beyond normal variants, not documented previously, including
    - ST-segment or T-wave abnormalities,
    - Paroxysmal or sustained atrial or ventricular arrhythmias,
    - AV nodal conduction delays or intraventricular conduction defects, or
    - Continuous ambulatory electrocardiographic monitoring that detects frequent atrial or ventricular ectopy

    or
  - — Evidence of focal or diffuse depressed left-ventricular (LV) function of indeterminate age identified by an imaging study (e.g., echocardiography or radionuclide ventriculography).

A probable case of acute myocarditis, in addition to the above symptoms and in the absence of evidence of any other likely cause of symptoms, has one of the following:

- Elevated cardiac enzymes, specifically, abnormal levels of cardiac troponin I, troponin T, or creatine kinase myocardial band (a troponin test is preferred);
- Evidence of focal or diffuse depressed LV function identified by an imaging study (e.g., echocardiography or radionuclide ventriculography) that is documented to be of new onset or of increased degree of severity (in the absence of a previous study, findings of depressed LV function are considered of new onset if, on follow-up studies, these findings resolve, improve, or worsen); or
- Abnormal result of cardiac radionuclide imaging (e.g., cardiac MRI with gadolinium or gallium-67 imaging) indicating myocardial inflammation.

A case of acute myocarditis is confirmed if histopathologic evidence of myocardial inflammation is found at endomyocardial biopsy or autopsy.

Case Definition for Acute Pericarditis

A suspected case of acute pericarditis is defined by the presence of

- Typical chest pain (i.e., pain made worse by lying down and relieved by sitting up and/or leaning forward) and
- No evidence of any other likely cause of such chest pain.

A probable case of acute pericarditis is a suspected case of pericarditis, or a case in a person with pleuritic or other chest pain not characteristic of any other disease, that, in addition, has one or more of the following:

- Pericardial rub, an auscultatory sign with one to three components per beat,
- ECG with diffuse ST-segment elevations or PR depressions without reciprocal ST depressions that are not previously documented, or
- Echocardiogram indicating the presence of an abnormal collection of pericardial fluid (e.g., anterior and posterior pericardial effusion or a large posterior pericardial effusion alone).

A case of acute pericarditis is confirmed if histopathologic evidence of pericardial inflammation is evident from pericardial tissue obtained at surgery or autopsy.

Source: MMWR 2003; 52 (21); 492-496

Appendix O AESI in HVTN 094

AESI for this protocol are autoimmune disorders. Representative examples of AESI include, but are not limited to the disorders on this list. Updates to AESI will be provided as an appendix to the *HVTN 094 Study Specific Procedures*.

Neuroinflammatory disorders
Optic neuritis
Multiple sclerosis
Acute disseminated encephalomyelitis (ADEM)
Myelitis/transverse myelitis
Encephalitis
Guillain Barre syndrome
Paresthesia (non-specific)
Uveitis
Demyelinating disease
Myasthenia gravis
Neuritis
Bell's palsy/facial palsy
Hypoaesthesia (non-specific)

Musculoskeletal and connective tissue diseases
Rheumatoid arthritis
Juvenile rheumatoid arthritis
Cutaneous lupus
Polymyositis
Psoriatic arthritis
Scleroderma
Temporal arteritis
Reactive arthritis
Systemic lupus erythematosus
Dermatomyositis
Polymyalgia rheumatica
Ankylosing spondylitis
Sjögren's syndrome
Wegener's granulomatosis
Anti-neutrophil cytoplasmic autoantibody(ANCA)-associated vasculitis
Mixed connective tissue disease
Mono-articular arthritis
Arthritis/bursitis (non-specified)
Sarcoidosis
Behçet's syndrome
Pauci-Articular arthritis

Infection and infestations
Fever of unknown origin

Gastrointestinal disorders
Crohn's disease
Celiac disease
Inflammatory bowel disease (non-specific)
Ulcerative colitis
Ulcerative proctitis

Immune system disorders
Idiopathic thrombocytopenic purpura (ITP)
Autoimmune hepatitis
Autoimmune hemolytic anemia
Anti-ds DNA increased
Anti-phospholipid syndrome
Autoimmune glomerulonephritis
Antinuclear antibody(ANA) levels increased
Serum sickness

Thyroid disorders
Grave's disease
Basedow's syndrome (Grave's disease)
Hyperthyroidism
Thyroid-stimulating hormone (TSH) levels high
Thyroiditis
Goiter (non-specified)
Hypothyroidism
Thyroid disease (non-specific)

Renal and urinary disorders
Nephritis
Hematuria
Renal failure
Proteinuria
Glomerulonephritis

Skin disorders
Psoriasis and psoriatic arthropathy
Erythema
Nodosum
Dermatomyositis
Alopecia
Stevens-Johnson's syndrome
Vitiligo

Vascular disorders
Raynaud's phenomena
Vasculitis

Other disorders
Carditis
Pericarditis
Insulin-dependent diabetes mellitus (IDDM)
Myocarditis
Pulmonary embolism
