# Supplementary material for: Immunogenicity of a novel Clade B HIV-1 vaccine combination: Results of phase 1 randomized placebo controlled trial of an HIV-1 GM-CSF-expressing DNA prime with a modified vaccinia Ankara vaccine boost in healthy HIV-1 uninfected adults
Source: PLoS One. 2017 Jul 20;12(7):e0179597. doi: 10.1371/journal.pone.0179597 (PMC5519050; doi:10.1371/journal.pone.0179597)
Supplement: S1 Table — (DOCX) [file pone.0179597.s003.docx]

**Table S1: Demographics and Vaccination Frequencies**

|  | **Placebo**  **(N=8)** | **1/10 DgDgMM_M**  **(N=10)** | **DgDgMM_M**  **(N=16)** | **DgDgM_M**  **(N=14)** | **Total**  **(N=48)** |
| --- | --- | --- | --- | --- | --- |
| **Sex** |  |  |  |  |  |
| **Male** | **3 (38%)** | **4 (40%)** | **2 (13%)** | **9 (64%)** | **18 (38%)** |
| **Female** | **5 (63%)** | **6 (60%)** | **14 (88%)** | **5 (36%)** | **30 (63%)** |
|  |  |  |  |  |  |
| **Race** |  |  |  |  |  |
| **White – non-Hispanic** | **5 (63%)** | **7 (70%)** | **11 (69%)** | **7 (50%)** | **30 (63%)** |
| **Black/African American – non-Hispanic** | **1 (13%)** | **2 (20%)** | **0 (0%)** | **2 (14%)** | **5 (10%)** |
| **Hispanic** | **1 (13%)** | **0 (0%)** | **2 (13%)** | **3 (21%)** | **6 (13%)** |
| **Asian** | **0 (0%)** | **1 (10%)** | **1 (6%)** | **2 (14%)** | **4 (8%)** |
| **Native Hawaiian/Pacific Islander** | **0 (0%)** | **0 (0%)** | **0 (0%)** | **0 (0%)** | **0 (0%)** |
| **Native American/Alaskan Native** | **0 (0%)** | **0 (0%)** | **0 (0%)** | **0 (0%)** | **0 (0%)** |
| **Multiracial** | **1 (13%)** | **0 (0%)** | **2 (13%)** | **0 (0%)** | **3 (6%)** |
| **Other** | **0 (0%)** | **0 (0%)** | **0 (0%)** | **0 (0%)** | **0 (0%)** |
|  |  |  |  |  |  |
| **Age (Years)** |  |  |  |  |  |
| **Less than 18** | **0 (0%)** | **0 (0%)** | **0 (0%)** | **0 (0%)** | **0 (0%)** |
| **18 – 20** | **0 (0%)** | **2 (20%)** | **3 (19%)** | **3 (21%)** | **8 (17%)** |
| **21 – 30** | **6 (75%)** | **7 (70%)** | **9 (56%)** | **8 (57%)** | **30 (63%)** |
| **31 – 40** | **2 (25%)** | **1 (10%)** | **2 (13%)** | **3 (21%)** | **8 (17%)** |
| **41 – 50** | **0 (0%)** | **0 (0%)** | **2 (13%)** | **0 (0%)** | **2 (4%)** |
| **Over 50** | **0 (0%)** | **0 (0%)** | **0 (0%)** | **0 (0%)** | **0 (0%)** |
|  |  |  |  |  |  |
| **Median** | **24.5** | **23.0** | **25.5** | **26.0** | **25.0** |
| **Range** | **21 – 33** | **19 – 35** | **19 – 45** | **19 – 35** | **19 – 45** |
|  |  |  |  |  |  |
| **Vaccination Frequencies** |  |  |  |  |  |
| **Day 0** | **8 (100%)** | **10 (100%)** | **16 (100%)** | **14 (100%)** | **48 (100%)** |
| **Day 56** | **8 (100%)** | **10 (100%)** | **15 (94%)** | **13 (93%)** | **46 (96%)** |
| **Day 112** | **8 (100%)** | **9 (90%)** | **15 (94%)** | **13 (93%)** | **45 (94%)** |
| **Day 168** | **3 (75%)** | **9 (90%)** | **14 (88%)** |  | **26 (87%) (1)** |
| **Day 224** | **5 (83%)** | **9 (90%)** |  | **13 (93%)** | **27 (90%) (2)** |
| **Day 303** | **2 (100%)** |  | **13 (81%)** |  | **15 (83%) (3)** |
| **Notes: Day 168 calculated on a basis of 30 expected participants (2) Day 224 calculated on a basis of 30 expected participants (3) Day 303 calculated on a basis of 18 expected participants** | | | | | |
